# Nicotinonitrile based dual inhibitors of tubulin and topoisomerase II exhibit potent anticancer activity

**DOI:** 10.1038/s41598-026-47995-5

**Published:** 2026-06-25

**Authors:** Eman S. Hassan, Abdalla E. A. Hassan, Shaikha S. Al Neyadi, Yasir S. Raouf, Hanem M. Awad, Zakaria K. M. Abdel-Samii, Amany M. M. Al-Mahmoudy, Reham A I. Abou-Elkhair

**Affiliations:** 1 Applied Nucleic Acids Research center, Zagazig University, Zagazig, Egypt; 2 Department of Chemistry, Faculty of Science, Zagazig University, Zagazig, Egypt; 3 Department of Pharmaceutical Organic Chemistry, Faculty of Pharmacy, Zagazig University, Zagazig, Egypt; 4 Department of Chemistry, College of Science, United Arab Emirates University, P.O. Box 15551, Al Ain, UAE; 5 Tanning Materials & Leather Technology Department, National Research Centre, Dokki, Giza 12622 Egypt

**Keywords:** 2,4,6-trisubstituted nicotinonitriles, Azido/tetrazolo nicotinonitriles. Iminophosphorane, Tubulin polymerization inhibitors, Topoisomerase II inhibitors, Combretastatin (A-4), Biochemistry, Cancer, Chemical biology, Chemistry, Drug discovery

## Abstract

A series of 2,4,6-trisubstituted nicotinonitriles, compounds **10**–**41**, was designed as pyridine bridged analogs of combretastatin A4 and evaluated as dual inhibitors of topoisomerase II and tubulin polymerization. Anticancer activity was tested in MCF7, HepG2, and HCT116 cells using the LDH assay. Several compounds including **19**–**24**, **26**–**27**, **33**–**35**, **37**–**38**, and **41** showed strong cytotoxicity in MCF7 cells, while compounds **20**, **26**, **38**, **39**, and **41** displayed moderate activity in HepG2 cells with good selectivity toward BJ1 normal cells. Tubulin polymerization assays identified compounds **20**, **26**, and **37** as the most active, showing inhibition values of 74.7%, 75.0%, and 74.3%, compared with 72.1% for combretastatin A4. Compound **37** showed strong topoisomerase II inhibition (82.4%), while compound **20** displayed moderate inhibition (70.3%), both compared with DOX (81.6%). Cell cycle analysis indicated that compounds **20** and **26** induced G2 and M phase arrest in MCF7 cells and promoted apoptosis. Molecular docking confirmed favorable binding interactions with both tubulin and topoisomerase II. These results highlight 2,4,6-trisubstituted nicotinonitriles as promising dual target anticancer candidates and support their further optimization.

**Supplementary Information:**

The online version contains supplementary material available at 10.1038/s41598-026-47995-5.

## Introduction

Cancer remains one of the leading causes of mortality worldwide, accounting for nearly 10 million deaths per year (approximately one in six deaths)^1^. Global cancer incidence continues to rise, with an estimated 20 million new cases diagnosed in 2022 alone^2^. Beyond the human toll, the economic burden of cancer is enormous, the global cost of cancer is projected to reach about 25 trillion dollars (in 2017 international dollars) over the period 2020–2050. These sobering statistics underscore the urgent need for more effective and innovative anticancer therapies^1^.

**Fig. 1:**
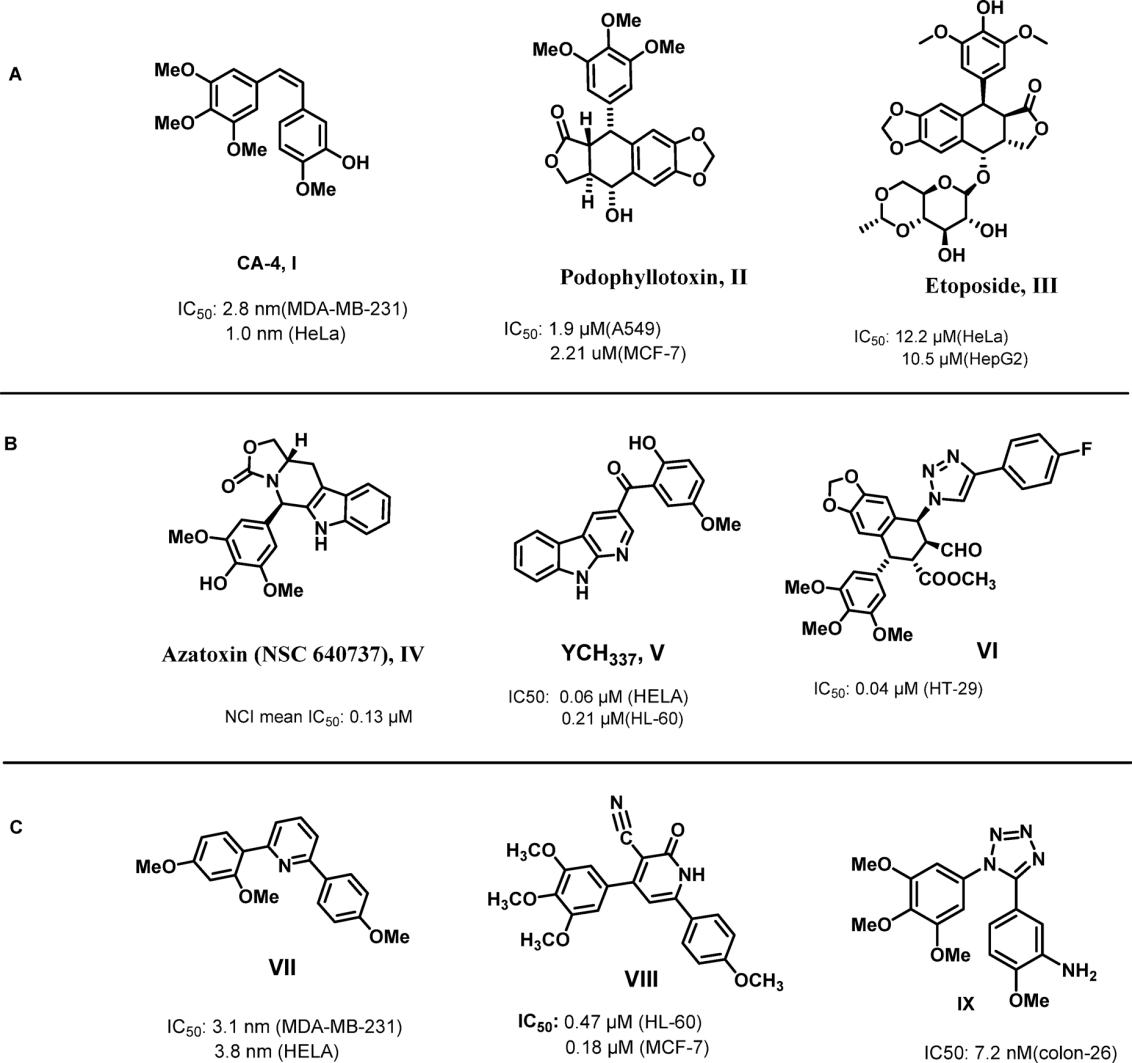
(**A**) Structures of reported tubulin inhibitors anticancer agents. (**B**) Structures of dual tubulin/Topo II inhibitors. (**C**) Structures of heterocycle-bridged CA4 analogues with potent anticancer activity.

Current treatment modalities such as surgery, radiation, chemotherapy, and targeted therapy have improved outcomes for many cancers, yet treatment failures, resistance, and side effects remain significant challenges. Conventional single-target chemotherapeutic agents often face issues such as dose-limiting toxicity and the emergence of drug resistance, prompting the exploration of new strategies in drug design and discovery. By engaging two or more cancer-relevant pathways simultaneously, a multi-target agent can exploit synergistic anticancer effects and potentially overcome resistance mechanisms that would thwart a single-target drug^3^. In this context, dual inhibitors of tubulin polymerization and DNA topoisomerase II (Topo II) are of great therapeutic interest. Both tubulin and Topo II are well-validated targets in oncology, and their inhibition leads to complementary anticancer effects^4^. Microtubules assembled from tubulin are essential for mitosis and cell division. Agents that disrupt tubulin polymerization, so-called microtubule destabilizers, cause cell cycle arrest and apoptosis of rapidly dividing cells^5^. Topo II is an enzyme that relieves DNA supercoiling during replication and transcription; inhibitors of Topo II induce DNA strand breaks and prevent genome replication, which is lethal to proliferating cancer cells^6^. Tubulin inhibitors and Topo II inhibitors are frequently used in combination chemotherapy regimens for synergistic efficacy. This synergism has inspired the design of single molecules capable of concurrently targeting both microtubule assembly and DNA topoisomerase activity^3^. Several examples illustrate the therapeutic value of these target classes and lay the groundwork for dual inhibitors. Combretastatin A4 (CA4), a natural cis-stilbene isolated from Combretum Caffrum, is a potent tubulin polymerization inhibitor that binds to the colchicine site on β-tubulin^5^. CA4 and its analogues cause mitotic arrest and have shown broad anticancer activity, including against multidrug-resistant tumors^7^. However, the clinical application of CA4 is limited by its poor aqueous solubility and instability of its cis-olefin which can isomerize to the less active trans form (Fig. 1A)^5,7^. Podophyllotoxin, a lignan from the mayapple plant, is another anti-mitotic agent that binds tubulin at a site distinct from the vinca alkaloid site and inhibits microtubule assembly (Fig. 1A)^8^. Interestingly, semisynthetic derivatives of podophyllotoxin, etoposide (VP-16) and teniposide (TM-26) were developed to forgo tubulin activity and instead act as Topo II inhibitors^8,9^. Etoposide, a cornerstone of many chemotherapy regimens, intercalates DNA and stabilizes the Topo II–DNA cleavable complex, leading to lethal DNA double-strand breaks in cancer cells (Fig. 1A)^10^. Another relevant example is azatoxin (NSC 640737), a synthetic hybrid molecule encompassing the structural elements of etoposide and ellipticine, displays dual mechanism of action; at lower concentrations it behaves predominantly as a microtubule destabilizer, while at higher concentrations it inhibits Topo II, ultimately inducing both mitotic arrest and DNA damage in cancer cells(Fig. 1B)^11^. Compound **V** and **VI** also have dual activity of Topo II and tubulin inhibition (Fig. 1B)^4,12^.

**Fig. 2:**
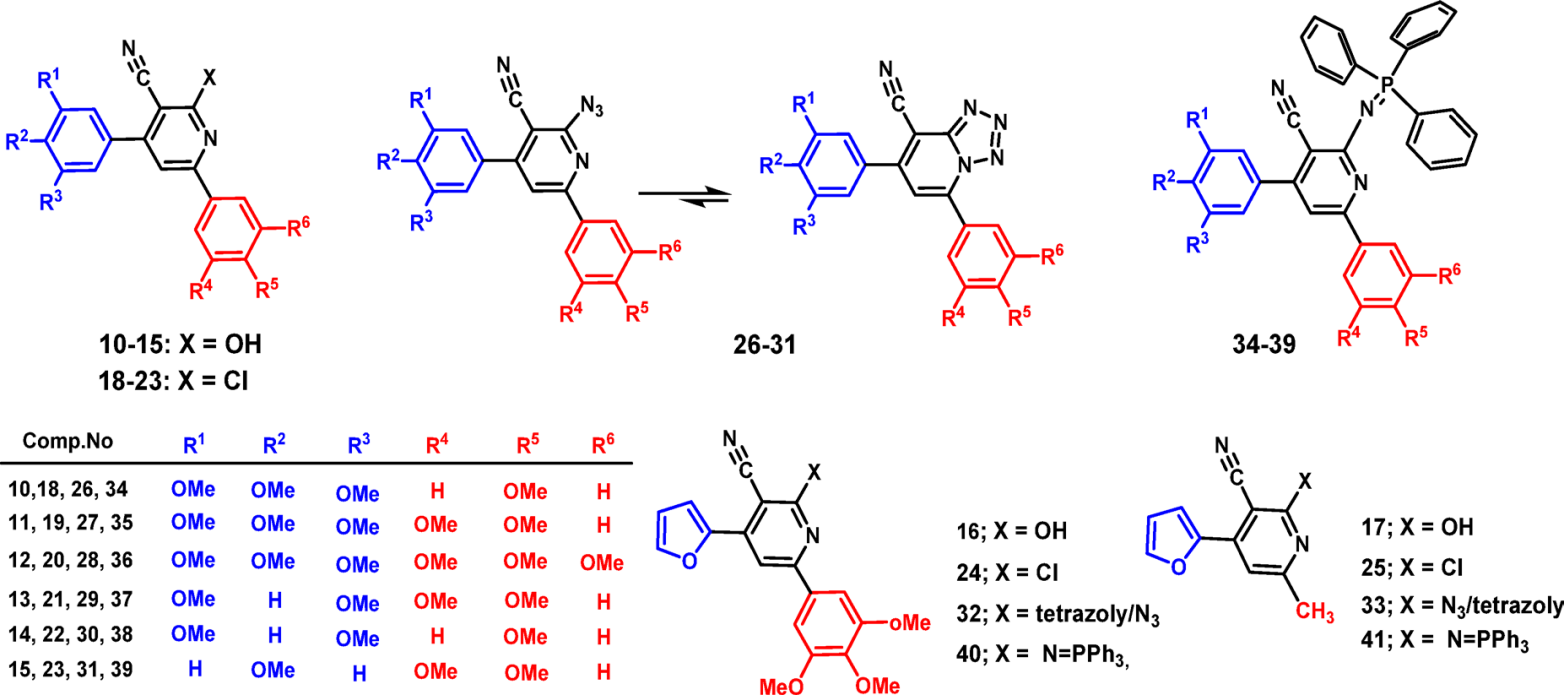
Structures of the synthesized 2,4,6-trisubstituted nicotinonitrile derivatives 10–41.

In this context, 4,6-diarylnicotinonitriles were selected as a promising scaffold due to their rigid, pyridine-bridged structure that mimics the bioactive conformation of CA4 while improving metabolic stability. Figure 1C highlights examples of nicotinonitrile and heterocycle-bridged CA4 analogues with notable anticancer activity, where the cytotoxic potency is strongly influenced by substituents on rings A and B and by the nature of the central linker^13,14^. In this study, we focused on 2,4,6-trisubstituted nicotinonitriles scaffolds that incorporate essential structural elements for dual anti-tubulin and Topo II inhibition, namely, aryl rings (A and B) connected by a rigid pyridine linker. Modifications at the 2-position, including hydroxy, chloro, azido/tetrazolo and iminophosphorane moieties on the central pyridine ring, were introduced to enhance DNA intercalation and promote Topo II inhibitory activity (Fig. 2).

Structure–activity relationship (SAR) studies on CA4 and its analogues have shown that optimal antiproliferative activity depends on the presence of 3,4,5-trimethoxy and/or 3,5-dimethoxy substitutions on ring A, as well as the cis-configuration of the vinyl bridge. In contrast, ring B tolerates various structural changes; the 3-hydroxy group is not essential for activity, whereas the 4-methoxy group is critical for cytotoxicity (Compounds **VII**, **VIII** and **IX**, Fig. 1C)^13–15^. Compounds exhibiting dual inhibition of tubulin and Topo II often feature per-methoxyphenyl groups, extended cyclic systems, and azole rings within their structures (Compounds **IV**,** V**, and **VI**, Fig. 1B)^4,11,12^.

**Fig. 3:**
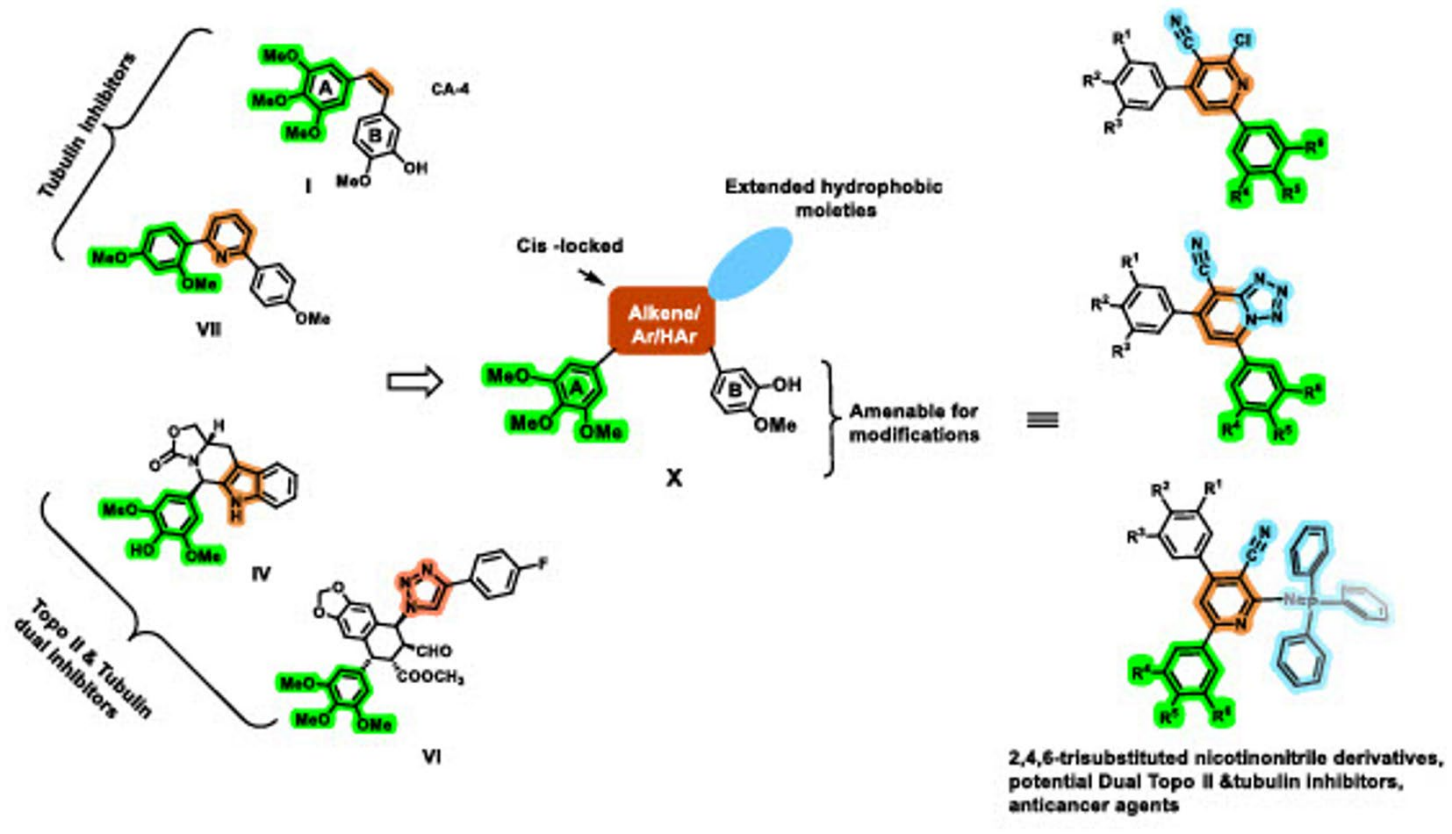
Design of pyridine bridged CA4 analogs.

The design rationale for the hybrid molecules **X**, which incorporate key structural features aimed at dual inhibition of Topo II and tubulin polymerization is illustrated in Fig. 3. Ring A includes varying numbers of methoxy groups that serve as hydrogen bond acceptors within the colchicine binding site of tubulin, along with a cis-double bond, mimicking the rigid pyridine ring. To enhance Topo II binding, the pyridine ring is functionalized at the 2-position with hydrophobic substituents such as chloro, azido/tetrazolo, and iminophosphorane groups, while ring B is substituted with different number of methoxy groups (compounds **18–41**, Fig. 3). Figure 3 summarizes the design of the hybrid molecules, which combine methoxy-substituted aryl rings and hydrophobic groups on the 2-position of the pyridine core to support dual inhibition of Topo II and tubulin.

## Results and discussion

### Chemistry

**Scheme 1:**
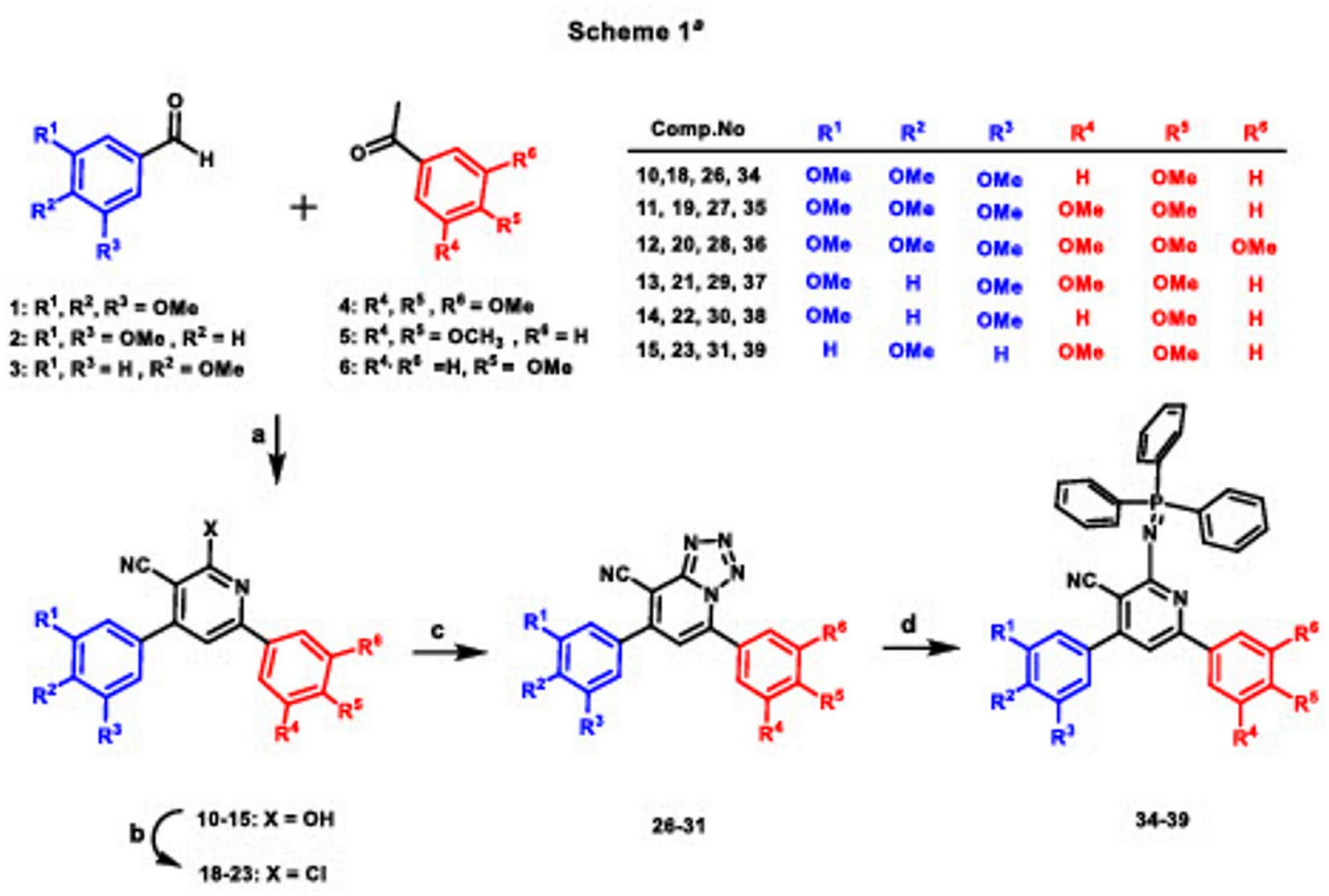
^a^Reagents and conditions: (a) CNCH_2_COOEt, CH_3_COONH_4_, EtOH, 12–14 h, reflux, (b) POCl_3_, *N*,* N-*dimethyl-aniline, 12–16 h, reflux, (c) NaN_3_, DMF, 12 h, 80 °C, (d) PPh_3_, toluene, 15 min., reflux.

**Scheme 2:**
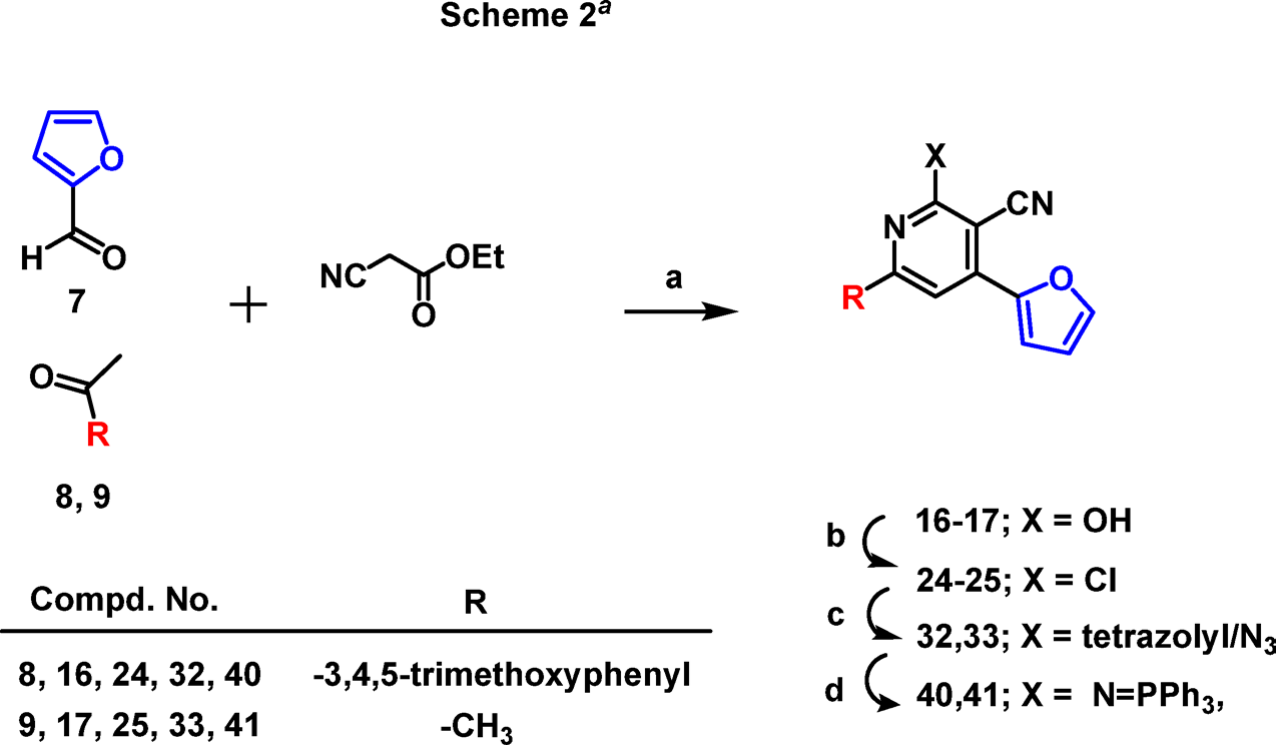
^a^Reagents and conditions. (a) CH_3_COONH_4_, EtOH, 12–14 h, reflux, (b) POCl_3_, *N*,* N*-dimethylaniline, 12–16 h, reflux, (c) NaN_3_, DMF, 12 h, 80 °C, (d) PPh_3_, toluene, 15 min., reflux.

The strategy adopted for the synthesis of the target compounds **10–41** is outlined in scheme 1 and scheme 2. 2-Oxonicotinonitriles (2-ONNs) **10–17** were synthesized by three component-one pot reaction of corresponding aldehydes **1**,** 2**,** 3**, and **7**; corresponding ketones **4**,** 5**,** 6**,** 8**,** 9**; and ethyl cyanoacetate in the presence of ammonium acetate in reflux ethanol^16–18^. An alternative pathway for the synthesis of 2-ONNs involves the reaction of chalcones, generated from the corresponding aldehydes and ketone derivatives, with ethyl cyanoacetate in the presence of ammonium acetate^19,20^. The structure of the 2-ONNs was confirmed by ^1^H-NMR, ^13^C-APT-NMR, ATR-IR spectra. For instance, compound **12** displayed a broad singlet at δ 12.69 ppm corresponding to the NH proton and its^13^ C-APT-NMR spectrum showed signals at *δ* 117.0 and *δ* 162.3 ppm corresponding to the -CN and C = O carbons (Scheme 1). Treatment of the 2-ONNs derivatives **10–17** with POCl_3_ in the presence of *N*,* N*-dimethylaniline at reflux temperature provided the corresponding 2-chloronicotinonitriles **18–25**, respectively in good yields.

Constructing a tetrazolo ring fused at the N-1 and the C-2 position of the pyridine ring envisioned accessible given the fact that nitrogenous six-membered heterocycles with an azido group positioned at the α-position to the ring-nitrogen tend to form a tetrazolo ring depending on the structure and the physical state of the molecule^21,22^. Treatment of the 2-chloronicotinamide derivatives **18–25** with NaN_3_ in DMF at 80 °C provided the corresponding 2-azido/tetrazolo derivatives **26–33**, respectively in good yields. ATR-IR spectra of compounds **26–33** showed variable intensities of the signals at 2130 cm^−1^ (ν_max_/cm^−1^) where is almost absent for compounds **29** and **33** in the solid state. DFT tautomer distribution (azido/tetrazolo) calculation in polar phase revealed the predominance of the tetrazolo-form for compounds **26** and **27**, while the azido-form predominates for compounds **33** (Fig. 6 and supplementary information S4.2.6.1 part). Treatment of the azido/tetrazolo derivatives **26–33** with triphenylphosphine under Staudinger reaction conditions provided the corresponding iminophosphorane derivative **34–41**, respectively in good yields. The 4-furyl derivatives, **16–17**,** 24–25**,** 32–33**, and **40–41** were synthesized using an analogous manner as previously described (Scheme 2).

### Biological evaluations

#### Cytotoxic activity

The cytotoxic activity of the newly synthesized 2-chloronicotinonitrile derivatives **18**–**24**, azido/ tetrazolo derivatives **26–33**, and their corresponding iminophosphorane analogues **34**–**41** was evaluated in vitro against three human cancer cell lines: HepG2 (liver carcinoma), HCT116 (colorectal carcinoma), and MCF7 (breast adenocarcinoma), as well as the normal human skin fibroblast line BJ1 (Table 1). The assays were performed using the LDH method, with doxorubicin (DOX) as the positive control. All tested compounds demonstrated dose-dependent inhibition of the three cancer cell lines, while showing no significant cytotoxicity toward the non-tumor BJ1 cells, in contrast to DOX. The inhibitory concentrations (IC_50_) are summarized in Table 1. The 2-chloropyridine derivative **20** (4,6-bis(3,4,5-trimethoxyphenyl)) exhibited potent activity against the MCF7 cell line (IC_50_ = 2.4 µM, compared with DOX IC_50_ = 2.8 µM) and considerable activity against HepG2 (6.1 µM). In comparison, the 4-(3,5-dimethoxyphenyl)-6-(3,4-dimethoxyphenyl) pyridine derivative **21** retained strong activity against MCF7 (IC_50_ = 2.8 µM), but its potency against HepG2 decreased nearly two-fold (IC_50_ = 12.3 µM). A similar trend was observed with the tetrazolo derivatives, particularly compound **26**, which showed significant inhibition of MCF-7 (IC_50_ = 2.4 µM) and moderate suppression of HepG2 (IC_50_ = 6.3 µM). Consistent with these findings, the iminophosphorane series also displayed structure-dependent activity, with the 4-(3,5-dimethoxyphenyl)-6-(4-methoxyphenyl)pyridine derivative **38** emerging as one of the most active members against MCF7 (IC_50_ = 2.5 µM) and showing moderate potency against HepG2 (12.2 µM). Importantly, the addition of an extra methoxy group at the 3-position of the 6-phenyl ring diminished activity against HepG2 by 2.7-fold, highlighting the sensitivity of activity to subtle substituent modifications. These results clearly demonstrate that structural modifications within this scaffold strongly influence anticancer potency and selectivity. Compounds bearing trimethoxyphenyl groups at the 4- and 6-positions of the nicotinonitrile ring consistently showed enhanced cytotoxic activity, particularly against the MCF7 breast cancer cell line.

**Table 1:** In vitro, cytotoxicity (IC_50_, µM) of compounds **18–24**,** 26–41** against MCF7, HepG2, and HCT116 Cell lines.

Compd. No.		In vitro cytotoxicity IC_50_ µM ± SD	
	MCF7	HepG2	HCT116
18	2.9 ± 0.2	26.2 ± 2.5	45.6 ± 4.1
19	2.7 ± 0.2	24.1 ± 2.2	44.1 ± 4.7
20	2.4 ± 0.1	6.1 ± 0.5	34.4 ± 3.2
21	2.8 ± 0.2	12.3 ± 1.5	48.1 ± 4.2
22	2.6 ± 0.2	25.3 ± 2.3	36.8 ± 3.5
23	2.6 ± 0.2	29.4 ± 2.3	49.1 ± 5.1
24	2.5 ± 0.2	22.7 ± 2.1	49.3 ± 4.2
26	2.4 ± 0.1	6.3 ± 0.3	63.4 ± 5.3
27	2.5 ± 0.2	25.5 ± 2.4	60.1 ± 6.2
28	3.0 ± 0.2	29.9 ± 3.1	78.6 ± 5.9
29	3.1 ± 0.3	27.1 ± 2.5	55.4 ± 5.1
30	3.2 ± 0.2	27.1 ± 2.2	40.5 ± 3.3
31	3.1 ± 0.3	25.7 ± 2.4	31.0 ± 2.5
32	3.0 ± 0.3	28.2 ± 2.5	98.0 ± 8.6
33	2.6 ± 0.2	25.2 ± 2.5	40.7 ± 3.9
34	5.1 ± 0.2	28.4 ± 2.3	49.9 ± 4.2
35	2.7 ± 0.1	32.4 ± 3.1	57.6 ± 4.1
36	3.0 ± 0.1	25.2 ± 2.1	41.7 ± 4.1
37	2.6 ± 0.1	29.3 ± 2.5	36.0 ± 3.3
38	2.5 ± 0.1	12.2 ± 1.1	31.5 ± 3.2
39	3.0 ± 0.1	12.6 ± 1.1	32.5 ± 3.2
40	5.7 ± 0.2	27.7 ± 2.1	55.7 ± 4.9
41	2.4 ± 0.1	27.3 ± 2.3	38.2 ± 2.8
DOX	2.8 ± 0.3	2.6 ± 0.2	12.5 ± 1.5

#### Inhibition of tubulin polymerization in MCF-7 cells

To assess whether the antiproliferative effects of the most active compounds were associated with tubulin disruption, ten derivatives **20**,** 22**,** 24**,** 26**,** 27**,** 33**,** 35**,** 37**,** 38**, and **41** were evaluated for their ability to inhibit tubulin polymerization in MCF7 cells. CA4 was used as a positive control (Table 2). All tested compounds demonstrated significant inhibition of tubulin assembly. Among them, compounds **20**,** 26**,** and 37** showed strong activity, each surpassing CA4 in potency. The remaining derivatives exhibited inhibitory activity comparable to that of CA4. Importantly, there was a clear correlation between the extent of tubulin inhibition and the observed cytotoxicity for most compounds. For instance, **20** and **26**, which were the most potent antiproliferative agents (IC_50_ = 2.4 µM), also showed the highest inhibition of tubulin polymerization (74.7% and 75%, respectively). However, this relationship was not universal. For example, compound **41** exhibited stronger antiproliferative activity than **37** (IC_50_ = 2.4 µM vs. 2.6 µM), yet its effect on tubulin polymerization was weaker (61.2% vs. 74.3%). This indicates that additional mechanisms may contribute to the cytotoxicity of certain derivatives. These findings strongly support tubulin polymerization inhibition as a major mechanism underlying the cytotoxicity of the most active compounds, particularly **20** and **26** derivatives.

**Table 2:** Inhibition of tubulin polymerization (%) and cytotoxic activity (IC_50_, µM) of selected compounds in MCF7 cells.

Compd. No.	tubulin polymerization % inhibition	MCF7 IC_50_ µM ± SD
20	74.7%	2.4 ± 0.1
22	70.9%	2.6 ± 0.2
24	55.4%	2.5 ± 0.2
26	75%	2.4 ± 0.1
27	49.8%	2.5 ± 0.2
33	68.9%	2.6 ± 0.2
35	61.9%	2.7 ± 0.1
37	74.3%	2.6 ± 0.1
38	71.7%	2.5 ± 0.1
41	61.2%	2.4 ± 0.1
CA4	72.1%	-----------

#### Inhibition of topoisomerase II in MCF-7 cells

Following the evaluation of antiproliferative activity and confirmation of tubulin polymerization inhibition, we further examined whether the most active compounds also target topoisomerase II (Topo II), aiming to identify potential dual inhibitors. A selected set of candidates was screened for their ability to inhibit Topo II in vitro in MCF7 cells, with DOX used as a reference standard. Among the tested compounds, the active derivative **37** exhibited the strongest Topo II inhibitory activity, surpassing that of DOX (Table 3). In addition, **20** displayed moderate Topo II inhibition. Taken together, these findings indicate that compounds **20** and **37** act as Topo II inhibitors, while compounds **26**,** 20**,** 37**,** 38**, and **22** function as potent tubulin inhibitors, respectively. Compounds **20** and **37** emerge as dual-acting agents, capable of interfering with both tubulin polymerization and Topo II function. The combined evidence suggests that the antiproliferative activity of this compound series is primarily mediated through tubulin inhibition, but with certain derivatives, particularly **20** and **37**, also exerting strong Topo II inhibition. This dual-target profile highlights these molecules as promising leads for the development of multitarget anticancer agents with enhanced therapeutic potential.

**Table 3:** Inhibition of Topo II (%) and cytotoxic activity (IC_50_, µM) of selected compounds in MCF7 cells.

Compd. No.	Topo II % inhibition	MCF7 IC_50_ µM ± SD
20	70.3%	2.4 ± 0.1
22	42%	2.6 ± 0.2
24	55.3%	2.5 ± 0.2
26	43.7%	2.4 ± 0.1
37	82.4%	2.6 ± 0.1
DOX	81.6%	2.8 ± 0.3

#### In vitro propidium iodide flow cytometry cell cycle analysis

**Fig. 4:**
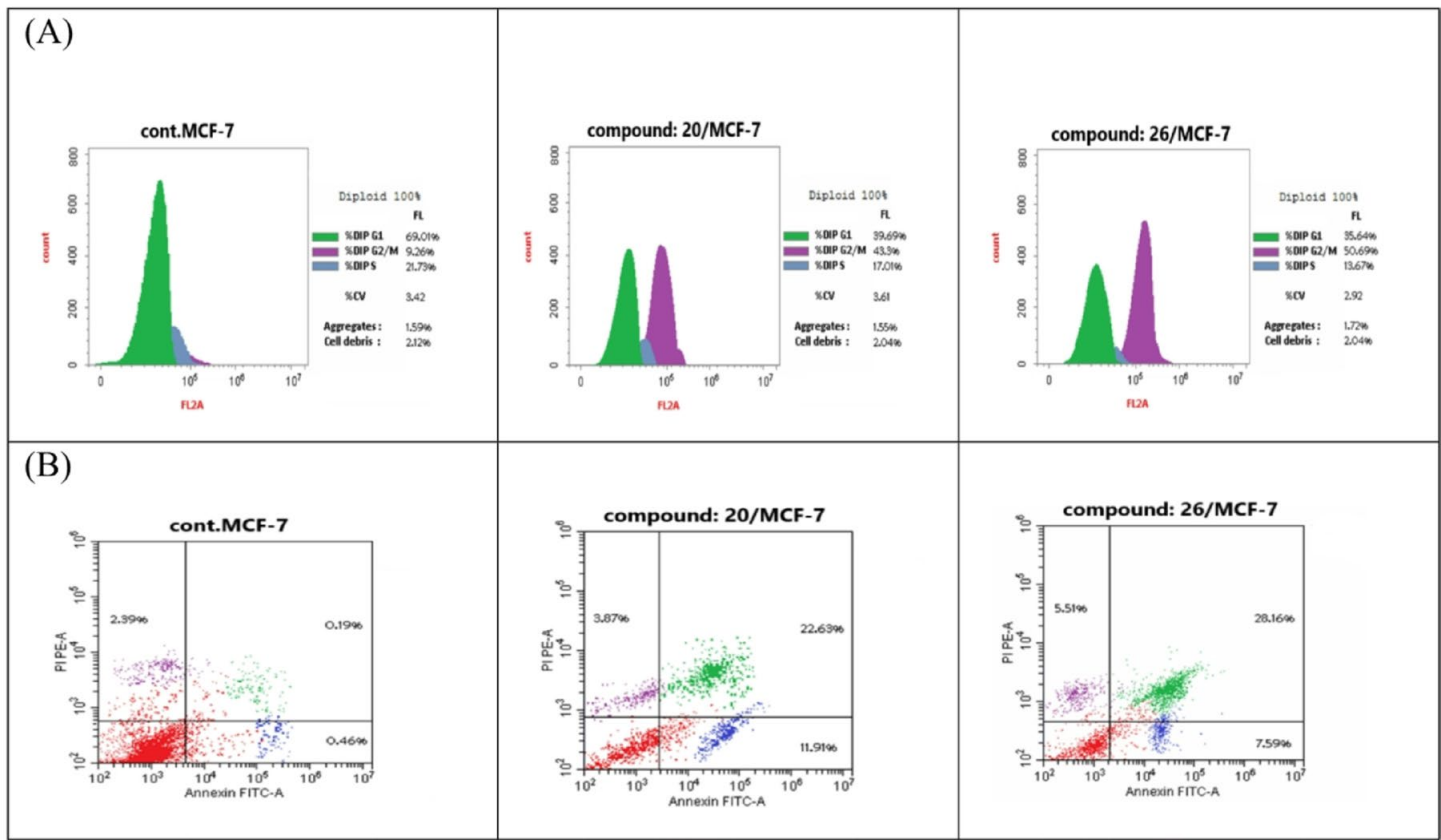
(**A**) Flow cytograms showing DNA distribution profiles of 20 (2.4 µM) and 26 (2.4 µM) treated MCF7 cells in different phases of the cell cycle compared with untreated MCF7 cells. (**B**) Flow cytograms show live and dead cells after Annexin FITC-A/ PI staining. MCF7 cells were incubated without and with 20 and 26 (2.4 and 2.4 µM) for 48 h. Representative images from three experiments are shown.

Arrest of cells in the G2/M phase is a hallmark of both tubulin polymerization inhibitors and topoisomerase II inhibitors. To further elucidate the mechanism of action of the most active derivatives **20** and **26**, we performed cell cycle analysis in MCF7 cells using propidium iodide. flow cytometry (Table 4; Fig. 4a). Compared with untreated MCF7 cells, which displayed a distribution of 69.01% in G0/G1, 21.73% in S, and 9.26% in G2/M phase, treatment with compounds **20** and **26** caused a dramatic accumulation of cells in the G2/M phase. Specifically, the percentage of cells in G2/M increased to 43.3% for **20** and 50.69% for **26**, accompanied by a marked reduction in both G0/G1 and S populations. This arrest profile closely resembled that induced by the reference drugs CA4 (tubulin inhibitor) and DOX (Topo II inhibitor). Taken together, these results confirm that compounds **20** and **26** exert their antiproliferative effects by blocking cell cycle progression at the G2/M phase, consistent with their observed tubulin polymerization inhibition. Compound **20** also exhibited moderate Topo II inhibition, suggesting a dual mechanism of action. The combined evidence indicates that **20** and **26** suppress MCF7 cell proliferation through induction of G2/M arrest, primarily *via* disruption of tubulin dynamics, with **20** additionally engaging Topo II. This dual contribution highlights their potential as multitarget anticancer agents with enhanced efficacy.

**Table 4:** Cell cycle analysis of compounds **20** at 2.4 µM, and **26** at 2.4 µM in MCF7 cell line.

Compd. No.	%G0-G1	%S	%G2/M
20	39.69	17.01	43.3
26	35.64	13.67	50.69
cont. MCF7	69.01	21.73	9.26

#### Apoptosis and necrosis analysis by annexin V-FITC/PI staining

To further elucidate the mechanism underlying the cytotoxicity of the most active compounds **20** and **26**, their ability to induce apoptosis in MCF7 cells was assessed using annexin V-FITC/PI dual staining followed by flow cytometry quantification and fluorescence microscopy detection (Table 5; Fig. 4b). Cells were treated at their respective IC_50_ concentrations, and the distribution of viable, apoptotic, and necrotic populations was analyzed. Compared with untreated control cells, which showed minimal apoptosis (3.04% total apoptotic cells), treatment with compound **20** markedly increased the proportion of apoptotic cells to 38.41% (11.91% early, 22.63% late), while compound **26** induced an even higher apoptotic fraction of 41.26% (7.58% early, 28.16% late). Only minor increases in necrosis were observed (3.87% for 20 and 5.51% for 26), confirming apoptosis as the predominant mechanism of cell death. These findings are consistent with the results of tubulin polymerization and Topo II inhibition studies, as well as cell cycle analysis, where both **20** and **26** induced G2/M arrest in MCF7 cells. The induction of apoptosis through G2/M blockade strongly supports disruption of microtubule dynamics as the primary mechanism of action. Furthermore, the dual activity of **20** on both tubulin and Topo II may account for its slightly distinct apoptotic profile compared with **26**. Compounds **20** and **26** effectively trigger apoptosis in MCF7 cells, in line with their strong antiproliferative activity, inhibition of tubulin polymerization, and induction of G2/M arrest. The data firmly establish apoptosis as the main mode of cell death for these derivatives, underscoring their promise as potent anticancer leads with multitarget potential.

**Table 5:** Apoptosis and necrosis analysis of compounds **20** and **26**.

Compd. No.	Total	Apoptosis Early	Late	Necrosis
20	38.41	11.91	22.63	3.87
26	41.26	7.58	28.16	5.51
cont. MCF7	3.04	0.46	0.19	2.39

### In silico study

#### Aqueous-phase density functional theory supports low energy tetrazolo tautomers

As previously mentioned, heteroaryl azides with an azido-group α to a pyridyl ring nitrogen are known to undergo intramolecular cyclization into tetrazolo tautomers, especially within the context of six-membered heterocycles. To evaluate this behavior in our ligand series, density functional theory (DFT) calculations were run on relevant azido-compounds **26**, **27**, and **33**. All geometries were first optimized using B3LYP/6-31G** in the aqueous phase using an aqueous continuum solvation model (PCM) before higher-level single point energies were run using B3LYP/6–31 + G**, which incorporated relevant diffuse electronic functions, which better described the delocalized nature of the tetrazolo tautomers. From the resulting solution-phase energies (in Hartree), relative Gibbs free energies were obtained for each tautomeric form. Boltzmann distributions (at 298 K) were then used to estimate tautomeric populations. A summary of these results can be found in Fig. 6. For **26**, the tetrazolo tautomer was predicted to be the global minimum, lying approximately ΔG = 1.11 kcal mol^−1^ (4.64 kJ mol^−1^) lower in energy than the corresponding azido form. This relatively large energy difference translated into an estimate equilibrium population of 86.8% (tetrazolo) and only 13.2% (azido), supporting a strong thermodynamic preference for ring-closure in aqueous media. In **27**, a similar but less pronounced trend was observed. The tetrazolo tautomer was stabilized by ΔG = 0.59 kcal mol^−1^ (2.47 kJ mol^−1^) relative to the azido form, corresponding to a population distribution of 72.9% (tetrazolo) compared to 27.1% (azido) under equilibrium conditions. The smaller energy gap suggests that both species may co-exist in solution, although the tetrazolo form remains dominant. Finally, for **33**, the energetics were reversed, revealing the azido-tautomer to be more stable by a value of ΔG = 1.15 kcal mol^−1^ (4.81 kJ mol^−1^). This translated into an estimated population of 12.6% (tetrazolo) and 87.4% (azido), suggesting the open-ring form to be more stable in water. Overall, these results confirm that our compounds do exhibit energetically favorable bias towards the cyclized tetrazolo form (in water), especially within the larger compounds **26** and **27**. Overall, this data supports for the hypothesis that heteroaryl azides with proximal nitrogen can preferentially cyclize into tetrazolos.

**Fig. 5:**
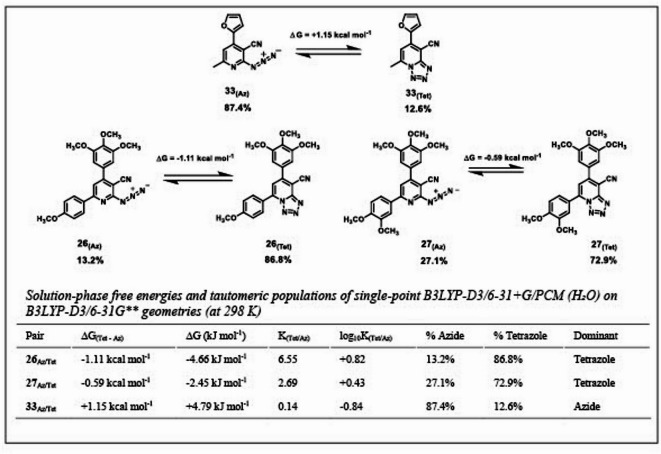
Solution-phase Gibbs free energies (ΔG), equilibrium constants, and tautomeric populations for the azido-tetrazole equilibrium of **26**,** 27**,** and 33** in water. Relative stabilities of the azido-tetrazolo forms were evaluated using DFT via B3LYP-D3/6-31G** with single-point energy calculations in water (PCM solvation model) on geometries optimized at B3LYP-D3/6–31 + G**. ΔG values are reported relative to the tetrazole; negative values indicate the tetrazole is favored. Equilibrium populations estimated using a Boltzmann distribution at 298 K.

#### Glide docking against Topo II, Topo II-DNA, and α/β-tubulin

**Fig. 6:**
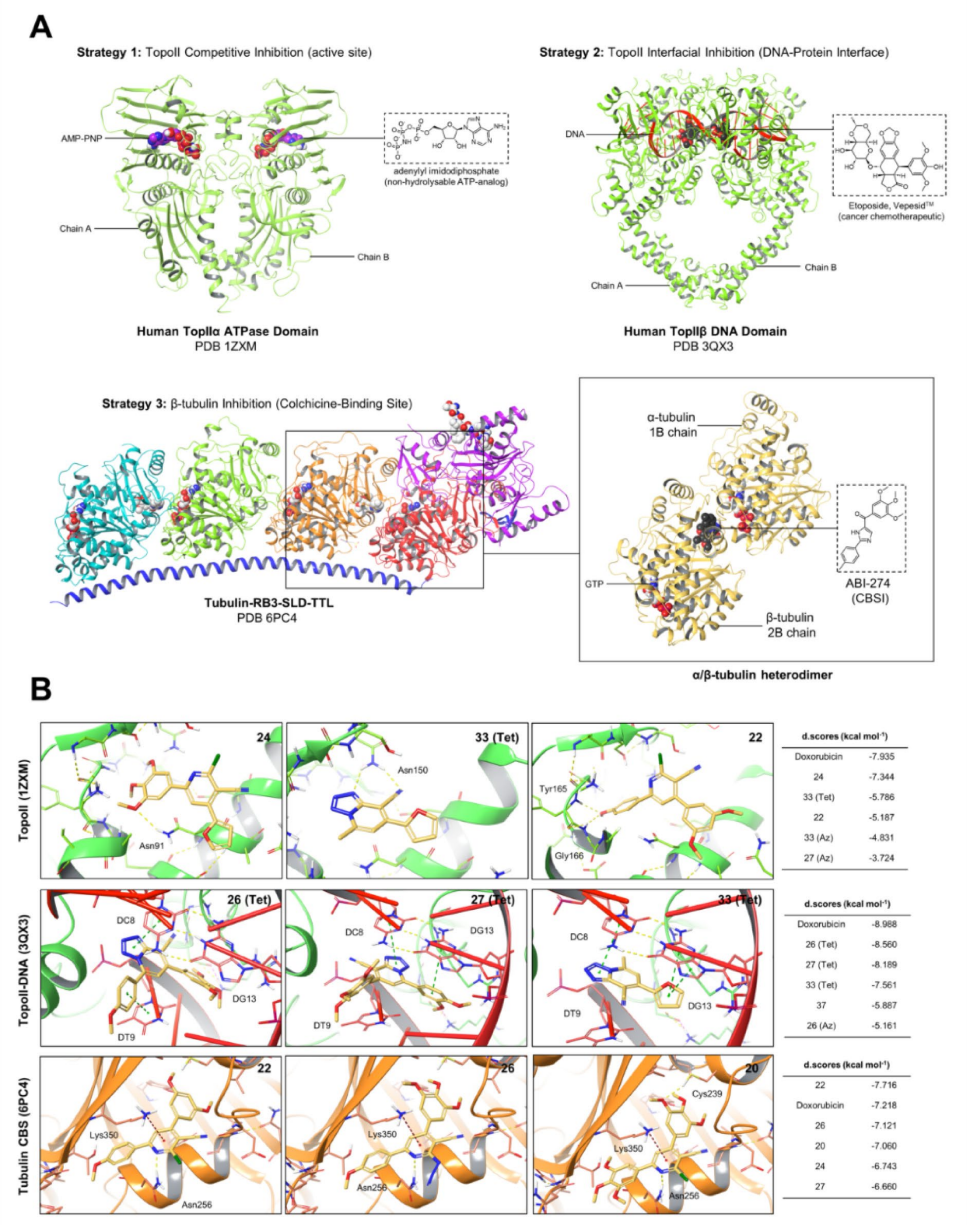
(**A**) Summary of the three docking strategies employed in our study: Strategy 1, competitive inhibition at the Topo IIα ATPase catalytic site (PDB 1ZXM), using AMP–PNP as the cognate ligand; Strategy 2, interfacial inhibition at the Topo IIβ DNA-protein interface (PDB 3QX3), with the clinical drug etoposide as a control; and Strategy 3, β-tubulin inhibition at the colchicine-binding site of the α/β-tubulin heterodimer (PDB 6PC4), using ABI-274 as a CBSI reference. (**B**) Protein binding sites are shown in cartoon representation with co-crystallized ligands highlighted and docking scores (in kcal mol^*−*1^) of the top 5 ligands are shown in the inset tables.

To gain insights into the molecular recognition and intermolecular forces that may drive the biochemical activity of our ligands, a series of docking experiments were run against our targets of interest, topoisomerase II and tubulin protein. Given the inherently complex pharmacology of Topo II (due to the presence of multiple binding sites), we elected to use two docking strategies for this target. First, we focused on the catalytic active site, using a human Topo II ATPase domain crystal structure co-crystallized with AMP-PNP (1ZXM, *r* = 1.87 Å), where we probed the orthosteric site. In parallel, we also selected a Topo II-DNA crystal complex bound to anti-cancer chemotherapeutic etoposide (3QX3, *r* = 2.16 Å) where we surveyed the DNA-protein interface as a potential binding site. In addition to Topo II, our ligands do inhibit tubulin polymerization, so we also evaluated protein-ligand docking against a tubulin-RB3-SLD-TTL complex co-crystallized with colchicine analog ABI-274 (6PC4, *r* = 2.60 Å), where we focused on the colchicine-binding site within the α/β-tubulin heterodimer. These calculations involved the following ligands: **20**,** 22**,** 24**,** 26**,** 27**,** 33**,** 35**,** 37**,** 38**,** 41** and corresponding **26** (tetrazolo), **27** (tetrazolo), **33** (tetrazolo) tautomers. DOX was also evaluated against all 3 targets as positive control. All protein targets first underwent standard protein preparation workflow, including the filling in of missing residues, assignment of bond orders, and the optimization of hydrogen-bond networks. Ligand structures were also prepared using a LigPrep protocol, ensuring correct three-dimensional structures, ionization states (Epik, pH 7.00 ± 2.00), and low energy minimizations at physiological pH. Receptor grids were generated for each protein target (10 × 10 × 10 Å) centered around the cognate co-crystallized ligands, before a glide docking protocol was performed (Glide-XP) enabling flexible sampling of ligand conformations within the prepared grids to deduce relevant binding poses and quantitative docking scores (in kcal mol^−1^). This calculation was performed against Topo II, Topo II-DNA, and tubulin proteins, with the most relevant results summarized in Fig. 6. In the catalytic site within the Topo II ATPase domain (1ZXM), several ligands were found to exhibit favorable docking scores. This includes positive control DOX (-7.935 kcal mol^−1^), as well as the chloropyridyl aryl analog **24** (-7.344 kcal mol^−1^), furanyl **33** (tet) (-5.786 kcal mol^−1^), and **22** (-5.187 kcal mol^−1^). In the active site, **24** was found to form several favorable hydrophobic contacts as well as a hydrogen bond between its aryl methoxy motif and nearby Asn91. The structurally simple **33** (in the tetrazolo form) was found to form a stable hydrogen bond network between a tetrazolo-nitrogen and its nearby cyano group with Asn150 in the Topo II pocket. Similar to **24**, compound **22** also occupied the Topo II active site in a similar pose, exhibiting two hydrogen bonds between the methoxy group and the peptide backbone of Tyr165 and Gly166. Binding poses within the Topo II-DNA interface (3QX3) provided important insight into the benefits of the tetrazolo tautomer (compared to the azido form). In this docking experiment, the ligands with the most favorable free energies of binding include control DOX (-8.988 kcal mol^−1^), **26** (tetrazolo, -8.560 kcal mol^−1^), **27** (tetrazolo, -8.189 kcal mol^*−*1^), and **33** (tetrazolo, -7.561 kcal mol^*−*1^). Interestingly, the highest affinity ligands were all in the tetrazolo form, and the ligands returned improved values in the Topo II-DNA interface compared to the Topo II ATPase active site. As seen in Fig. 6, this is primarily driven by the formation of favorable π-π stacking events between the aromatic tetrazolo and methoxybenzene motifs and neighboring DNA nucleotides (e.g., DC8, DT9, DG13). π-π stacking is a well-established non-covalent intermolecular force, typically arising from quadrupole-quadrupole or dispersion forces. Depending on its geometry (e.g., face-to-face, edge-to-face, or offset-stacking), a standard π-π stacking event can contribute between ~ 1–3 kcal mol^−1^ of energy to the observed binding affinity, often stabilizing protein-ligand interactions through aromatic ring complementarity. Compound **26** (tetrazolo) optimally occupies the Topo II-DNA interface and engaged the dsDNA strand *via* two π-π stacking events between the methoxy benzene and DT9 (thymine) as well as the tetrazolo ring and DC8 (cytosine). **27** (tetrazolo) was able to form similar interactions in the Topo II-DNA interfacial pocket, with π-π stacking of its secondary methoxy benzene ring and DG13 (guanine), as well as a similar stacking event between its tetrazolo motif and DC8 (cytosine). Finally, while the smaller **33** (tetrazolo) lost hydrophobic contacts, it compensated them *via* three π-π stacking events, two between its furan core and the adenine system of DG13 (guanine), as well as its tetrazolo and DC8 (cytosine). These interactions were unique to the tetrazolo tautomers of compounds **26**, **27**, and **33**. In the colchicine-binding site within the α/β-tubulin heterodimer (6PC4), the top ligands include **22** (-7.716 kcal mol^*−*1^), DOX (-7.218 kcal mol^*−*1^), **26** (-7.121 kcal mol^*−*1^), and **20** (-7.060 kcal mol^*−*1^). First, **22** occupied the colchicine-binding site in a canonical manner, with both methoxy benzene motifs on either end, and uniquely was found to exhibit a powerful π-cation stacking interaction between its central chloro-pyridine ring and a nearby positively charged Lys350. Unlike the standard π-π stacking event, a π–cation interaction involves the electrostatic attraction between an aromatic π-system and a nearby positively charged residue (commonly Lys or Arg) and is generally stronger, contributing ~ 5–10 kcal·mol⁻¹ as a function of distance and orientation. In addition, the pyridyl nitrogen was also found to have a hydrogen bond to nearby Asn256. Remarkably, compound **26** retained the exact same molecular recognition profile, with the π–cation interaction with Lys350 and hydrogen bond to Asn256. Furthermore, **20** also maintained the same interactions with Lys350 and Asn256. This conservation of intermolecular forces strengthens our confidence in these predicted binding poses, given consistent interactions across three separate docking experiments. Overall, our docking analyses demonstrate that the azido/tetrazolo scaffolds favorably engage Topo II and tubulin targets through a combination of hydrogen bonding, hydrophobic contacts, and aromatic π-π stacking or π-cation interactions. The tetrazolo tautomers displayed enhanced recognition at the Topo II-DNA interface, where multiple π–π stacking events with nearby nucleotides improved binding affinities relative to the ATPase catalytic site. In tubulin, the identification of a robust π–cation interaction with Lys350, conserved across several ligands, provided further confidence in the reliability of these predicted binding poses. Compounds **20**, and **26** emerged among the most favorable binders across all three protein systems, supporting a possible binding mechanism to explain the observed biochemical activities against Topo II and tubulin. Taken together, these results support the biological potential of this chemotype and highlight the value of tautomeric control and substituent tuning in optimizing noncovalent target engagement against clinically relevant proteins.

Overall, the 2,4,6-trisubstituted nicotinonitriles, especially compounds **20**, **26**, and **37**, showed potent and selective antiproliferative activity, mainly through tubulin polymerization inhibition with additional Topo II inhibition for some derivatives. SAR trends, cell cycle arrest at G2/M, apoptosis induction, and docking studies together support a dual mechanism involving microtubule disruption and Topo II–DNA interference. These results validate this nicotinonitrile scaffold as a promising basis for further development of multitarget anticancer agents.

## Conclusions

A series of 2,4,6-trisubstituted nicotinonitriles **10–41** as dual inhibitors of tubulin polymerization and Topo II was synthesized in this study. Compounds **20**,** 26**,** and 41** showed the highest in vitro cytotoxic activity against MCF7 cell line, while compounds **20**,** 26** showed moderate activity against HepG2 cell line maintaining high selectivity toward normal cells. In enzyme-based assays, compounds **26**,** 20**, and **37** exhibited significant tubulin polymerization inhibitory activity, respectively. Compound **37** showed strong inhibitory activity against Topo II, while compound **20** showed moderate Topo II inhibitory activity. Compounds **20** and **37** exhibited the most pronounced dual-target activity, effectively disrupting tubulin polymerization and inhibiting Topo II. Studies confirmed their ability to induce G2/M phase arrest and trigger apoptosis in MCF7 cells. Molecular docking analyses support the favorable binding interactions of these compounds with both targets, in line with the biological results. Collectively, these findings establish 2,4,6-trisubstituted nicotinonitriles as a promising structural scaffold for the future development of potent, selective, and dual-targeted anticancer therapeutics with improved clinical prospects.

## Experimental section

### Chemistry

All chemical reagents were purchased from commercial sources with a high percentage of purity. The melting points (°C) of the synthesized compounds were determined in open capillaries using Stuart melting point apparatus and were uncorrected. NMR, IR spectra and UV-Vis analyses were carried out at the Applied Nucleic Acid Research Center, Faculty of Sciences, Zagazig University, Zagazig, Egypt. The mass spectra analyses, and elemental analyses (C, H, N) were carried out at the Regional Center for Mycology and Biotechnology, Al-Azhar University, Nasr City, Egypt. The^1^ H-NMR and^13^ C APT NMR spectra were recorded on a Bruker Avance III 400 MHz High Performance Digital FT-NMR spectrometer using dimethyl sulfoxide (DMSO-*d*_6_) or deuterated chloroform (CDCl_3_) as solvent. Chemical shifts are reported in δ (ppm). The IR spectra (ATR, ν_max_/cm^−1^) of the compounds were recorded on a Bruker Alpha FT-IR spectrometer. The UV-Visible analyses were carried out by UV-Visible Carry 90 instrument. Mass spectra were obtained using a GC/MS Mat 112 S mass spectrometer under the EI^+^ ionization technique/mode. Elemental analyses were performed using a Vario MICRO cube (Elementar) CHNS analyzer. All reactions were monitored by thin layer chromatography on silica gel 60 GF245 (E-Merck, Germany) using UV lamp for visualization at a wavelength (λ) of 254 nm. Compounds **10**,** 11**,** 12**,** 15**,** 18** were previously reported^20,23–28^.

#### General method for synthesis of 4,6-disubstituted-2-oxo-nicotinonitrile derivatives (**10–17**)

A mixture of selected ketone **4**,** 5**,** 6**,** 8**, or **9** (10 mmol), selected aldehyde **1**,** 2**,** 3**,** 7** (10 mmol), ethyl cyanoacetate (15 mmol, 1.59 ml) and ammonium acetate (80 mmol, 6.1 g) in absolute ethanol (30 ml) was heated to reflux for 12–14 h. The reaction was monitored by TLC till it finished, then the reaction mixture was left to reach room temperature. The formed precipitate was filtered, washed successively with ethanol and dried under vacuum to afford pure compounds **10–17**.

6-(4-Methoxyphenyl)-2-oxo-4-(3,4,5-trimethoxyphenyl)-1,2-dihydropyridine-3-carbonitrile (**10**)^23–25^. Yellow solid; yield: (78%); mp: 270–272 °C (lit.^23^ <300 °C); ^1^H NMR (400 MHz, DMSO-*d*_*6*_) δ ppm: 12.63 (s, 1H, NH), 7.89 (d, *J* = 8.9 Hz, 2 H, Ar-H), 7.08 (d, *J* = 9.0 Hz, 2 H, Ar-H), 7.04 (s, 2 H, Ar-H), 6.84 (s, 1H, C_5_-H of pyridone), 3.85 (s, 6 H, 2OCH_3_), 3.83 (s, 3 H, OCH_3_), 3.73 (s, 3 H, OCH_3_); ^13^C APT NMR (100 MHz, DMSO-*d*_*6*_) δ ppm: 162.20, 161.72, 159.54, 152.85, 150.94, 139.11, 131.36, 129.49, 124.37, 116.98, 114.36, 106.07, 105.16, 97.02, 60.14, 56.16, 55.53; IR (ATR, ν_max_/cm^*−*1^): 3446 (NH), 3081 (CH-Ar), 2830 (CH-alipha), 2216 (CN), 1651 (C = O); UV/Vis: λ_max_ 375, 260 nm.

6-(3,4-Dimethoxyphenyl)-2-oxo-4-(3,4,5-trimethoxyphenyl)-1,2-dihydropyridine-3-carbonitrile (**11**)^20^. Yellow solid; yield (77%); mp: 275–277 °C (lit^20^. 250–252 °C); ^1^H NMR (400 MHz, DMSO-*d*_6_) δ ppm: 12.63 (s, 1H, NH), 7.54 (d, *J* = 8 Hz, 1H, Ar-H), 7.48 (s, 1H, Ar-H ), 7.10 (d, *J* = 8 Hz, 1H, Ar-H), 7.03 (s, 2 H, Ar-H), 6.89 (s, 1H, C_5_-H of pyridone), 3.86 (s, 3 H, OCH_3_), 3.86 (s, 6 H, 2OCH_3_), 3.83 (s, 3 H, OCH_3_), 3.74 (s, 3 H, OCH_3_); ^13^C APT NMR (100 MHz, DMSO-*d*_6_) δ ppm: 162.12, 159.71, 152.89, 151.47, 148.79, 139.14, 131.43, 124.22, 121.17, 117.01, 111.70, 110.83, 106.10, 60.18, 56.20, 55.76; IR (ATR, ν_max_/cm^*−*1^): 3446 (NH), 3013 (CH-Ar), 2831 (CH-alipha), 2215 (CN), 1651 (C = O); UV/Vis: λ_max_ 380 nm.

2-Oxo-4,6-bis(3,4,5-trimethoxyphenyl)-1,2-dihydropyridine-3-carbonitrile **(12)**^23^. Yellow solid; yield: (75%); mp: 280–282 °C (lit^23^. 286–288 °C); ^1^H NMR (400 MHz, DMSO-*d*_6_) δ ppm: 12.68 (s, 1H, NH), 7.19 (s, 2 H, Ar-H), 7.04 (s, 2 H, Ar-H), 6.97 (s, 1H, C_5_-H of pyridone), 3.88 (s, 12 H, 4OCH_3_), 3.74 (s, 6 H, 2OCH_3_); ^13^C APT NMR (100 MHz, DMSO-*d*_6_) δ ppm: 161.94, 159.80, 153.04, 152.86, 139.96, 139.14, 131.29, 127.22, 116.80, 106.13, 105.40, 60.14, 60.13, 56.21, 56.19; IR (ATR, ν_max_/cm^*−*1^): 3502 (NH), 3015 (CH-Ar), 2837 (CH-alipha), 2221 (CN), 1646 (C = O); UV/Vis: λ_max_ 370 nm.

6-(3,4-Dimethoxyphenyl)-4-(3,5-dimethoxyphenyl)-2-oxo-1,2-dihydropyridine-3-carbonitrile (**13**). Yellow solid; yield: (78%); mp: 270–272 °C; ^1^H NMR (400 MHz, DMSO-*d*_6_) δ ppm: 12.60 (s, 1H, NH), 7.49 (d, *J* = 8.5 Hz, 1H, Ar-H), 7.42 (s, 1H, Ar-H), 7.03 (d, *J* = 8.6 Hz, 1H, Ar-H), 6.78 (s, 2 H, Ar-H), 6.78 (s, 1H, Ar-H), 6.62 (s, 1H, C_5_-H of pyridone), 3.80 (s, 3 H, OCH_3_), 3.77 (s, 3 H, OCH_3_), 3.76 (s, 6 H, 2OCH_3_); ^13^C APT NMR (100 MHz, DMSO-*d*_6_) δ ppm: 162.01, 160.48, 159.66, 151.46, 148.76, 138.10, 124.15, 121.14, 116.61, 111.65, 110.79, 106.31, 101.86, 55.72, 55.49; IR (ATR, ν_max_/ cm^*−*1^): 3502 (NH), 3065 (CH-Ar), 2834 (CH-alipha), 2222 (CN), 1645 (C = O); UV/Vis: λ_max_ 380 nm; MS, *m/z*: 392.53 (M^+^); analysis (calcd., found for C_22_H_20_N_2_O_5_): C (67.34, 67.48), H (5.14, 5.23), N (7.14, 7.41).

4-(3,5-Dimethoxyphenyl)-6-(4-methoxyphenyl)-2-oxo-1,2-dihydropyridine-3-carbonitrile (**14**). Yellow solid; yield: (76%); mp: 260–262 °C; ^1^H NMR (400 MHz, DMSO-*d*_6_) δ ppm: 12.68 (s, 1H, NH), 7.90 (d, *J* = 7.8 Hz, 2 H, Ar-H), 7.08 (d, *J* = 8.8 Hz, 2 H, Ar-H), 6.85 (d, *J* = 2.1 Hz, 2 H, Ar-H), 6.77 (s, 1H, Ar-H), 6.68 (s, 1H, C_5_-H of pyridone), 3.84 (s, 3 H, OCH_3_), 3.82 (s, 6 H, 2OCH_3_); ^13^C APT-NMR (100 MHz, DMSO-*d*_*6*_) δ ppm: 161.77, 160.50, 138.07, 129.52, 116.59, 114.39, 106.29, 102.00, 55.54, 55.52; IR (ATR, ν_max_/cm^*−*1^): 3450 (NH), 3050 (CH-Ar), 2837 (CH-alipha), 2211 (CN), 1646 (C = O); UV/Vis: λ_max_ 375, 265 nm; MS, *m/z*: 362.67 (M^+^); analysis (calcd., found for C_21_H_18_N_2_O_4_): C (69.60, 69.43), H (5.01, 5.20), N (7.73, 7.96).

6-(3,4-Dimethoxyphenyl)-4-(4-methoxyphenyl)-2-oxo-1,2-dihydropyridine-3-carbonitrile (**15**)^26–28^. Yellow solid; yield: (77%); mp: 255–257 °C (lit^26^. 262–264 °C); ^1^H NMR (400 MHz, DMSO-*d*_6_) δ ppm: 12.57 (s, 1H, NH), 7.72 (d, *J* = 8.8 Hz, 2 H, Ar-H), 7.53 (d, *J* = 8.5 Hz, 1H, Ar-H), 7.47 (s, 1H, Ar-H), 7.12 (d, *J* = 8.8 Hz, 2 H, Ar-H), 7.08 (d, *J* = 8.6 Hz, 1H, Ar-H), 6.80 (s, 1H, C_5_-H of pyridone), 3.86 (s, 3 H, OCH_3_), 3.85 (s, 3 H, OCH_3_), 3.83 (s, 3 H, OCH_3_); ^13^C APT NMR (100 MHz, DMSO-*d*_6_) δ ppm: 162.21, 161.06, 159.35, 151.40, 148.77, 130.02, 128.26, 124.24, 121.04, 117.08, 114.20, 111.69, 110.76, 55.74, 55.46; IR (ATR, ν_max_/cm^*−*1^): 3490 (NH), 3065 (CH-Ar), 2904 (CH-alipha), 2222 (CN), 1651 (C = O); UV/Vis: λ_max_ 380 nm.

4-(Furan-2-yl)-2-oxo-6-(3,4,5-trimethoxyphenyl)-1,2-dihydropyridine-3-carbonitrile (**16**). Yellow solid; yield: (78%) ; mp: 254–256 °C; ^1^H NMR (400 MHz, DMSO-*d*_6_) δ ppm: 12.56 (s, 1H, NH), 8.14 (d, *J* = 1.3 Hz, 1H, furyl-H), 7.72 (d, *J* = 3.5 Hz, 1H, furyl-H), 7.18 (s, 2 H, Ar-H), 7.08 (s, 1H, C_5_-H of pyridone), 6.87 (dd, *J* = 3.6, 1.7 Hz, 1H, furyl-H), 3.91 (s, 6 H, 2OCH_3_), 3.74 (s, 3 H, OCH_3_); ^13^C APT NMR (100 MHz, DMSO-*d*_6_) δ ppm: 153.02, 139.83, 116.79, 116.56, 113.34, 105.23, 60.14, 56.20; IR (ATR, ν_max_/cm^*−*1^): 3446 (NH), 3031 (CH-Ar), 2838 (CH-alipha), 2217(CN), 1645 (C = O); UV/Vis: λ_max_ 385, 340 nm; MS, *m/z*: 352.17 (M^+^); analysis (calcd., found for C_19_H_16_N_2_O_5_): C (64.77, 64.58), H (4.58, 4.72), N (7.95, 8.17).

4-(Furan-2-yl)-6-methyl-2-oxo-1,2-dihydropyridine-3-carbonitrile (**17**). Yellow solid; yield: (77%); mp: 221–223 °C; ^1^H NMR (400 MHz, DMSO-*d*_6_) δ ppm: 12.36 (s, 1H, NH), 8.05 (d, *J* = 1.2 Hz, 1H, C_5_-H of furan), 7.54 (d, J = 3.6 Hz, 1H, C_3_-H of furan), 6.80 (dd, *J* = 3.6, 1.8 Hz, 1H, C_4_-H of furan), 6.59 (s, 1H, C_5_-H of pyridone), 2.28 (s, 3 H, CH_3_); ^13^C APT NMR (100 MHz, DMSO-*d*_*6*_) δ ppm: 161.66, 151.93, 147.60, 146.94, 145.30, 116.92, 115.85, 113.31, 101.33, 91.30, 19.20.

#### General method for synthesis of 4,6-disubstituted-2-chloro-nicotinonitrile derivatives (**18–25**)

A mixture of selected pyridone derivative **10–17** (5 mmol) and POCl_3_ (25 mmol, 2.3 ml) in presence of *N*,* N-*dimethylaniline (15 mmol, 1.9 ml) was heated to reflux for 12–16 h. The reaction mixture was monitored by TLC till it finished. It was cooled to room temperature and poured into crushed ice with stirring till precipitation. The formed precipitate was filtered off, washed successively with water, crystallized from ethanol and dried under vacuum to obtain pure compounds **18–25**.

2-Chloro-6-(4-methoxyphenyl)-4-(3,4,5-trimethoxyphenyl)nicotinonitrile (**18**)^25^. Buff solid; yield: (81%); mp: 188–190 °C; ^1^H NMR (400 MHz, CDCl_3_) δ ppm: 8.06 (d, *J* = 8.8 Hz, 2 H, Ar-H), 7.69 (s, 1H, C_5_-H of pyridine), 7.02 (d, *J* = 8.8 Hz, 2 H, Ar-H), 6.83 (s, 2 H, Ar-H), 3.95 (s, 6 H, 2OCH_3_), 3.93 (s, 3 H, OCH_3_), 3.89 (s, 3 H, OCH_3_); ^13^C APT NMR (100 MHz, DMSO-*d*_6_) δ ppm: 161.93, 158.63, 155.95, 152.97, 152.32, 139.13, 130.42, 129.50, 127.84, 118.40, 115.70, 114.48, 106.63, 105.20, 60.14, 56.21, 55.46; IR (ATR, ν_max_/cm^*−*1^): 3001 (CH-Ar), 2831 (CH-alipha), 2224 (CN); UV/Vis: λ_max_ 340 nm; MS, *m/z*: 410.34 (M^+^), 412.07(M^+ 2^); analysis (calcd., found for C_22_H_19_ClN_2_O_4_): C (64.32, 64.50), H (4.66, 4.75), N (6.82, 7.09).

2-Chloro-6-(3,4-dimethoxyphenyl)-4-(3,4,5trimethoxyphenyl)nicotinonitrile (**19**). Green solid; yield: (85%); mp: 212–214 °C; ^1^H NMR (400 MHz, DMSO-*d*_6_) δ ppm: 8.22 (s, 1H, C_5_-H of pyridine), 7.88 (d, *J* = 7.4 Hz, 1H, Ar-H), 7.75 (s, 1H, Ar-H), 7.16 (s, 1H, Ar-H), 7.11 (s, 2 H, Ar-H), 3.87 (s, 9 H, 3OCH_3_), 3.76 (s, 6 H, 2OCH_3_); ^13^C APT NMR (100 MHz, DMSO-*d*_6_) δ ppm: 159.22, 156.51, 153.45, 152.64, 152.24, 149.54, 139.57, 130.99, 128.42, 121.89, 119.16, 116.17, 112.24, 111.00, 107.14, 105.81, 60.63, 56.72, 56.24, 56.17; IR (ATR, ν_max_/cm^*−*1^): 3010 (CH-Ar), 2940 (CH-alipha), 2220 (CN); UV/Vis: λ_max_ 350 nm; MS, *m/z*: 440.22 (M^+^), 442.34 (M^+ 2^); analysis (calcd., found for C_23_H_21_ClN_2_O_5_): C (62.66, 62.89), H (4.80, 4.72), N (6.35, 6.47).

2-Chloro-4,6-bis(3,4,5-trimethoxyphenyl)nicotinonitrile (**20**). Green solid; yield: (87%); mp: 244–246 °C; ^1^H NMR (400 MHz, DMSO-*d*_6_) δ ppm: 8.32 (s, 1H, C_5_-H of pyridine), 7.51 (s, 2 H, Ar-H), 7.12 (s, 2 H, Ar-H), 3.88 (d, *J* = 7.5 Hz, 12 H, 4OCH_3_), 3.75 (d, *J* = 3.0 Hz, 6 H, 2OCH_3_); ^13^C APT NMR (100 MHz, DMSO-*d*_6_) δ ppm: 158.70, 156.37, 153.39, 153.09, 152.17, 140.48, 139.22, 131.00, 130.54, 119.60, 115.66, 106.80, 106.23, 105.34, 60.32, 60.27, 56.35, 56.32; IR (ATR, ν_max_/cm^*−*1^): 3001 (CH-Ar), 2831 (CH-alipha), 2224 (CN); UV/Vis: λ_max_ 345 nm; MS, *m/z*: 470.84 (M^+^), 472.28 (M^+ 2^); analysis (calcd., found for C_24_H_23_ClN_2_O_6_): C (61.21, 61.43), H (4.92, 5.06), N (5.95, 6.12).

2-Chloro-6-(3,4-dimethoxyphenyl)-4-(3,5-dimethoxyphenyl)nicotinonitrile (**21**). Buff solid; yield: (86%); mp: 194–196 °C; ^1^H NMR (400 MHz, DMSO-*d*_6_) δ ppm: 8.21 (s, 1H, C_5_-H of pyridine), 7.89 (dd, *J* = 8.5, 1.9 Hz, 1H, Ar-H), 7.76 (d, *J* = 1.8 Hz, 1H, Ar-H), 7.12 (d, *J* = 8.6 Hz, 1H, Ar-H), 6.91 (d, *J* = 2.1 Hz, 2 H, Ar-H), 6.72 (s, 1H, Ar-H), 3.87 (s, 3 H, OCH_3_), 3.85 (s, 3 H, OCH_3_), 3.83 (s, 6 H, 2OCH_3_); ^13^C APT NMR (100 MHz, DMSO-*d*_6_) δ ppm: 161.05, 159.36, 156.50, 152.59, 152.27, 149.55, 137.65, 128.38, 121.92, 119.14, 115.89, 112.26, 111.00, 107.45, 105.94, 102.36, 56.26, 56.17, 56.05; IR (ATR, ν_max_/cm^*−*1^): 3011 (CH-Ar), 2841 (CH-alipha), 2233 (CN); UV/Vis: λ_max_ 350, 285 nm; MS, *m/z*: 410.21 (M^+^), 412.38 (M^+ 2^); analysis (calcd., found for C_22_H_19_ClN_2_O_4_): C (64.32, 64.57), H (4.66, 4.81), N (6.82, 7.04).

2-Chloro-4-(3,5-dimethoxyphenyl)-6-(4-methoxyphenyl)nicotinonitrile (**22**). Buff solid; yield: (84%); mp: 221–223 °C; ^1^H NMR (400 MHz, DMSO-*d*_6_) δ ppm: 8.22 (d, *J* = 8.9 Hz, 2 H, Ar-H), 8.14 (s, 1H, C_5−_H of pyridine), 7.09 (d, *J* = 8.9 Hz, 2 H, Ar-H), 6.92 (d, *J* = 2.1 Hz, 2 H, Ar-H), 6.71 (t, *J* = 2.1 Hz, 1H, Ar-H), 3.85 (s, 3 H, OCH_3_), 3.83 (s, 6 H, 2OCH_3_); ^13^C APT NMR (100 MHz, DMSO-*d*_6_) δ ppm: 162.44, 161.05, 159.25, 156.41, 152.75, 137.56, 130.00, 128.27, 118.89, 115.89, 114.98, 107.38, 105.81, 102.48, 56.03, 55.95; IR (ATR, ν_max_/cm^*−*1^): 3016 (CH-Ar), 2841 (CH-alipha), 2224 (CN); UV/Vis: λ_max_ 335 nm; MS, *m/z*: 380.77 (M^+^), 382.62 (M^+ 2^); analysis (calcd., found for C_21_H_17_ClN_2_O_3_): C (66.23, 66.45), H (4.50, 4.61), N (7.36, 7.63).

2-Chloro-6-(3,4-dimethoxyphenyl)-4-(4-methoxyphenyl)nicotinonitrile (**23**). Brown solid; yield: (88%); mp: 217–219 °C; ^1^H NMR (400 MHz, DMSO-*d*_6_) δ ppm: 8.16 (s, 1H, C_5_-H of pyridine), 7.88 (s, 1H, Ar-H), 7.76 (s, 2 H, Ar-H), 7.17 (s, 2 H, Ar-H), 6.71 (s, 1H, Ar-H), 6.63 (s, 1H, Ar-H), 3.86 (s, 9 H, 3OCH_3_); ^13^C APT NMR (100 MHz, DMSO-*d*_6_) δ ppm:160.97, 158.63, 155.71, 152.26, 151.62, 148.97, 130.44, 128.77, 127.93, 127.32, 121.26, 118.32, 116.02, 115.70, 114.27, 112.31, 111.70, 110.38, 104.81, 55.70, 55.62, 55.42; IR (ATR, ν_max_/cm^*−*1^): 3079 (CH-Ar), 2838 (CH-alipha), 2223 (CN); UV/Vis: λ_max_ 345 nm; MS, *m/z*: 380.25 (M^+^), 382.05 (M^+ 2^); analysis (calcd., found for C_21_H_17_ClN_2_O_3_): C (66.23, 66.50), H (4.50, 4.62), N (7.36, 7.63).

2-Chloro-4-(furan-2-yl)-6-(3,4,5-trimethoxyphenyl)nicotinonitrile (**24**). Green solid; yield: (89%) ; mp: 210–212 °C; ^1^H NMR (400 MHz, DMSO-*d*_6_) δ ppm: 8.37 (s, 1H, C_5_-H of pyridine), 8.15 (s, 1H, furyl-H), 7.77 (s, 1H, furyl-H), 7.46 (s, 2 H, Ar-H), 6.89 (s, 1H, furyl-H), 3.90 (d, *J* = 5.7 Hz, 6 H, 2OCH3), 3.75 (s, 3 H, OCH3); ^13^C APT NMR (100 MHz, DMSO-*d*_6_) δ ppm: 158.69, 153.35, 153.25, 152.66, 147.25, 142.68, 131.65, 130.85, 115.89, 115.63, 114.47, 114.30, 113.45, 105.11, 104.88, 100.49, 60.27, 56.44, 56.28; IR (ATR, ν_max_/cm^*−*1^): 3050 (CH-Ar), 2835 (CH-alipha), 2227 (CN); UV/Vis: λ_max_ 360, 300, 255 nm; MS, *m/z*: 370.31 (M^+^), 372.27(M^+ 2^); analysis (calcd., found for C_19_H_15_ClN_2_O_4_): C (61.55, 61.79), H (4.08, 4.21), N (7.56, 7.80).

2-Chloro-4-(furan-2-yl)-6-methylnicotinonitrile (**25**). Yellow solid; yield: (77%); mp: 226–228 °C; ^1^H NMR (400 MHz, CDCl_3_) δ ppm: 7.65 (s, 2 H, C_5_-H of furan and C_5_-H of pyridine), 7.59 (s, 1H, C_3_-H of furan), 6.64 (d, *J* = 1.6 Hz, 1H, C_4_-H of furan), 2.63 (s, 3 H, CH_3_); ^13^C APT NMR (100 MHz, CDCl_3_) δ ppm: 163.05, 153.97, 147.23, 145.67, 142.49, 117.10, 115.68, 115.56, 113.31, 100.71, 25.01.

#### General procedure for synthesis of 4,6-disubstituted-2-azido/tetrazolo-nicotinonitrile derivatives (**26–33**)

To a solution of selected chloro pyridine derivatives **18–25** (3 mmol) in DMF, a solution of sodium azido (12 mmol, 0.7 gm) in least amount of water was dropped into, then the reaction mixture was allowed to be stirred for 12 h at 80 °C. The reaction mixture was poured into water till precipitation then filtered off, washed successively with water then hexane and dried under vacuum to get compounds **26–33**.

5-(4-methoxyphenyl)-7-(3,4,5-trimethoxyphenyl)tetrazolo[1,5-a]pyridine-8-carbonitrile (**26**). Green solid; yield: (86%); mp: 184–186 °C; ^1^H NMR (400 MHz, DMSO-*d*_6_) δ ppm: 8.23 (s, 2 H, Ar-H), 7.90 (s, 1H, C_5_-H of pyridine), 7.24 (s, 2 H, Ar-H), 7.10 (d, *J* = 14.2 Hz, 2 H, Ar-H), 3.90 (s, 9 H, 3OCH_3_), 3.79 (s, 3 H, OCH_3_); ^13^C APT NMR (100 MHz, DMSO-*d*_6_) δ ppm: 162.40, 153.44, 152.43, 149.81, 141.65, 139.92, 132.07, 130.23, 121.81, 116.94, 114.64, 107.22, 93.61, 60.62, 56.60, 55.95; IR (ATR, ν_max_/cm^*−*1^): 3010 (CH-Ar), 2841 (CH-alipha), 2227 (CN), 2126(N_3_); UV/Vis: λ_max_ 350, 295 nm; MS, *m/z*: 417.46 (M^+^); analysis (calcd., found for C_22_H_19_N_5_O_4_): C (63.30, 63.43), H (4.59, 4.78), N (16.78, 17.05).

5-(3,4-dimethoxyphenyl)-7-(3,4,5-trimethoxyphenyl)tetrazolo[1,5-a]pyridine-8-carbonitrile (**27**). Green solid; yield: (88%); mp: 165–167 °C; ^1^H NMR (400 MHz, DMSO-*d*_6_) δ ppm: 7.91 (s, 2 H, Ar-H), 7.78 (s, 1H, Ar-H), 7.24 (s, 2 H, Ar-H), 7.08 (s, 1H, C_5_-H of pyridine), 3.89 (s, 15 H, 5OCH_3_); ^13^C APT NMR (100 MHz, DMSO-*d*_6_) δ ppm: 153.62, 152.43, 149.96, 149.12, 141.87, 130.42, 124.17, 122.03, 117.34, 114.96, 113.59, 112.10, 107.74, 107.51, 93.93, 60.71, 56.80, 56.39, 56.34; IR (ATR, ν_max_/cm^*−*1^): 3022 (CH-Ar), 2841 (CH-alipha), 2225 (CN), 2134 (N_3_); UV/Vis: λ_max_ 360 nm; MS, *m/z*: 447.42 (M^+^); analysis (calcd., found for C_23_H_21_N_5_O_5_): C (61.74, 61.85), H (4.73, 4.90), N (15.65, 15.81).

5,7-bis(3,4,5-trimethoxyphenyl)tetrazolo[1,5-a]pyridine-8-carbonitrile (**28**). Yellow solid; yield: (85%); mp: 212–214 °C; ^1^H NMR (400 MHz, DMSO-*d*_6_) δ ppm: 8.03 (s, 1H, C_5_-H of pyridine), 7.52 (s, 2 H, Ar-H), 7.25 (s, 2 H, Ar-H), 3.90 (d, *J* = 5.2 Hz, 12 H, 4OCH_3_), 3.80 (d, *J* = 6.8 Hz, 6 H, 2OCH_3_); ^13^C APT NMR (100 MHz, DMSO-*d*_6_) δ ppm: 153.63, 153.38, 152.57, 149.84, 141.72, 140.96, 140.10, 130.30, 125.11, 118.16, 114.85, 108.24, 107.52, 94.62, 60.77, 60.71, 56.83, 56.79; IR (ATR, ν_max_/cm^*−*1^): 3050 (CH-Ar), 2838 (CH-alipha), 2226 (CN), 2135 (N_3_); UV/Vis: λ_max_ 350 nm; MS, *m/z*: 477.74 (M^+^); analysis (calcd., found for C_24_H_23_N_5_O_6_): C (60.37, 60.51), H (4.86, 4.92), N (14.67, 14.85).

5-(3,4-dimethoxyphenyl)-7-(3,5-dimethoxyphenyl)tetrazolo[1,5-a]pyridine-8-carbonitrile (**29**). Yellow solid; yield: (87%); mp: 178–180 °C; ^1^H NMR (400 MHz, DMSO-*d*_6_) δ ppm: 7.90 (s, 2 H, Ar-H), 7.77 (s, 1H, C_5_-H of pyridine), 7.24 (d, *J* = 7.5 Hz, 1H, Ar-H), 7.02 (s, 2 H, Ar-H), 6.77 (s, 1H, Ar-H), 3.90 (s, 3 H, OCH_3_), 3.86 (s, 9 H, 3OCH_3_); ^13^C APT NMR (100 MHz, DMSO-*d*_6_) δ ppm: 160.77, 152.00, 149.42, 148.67, 141.56, 136.62, 123.74, 121.48, 116.73, 114.17, 113.06, 111.58, 107.29, 102.30, 93.90, 55.92, 55.87, 55.66; IR (ATR, ν_max_/cm^*−*1^): 3005 (CH-Ar), 2838 (CH-alipha), 2227 (CN); UV/Vis: λ_max_ 360 nm; MS, *m/z*: 417.86 (M^+^); analysis (calcd., found for C_22_H_19_N_5_O_4_): C (63.30, 63.47), H (4.59, 4.73), N (16.78, 17.05).

7-(3,5-dimethoxyphenyl)-5-(4-methoxyphenyl)tetrazolo[1,5-a]pyridine-8-carbonitrile **(30).** Yellow solid; yield: (84%); mp: 180–182 °C; ^1^H NMR (400 MHz, DMSO-*d*_6_) δ ppm: 8.23 (d, *J* = 8.0 Hz, 2 H, Ar-H), 7.84 (s, 1H, C_5_-H of pyridine), 7.21 (d, *J* = 8.2 Hz, 2 H, Ar-H), 7.03 (s, 2 H, Ar-H), 6.76 (s, 1H, Ar-H), 3.90 (s, 3 H, OCH_3_), 3.85 (s, 6 H, 2OCH_3_); ^13^C APT NMR (100 MHz, DMSO-*d*_6_) δ ppm: 162.14, 160.75, 151.91, 149.41, 141.45, 136.53, 131.79, 121.46, 116.49, 114.31, 114.15, 107.22, 106.74, 102.37, 93.77, 55.64, 55.53; IR (ATR, ν_max_/cm^*−*1^): 3004 (CH-Ar), 2840 (CH-alipha), 2218 (CN), 2128 (N_3_); UV/Vis: λ_max_ 350, 275 nm: 350, 275; MS, *m/z*: 387.49 (M^+^); analysis (calcd., found for C_21_H_17_N_5_O_3_): C (65.11, 65.37), H (4.42, 4.59), N (18.08, 18.31).

5-(3,4-dimethoxyphenyl)-7-(4-methoxyphenyl)tetrazolo[1,5-a]pyridine-8-carbonitrile (**31**). Brown solid; yield: (86%); mp: 182–184 °C; ^1^H NMR (400 MHz, DMSO-*d*_6_) δ ppm: 7.92 (d, *J* = 6.3 Hz, 2 H, Ar-H), 7.87 (s, 1H, C_5_-H of pyridine), 7.78 (s, 1H, Ar-H), 7.74 (d, *J* = 4.6 Hz, 2 H, Ar-H), 7.24 (d, *J* = 4.8 Hz, 2 H, Ar-H), 3.91 (s, 3 H, OCH_3_), 3.90 (s, 6 H, 2OCH_3_); ^13^C APT NMR (100 MHz, DMSO-*d*_6_) δ ppm: 161.47, 151.83, 141.40, 130.98, 126.86, 123.63, 121.61, 116.72, 114.63, 113.03, 111.58, 92.59, 55.92, 55.84, 55.59; IR (ATR, ν_max_/cm^*−*1^): 3011 (CH-Ar), 2844 (CH-alipha), 2223 (CN), 2135 (N_3_); UV/Vis: λ_max_ 355, 255 nm; MS, *m/z*: 387.69(M^+^); analysis (calcd., found for C_21_H_17_N_5_O_3_): C (65.11, 64.98), H (4.42, 4.61), N (18.08, 18.27).

7-(furan-2-yl)-8-methyl-5-(3,4,5-trimethoxyphenyl)tetrazolo[1,5-a]pyridine (**32**). Yellow solid; yield: (88%); mp: 162–164 °C; ^1^H NMR (400 MHz, DMSO-*d*_6_) δ ppm: 8.25 (s, 1H, C_5_-H of pyridine), 8.02 (s, 1H, furyl-H), 7.93 (s, 1H, furyl-H), 7.44 (s, 2 H, Ar-H), 6.95 (s, 1H, furyl-H), 3.90 (s, 6 H, 2OCH_3_), 3.81 (s, 3 H, OCH_3_); ^13^C APT NMR (100 MHz, DMSO-*d*_6_) δ ppm: 152.86, 149.90, 147.63, 141.57, 140.41, 137.99, 124.58, 117.62, 114.14, 113.83, 113.30, 107.90, 107.62, 87.81, 60.29, 56.33; IR (ATR, ν_max_/cm^*−*1^): 3002 (CH-Ar), 2840 (CH-alipha), 2227 (CN), 2136 (N_3_); UV/Vis: λ_max_ 365, 300, 280 nm; MS, *m/z*: 377.51 (M^+^); analysis (calcd., found for C_19_H_15_N_5_O_4_): C (60.48, 60.73), H (4.01, 4.20), N (18.56, 18.83).

7-(furan-2-yl)-5-methyltetrazolo[1,5-a]pyridine-8-carbonitrile (**33**). Green solid; yield: (87%); mp: 228–230 °C; ^1^H NMR (400 MHz, DMSO-*d*_6_) δ ppm: 8.21 (s, 1H, C_5_-H of pyridine), 7.87 (s, 1H, furyl-H), 7.73 (s, 1H, furyl-H), 6.91 (s, 1H, furyl-H), 2.93 (s, 3 H, CH_3_); ^13^C APT NMR (100 MHz, DMSO-*d*_6_) δ ppm: 149.03, 147.96, 147.50, 141.50, 137.88, 116.95, 114.13, 113.86, 112.96, 87.29, 17.13; IR (ATR, ν_max_/cm^*−*1^): 3001 (CH-Ar), 2840 (CH-alipha), 2232 (CN); UV/Vis: λ_max_ 340, 275 nm; MS, *m/z*: 225.63 (M^+^); analysis (calcd., found for C_11_H_7_N_5_O): C (58.67, 58.90), H (3.13, 3.27), N (31.10, 30.98).

#### General procedure for synthesis of 4,6-disubstituted-2-iminophosphorane-nicotinonitrile derivatives (**34–41**)

A mixture of selected azido/tetrazolo compounds **26–33** (2 mmol) and triphenylphosphine (2.4 mmol, 629.49 mg) in toluene (15 ml) was heated to reflux for 15 min. The reaction mixture was followed by TLC until completion, then evaporated and the precipitate was washed with hexane to remove excess reagent. The pure fluorescent product was dried under vacuum to afford pure compounds **34–41**.

6-(4-Methoxyphenyl)-4-(3,4,5-trimethoxyphenyl)-2-((triphenyl-λ^5^-phosphaneylidene)amino)-nicotinonitrile (**34**). Buff solid; yield: (89%); mp: 211–213 °C; ^1^H NMR (400 MHz, DMSO-*d*_6_) δ ppm: 7.86 (s, 6 H, Ar-H ), 7.58 (s, 9 H, Ar-H), 7.43 (s, 1H, C_5_-H of pyridine), 7.16 (s, 2 H, Ar-H), 7.00 (s, 2 H, Ar-H), 6.80 (s, 2 H, Ar-H), 3.86 (s, 6 H, 2OCH_3_), 3.77 (s, 6 H, 2OCH_3_); ^13^C APT NMR (100 MHz, DMSO-*d*_*6*_) δ ppm: 164.89, 164.84, 160.65, 156.65, 154.81, 152.91, 138.31, 132.95, 132.64, 132.54, 131.65, 131.55, 130.48, 129.37, 129.08, 128.96, 128.86, 128.62, 128.37, 119.30, 113.82, 108.39, 106.10, 95.86, 95.62, 60.23, 56.16, 55.35; ^31^P NMR (162 MHz, DMSO-*d*_6_) δ ppm: 26.31 (s), 14.08 (s); IR (ATR, ν_max_/cm^*−*1^): 3010 (CH-Ar), 2837 (CH-alipha), 2206 (CN); UV/Vis: λ_max_ 355, 295 nm; MS, *m/z*: 652.32 (M^+^); analysis (calcd., found for C_40_H_34_N_3_O_4_P): C (73.72, 73.51), H (5.26, 5.43), N (6.45, 6.72).

6-(3,4-Dimethoxyphenyl)-4-(3,4,5-trimethoxyphenyl)-2-((triphenyl-λ^5^-phosphaneylidene)-amino)nicotinonitrile (**35**). Grey solid; yield: (87%); mp: 212–214 °C; ^1^H NMR (400 MHz, DMSO-*d*_6_) δ ppm: 7.89 (s, 6 H, Ar-H), 7.63 (d, *J* = 2.9 Hz, 3 H, Ar-H), 7.56 (s, 6 H, Ar-H), 7.27 (d, *J* = 6.9 Hz, 1H, Ar-H), 7.21 (s, 1H, Ar-H), 7.12 (s, 1H, C_5_-H of pyridine), 6.99 (s, 2 H, Ar-H), 6.87 (d, *J* = 6.9 Hz, 1H, Ar-H), 3.85 (s, 6 H, 2OCH_3_), 3.75 (d, *J* = 11.3 Hz, 6 H, 2OCH3), 3.40 (s, 3 H, OCH_3_); ^13^C APT NMR (100 MHz, DMSO-*d*_6_) δ ppm: 165.11, 157.02, 155.11, 153.26, 150.70, 148.98, 138.66, 133.31, 133.06, 132.96, 130.94, 129.70, 129.39, 129.27, 128.70, 120.57, 119.61, 111.75, 110.31, 109.06, 106.52, 96.37, 60.56, 56.53, 55.97, 55.66; ^31^P NMR (162 MHz, DMSO-*d*_6_) δ ppm: 25.64 (s), 13.67 (s); IR (ATR, ν_max_/^−1^): 3005 (CH-Ar), 2833 (CH-alipha), 2207 (CN); UV/Vis: λ_max_ 375, 290 nm; MS, *m/z*: 681.47 (M^+^); analysis (calcd., found for C_41_H_36_N_3_O_5_P): C (72.24, 72.43), H (5.32, 5. 39), N (6.16, 6.42).

4,6-Bis(3,4,5-trimethoxyphenyl)-2-((triphenyl-λ^5^-phosphaneylidene)amino)nicotinonitrile (**36**). Buff solid; yield: (90%); mp; 222–224 °C; ^1^H NMR (400 MHz, DMSO-*d*_6_) δ ppm: 7.91 (dd, *J* = 12.0, 7.6 Hz, 6 H, Ar-H), 7.64 (d, *J* = 6.7 Hz, 3 H, Ar-H), 7.57 (d, *J* = 4.8 Hz, 6 H, Ar-H), 7.31 (s, 1H, C_5_-H of pyridine), 7.00 (s, 2 H, Ar-H), 6.95 (s, 2 H, Ar-H), 3.87 (s, 6 H, 2OCH_3_), 3.75 (s, 3 H, OCH_3_), 3.67 (s, 3 H, OCH_3_), 3.54 (s, 6 H, 2OCH_3_); ^13^C APT NMR (100 MHz, DMSO-*d*_6_) δ ppm: 164.98, 164.92, 156.88, 155.37, 155.33, 153.27, 153.25, 139.36, 138.67, 133.79, 133.21, 133.04, 132.94, 129.55, 129.39, 129.27, 128.55, 119.49, 109.64, 106.57, 104.75, 97.34, 97.09, 60.57, 56.54, 56.27; ^31^P NMR (162 MHz, DMSO-*d*_6_) δ ppm: 13.28 (s); IR (ATR, ν_max_/cm^*−*1^): 3003 (CH-Ar), 2838 (CH-alipha), 2214 (CN); UV/Vis: λ_max_ 370, 295 nm; MS, *m/z*: 711.28 (M^+^); analysis (calcd., found for C_42_H_38_N_3_O_6_P): C (70.88, 71.14), H (5.38, 5.47), N (5.90, 6.12).

6-(3,4-Dimethoxyphenyl)-4-(3,5-dimethoxyphenyl)-2-((triphenyl-λ^5^-phosphaneylidene)amino)-nicotinonitrile (**37**). Grey solid; yield: (91%); mp: 135–137 °C; ^1^H NMR (400 MHz, DMSO-*d*_6_) δ ppm: 7.90 (d, *J* = 6.8 Hz, 6 H, Ar-H), 7.63 (d, *J* = 6.5 Hz, 6 H, Ar-H), 7.57 (s, 3 H, Ar-H), 7.40 (s, 1H, C_5_-H of pyridine), 7.24 (d, *J* = 5.9 Hz, 1H, Ar-H), 7.17 (s, 1H, Ar-H), 7.13 (s, 1H, Ar-H), 6.90–6.78 (m, 2 H, Ar-H), 6.63 (s, 1H, Ar-H), 3.79 (d, *J* = 18.7 Hz, 6 H, 2OCH_3_), 3.37 (d, *J* = 30.8 Hz, 6 H, 2OCH_3_); ^13^C APT NMR (100 MHz, DMSO-*d*_6_) δ ppm: 164.61, 160.42, 156.66, 154.61, 150.28, 148.54, 139.48, 132.61, 132.51, 131.56, 131.46, 130.41, 129.21, 128.96, 128.84, 128.21, 120.13, 118.86, 111.29, 109.83, 108.53, 106.48, 101.04, 95.90, 55.51, 55.42, 55.22; ^31^P NMR (162 MHz, DMSO-*d*_6_) δ ppm: 27.10 (s), 13.82 (s); IR (ATR, ν_max_/cm^*−*1^): 2967 (CH-Ar), 2835 (CH-alipha), 2208 (CN); UV/Vis: λ_max_ 370, 275 nm; MS, *m/z*: 651.55 (M^+^); analysis (calcd., found for C_40_H_34_N_3_O_4_P): C (73.72, 73.50), H (5.26, 5.39), N (6.45, 6.67).

4-(3,5-Dimethoxyphenyl)-6-(4-methoxyphenyl)-2-((triphenyl-λ^5^-phosphaneylidene)amino)-nicotinonitrile (**38**). Yellow solid; yield: (88%) ; mp: 128–130 °C; ^1^H NMR (400 MHz, DMSO-d_6_) δ ppm: 7.86 (dd, *J* = 11.5, 7.9 Hz, 6 H, Ar-H), 7.64 (d, *J* = 6.8 Hz, 3 H, Ar-H), 7.58 (d, *J* = 5.9 Hz, 6 H, Ar-H), 7.41 (d, *J* = 8.4 Hz, 2 H, Ar-H), 7.11 (s, 1H, C_5_-H of pyridine), 6.81 (d, *J* = 6.3 Hz, 2 H, Ar-H), 6.78 (s, 2 H, Ar-H), 6.63 (s, 1H, Ar-H), 3.81 (s, 6 H, 2OCH_3_), 3.76 (s, 3 H, OCH_3_); ^13^C APT NMR (100 MHz, DMSO-*d*_6_) δ ppm: 164.66, 160.56, 160.42, 156.61, 154.64, 139.48, 132.56, 132.46, 131.55, 131.46, 130.34, 129.27, 128.98, 128.86, 128.51, 128.27, 118.86, 113.72, 108.26, 106.43, 101.11, 95.82, 95.58, 55.41, 55.24; ^31^P NMR (162 MHz, DMSO-*d*_6_) δ ppm: 26.09 (s), 14.09 (s); IR (ATR, ν_max_/cm^*−*1^): 3012 (CH-Ar), 2840 (CH-alipha) 2207 (CN); UV/Vis: λ_max_ 370, 265 nm; MS, *m/z*: 621.40 (M^+^); analysis (calcd., found for C_39_H_32_N_3_O_3_P): C (75.35, 75.19), H (5.19, 5.30), N (6.76, 6.94).

6-(3,4-Dimethoxyphenyl)-4-(4-methoxyphenyl)-2-((triphenyl-λ^5^-phosphaneylidene)amino)-nicotinonitrile (**39**). brown solid; yield: (86%); mp: 129–131 °C; ^1^H NMR (400 MHz, DMSO-d6) δ ppm: 7.90 (s, 6 H, Ar-H), 7.61 (d, *J* = 25.3 Hz, 9 H, Ar-H), 7.41 (s, 3 H, Ar-H), 7.25 (s, 2 H, Ar-H), 7.12 (s, 2 H, Ar-H), 6.87 (s, 1H, C_5_-H of pyridine), 3.81 (d, *J* = 26.1 Hz, 6 H, 2OCH_3_), 3.41 (s, 3 H, OCH_3_); ^13^C APT NMR (100 MHz, DMSO-*d*_6_) δ ppm: 164.67, 160.12, 156.47, 154.20, 150.16, 148.49, 132.57, 132.47, 132.32, 131.51, 131.41, 130.49, 129.68, 129.25, 128.89, 128.72, 128.68, 128.26, 119.96, 119.11, 114.03, 111.25, 109.79, 108.47, 95.63, 55.46, 55.30, 55.17; ^31^P NMR (162 MHz, DMSO-*d*_6_) δ ppm: 26.03 (s), 13.73 (s); IR (ATR, ν_max_/cm^*−*1^): 3032 (CH-Ar), 2931 (CH-alipha), 2202 (CN); UV/Vis: λ_max_ 370, 265 nm; MS, *m/z*: 621.00 (M^+^); analysis (calcd., found for C_39_H_32_N_3_O_3_P): C (75.35, 75.12), H (5.19, 5.40), N (6.76, 7.04).

4-(Furan-2-yl)-6-(3,4,5-trimethoxyphenyl)-2-((triphenyl-λ^5^-phosphaneylidene)amino)nicotino-nitrile (**40**). Yellow solid; yield: (86%); mp: 230–232 °C; ^1^H NMR (400 MHz, DMSO-*d*_6_) δ ppm: 8.01 (d, *J* = 1.1 Hz, 1H, furyl-H), 7.89 (dd, *J* = 12.2, 7.3 Hz, 6 H, Ar-H), 7.64 (t, *J* = 7.3 Hz, 3 H, Ar-H), 7.60–7.52 (m, 6 H, Ar-H), 7.48 (s, 1H, C_5_-H of pyridine), 7.21 (dd, *J* = 27.4, 7.4 Hz, 1H, furyl-H), 6.93 (s, 2 H, Ar-H), 6.80 (dd, *J* = 3.5, 1.7 Hz, 1H, furyl-H), 3.68 (s, 3 H, OCH_3_), 3.56 (s, 6 H, 2OCH_3_); ^13^C APT NMR (100 MHz, DMSO-*d*_6_) δ ppm: 165.40, 157.21, 153.32, 149.43, 148.33, 141.97, 139.50, 133.76, 133.67, 133.07, 132.97, 129.55, 129.41, 129.29, 128.56, 119.47, 113.25, 104.60, 92.42, 60.57, 56.28; ^31^P NMR (162 MHz, DMSO-*d*_6_) δ ppm: 28.17 (s), 14.19 (s); IR (ATR, ν_max_/cm^*−*1^): 3012 (CH-Ar), 2934 (CH-alipha), 2207 (CN); UV/Vis: λ_max_ 385, 300 nm; MS, *m/z*: 611.45 (M^+^); analysis (calcd., found for C_37_H_30_N_3_O_4_P): C (72.66, 72.52), H (4.94, 5.11), N (6.87, 7.05).

4-(Furan-2-yl)-6-methyl-2-((triphenyl-λ^5^-phosphaneylidene)amino)nicotinonitrile (**41**). Cocoa solid; yield: (88%); mp: 222–224 °C; ^1^H NMR (400 MHz, DMSO-*d*_6_) δ ppm: 7.93 (s, 1H, C_5_-H of pyridine), 7.86 (dd, *J* = 12.0, 7.3 Hz, 6 H, Ar-H), 7.67–7.61 (m, 3 H, Ar-H), 7.60–7.53 (m, 6 H, Ar-H), 7.40 (d, *J* = 3.4 Hz, 1H, furyl-H), 6.80 (s, 1H, furyl-H), 6.73 (d, *J* = 1.6 Hz, 1H, furyl-H), 2.04 (s, 3 H, CH_3_); ^13^C APT NMR (100 MHz, DMSO-*d*_6_) δ ppm: 160.04, 144.99, 140.67, 132.84, 132.74, 132.27, 128.93, 128.74, 128.62, 127.93, 119.08, 112.55, 112.29, 106.74, 23.71; ^31^P NMR (162 MHz, DMSO-*d*_6_) δ ppm: 14.19 (s); IR (ATR, ν_max_/cm^*−*1^): 3054 (CH-Ar), 2840 (CH-alipha), 2206 (CN); UV/Vis: λ_max_ 360, 295 nm; MS, *m/z*: 459.13 (M^+^); analysis (calcd., found for C_29_H_22_N_3_OP): C (75.81, 75.68), H (4.83, 4.97), N (9.15, 9.41).

About the characterization data of all the final synthesized compounds (^1^H-NMR, ^13^C-APT NMR, IR spectra, UV-Vis analyses, Mass spectra and Elemental analyses), see supplementary information (Figs. S1-S161).

### Biological assay

#### In vitro anticancer activity

##### Materials and methods

Roswell Park Memorial Institute (RPMI) 1640 medium was purchased from sigma Chem. Co. (St. Louis, MO, USA). Fetal bovine serum (FBS) and fetal calf serum (FCS) were purchased from Gibco, UK. Dimethyl sulfoxide (DMSO) and methanol were of HPLC grade, and all other reagents and chemicals were of analytical reagent grade.

##### Cell culture

HepG2 (Human liver carcinoma), HCT116 (human colorectal carcinoma), MCF7 (human breast adenocarcinoma), and the normal human skin fibroblast (BJ-1) cell lines were purchased from the american type culture collection (Rockville, MD, USA) and maintained in RPMI-1640 medium which was supplemented with 10% heat-inactivated FBS, 100U/ml penicillin and 100U/ml streptomycin. The cells were grown at 37 °C in a humidified atmosphere of 5% CO_2_. All experiments were conducted thrice in triplicate (*n* = 3). All the values were represented as means ± SD. Significant differences between the means of parameters as well as IC_50_s were determined by probit analysis using SPSS software program (SPSS Inc., Chicago, IL).

##### Lactate dehydrogenase (LDH) assay

To determine the effect of each synthesized compound on membrane permeability in HepG2, MCF-7 and HCT116 cancer cell lines as well as BJ-1 normal cell line, a lactate dehydrogenase (LDH) release assay was used^29–33^. The cells were seeded in 24-well culture plates at a density of 1 × 10^4^ cells/well in 500 µL volume and allowed to grow for 18 h before treatment. After treatment with a series of different concentrations of each compound or DOX (positive control), the plates were incubated for 48 h. Then, the supernatant (40 µL) was transferred to a new 96 well to determine LDH release and 6% triton X-100 (40 µL) was added to the original plate for determination of total LDH. An aliquot of 0.1 M potassium phosphate buffer (100 µL, pH 7.5) containing 4.6 mM pyruvic acid was mixed to the supernatant using repeated pipetting. Then, 0.1 M potassium phosphate buffer (100 µL, pH 7.5) containing 0.4 mg/mL reduced β-NADH was added to the wells. The kinetic changes were read for 1 min using ELISA microplate reader in absorbance at wavelength 340 nm. This procedure was repeated with 40 µL of the total cell lysate to determine total LDH. The percentage of LDH release was determined by dividing the LDH released into the media by the total LDH following cell lysis in the same well. The cell Passage numbers for MCF 7 = P13, Hep G2 = P15, HCT 116 = P 9.

##### Statistical analysis

All experiments were conducted in triplicate (*n* = 3). All the values were represented as mean ± SD. Significant differences between the means of parameters as well as IC_50_s were determined by probit analysis using SPSS software program (SPSS Inc., Chicago, IL).

#### Inhibition of tubulin polymerization in MCF-7 cells

##### Materials

Beta-tubulin in vitro simple step ELISA^®^ (Enzyme-Linked Immunosorbent Assay) kit is designed for quantitative measurement of beta-tubulin protein in human cell and tissue homogenate extract samples. This is performed for most active compounds **20**,** 22**,** 24**,** 26**,** 27**,** 33**,** 35**,** 37**,** 38**,** 41** and CA-4 as control against MCF-7 cancer cell line to measure the percentage of β-tubulin polymerization inhibition.

##### Methodology

The SimpleStep ELISA^®^ employs an affinity tag labeled capture antibody and a reporter conjugated detector antibody which immunocapture the sample analyte in solution. This entire complex (capture antibody/analyte/detector antibody) is in turn immobilized *via* immunoaffinity of an anti-tag antibody coating the well. To perform the assay, samples or standards are added to the wells, followed by the antibody mix. After incubation, the wells are washed to remove unbound material. TMB development solution is added and during incubation is catalyzed by horseradish peroxidase (HRP), generating blue coloration. This reaction is then stopped by addition of stop solution completing any color change from blue to yellow. Signal is generated proportionally to the amount of bound analyte and the intensity is measured at 450 nm. Optionally, instead of the endpoint reading, development of TMB can be recorded kinetically at 600 nm. The concentration of beta-tubulin protein in the samples is determined by comparing the optical density (OD) of the samples to the standard curve. Each experiment was repeated two times (Table 2).

#### Inhibition of topoisomerase II in MCF-7 cells

##### Materials

Mouse TOP2B/Topoisomerase II Beta ELISAKit (Sandwich ELISA) is designed for the quantitative measurement of Topo II enzyme. This is performed for most active compounds **20**,** 22**,** 24**,** 26**,** 37** and DOX as control against MCF-7 cancer cell line to measure the percentage of Topo II enzyme inhibition.

##### Methodology

This assay is based on the sandwich ELISA principle. Each well of the supplied microtiter plate has been pre-coated with a target specific capture antibody. Standards or samples are added to the wells, and the target antigen binds to the capture antibody. Unbound standard or sample is washed away. A biotin-conjugated detection antibody is then added which binds to the captured antigen. Unbound detection antibody is washed away. An avidin-horseradish peroxidase (HRP) conjugate is then added which binds to the biotin. Unbound avidin-HRP conjugate is washed away. A TMB substrate is then added which reacts with the HRP enzyme resulting in color development. A sulfuric acid stop solution is added to terminate color development reaction and then the optical density (OD) of the well is measured at a wavelength of 450 nm ± 2 nm. An OD standard curve is generated using known antigen concentrations; the OD of an unknown sample can then be compared to the standard curve to determine its antigen concentration. Each experiment was repeated two times (Table 3).

#### In vitro propidium iodide flow cytometry cell cycle analysis

##### Materials

Propidium iodide flow cytometry kit (ab139418) is designed for quantitative DNA content analysis in tissue culture cells, so we performed propidium iodide cell cycle analysis for active compounds **20 and 26** using MCF-7 cell line. Propidium iodide staining of DNA is the classic means of cell cycle analysis. The staining procedure takes less than 1 h of total processing time and cells fixed in ethanol are stable for at least several weeks at 4 °C.

##### Methodology

Propidium iodide is a fluorescent molecule that binds nucleic acid with little or no sequence preference. Because propidium iodide binds RNA as well as DNA, RNaseA (ribonuclease A) is included in this kit to digest cellular RNA and thus decrease background RNA staining from the experiment. Since propidium iodide is membrane impermeant, ethanol is used to both fix and permeabilize cells. A flow cytometer is required for quantitative analysis. First, fix MCF-7 cells in 66% ethanol, store at + 4 °C for 2 h to 4 weeks and rehydrate cells in PBS. Finally, stain cells with propidium iodide then add RNase for 30 min. Collect propidium iodide fluorescence intensity on FL2 of a flow cytometer and 488nM laser excitation. A useful way to display propidium iodide data is on a histogram with the cell count on the y-axis and the propidium iodide fluorescence intensity on the x-axis.

#### Apoptosis and necrosis analysis by annexin V-FITC/PI staining

##### Materials

Annexin V apoptosis detection kit is used for analyzing apoptosis and necrosis for active compounds **20 and 26** in MCF7 cells using annexin V-FITC/PI dual staining followed by flow cytometry quantification and fluorescence microscopy detection.

##### Methodology

Detection is based on the observation that soon after initiating apoptosis, cells translocate the membrane phosphatidylserine (PS) from the inner face of the plasma membrane to the cell surface. Once on the cell surface, PS can be easily detected by staining with a fluorescent conjugate of annexin V, a protein that has a high affinity for PS. Detection can be analyzed by flow cytometry or/and by fluorescence microscopy. The kit can differentiate between apoptosis and necrosis when performing both annexin V-FITC and PI staining. Assay is summarized as first, induce apoptosis by desired method, collect 1–5 × 10^5^ cells by centrifugation, resuspend cells in 500 µl of 1× binding buffer, add 5 µl of annexin V-FITC and 5 µl of propidium iodide and finally incubate at room temperature for 5 min in the dark. After that, analyze annexin V-FITC binding by flow cytometry (Ex = 488 nm; Em = 530 nm) using FITC signal detector (usually FL1) and PI staining by the phycoerythrin emission signal detector (usually FL2) then, observe the cells under a fluorescence microscope using a dual filter set for FITC & rhodamine. Cells that have bound annexin V-FITC will show green staining in the plasma membrane. Cells that have lost membrane integrity will show red staining (PI) throughout the nucleus and a halo of green staining (FITC) on the cell surface.

#### In silico study

About the general computational modelling and docking in addition to DFT calculations, see supplementary file S4.2.6 part.

## Supplementary Information

Below is the link to the electronic supplementary material.

Supplementary Material 1

## Data Availability

Data provided within the manuscript or supplementary information files. The datasets generated and/or analyzed during the current study are available in the Zenodo repository: https://doi.org/10.5281/zenodo.17422196.
