# Supplementary material for: Nicotinonitrile based dual inhibitors of tubulin and topoisomerase II exhibit potent anticancer activity
Source: Sci Rep. 2026 Jun 25;16:19595. doi: 10.1038/s41598-026-47995-5 (PMC13303826; doi:10.1038/s41598-026-47995-5)
Supplement: Supplementary file 1 — Supplementary Material 1 [file 41598_2026_47995_MOESM1_ESM.docx]

**Supplementary information**

**Nicotinonitrile Based Dual Inhibitors of Tubulin and Topoisomerase II Exhibit Potent Anticancer Activity**

Eman Samir^1,3^, Abdalla E. A. Hassan^1,2^, Shaikha Alneyadi^4^, Yassir S. Raouf^4^, Hanem M. Awad^5^, Zakaria K.M. Abdel-Samii^3^, Amany M.M. Al-Mahmoudy^3^, Reham A. Abou-elkhair^1,2^.

^1^Applied Nucleic Acids Research center, Zagazig University, Zagazig Egypt. ^2^Department of Chemistry, Faculty of Science, Zagazig University, Zagazig, Egypt. ^3^Department of Pharmaceutical Organic Chemistry, Faculty of Pharmacy, Zagazig University, Zagazig, Egypt. ^4^Department of Chemistry, College of Science, United Arab Emirates University, P.O. Box 15551, Al Ain, UAE. ^5^National Research Centre, Tanning Materials & Leather Technology Department, Dokki, Giza 12622, Egypt.

**NMR spectra**


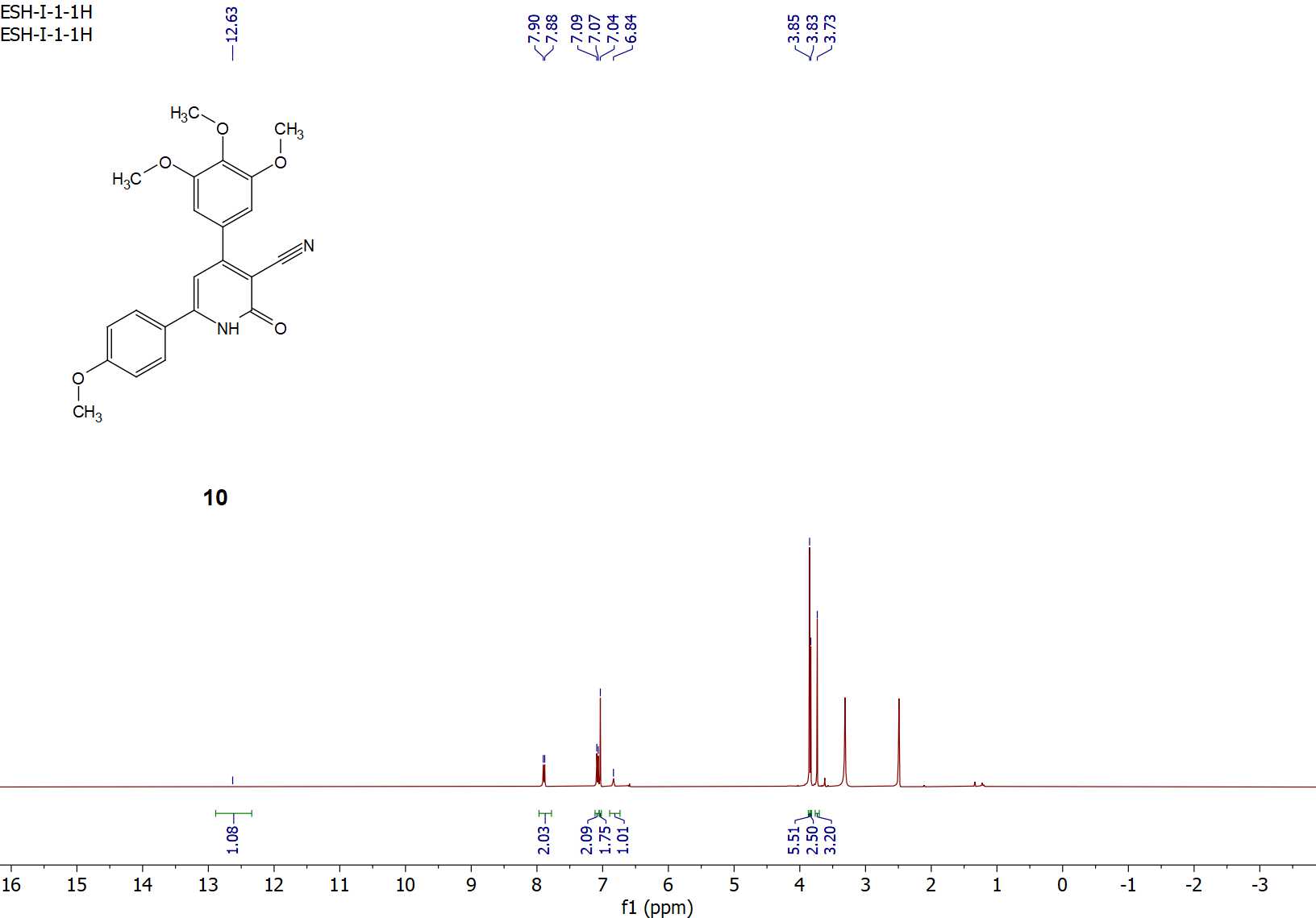


**Fig. S1:** ^1^H-NMR spectrum of compound 10.


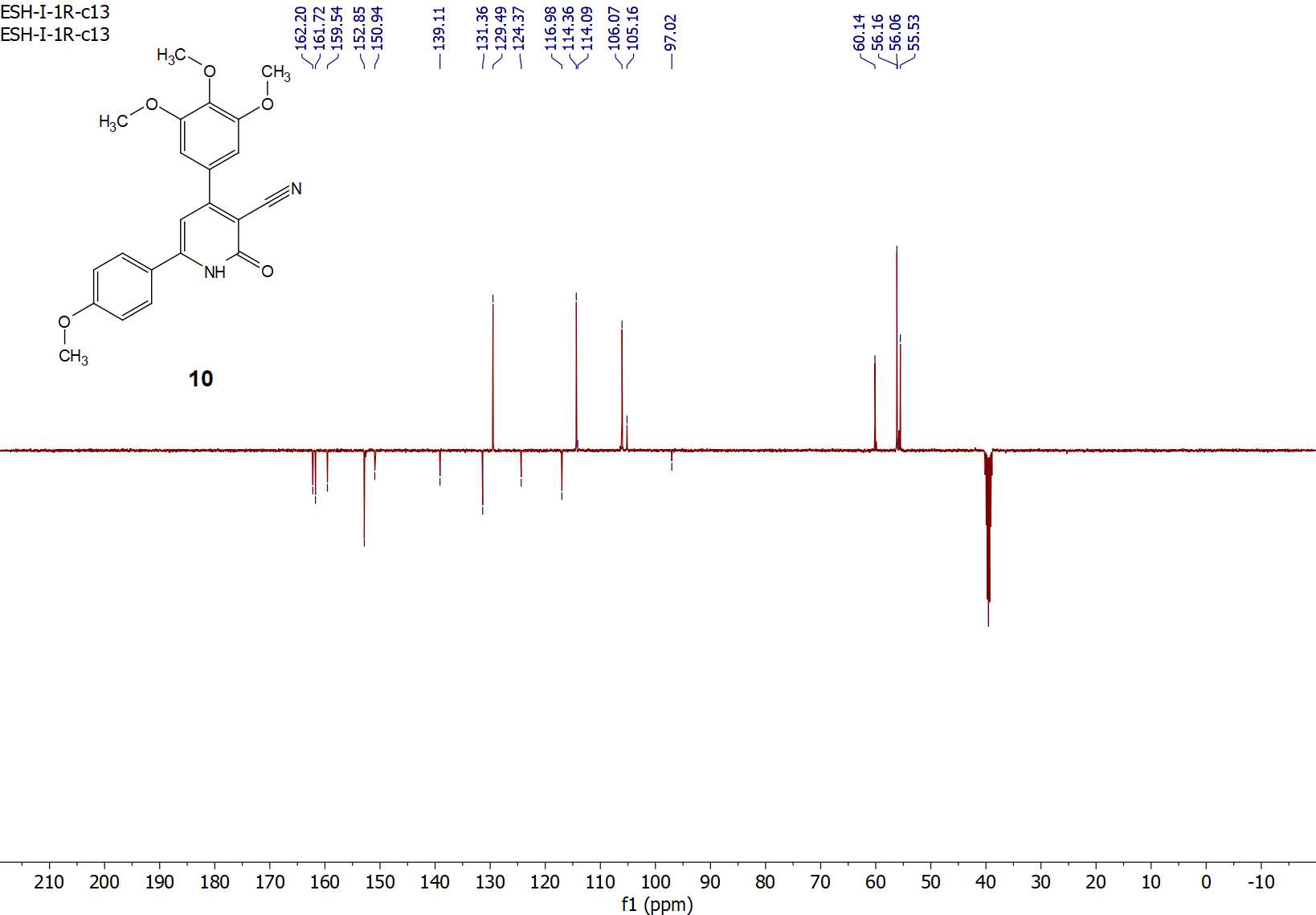


**Fig. S2:** ^13^C-APT NMR spectrum of compound 10.

**
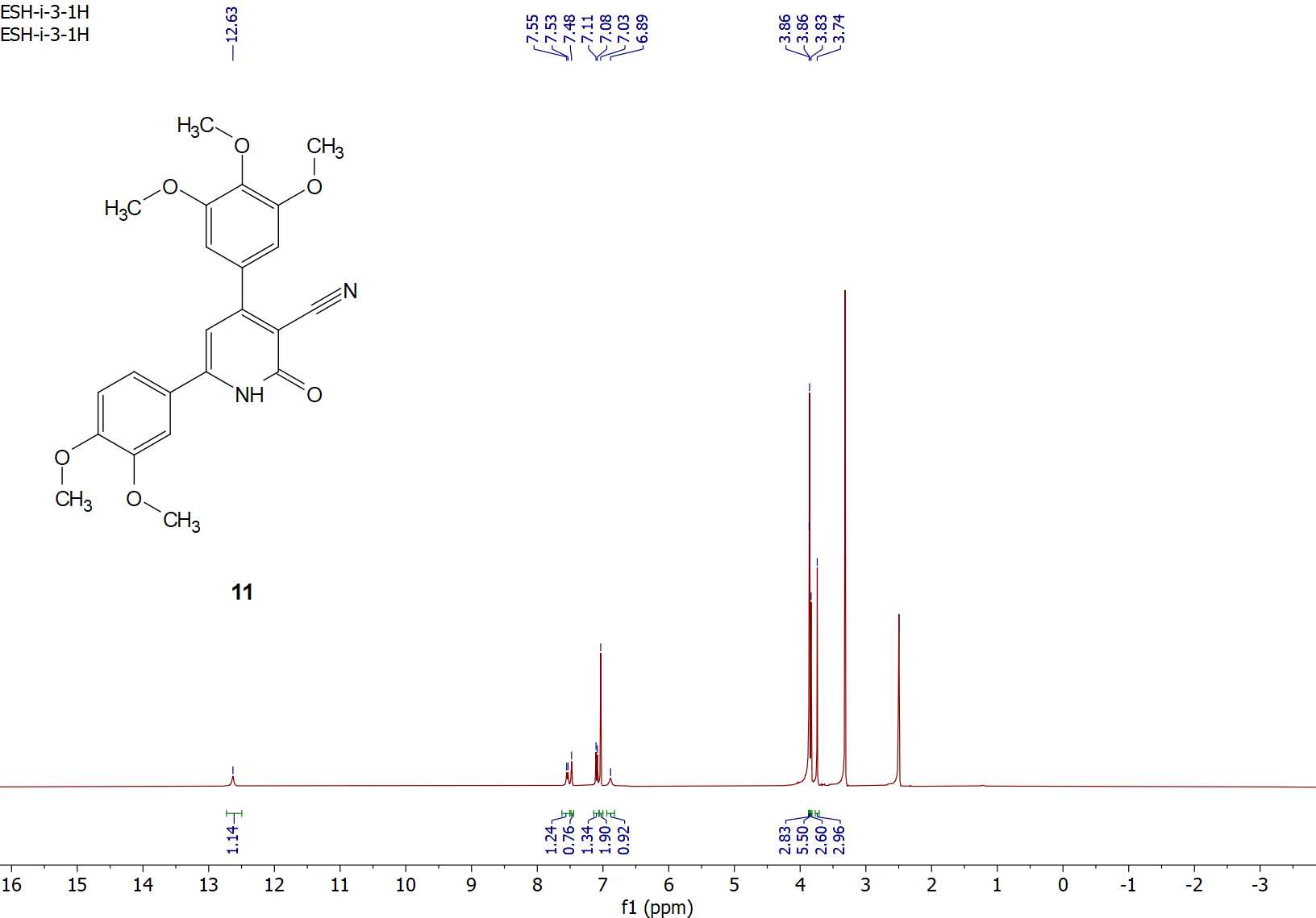
**

**Fig. S3:** ^1^H-NMR spectrum of compound 11.


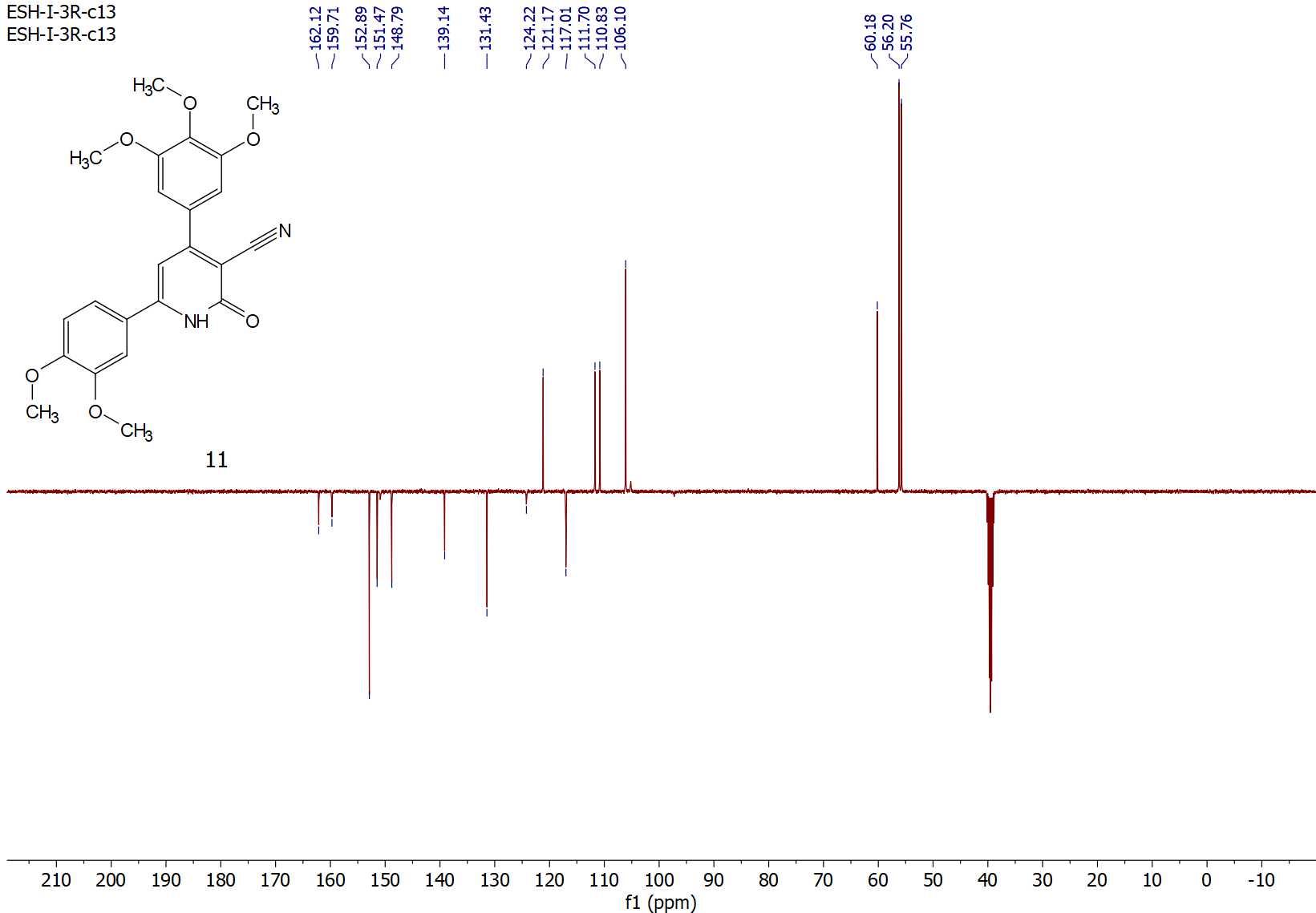


**Fig. S4:** ^13^C-APT NMR spectrum of compound 11.


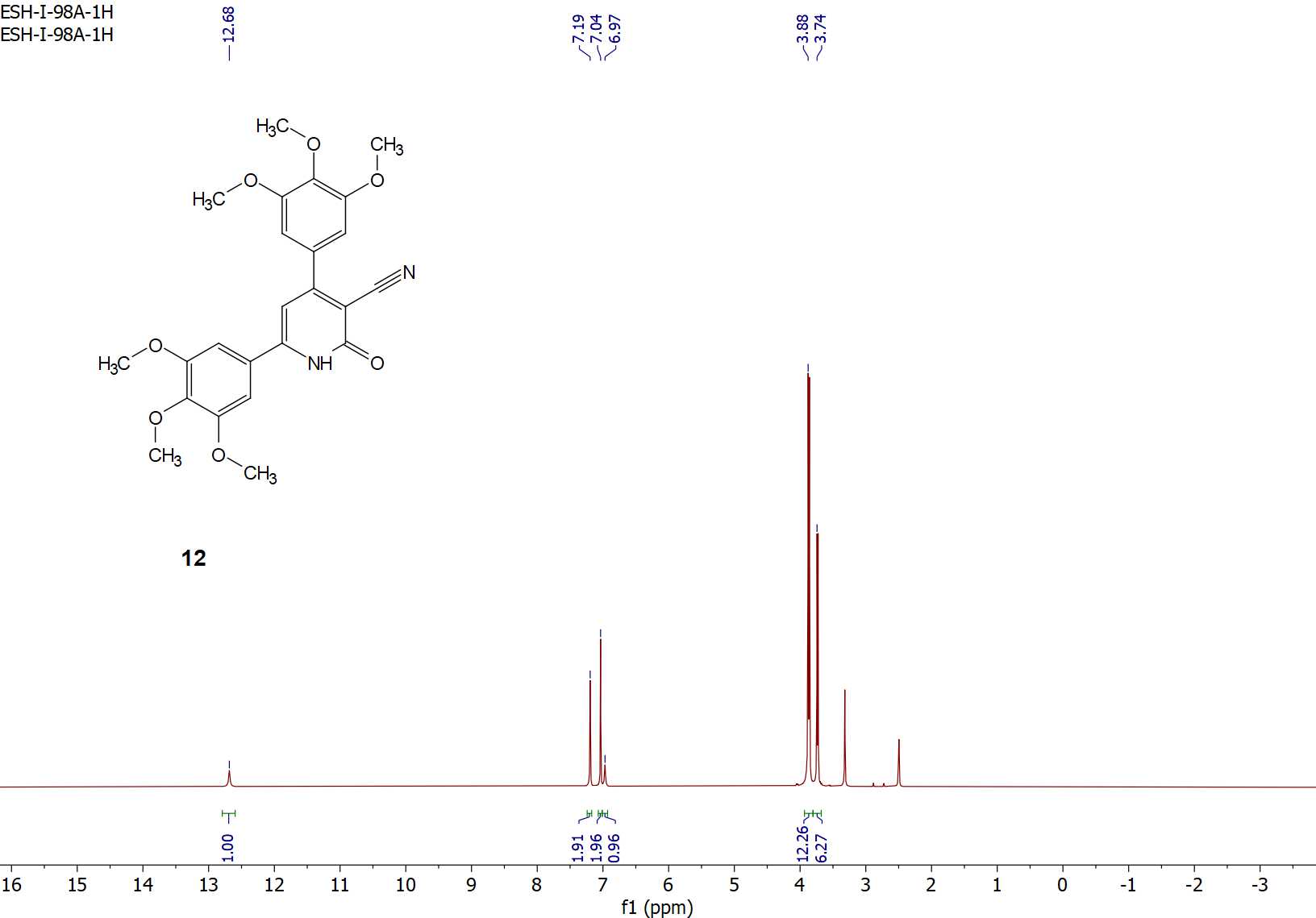


**Fig. S5:** ^1^H-NMR spectrum of compound 12.


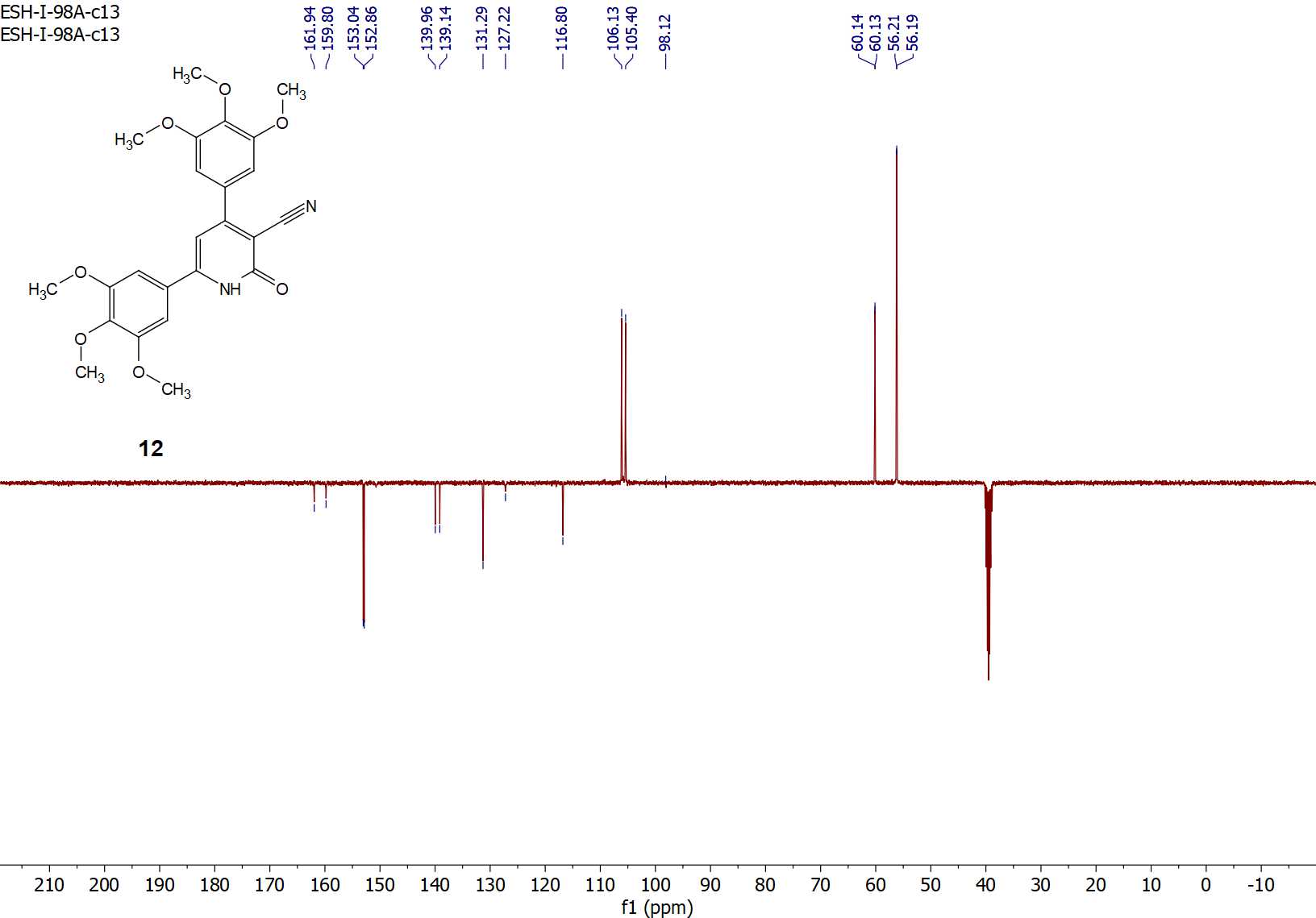


**Fig. S6:** ^13^C-APT NMR spectrum of compound 12.


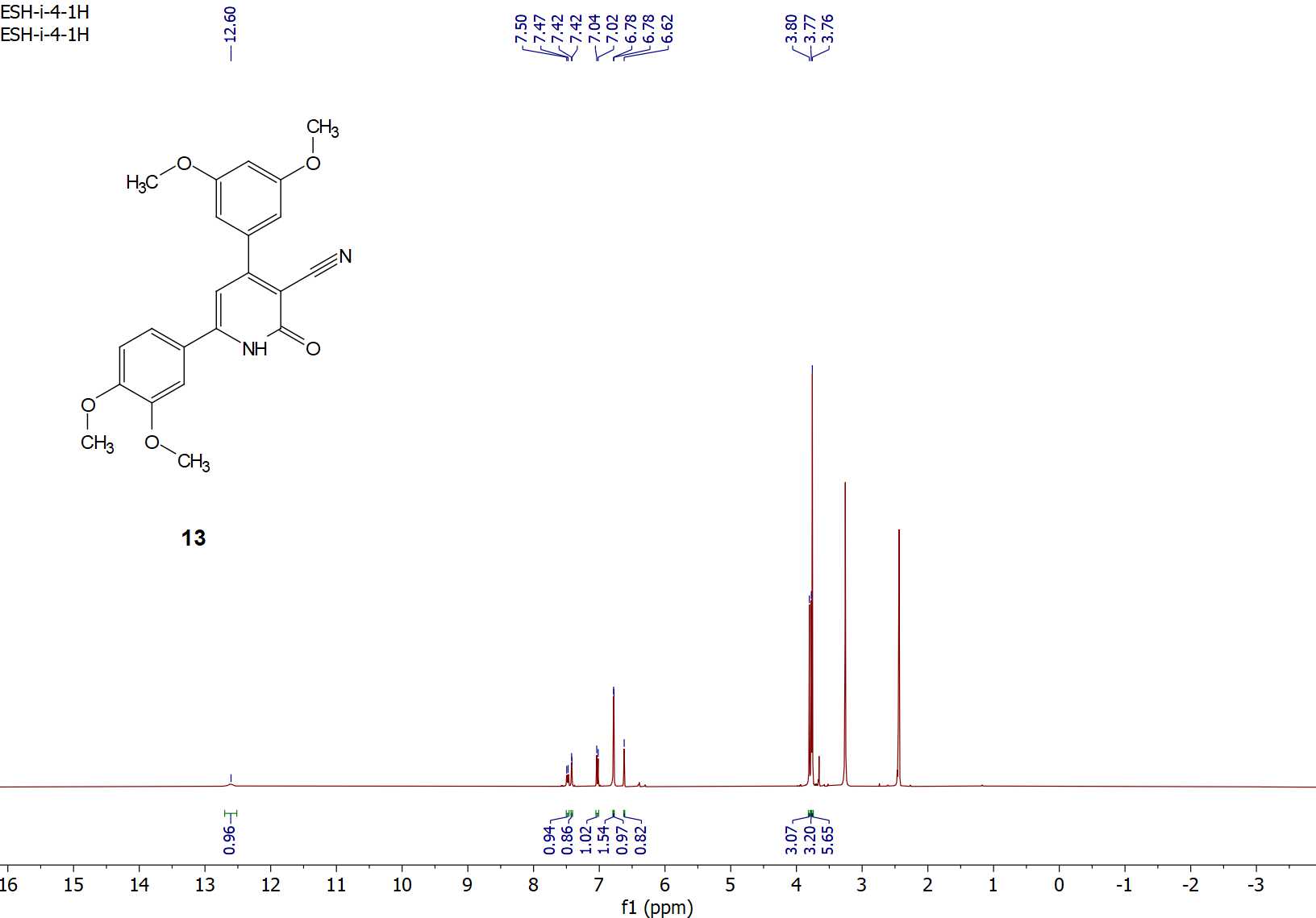


**Fig. S7:** ^1^H-NMR spectrum of compound 13.


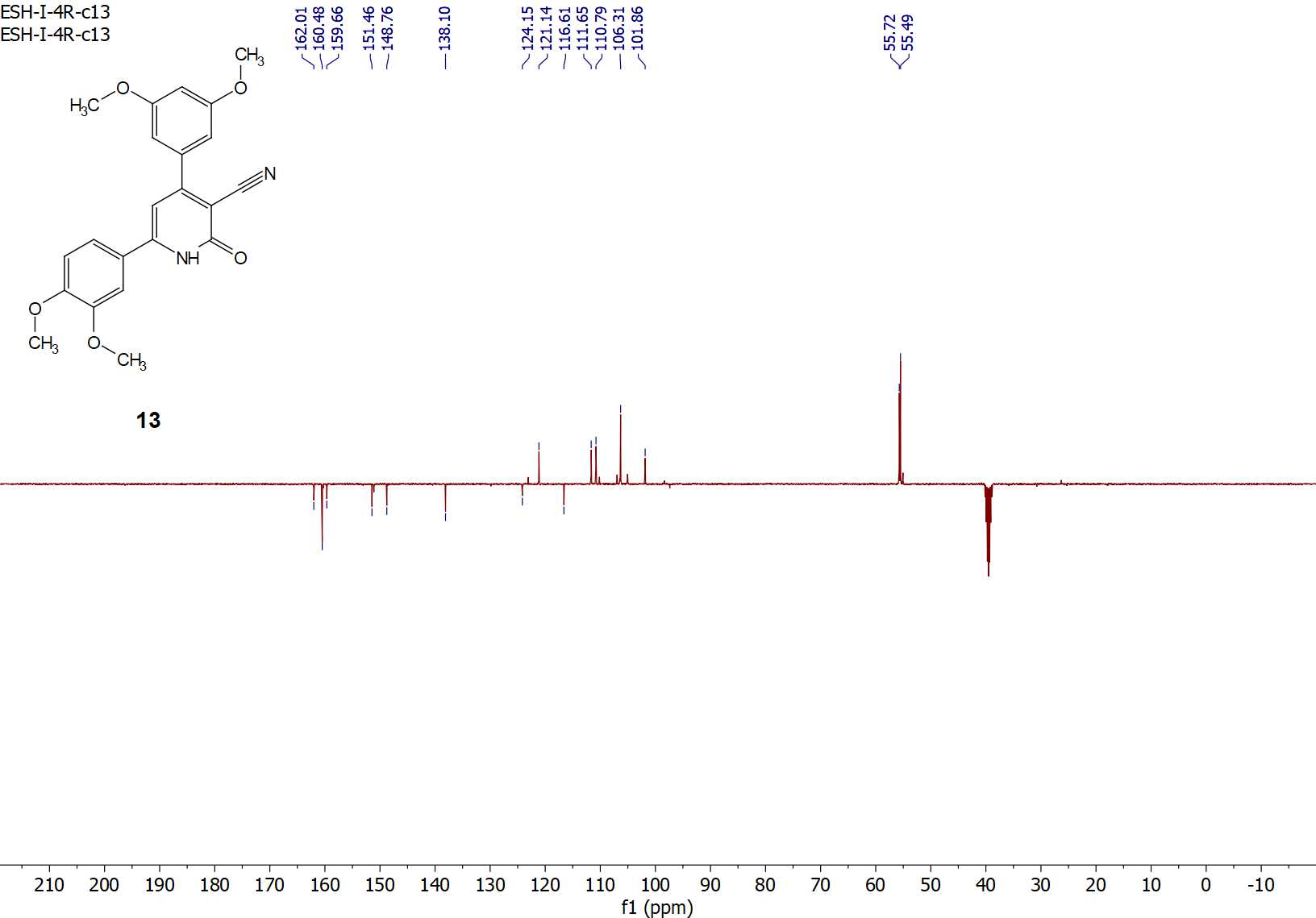


**Fig. S8:** ^13^C-APT NMR spectrum of compound 13.


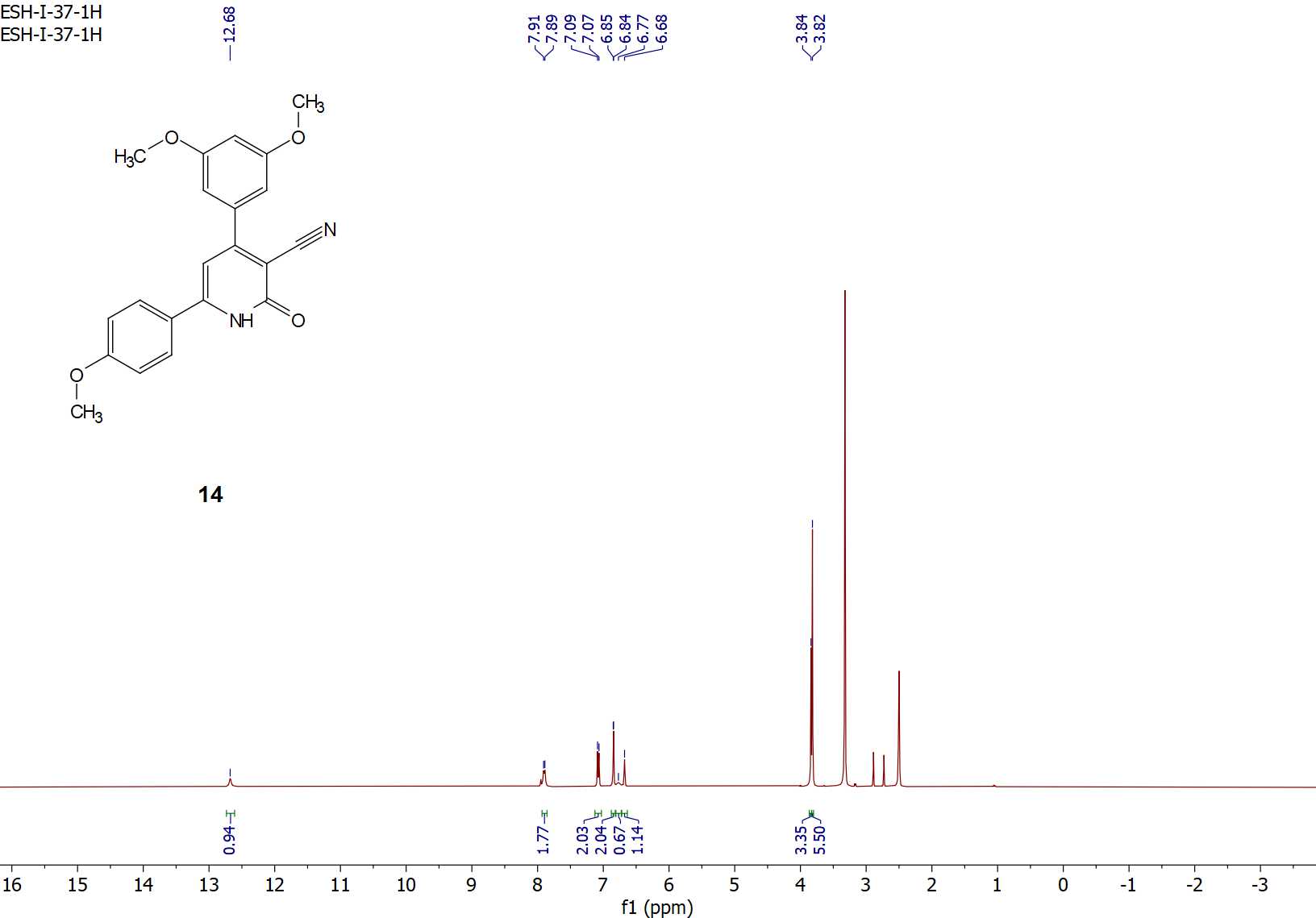


**Fig. S9:** ^1^H-NMR spectrum of compound 14.


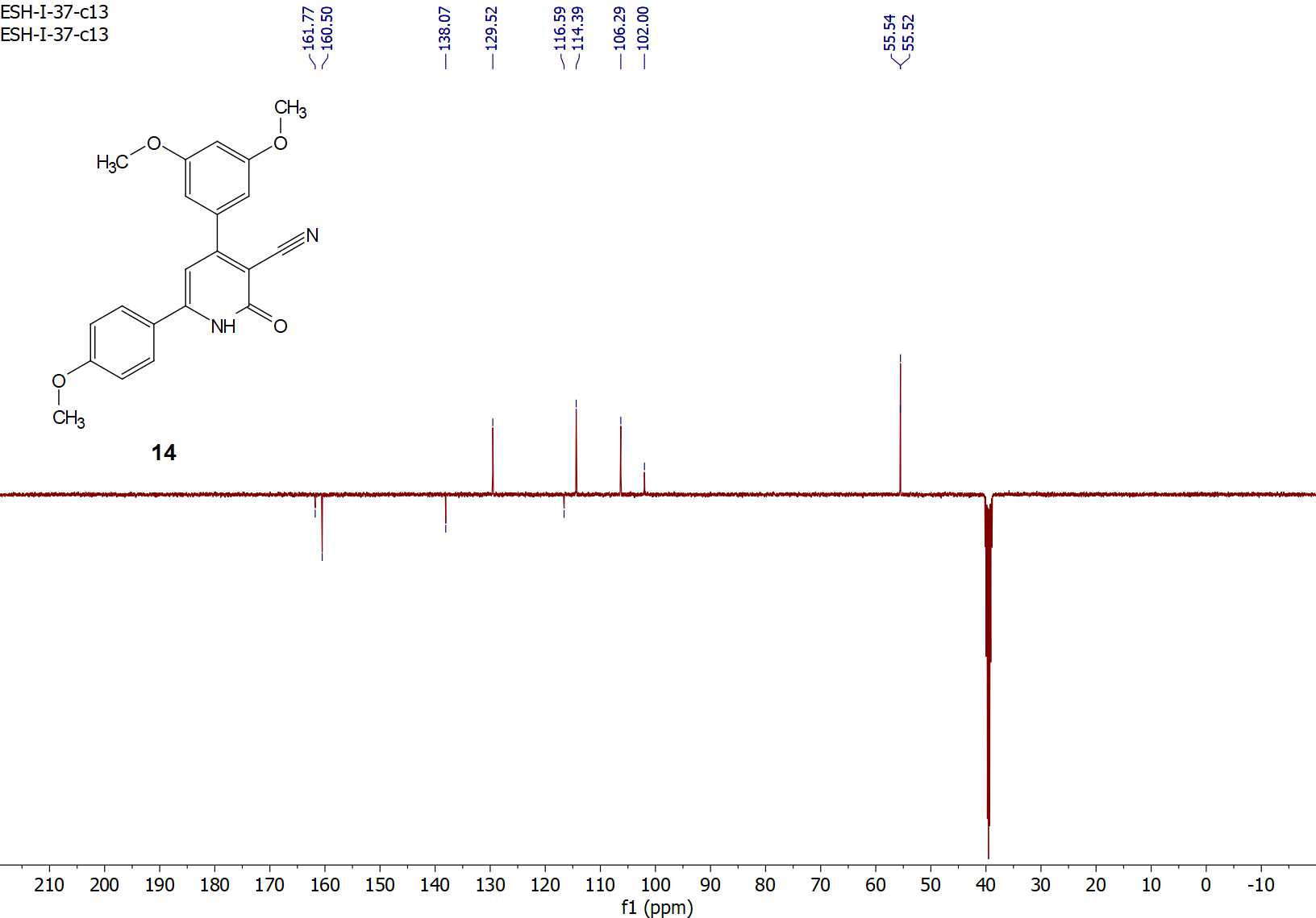


**Fig. S10:** ^13^C-APT NMR spectrum of compound 14.


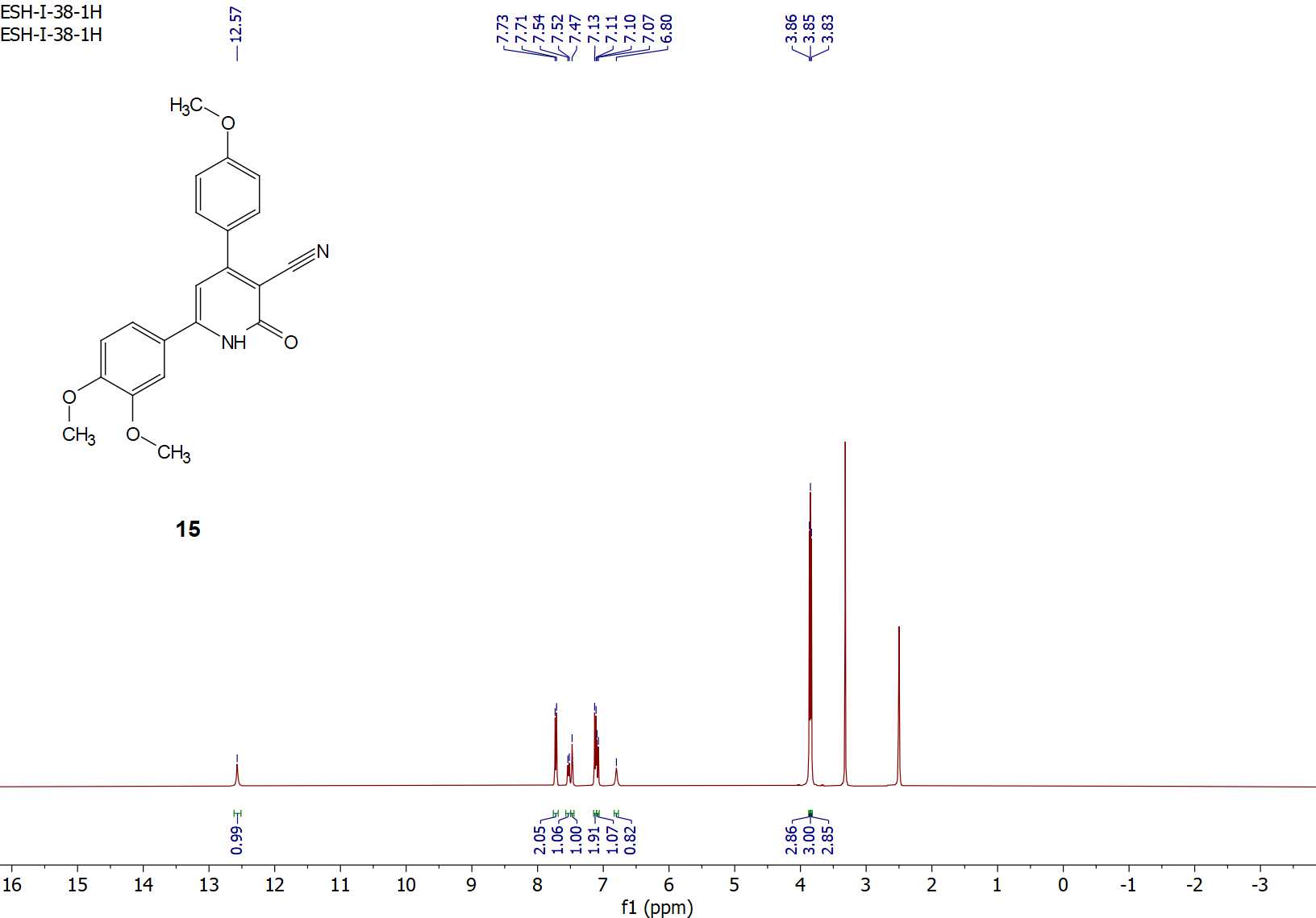


**Fig. S11:** ^1^H-NMR spectrum of compound 15.


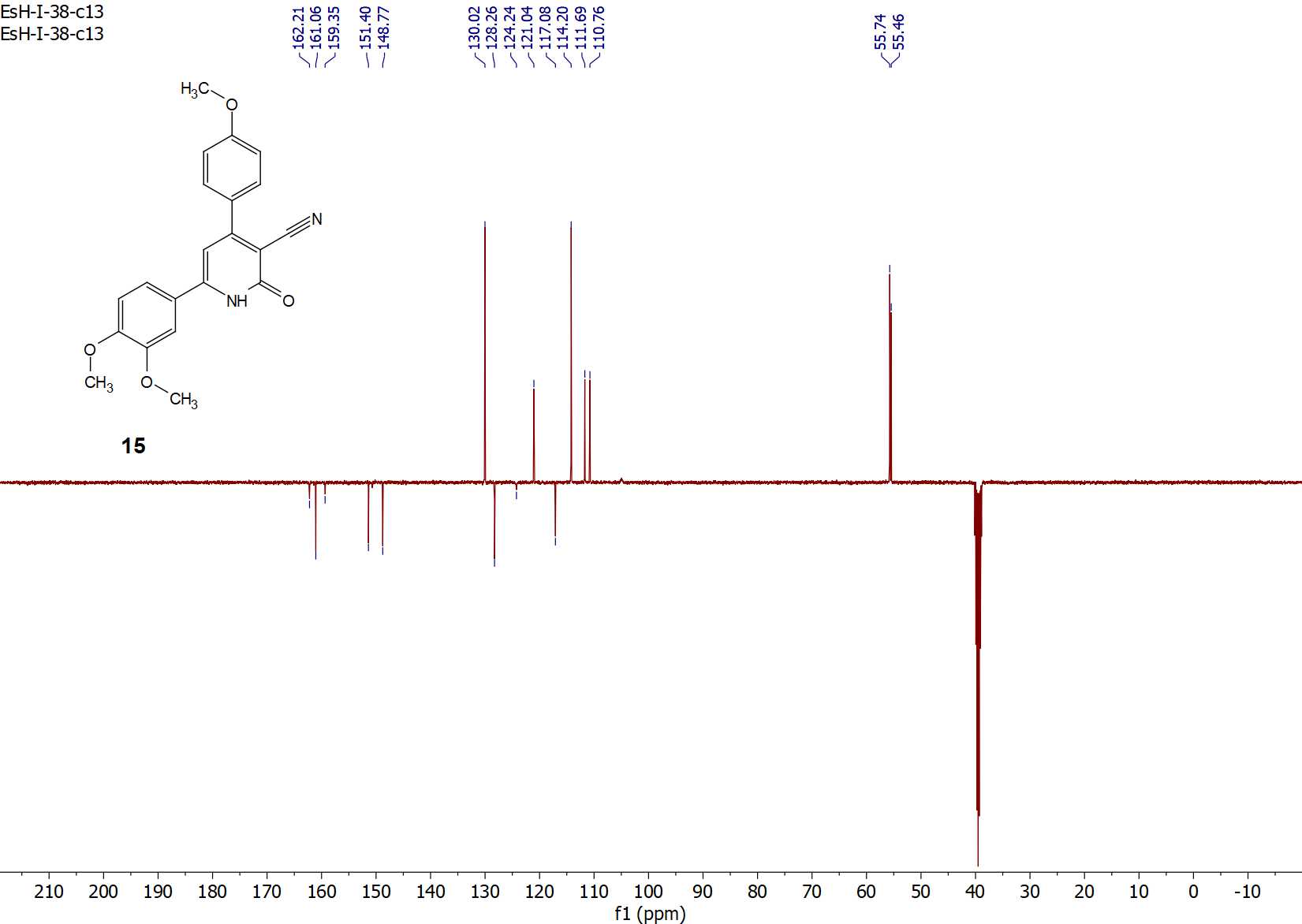


**Fig. S12:** ^13^C-APT NMR spectrum of compound 15.


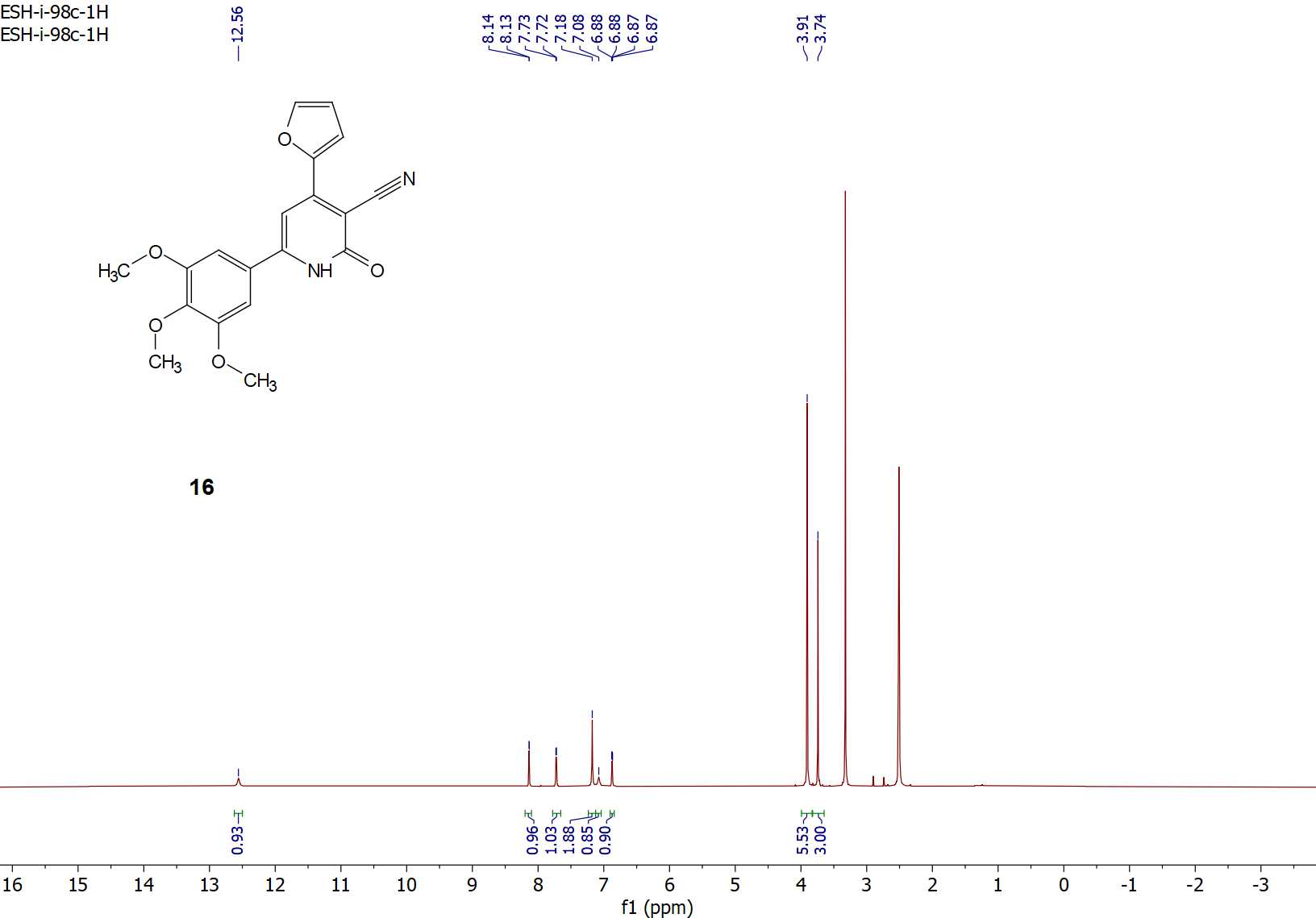


**Fig. S13:** ^1^H-NMR spectrum of compound 16.


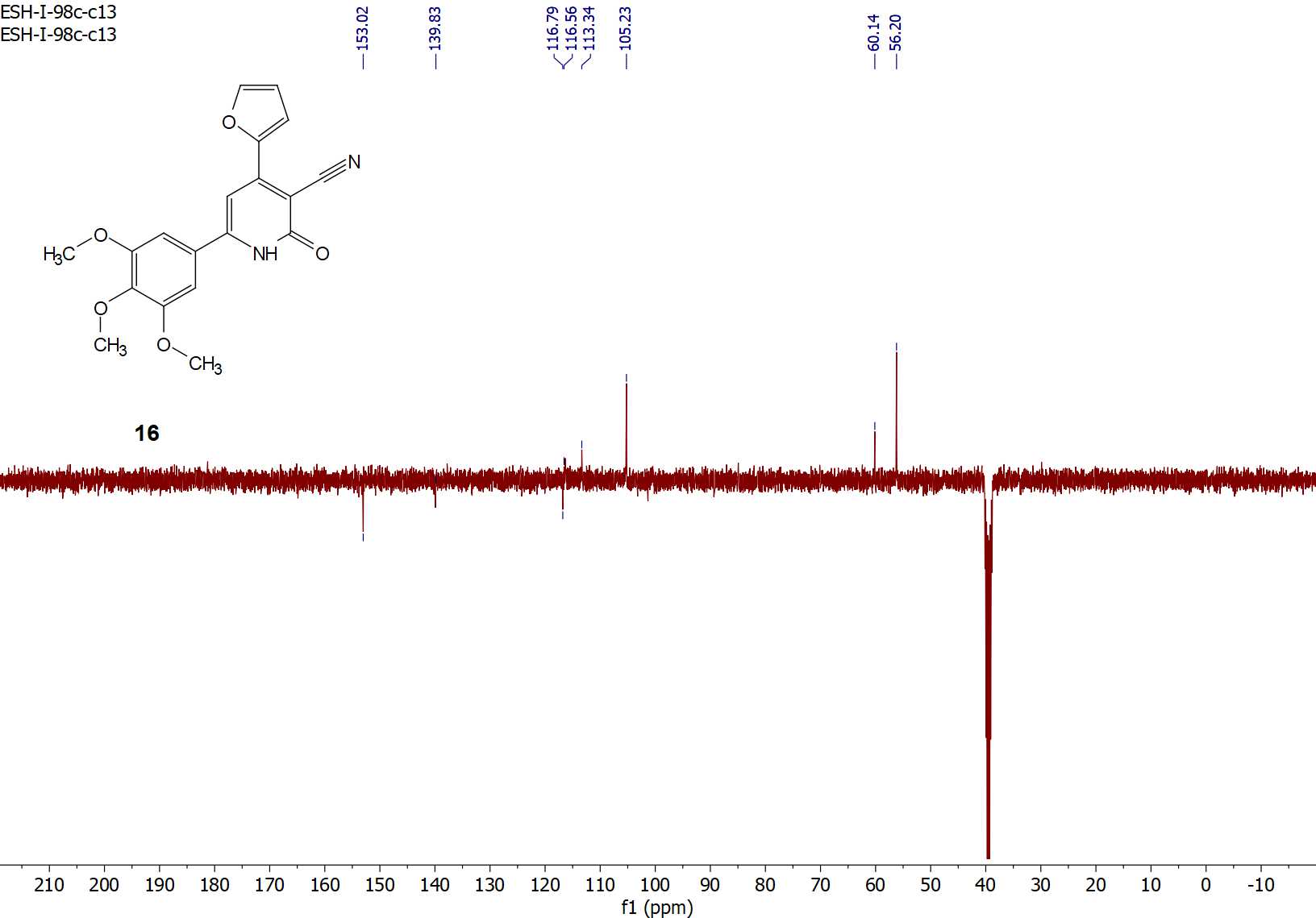


**Fig. S14:** ^13^C-APT NMR spectrum of compound 16.


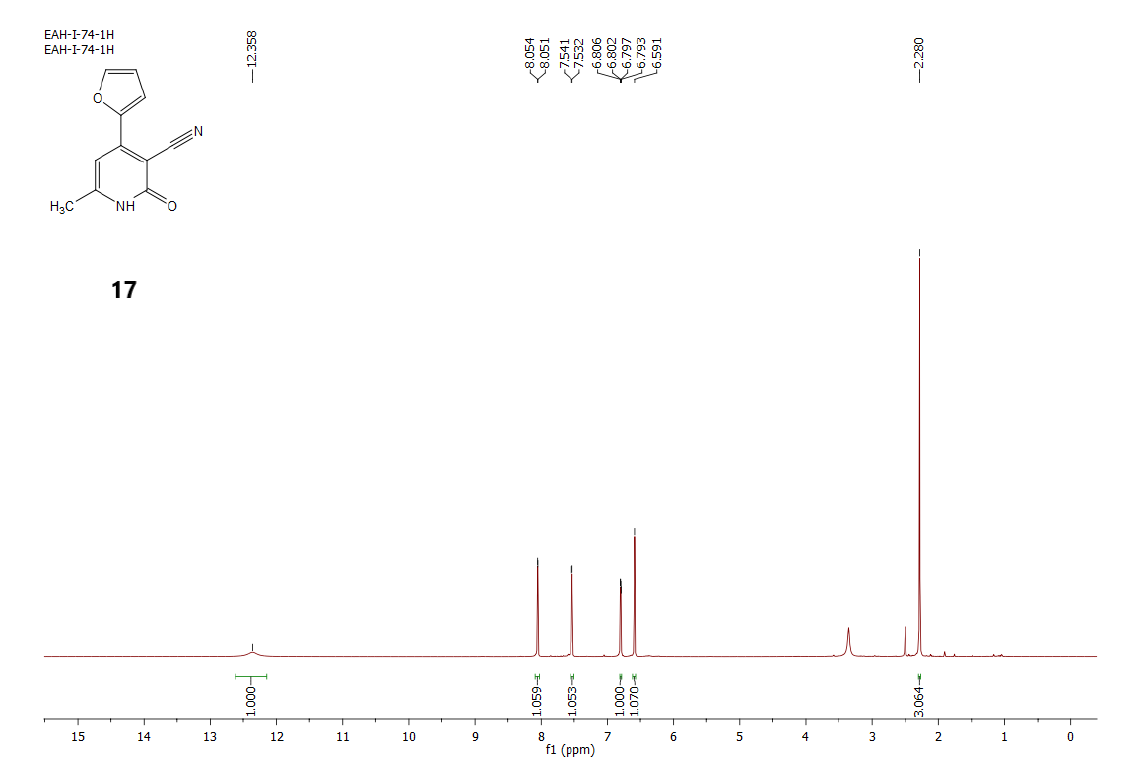


**Fig. S15:** ^1^H-NMR spectrum of compound 17.


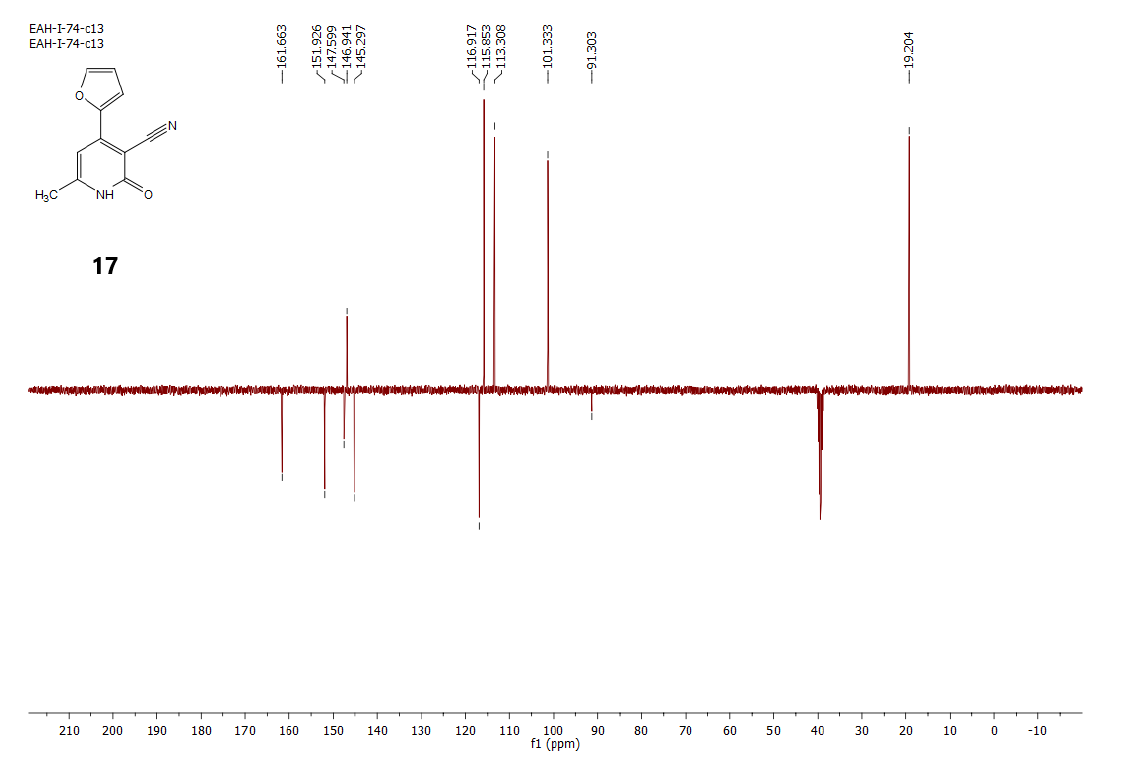


**Fig. S16:** ^13^C-APT NMR spectrum of compound 17.


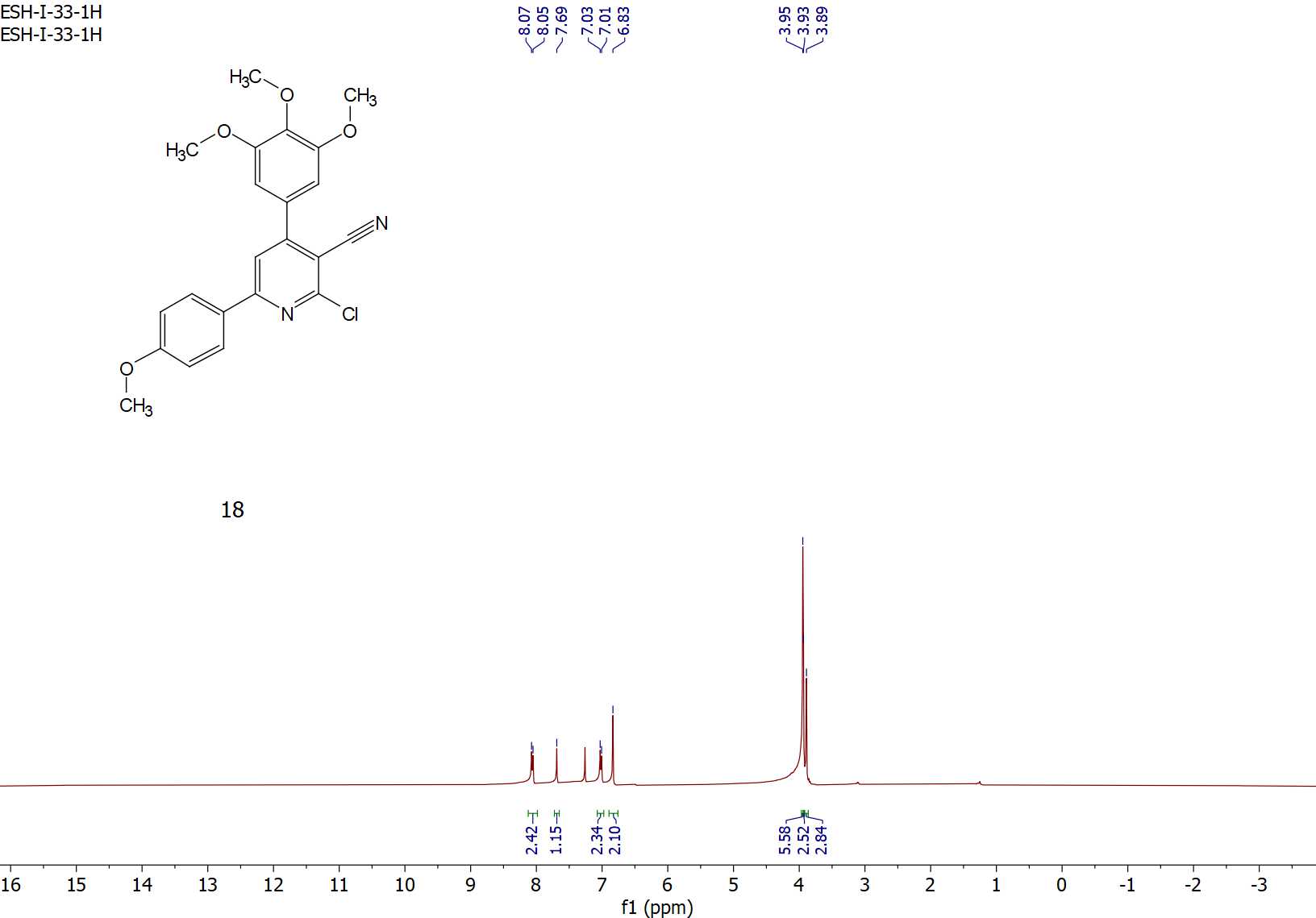


**Fig. S17:** ^1^H-NMR spectrum of compound 18.


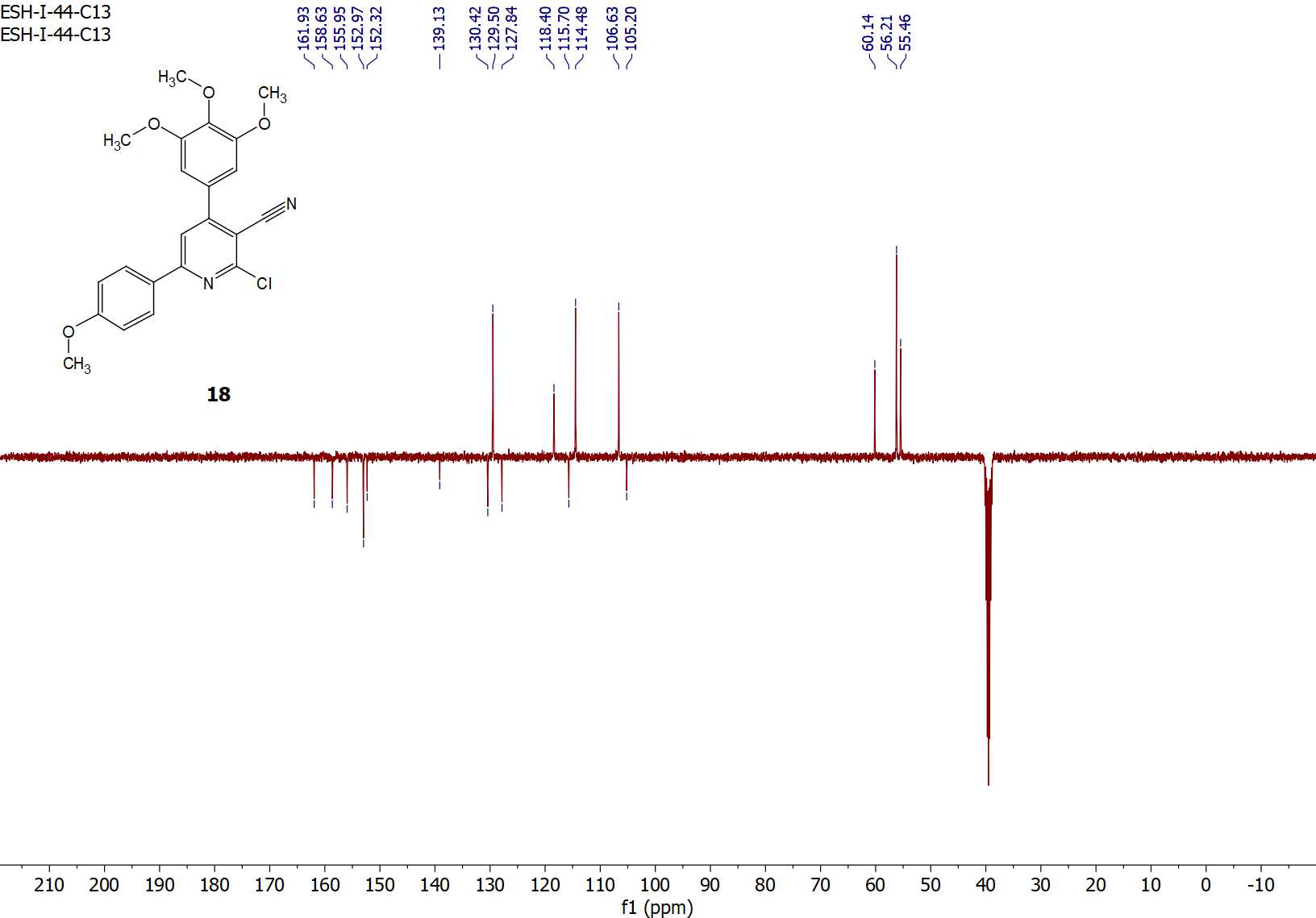


**Fig. S18:** ^13^C-APT NMR spectrum of compound 18.


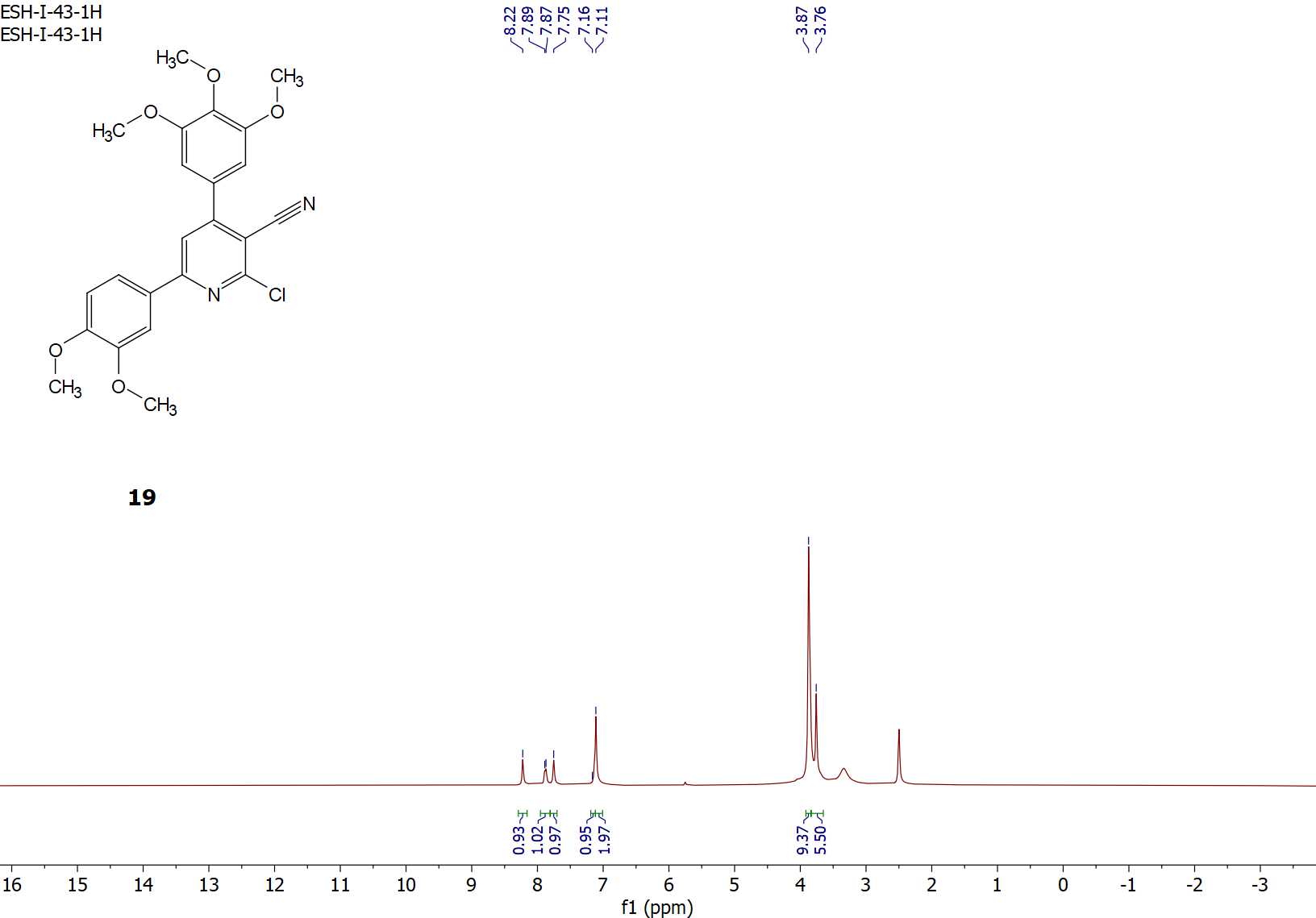


**Fig. S19:** ^1^H-NMR spectrum of compound 19.


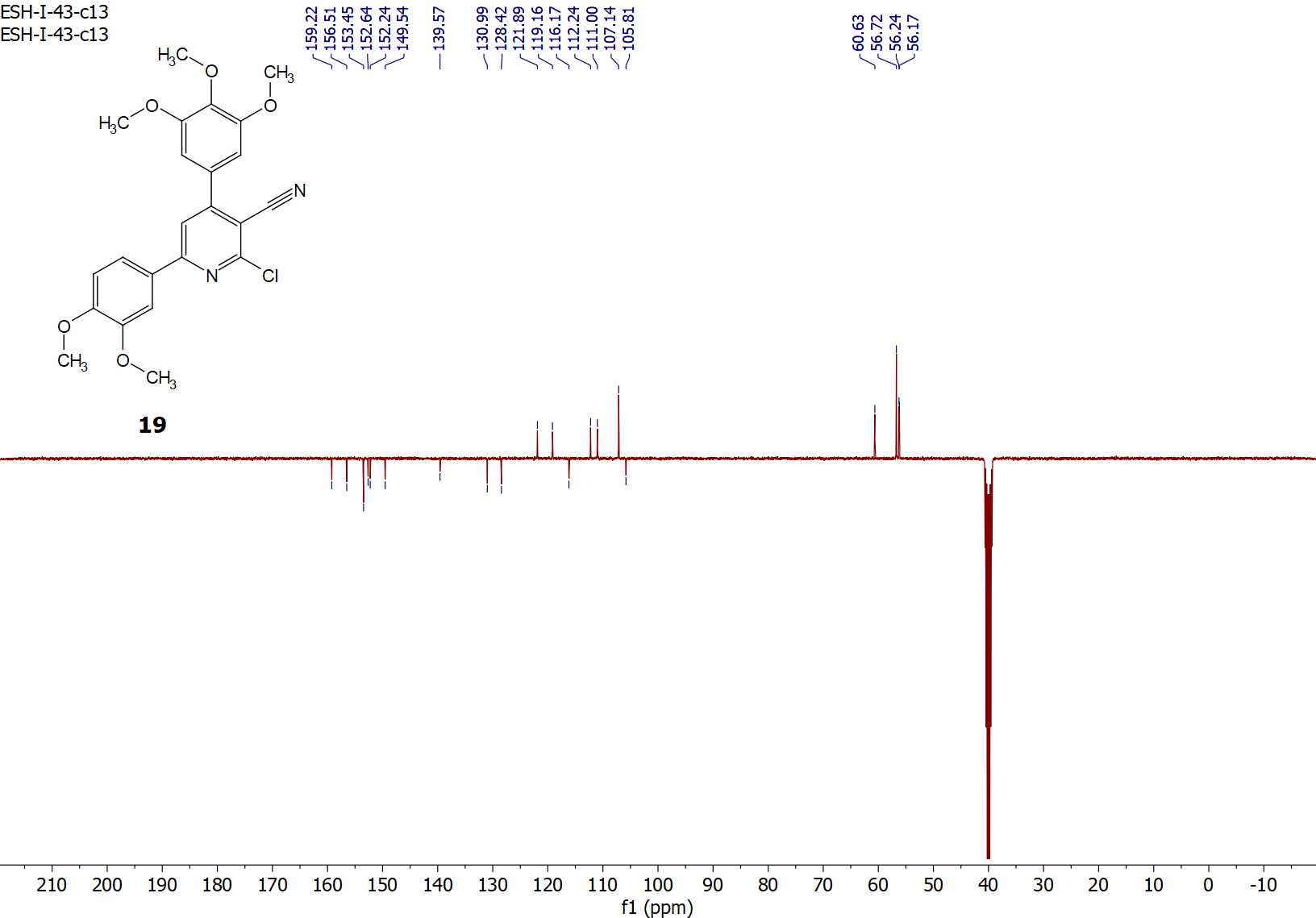


**Fig. S20:** ^13^C-APT NMR spectrum of compound 19.


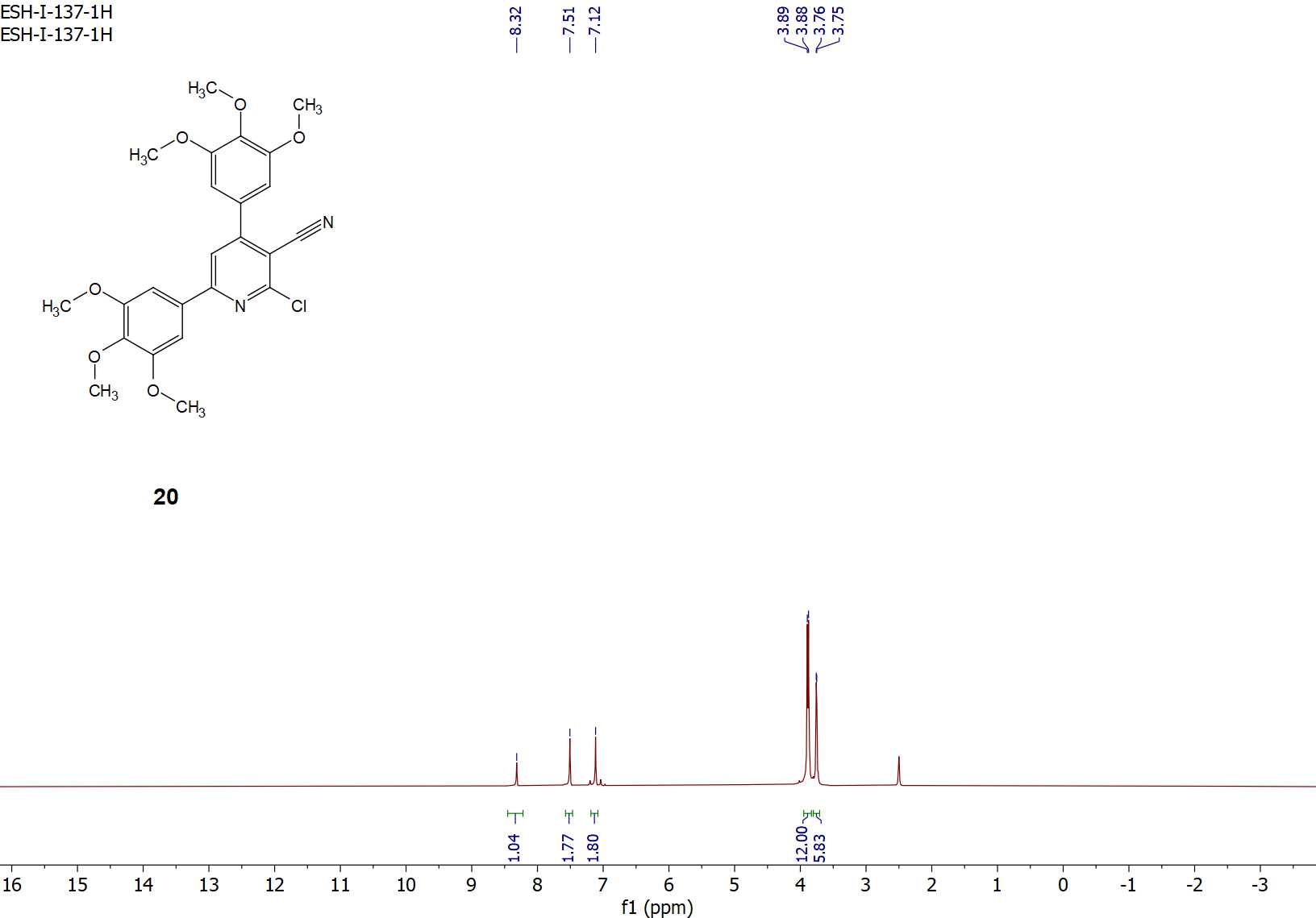


**Fig. S21:** ^1^H-NMR spectrum of compound 20.


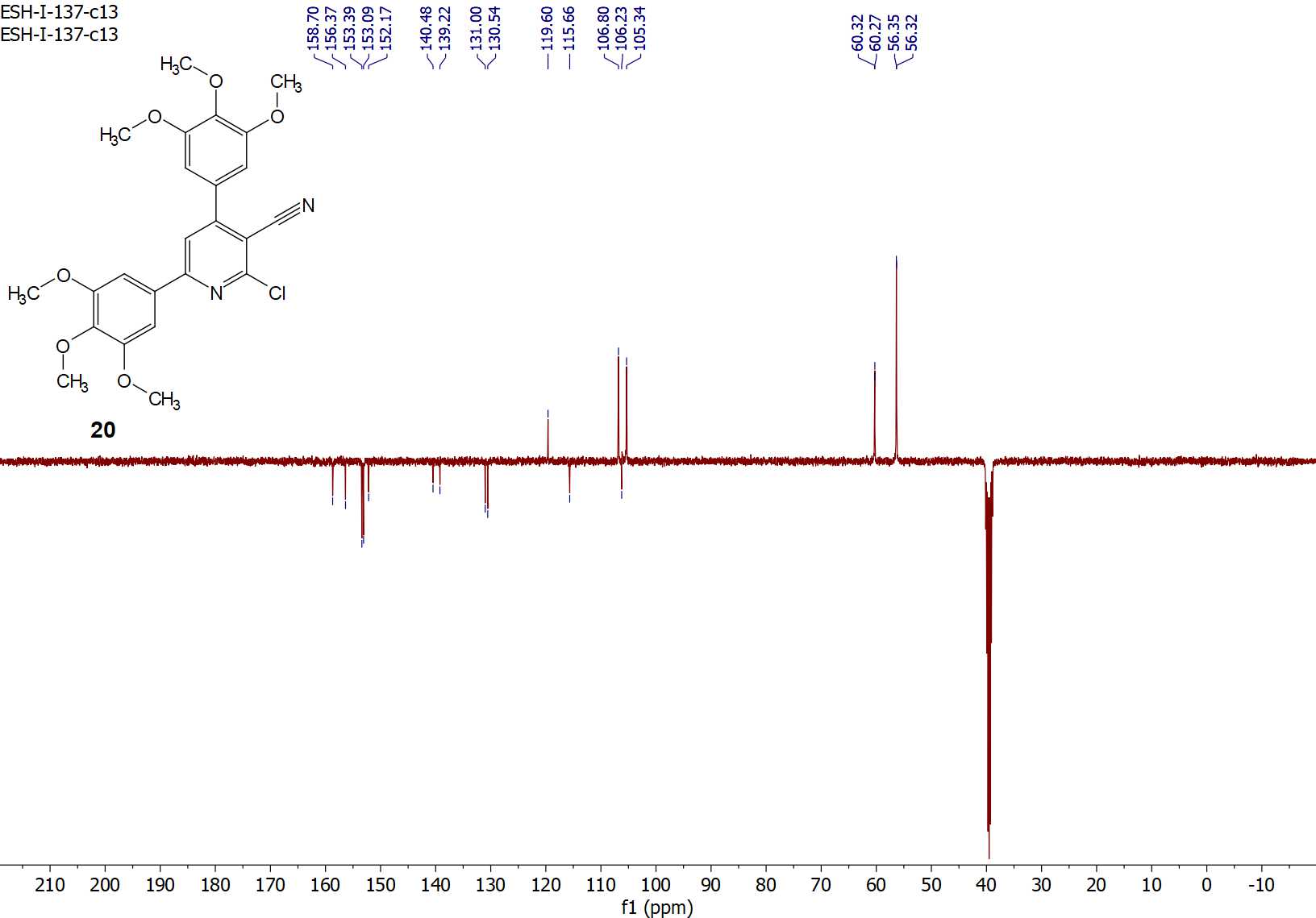


**Fig. S22:** ^13^C-APT NMR spectrum of compound 20.


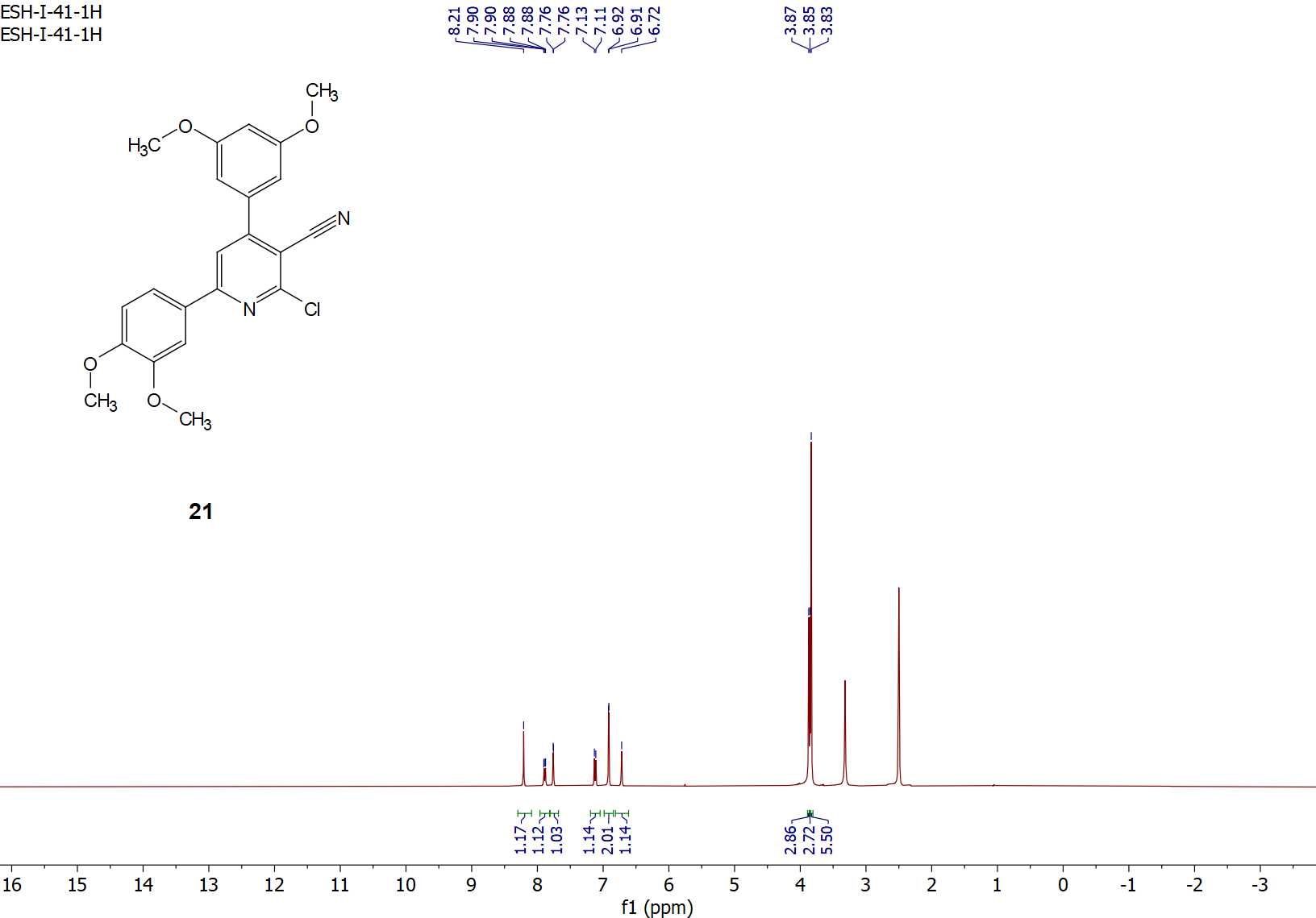


**Fig. S23:** ^1^H-NMR spectrum of compound 21.


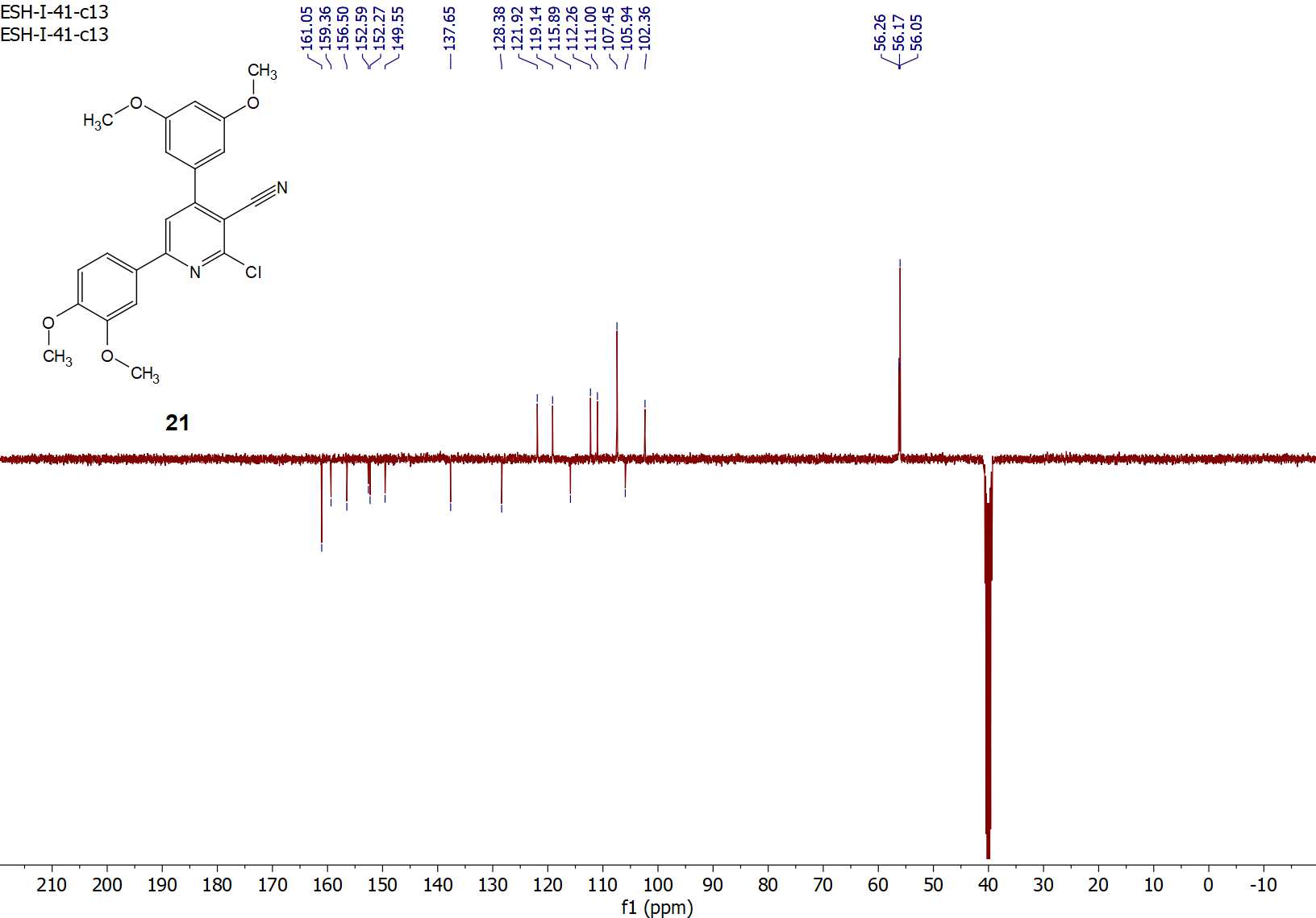


**Fig. S24:** ^13^C-APT NMR spectrum of compound 21.


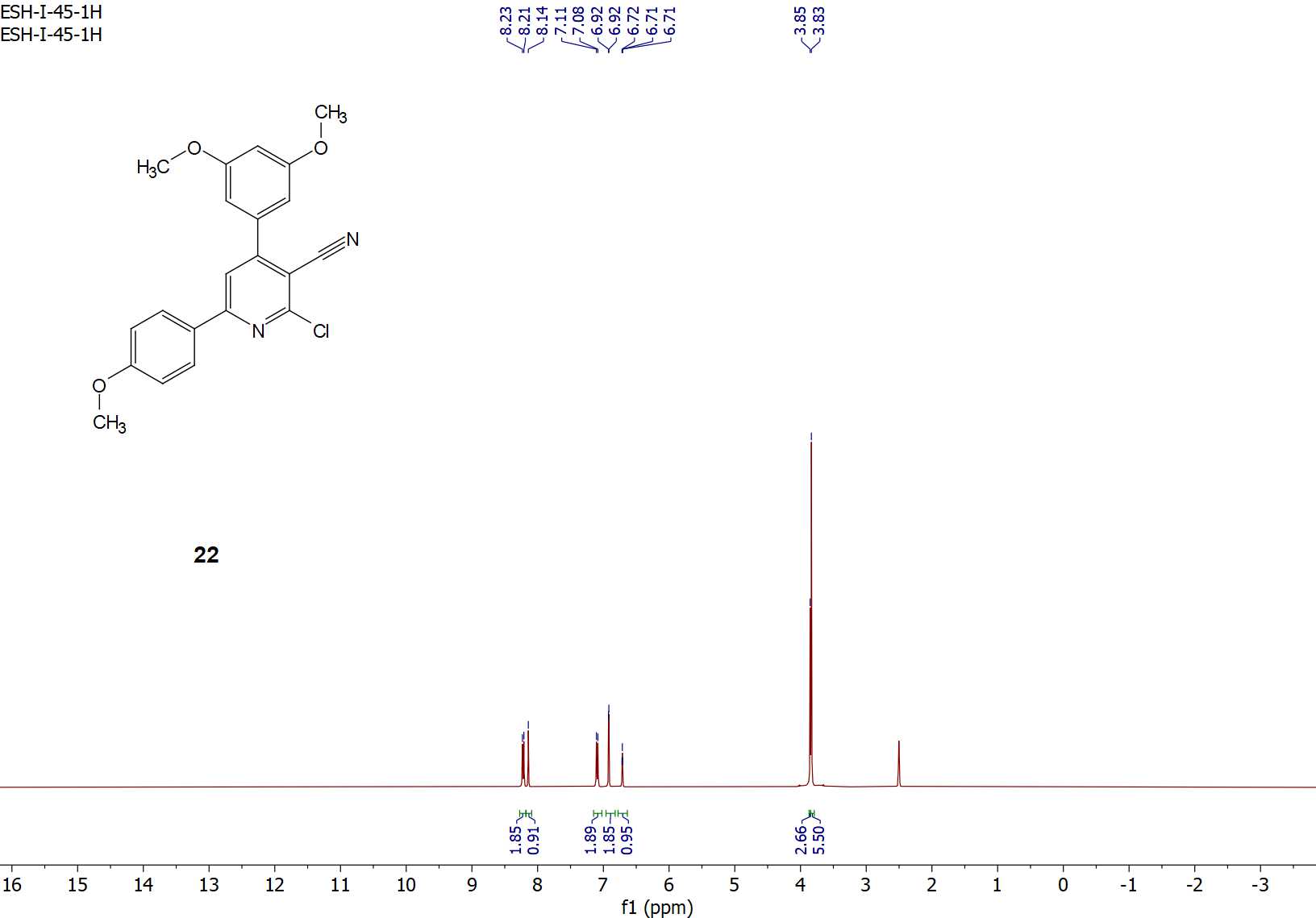


**Fig. S25:** ^1^H-NMR spectrum of compound 22.


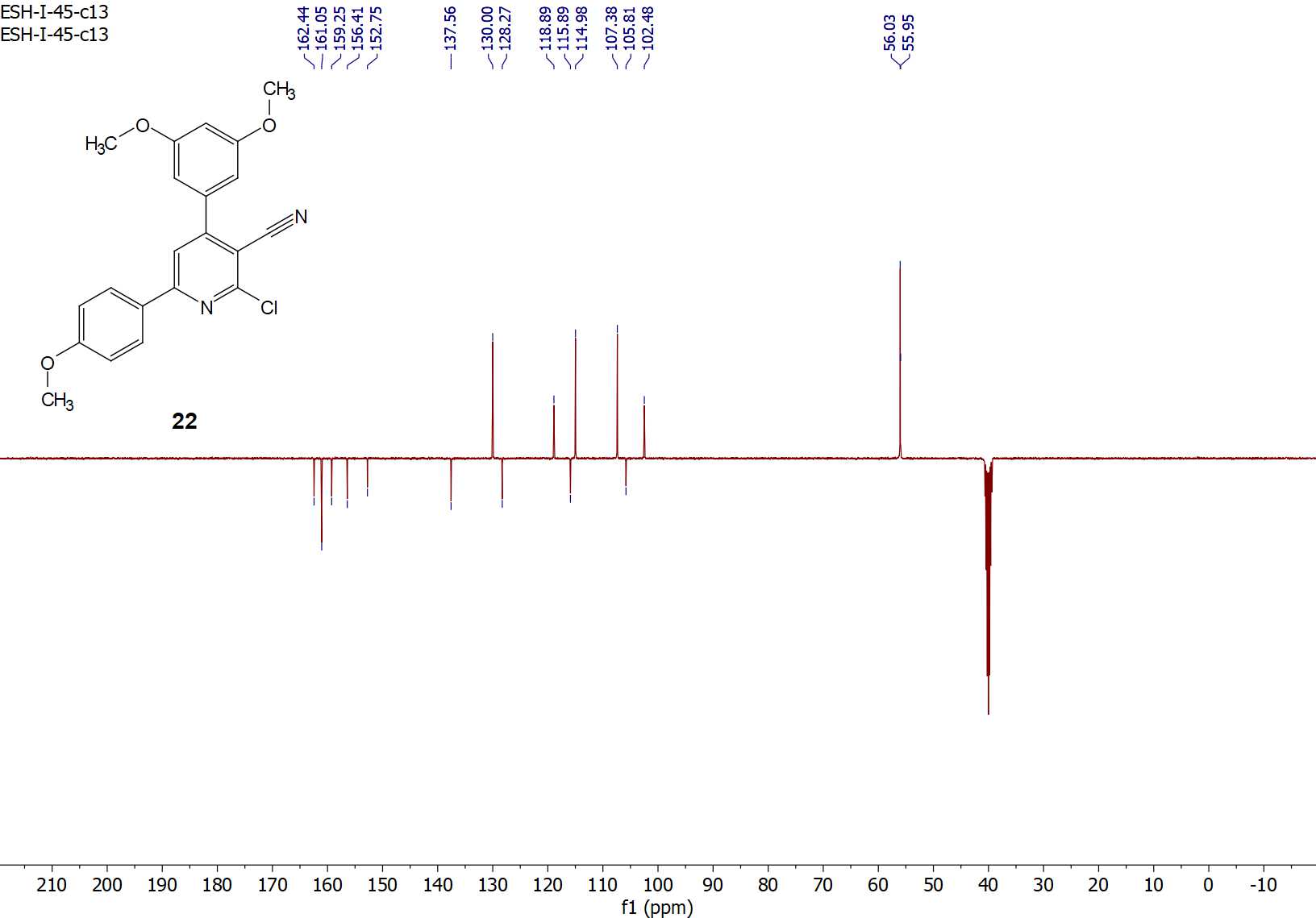


**Fig. S26:** ^13^C-APT NMR spectrum of compound 22.


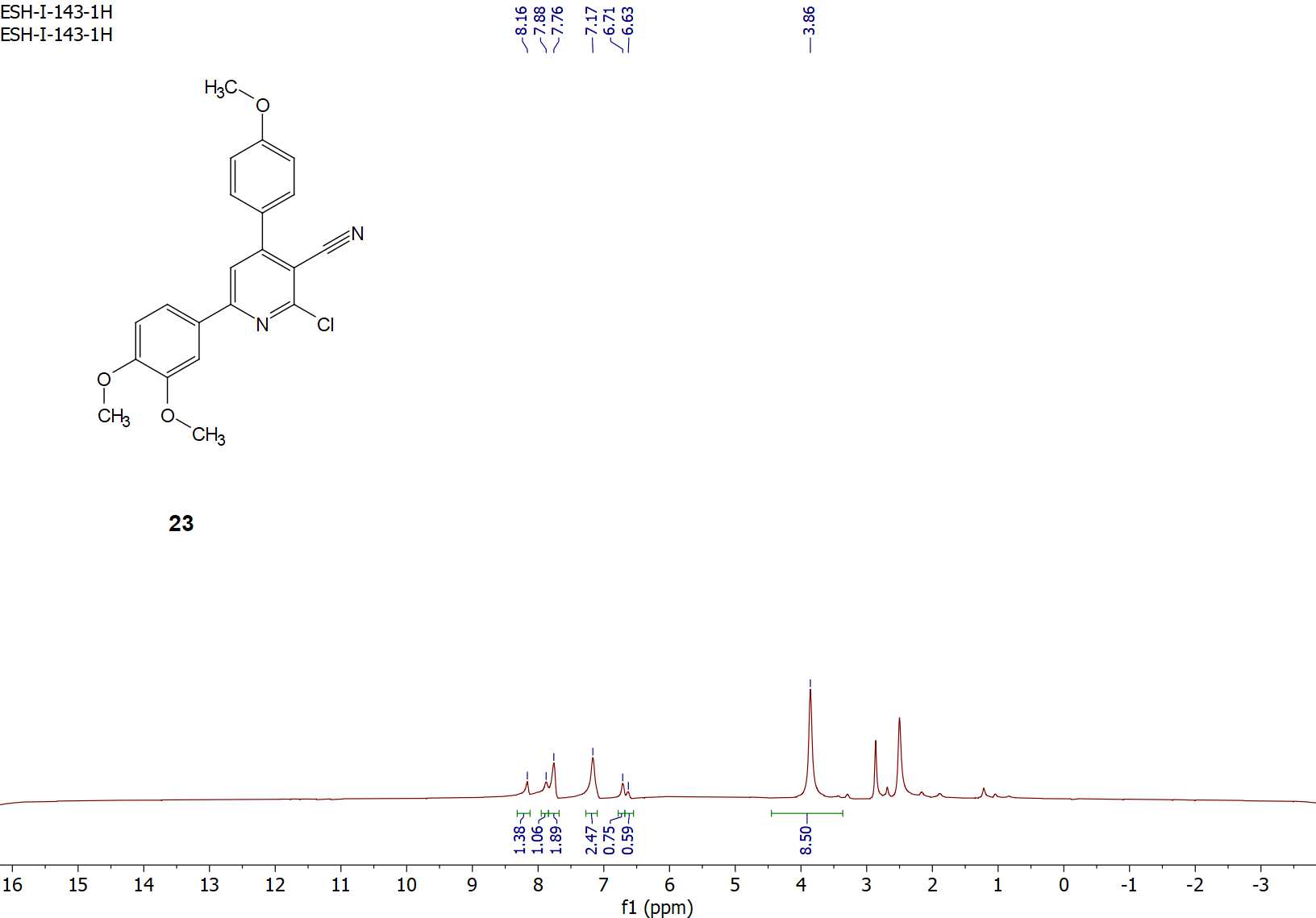


**Fig. S27:** ^1^H-NMR spectrum of compound 23.


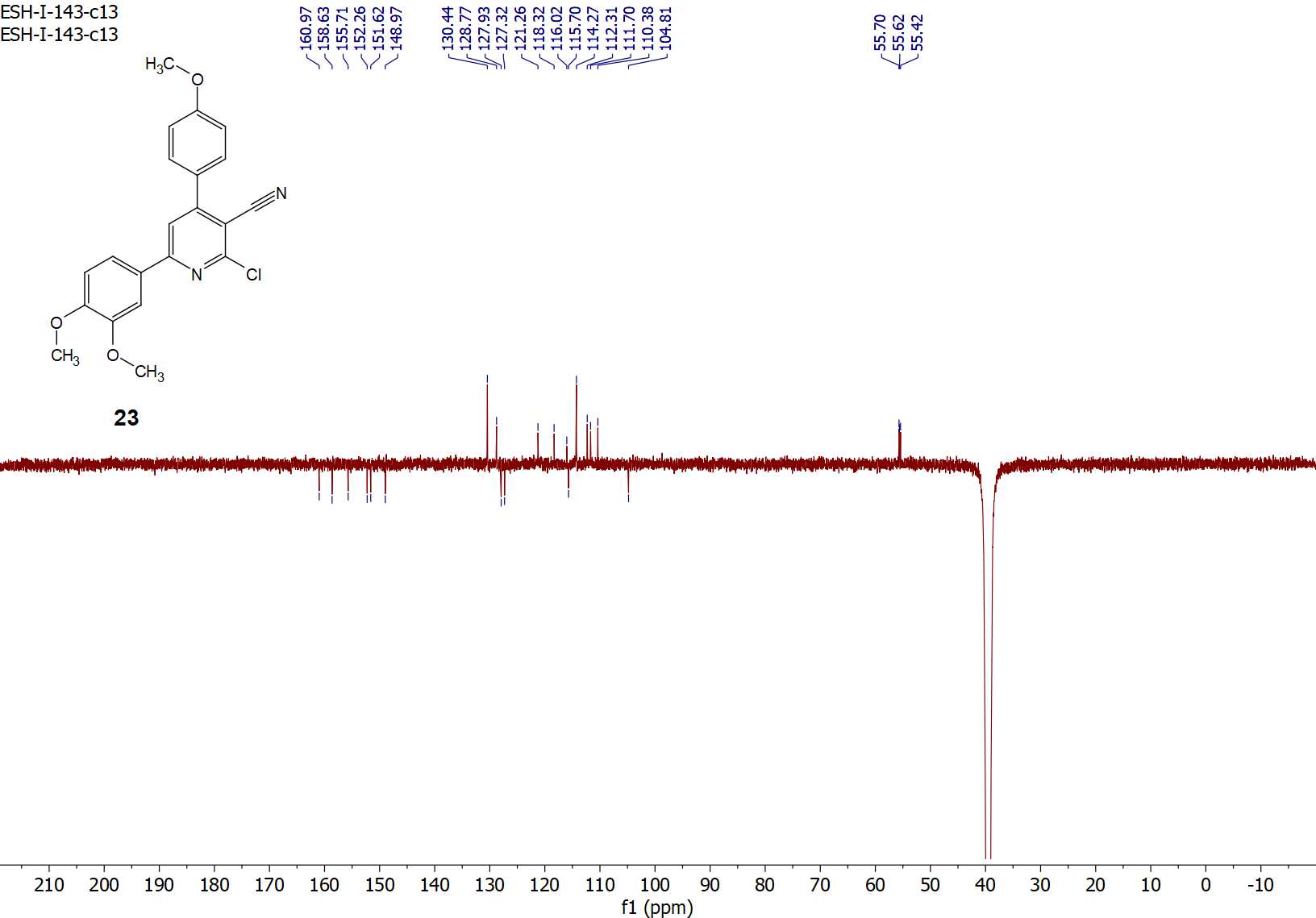


**Fig. S28:** ^13^C-APT NMR spectrum of compound 23.


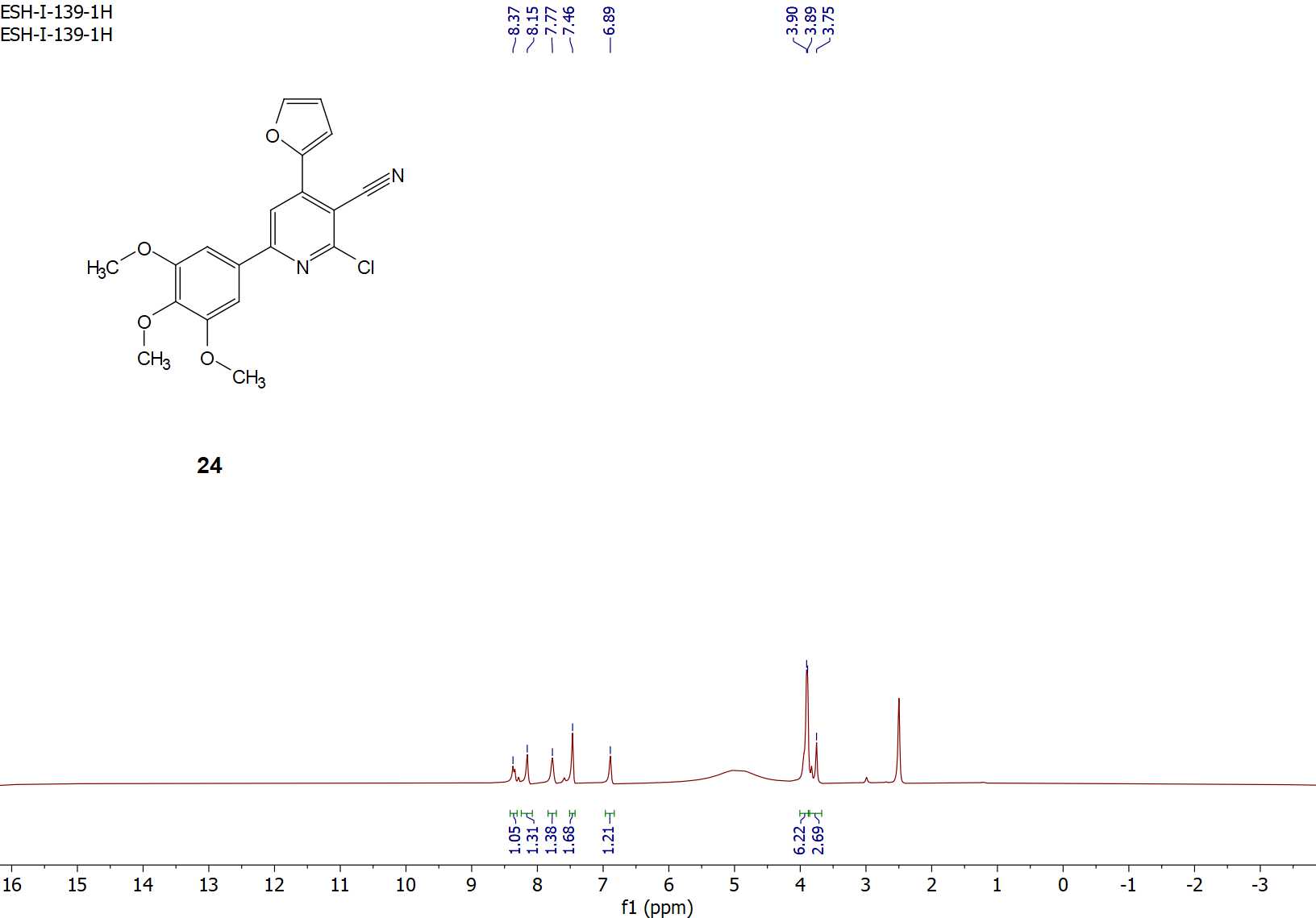


**Fig. S29:** ^1^H-NMR spectrum of compound 24.


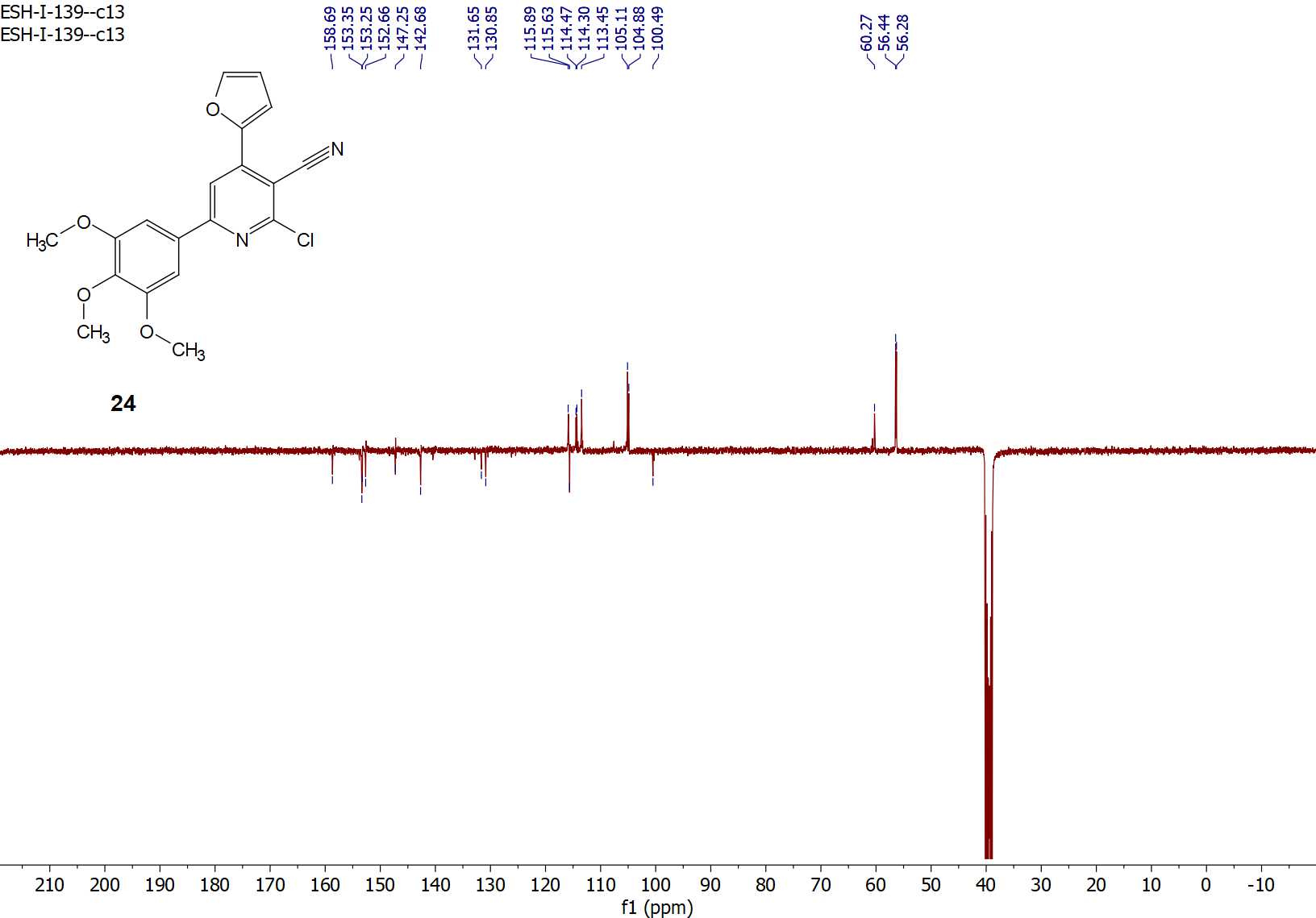


**Fig. S30:** ^13^C-APT NMR spectrum of compound 24.


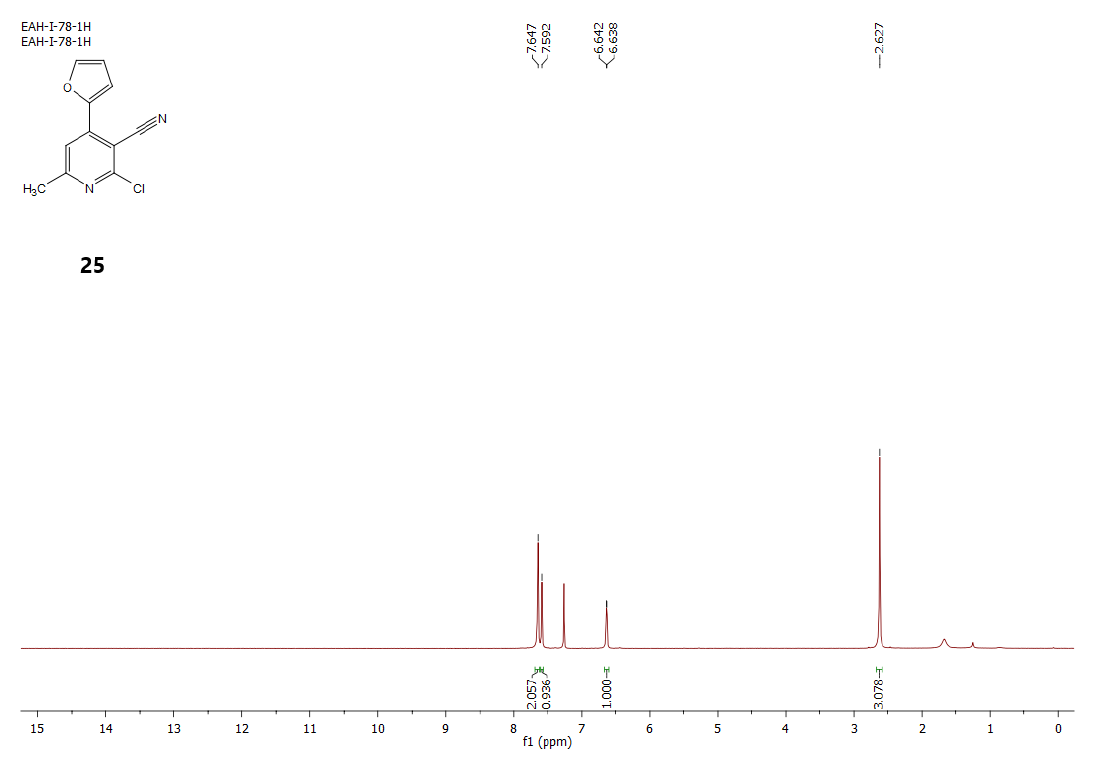


**Fig. S31:** ^1^H-NMR spectrum of compound 25.


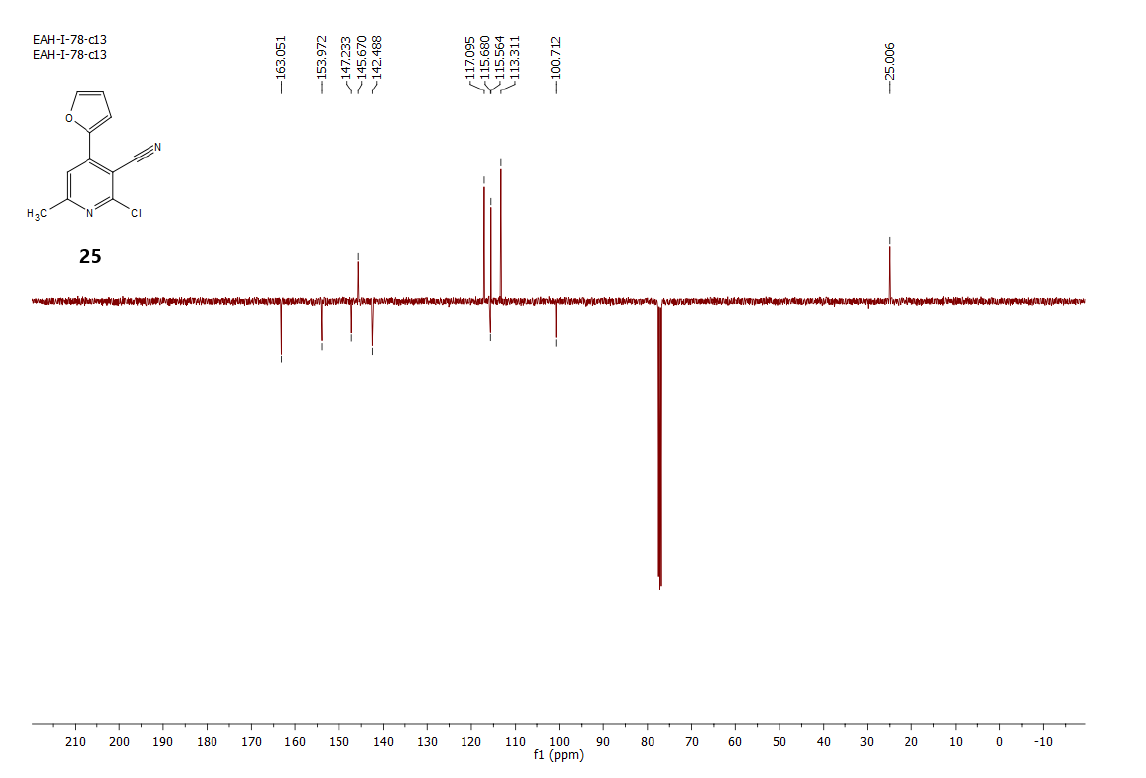


**Fig. S32:** ^13^C-APT NMR spectrum of compound 25.


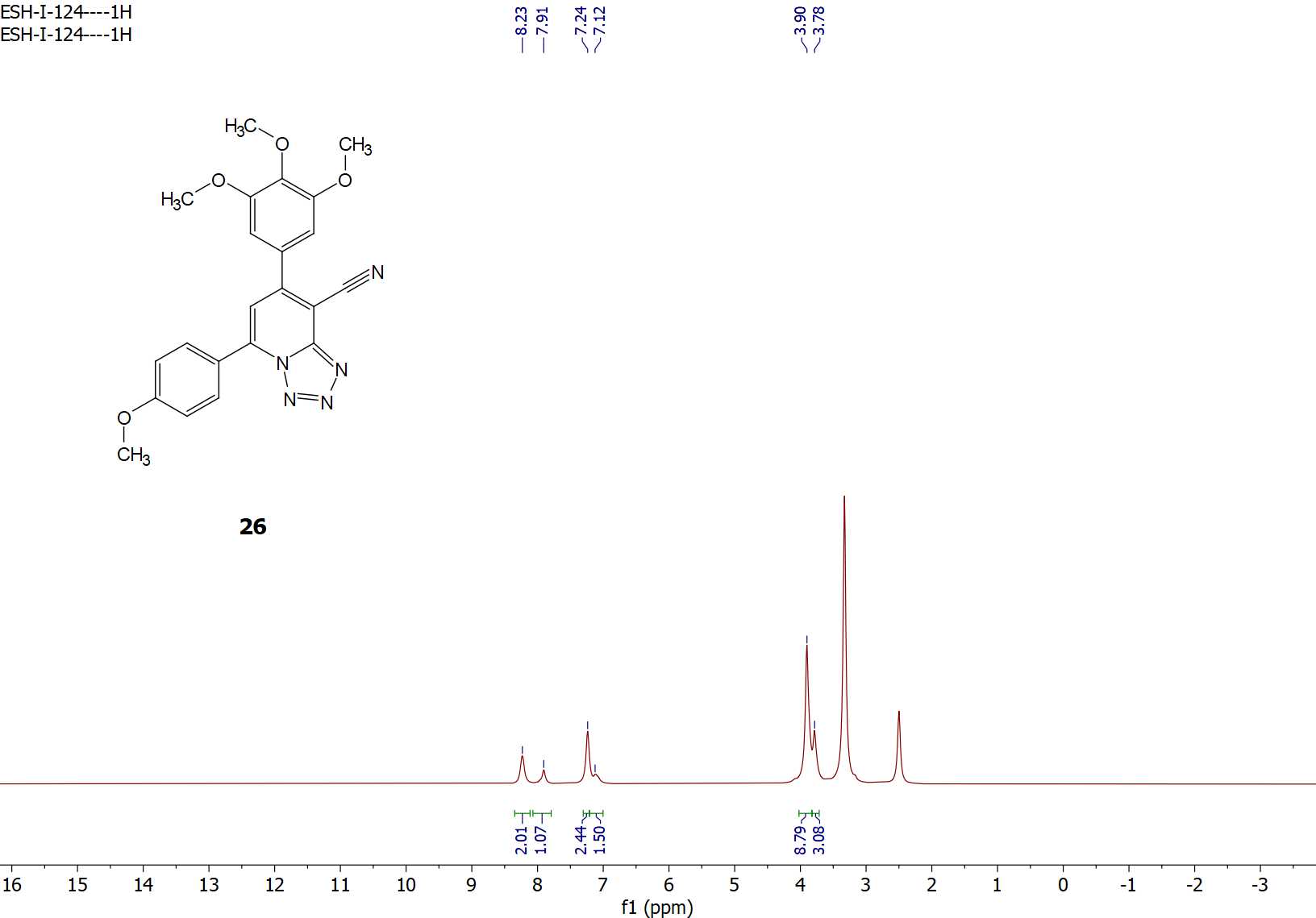


**Fig. S33:** ^1^H-NMR spectrum of compound 26.


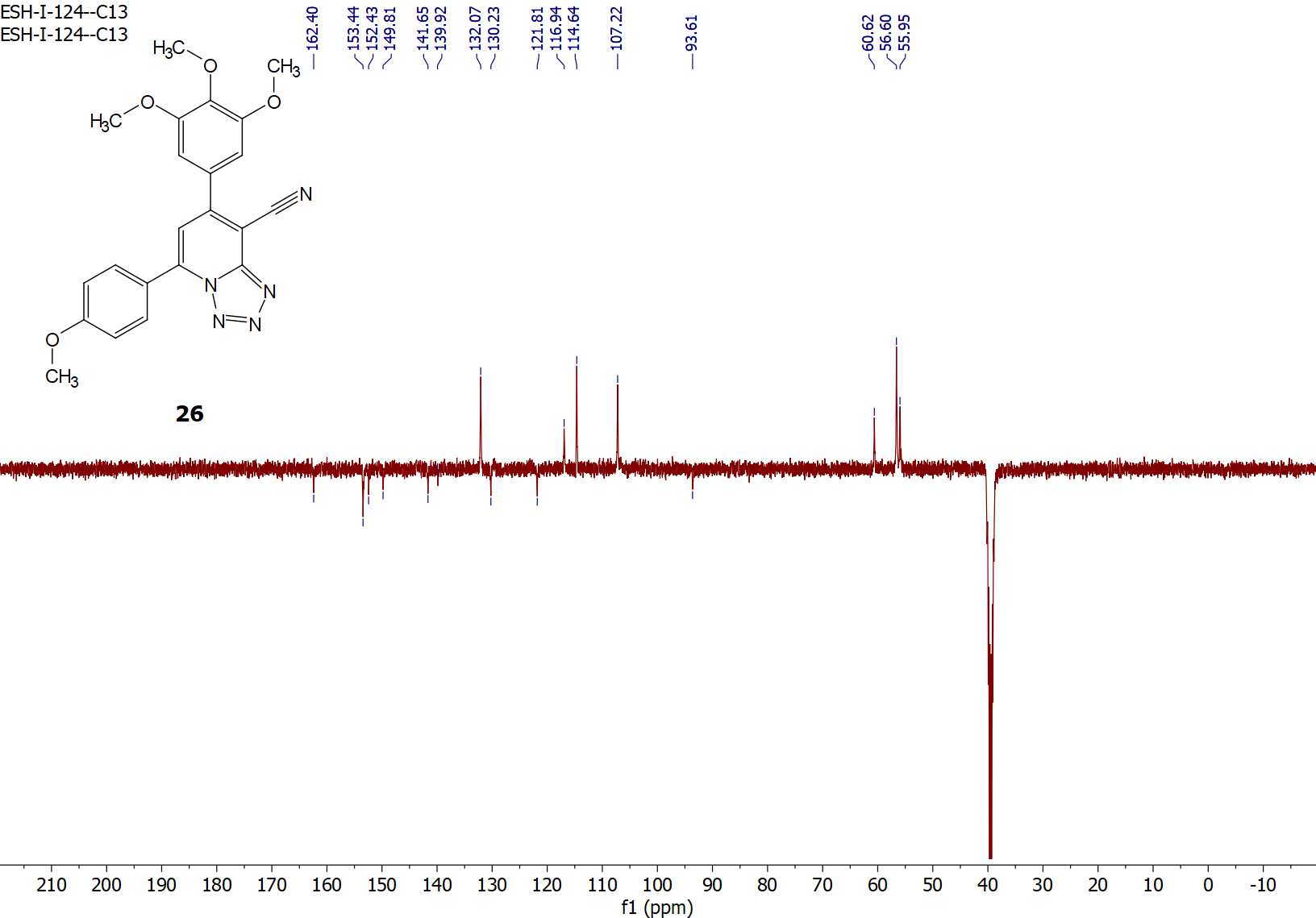


**Fig. S34:** ^13^C-APT NMR spectrum of compound 26.


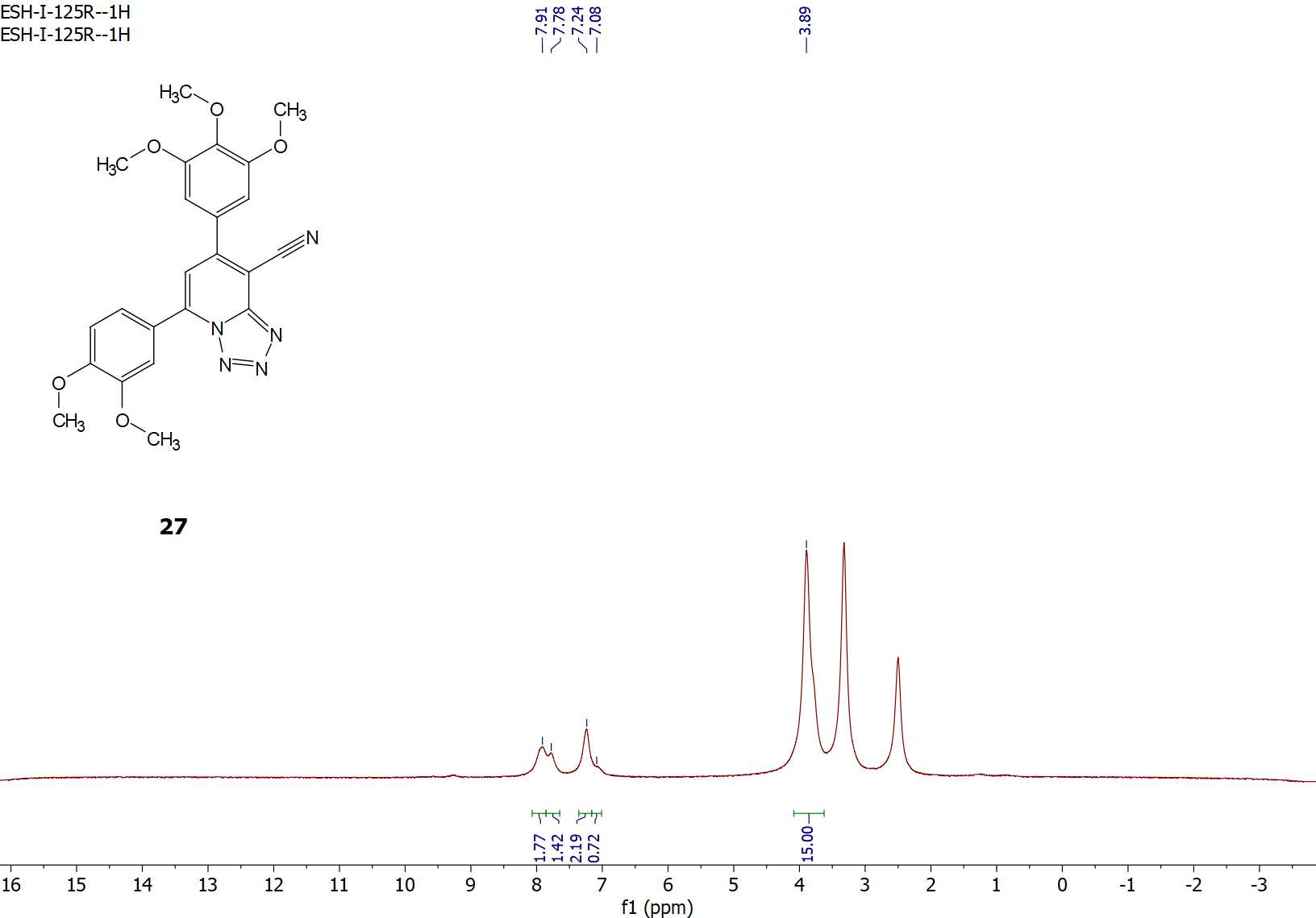


**Fig. S35:** ^1^H-NMR spectrum of compound 27.


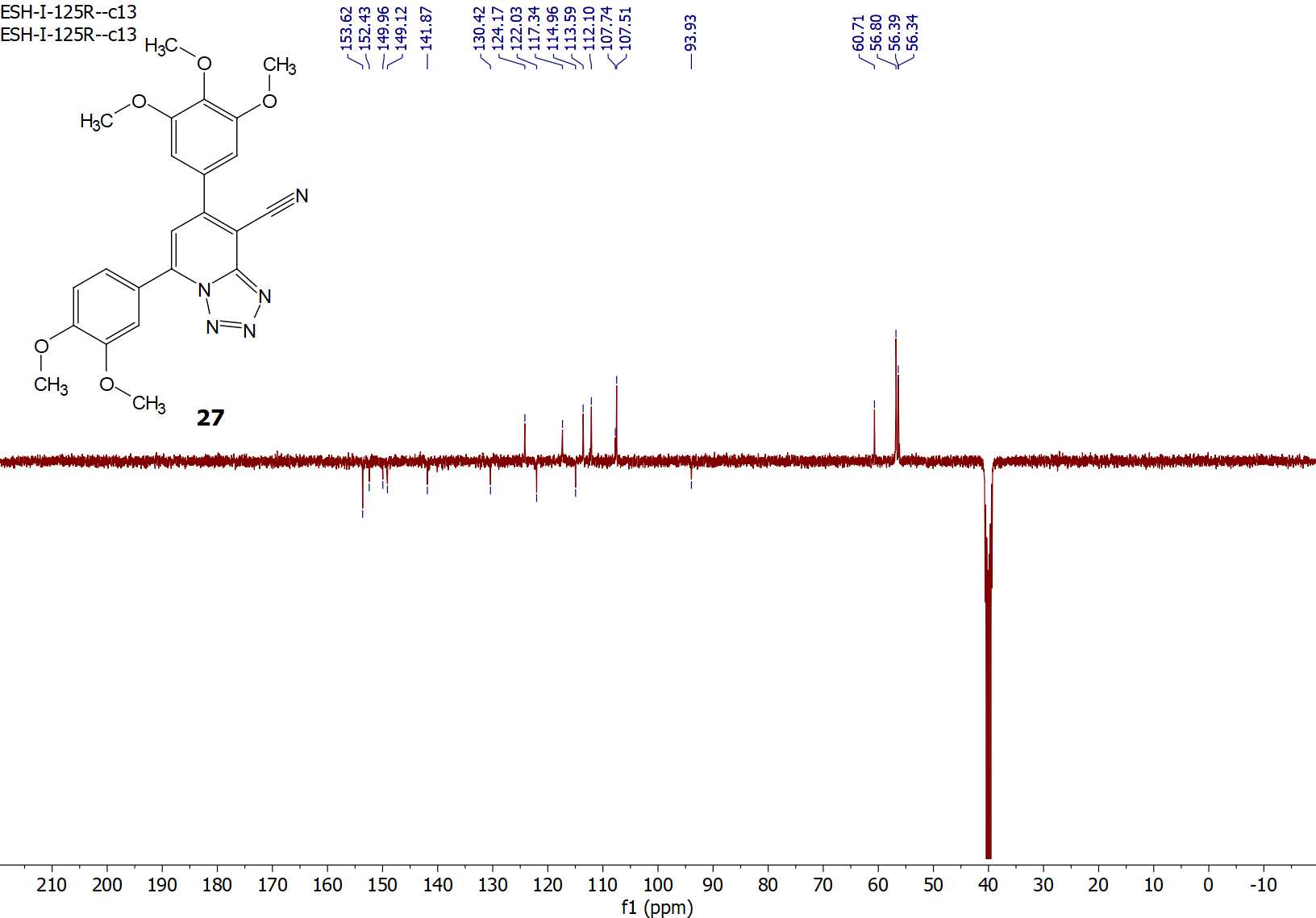


**Fig. S36:** ^13^C-APT NMR spectrum of compound 27.


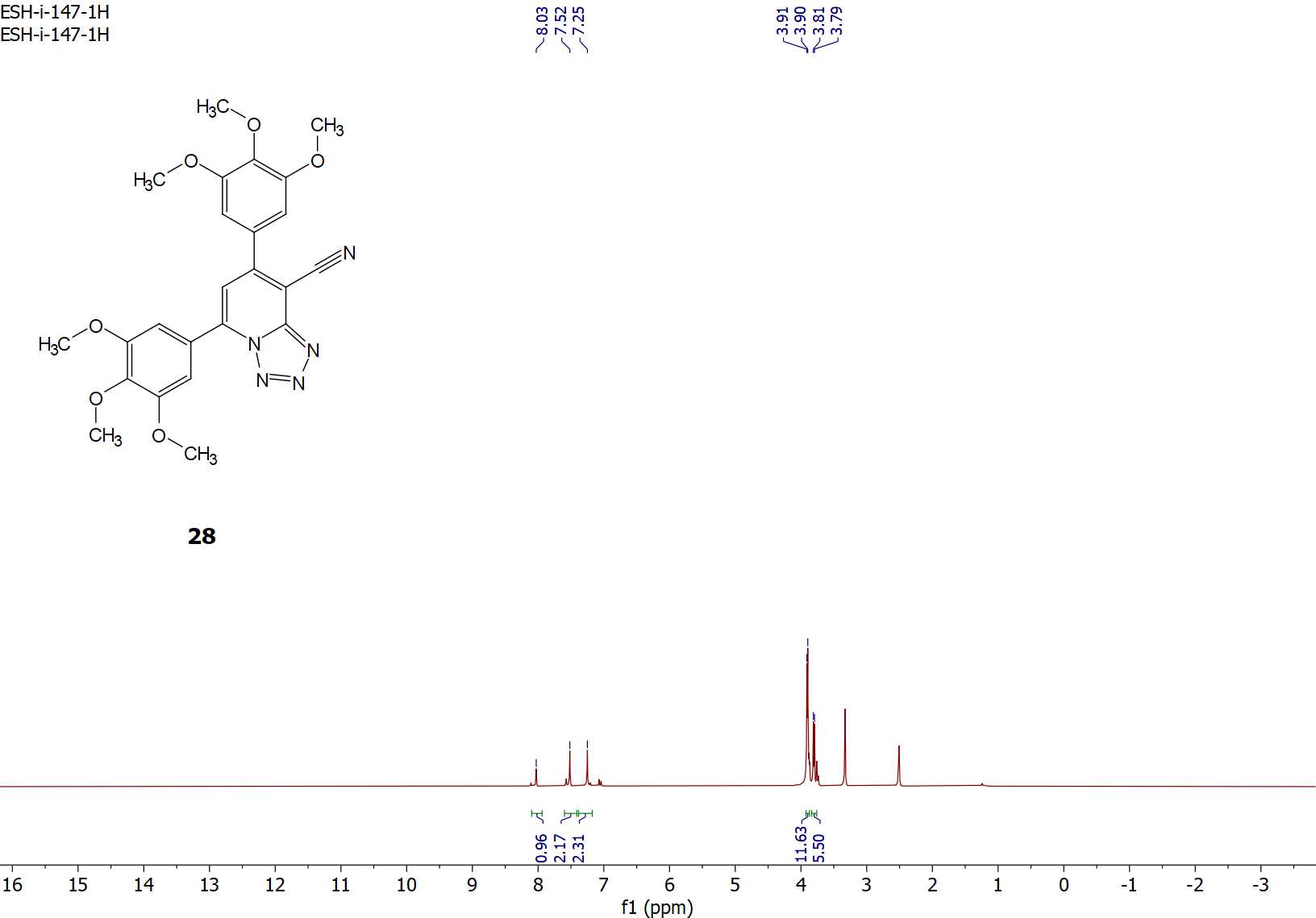


**Fig. S37:** ^1^H-NMR spectrum of compound 28.


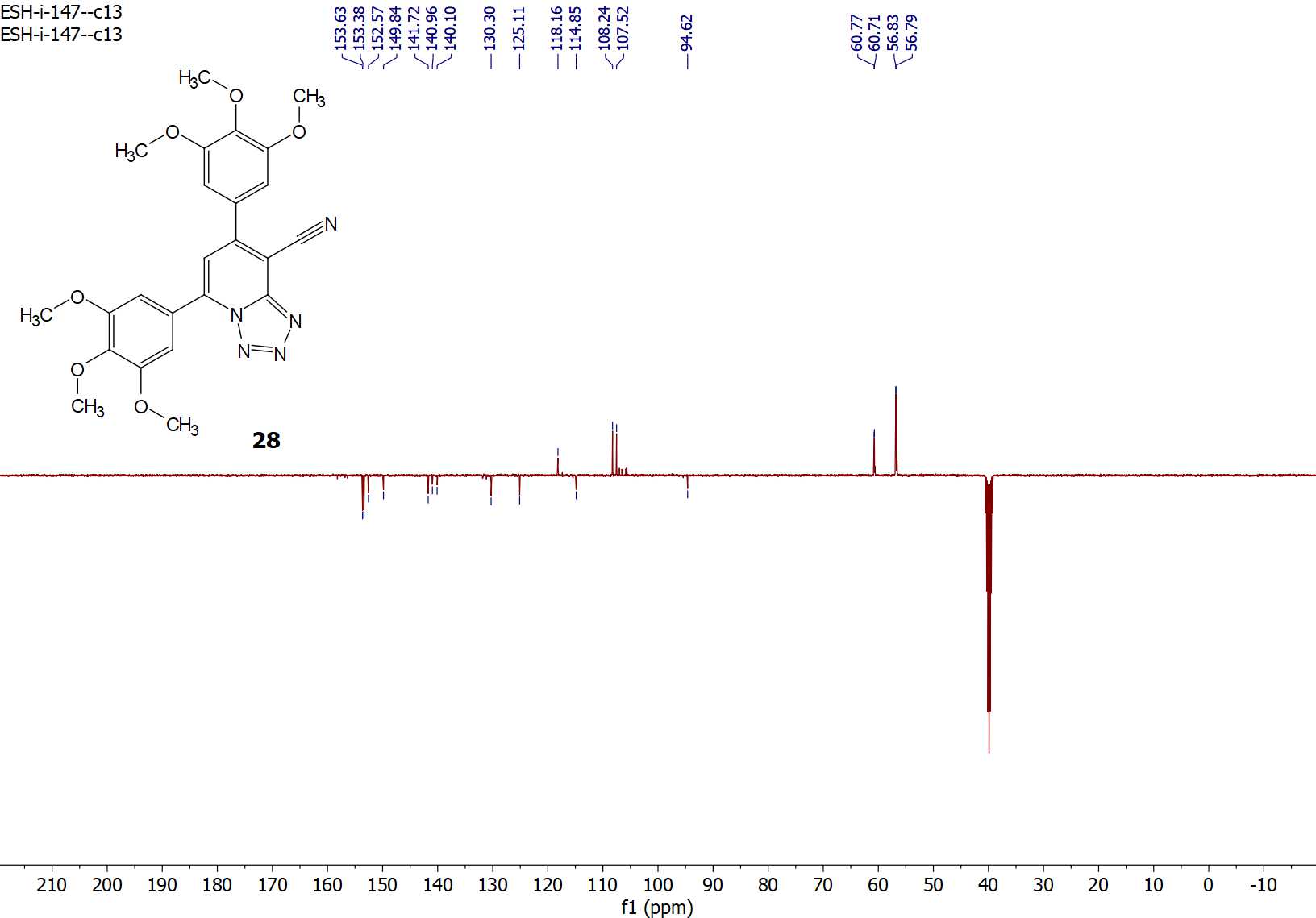


**Fig. S38:** ^13^C-APT NMR spectrum of compound 28.


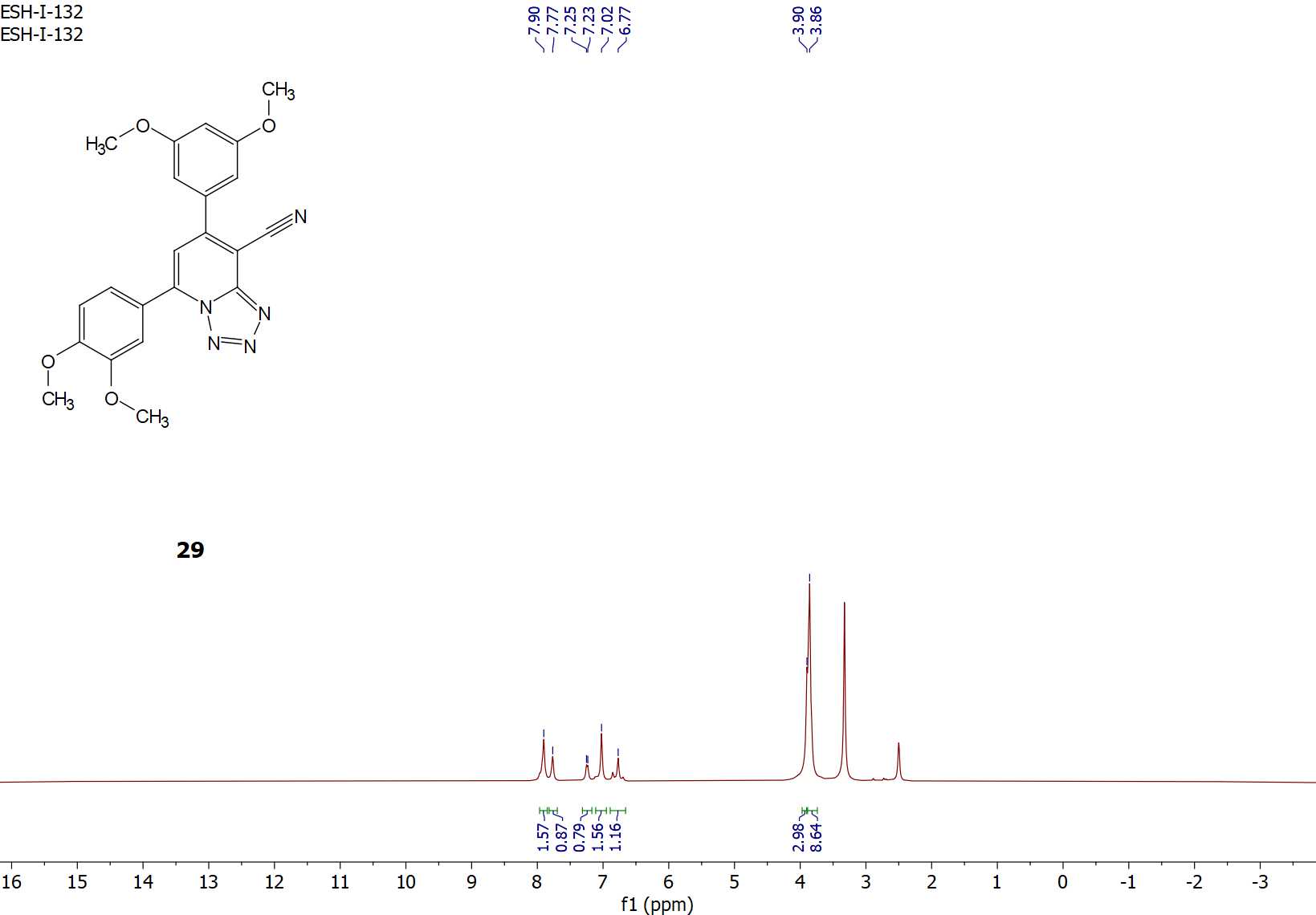


**Fig. S39:** ^1^H-NMR spectrum of compound 29.


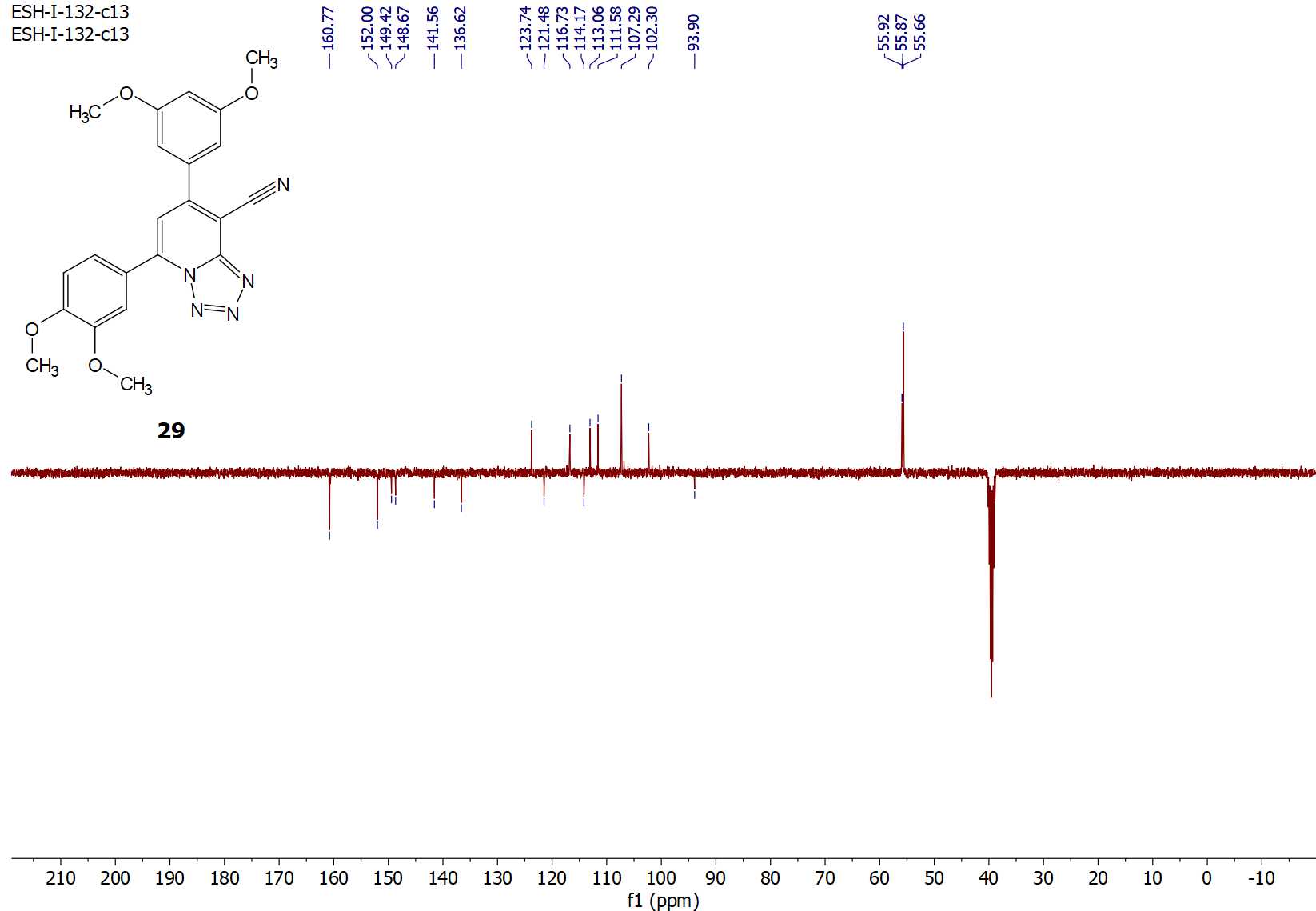


**Fig. S40:** ^13^C-APT NMR spectrum of compound 29.


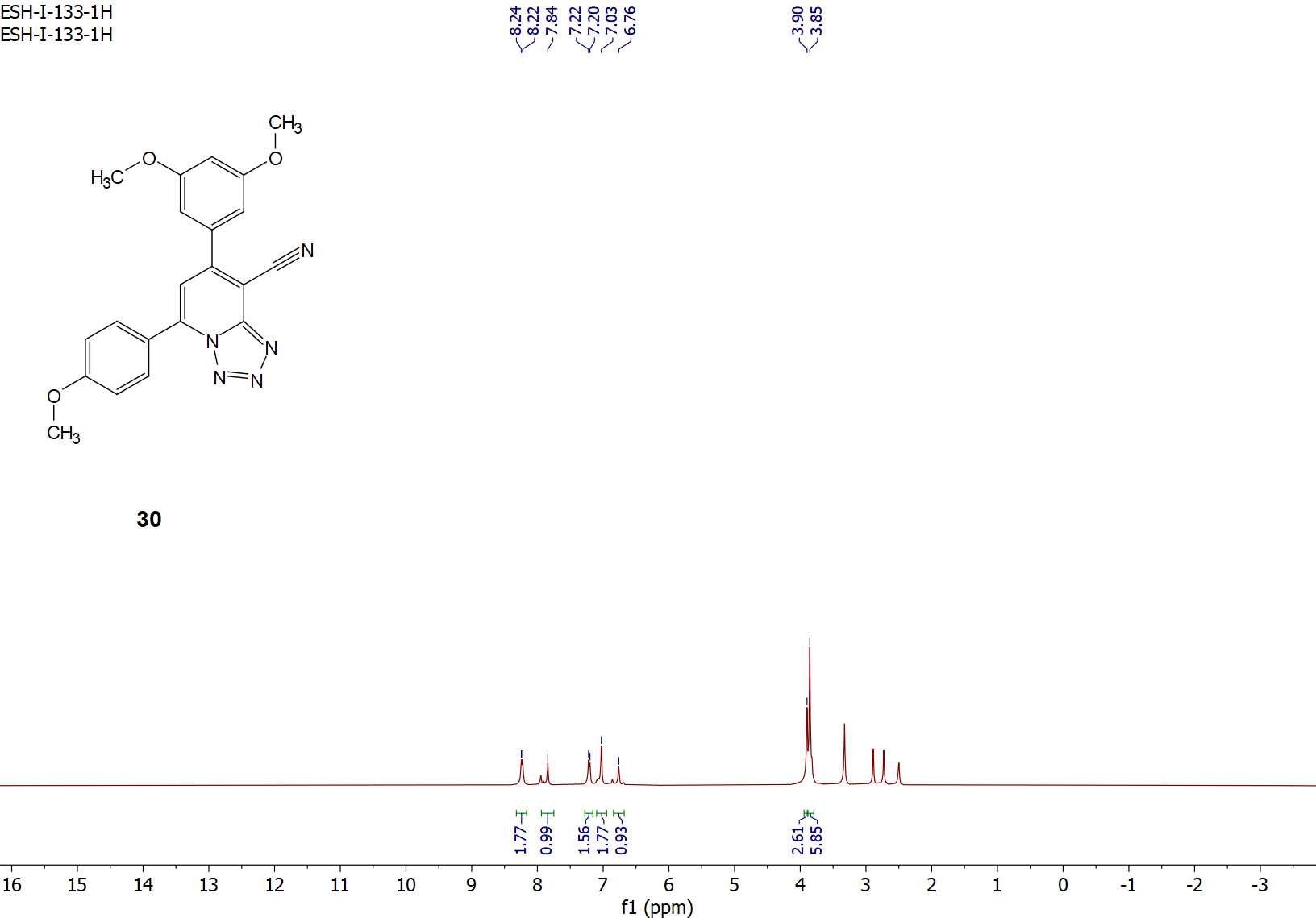


**Fig. S41:** ^1^H-NMR spectrum of compound 30.


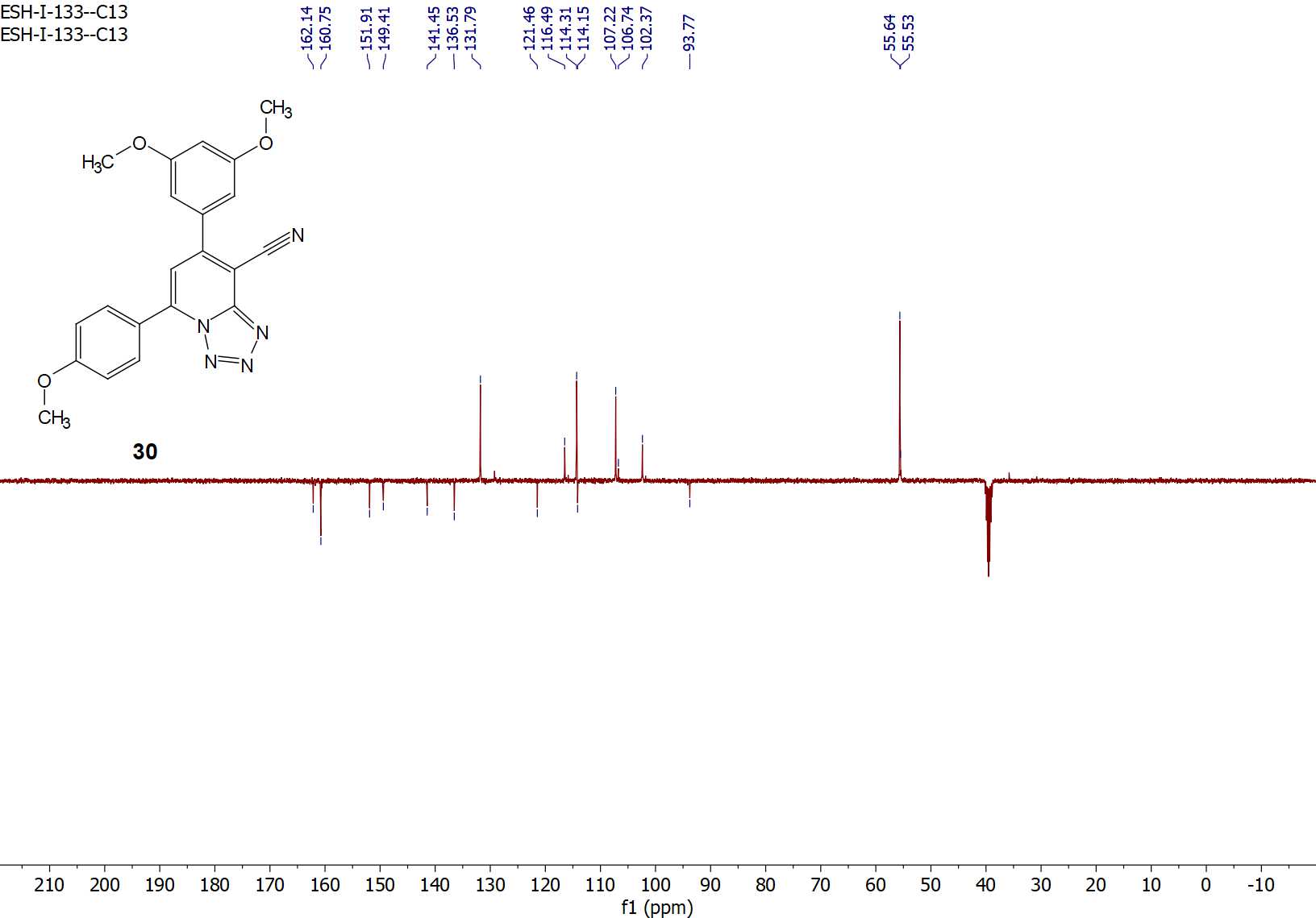


**Fig. S42:** ^13^C-APT NMR spectrum of compound 30.


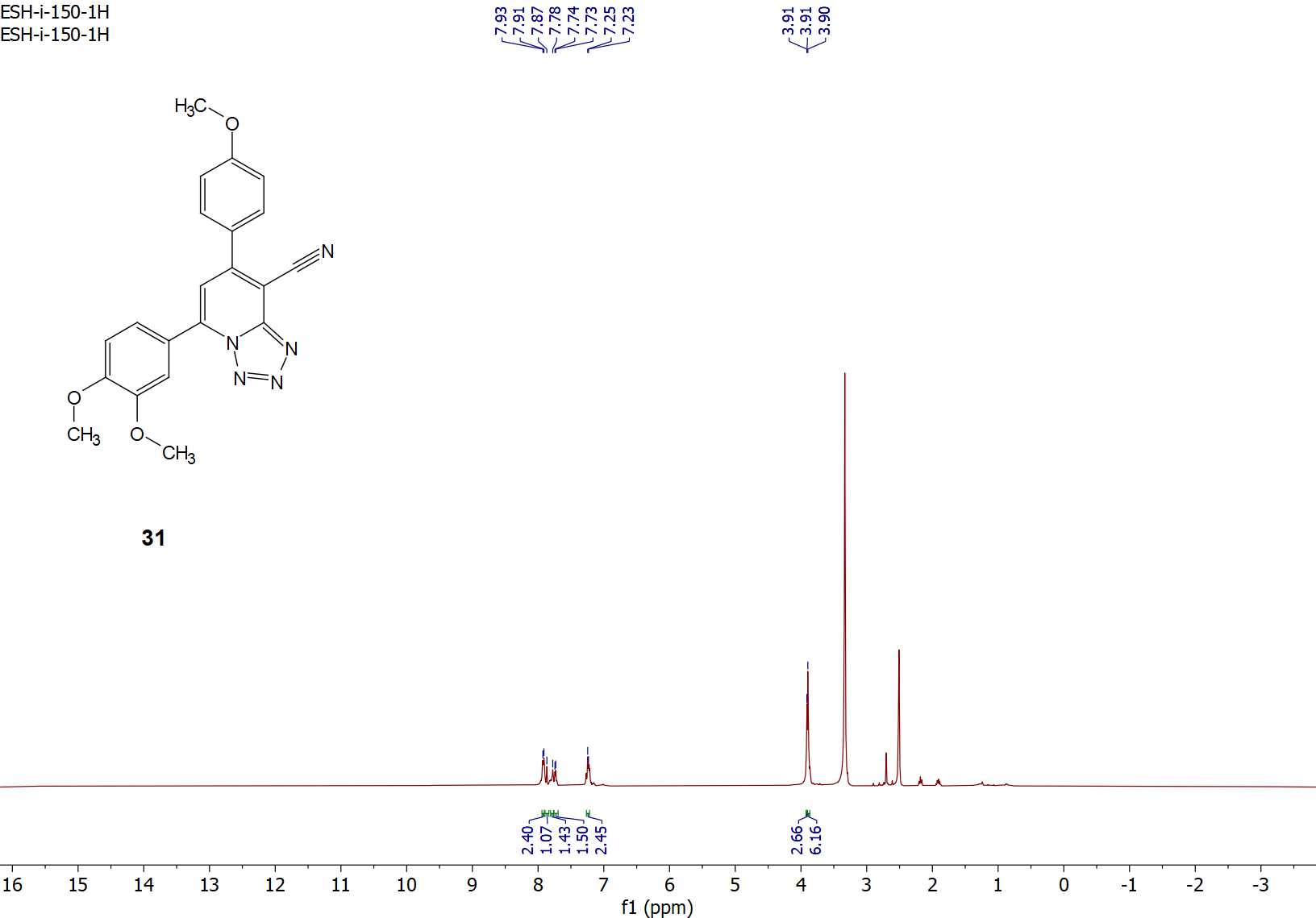


**Fig. S43:** ^1^H-NMR spectrum of compound 31.


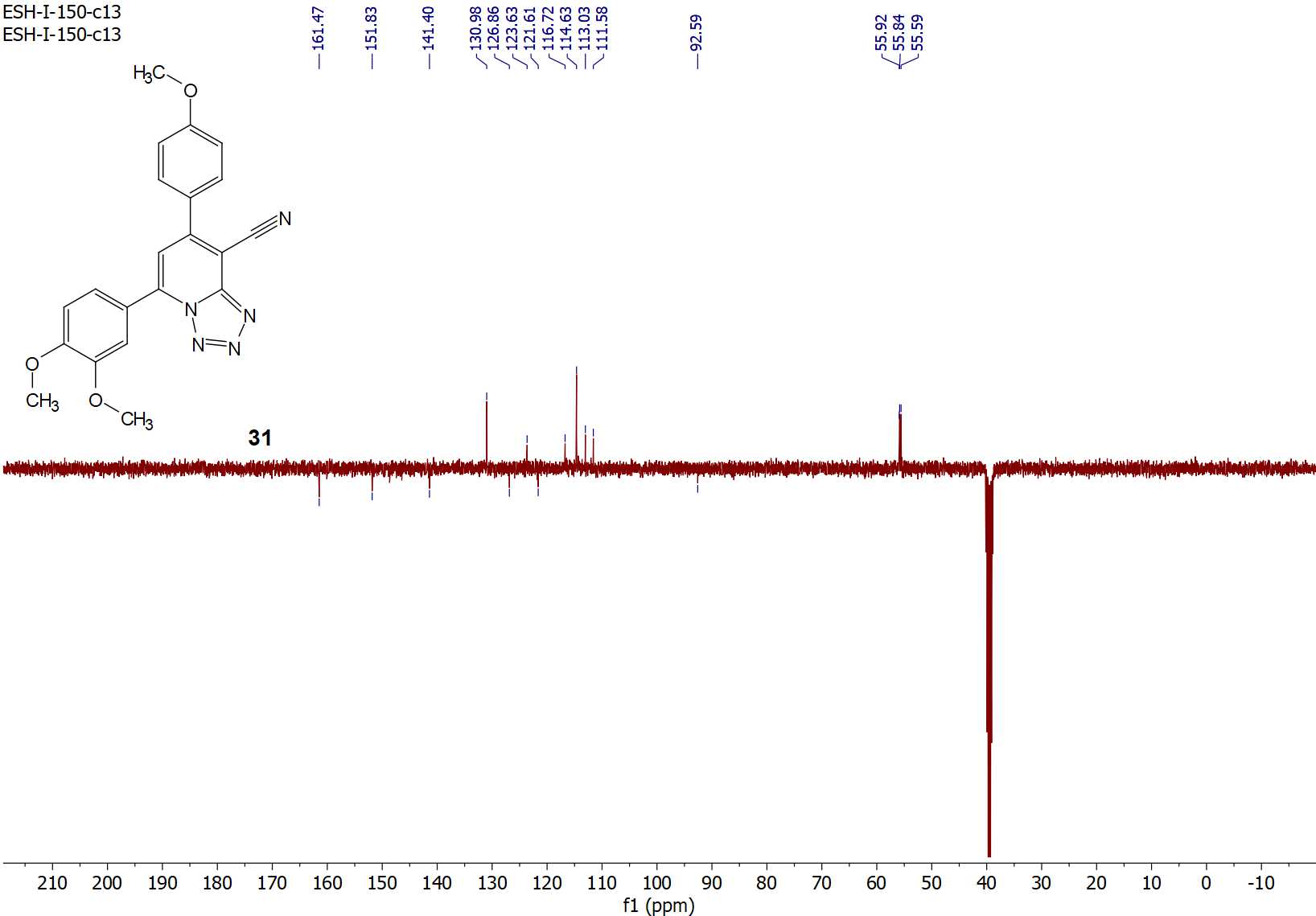


**Fig. S44:** ^13^C-APT NMR spectrum of compound 31.


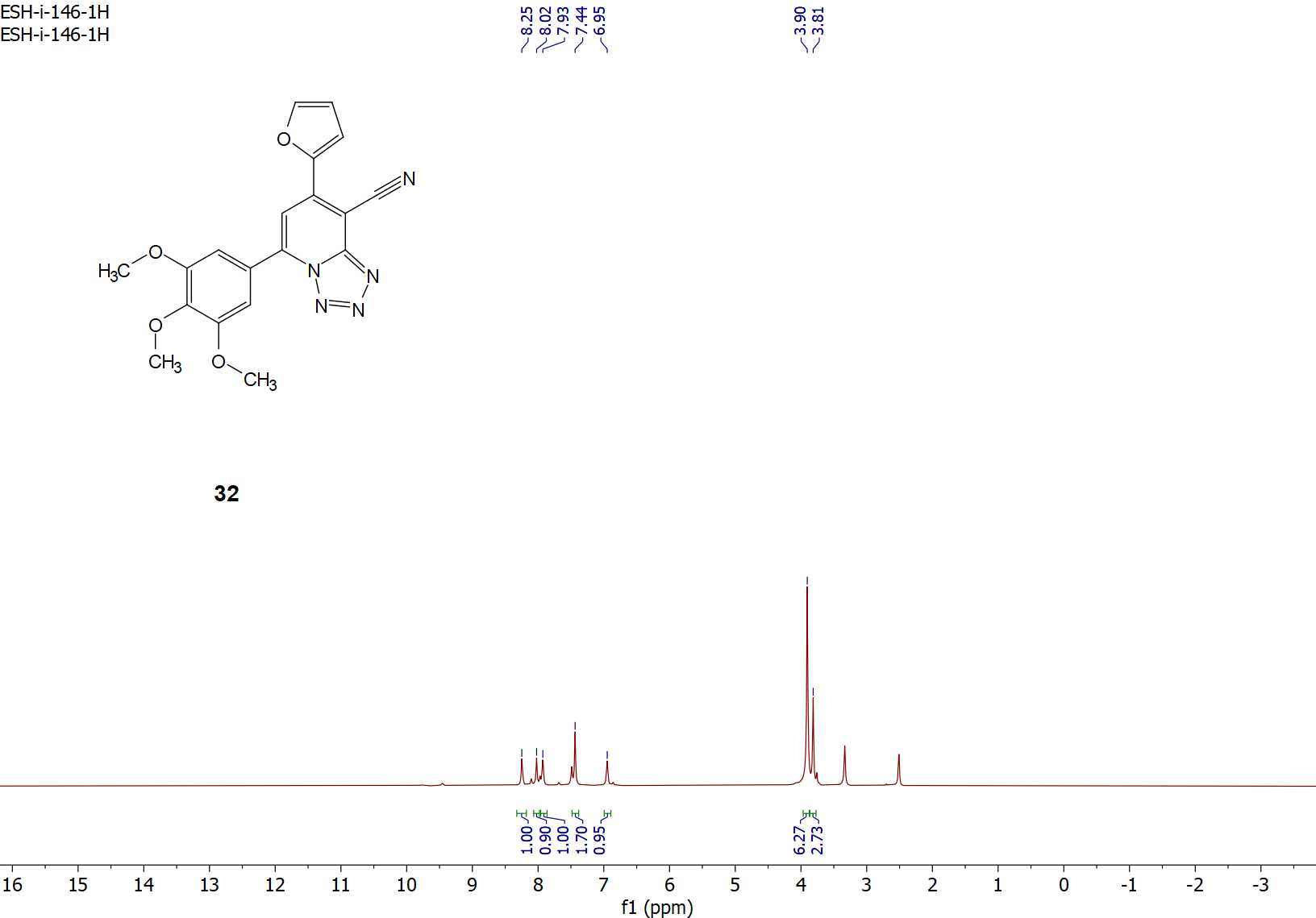


**Fig. S45:** ^1^H-NMR spectrum of compound 32.


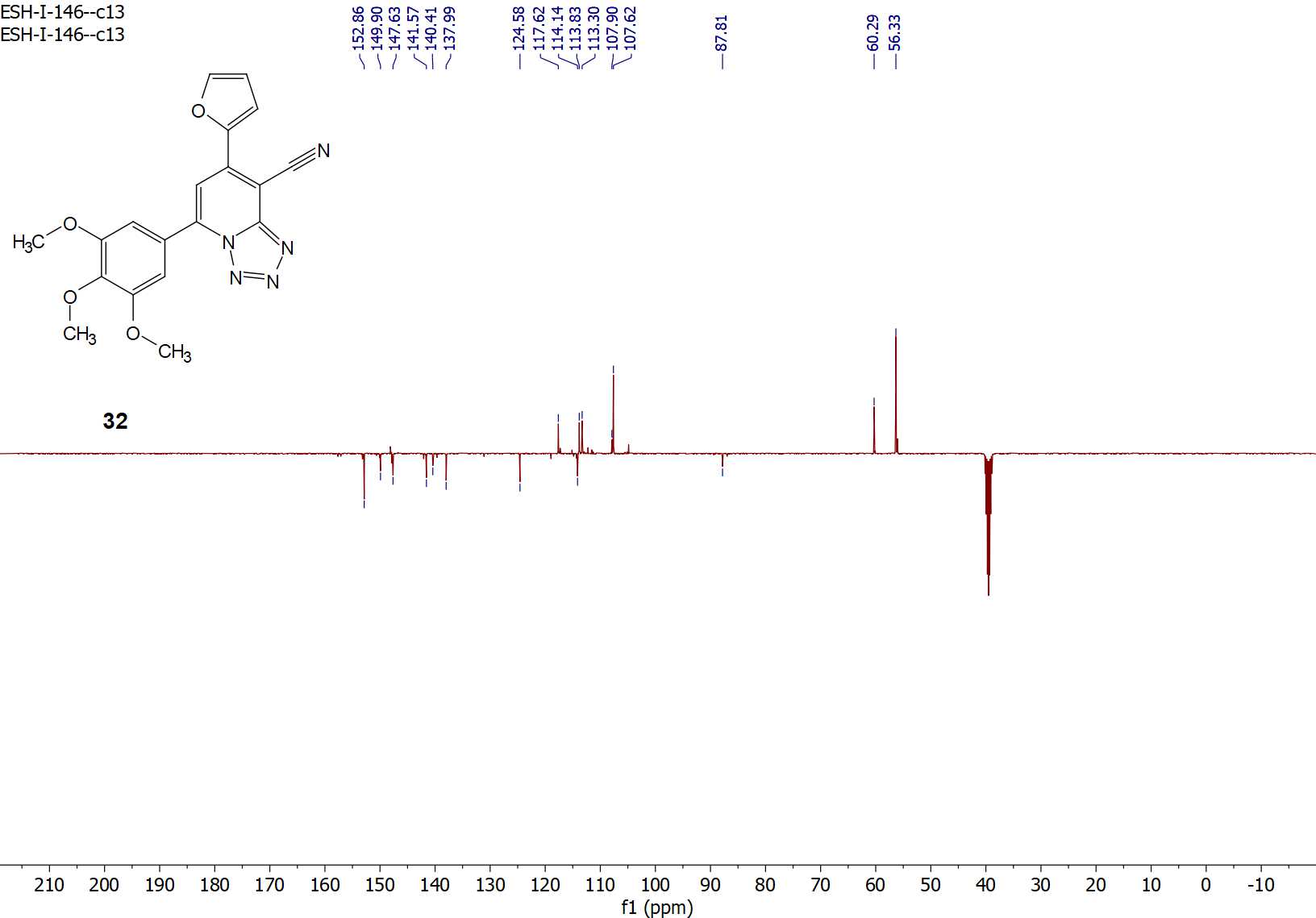


**Fig. S46:** ^13^C-APT NMR spectrum of compound 32.


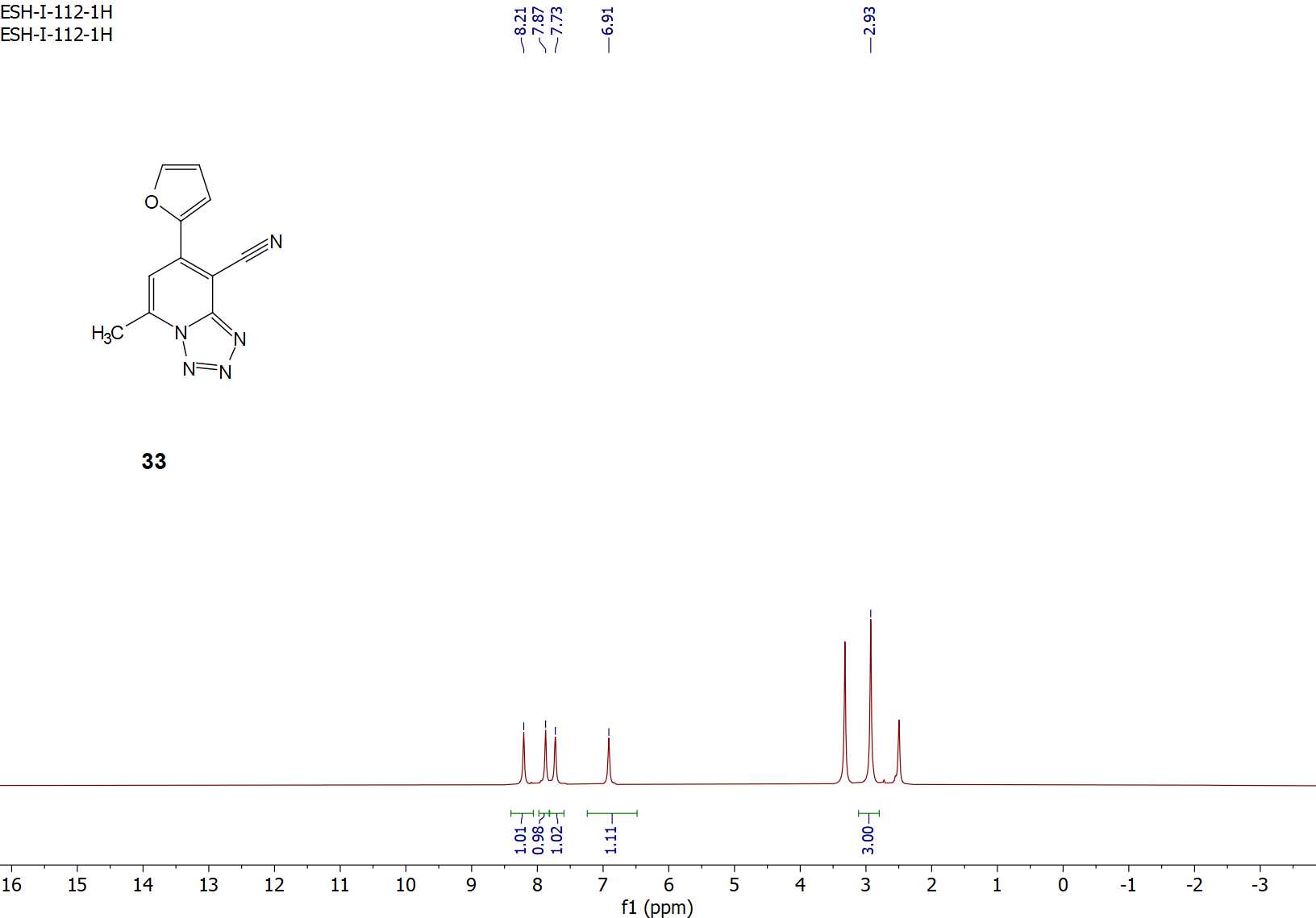


**Fig. S47:** ^1^H-NMR spectrum of compound 33.


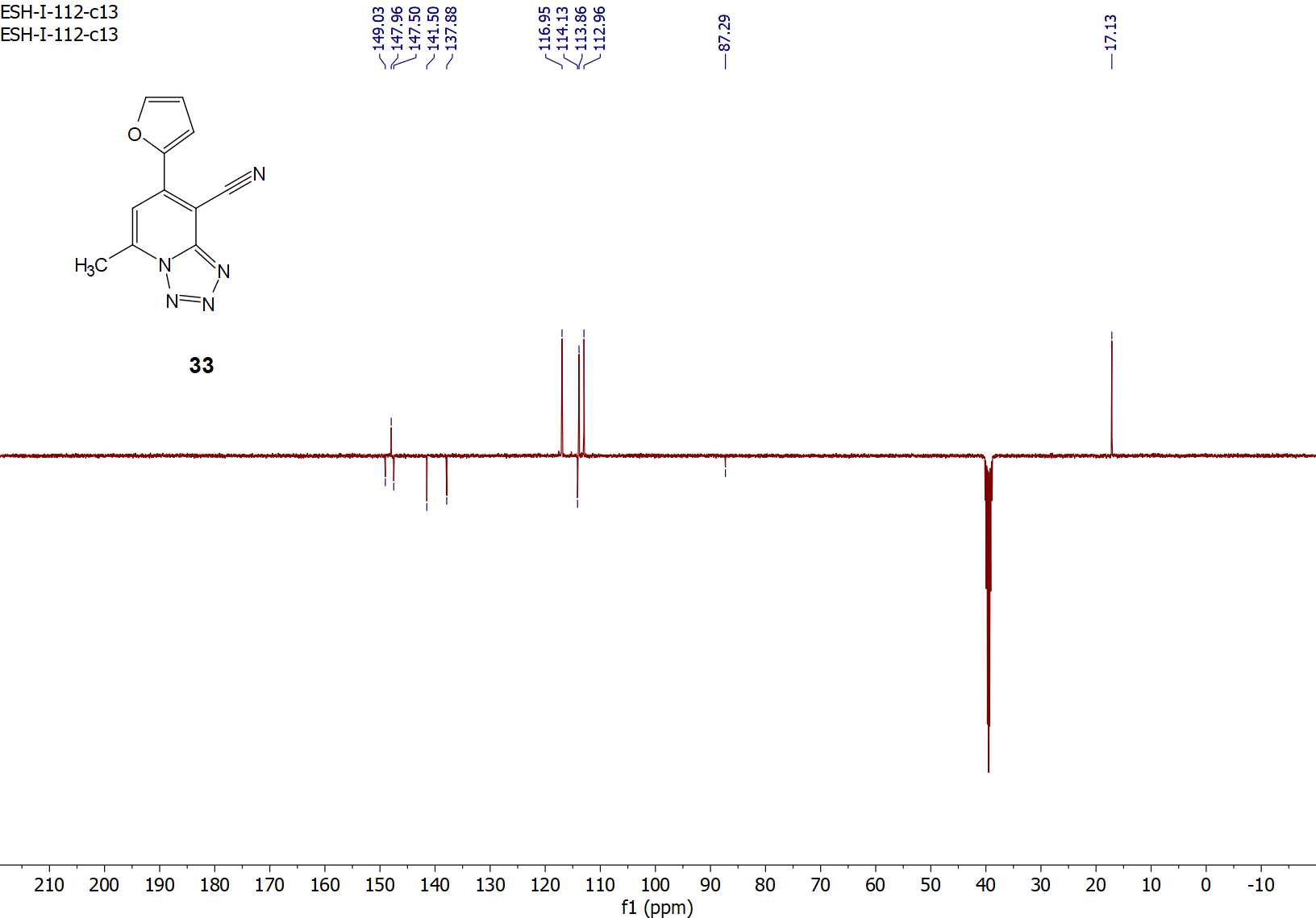


**Fig. S48:** ^13^C-APT NMR spectrum of compound 33.


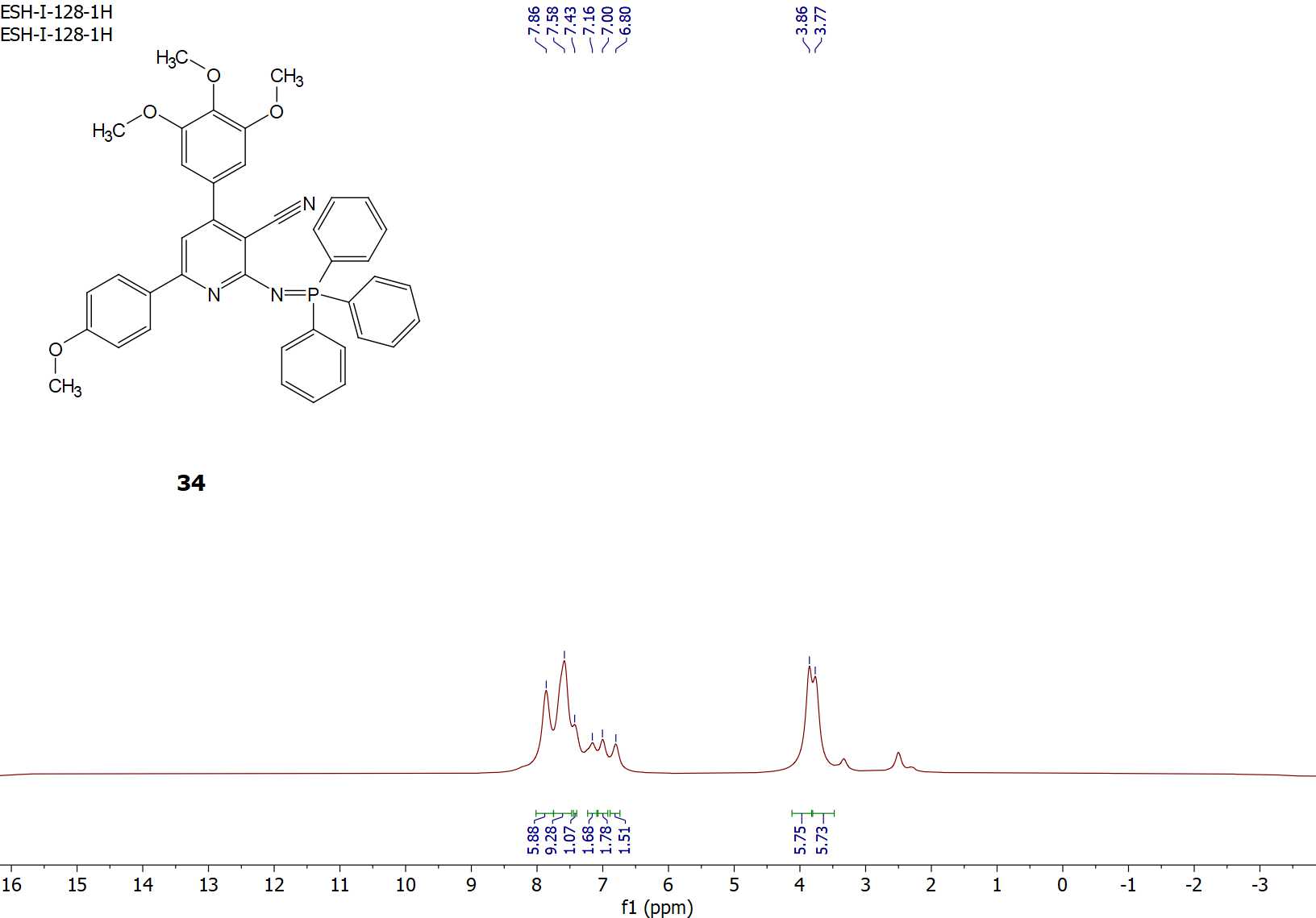


**Fig. S49:** ^1^H-NMR spectrum of compound 34.


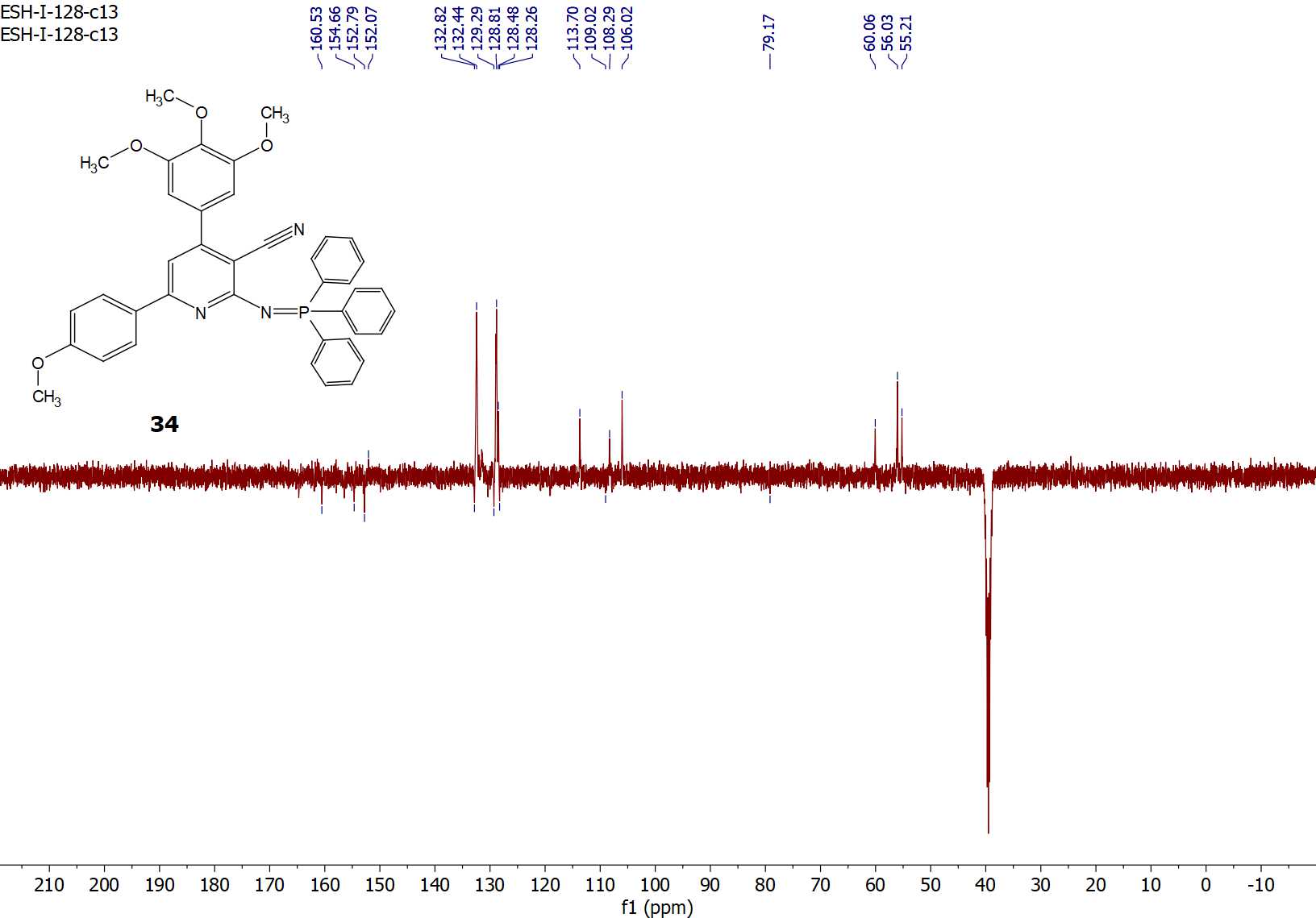


**Fig. S50:** ^13^C-APT NMR spectrum of compound 34.


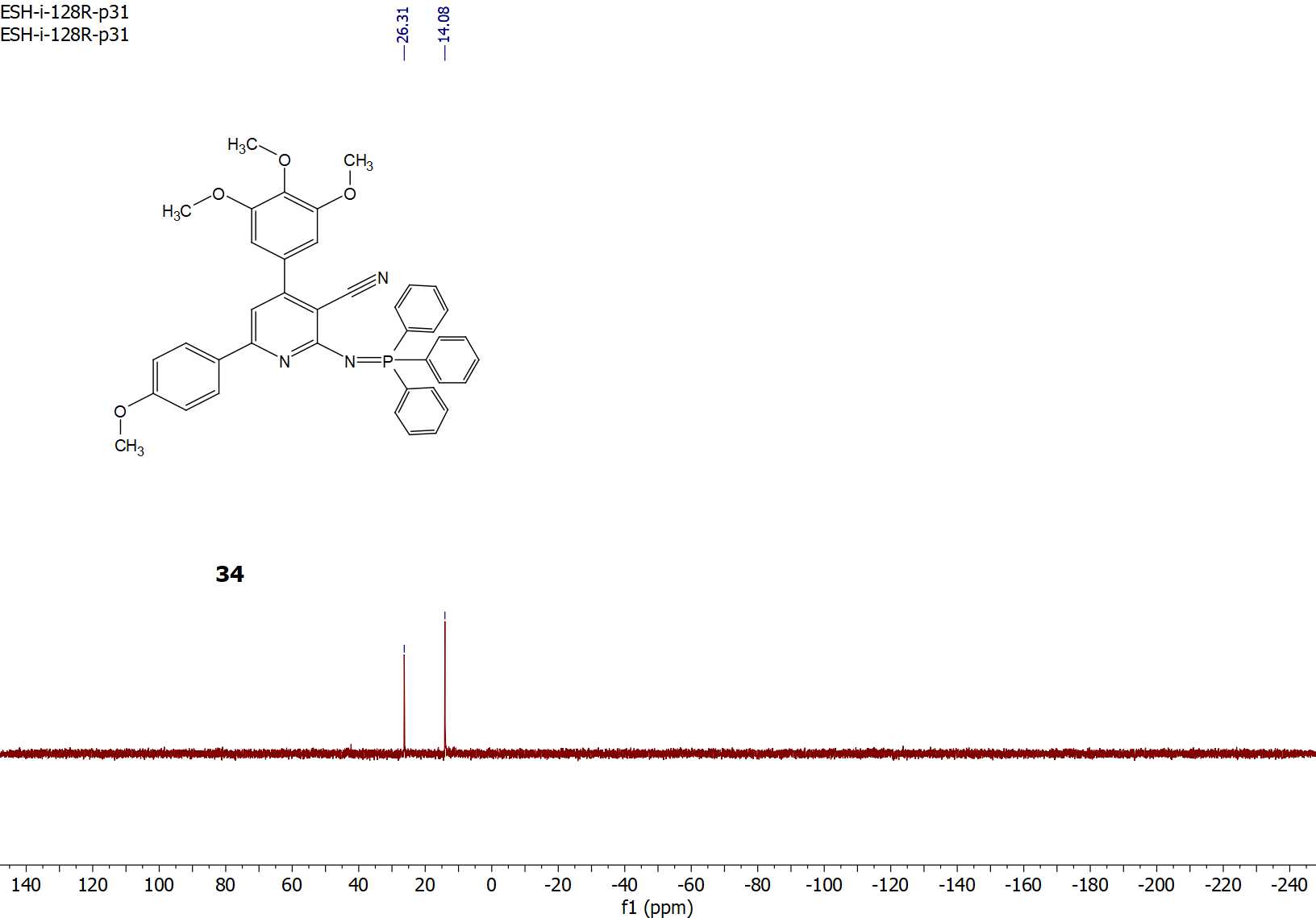


**Fig. S51:** ^31^P-NMR spectrum of compound 34.


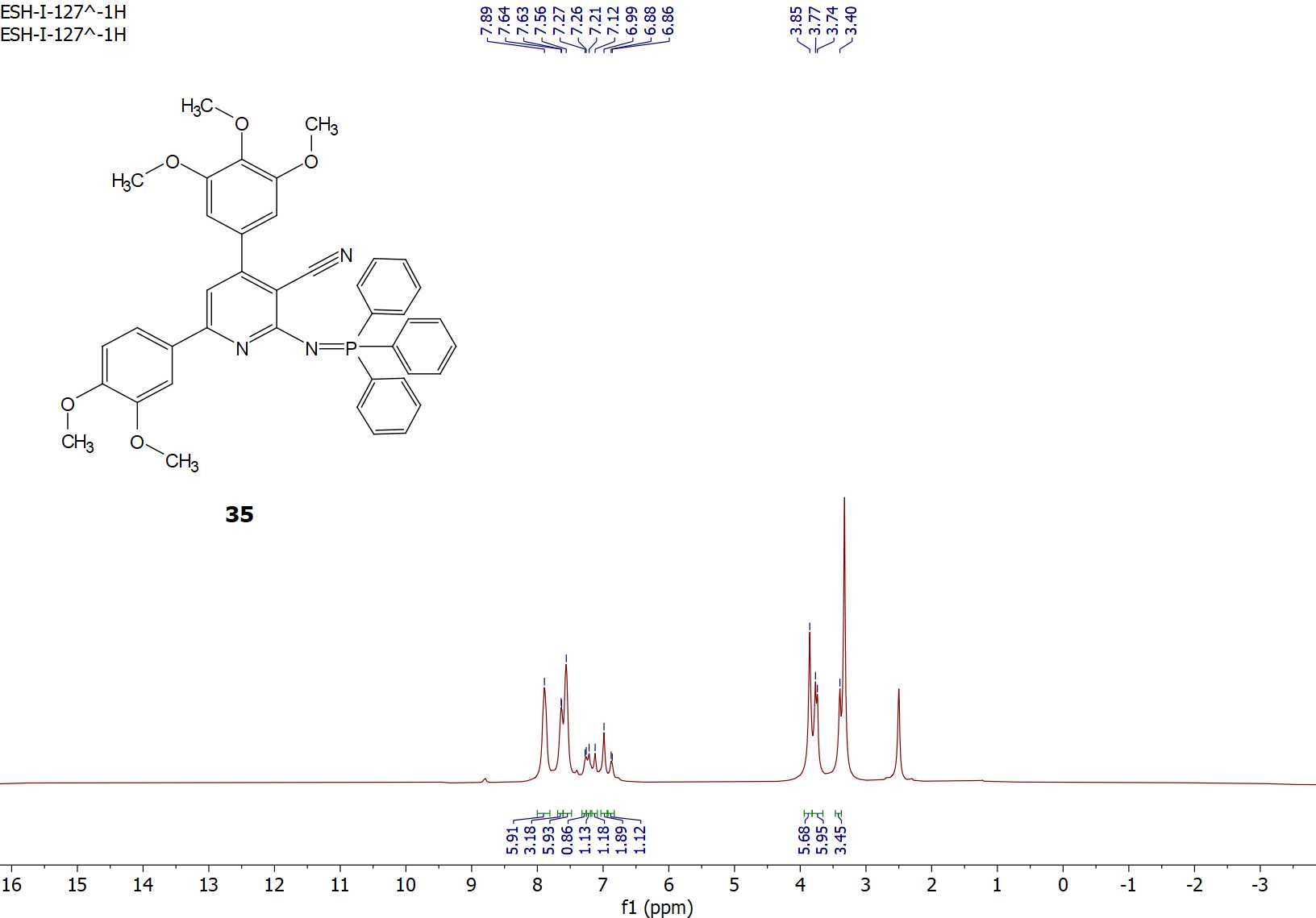


**Fig. S52:** ^1^H-NMR spectrum of compound 35.


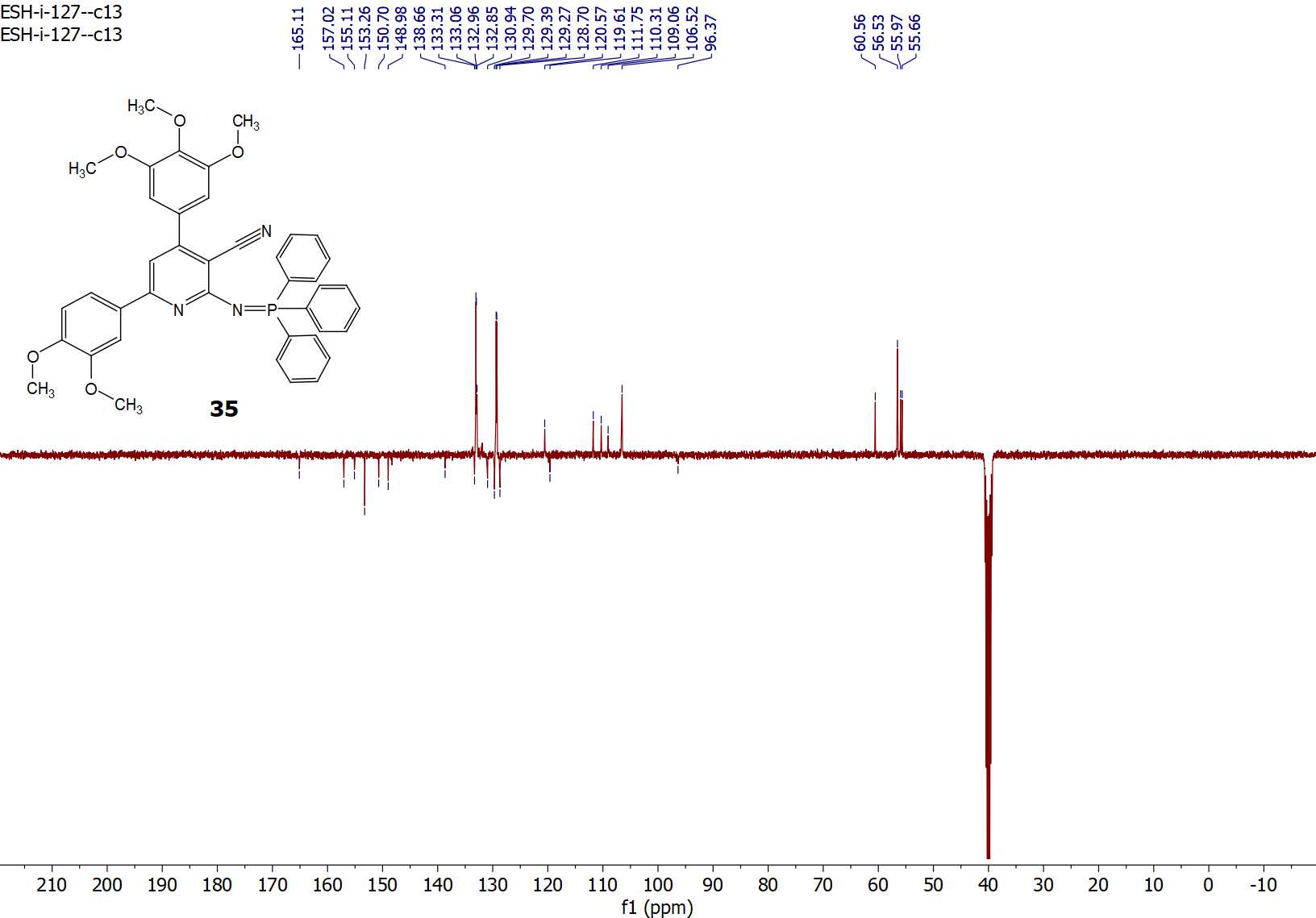


**Fig. S53:** ^13^C-APT NMR spectrum of compound 35.


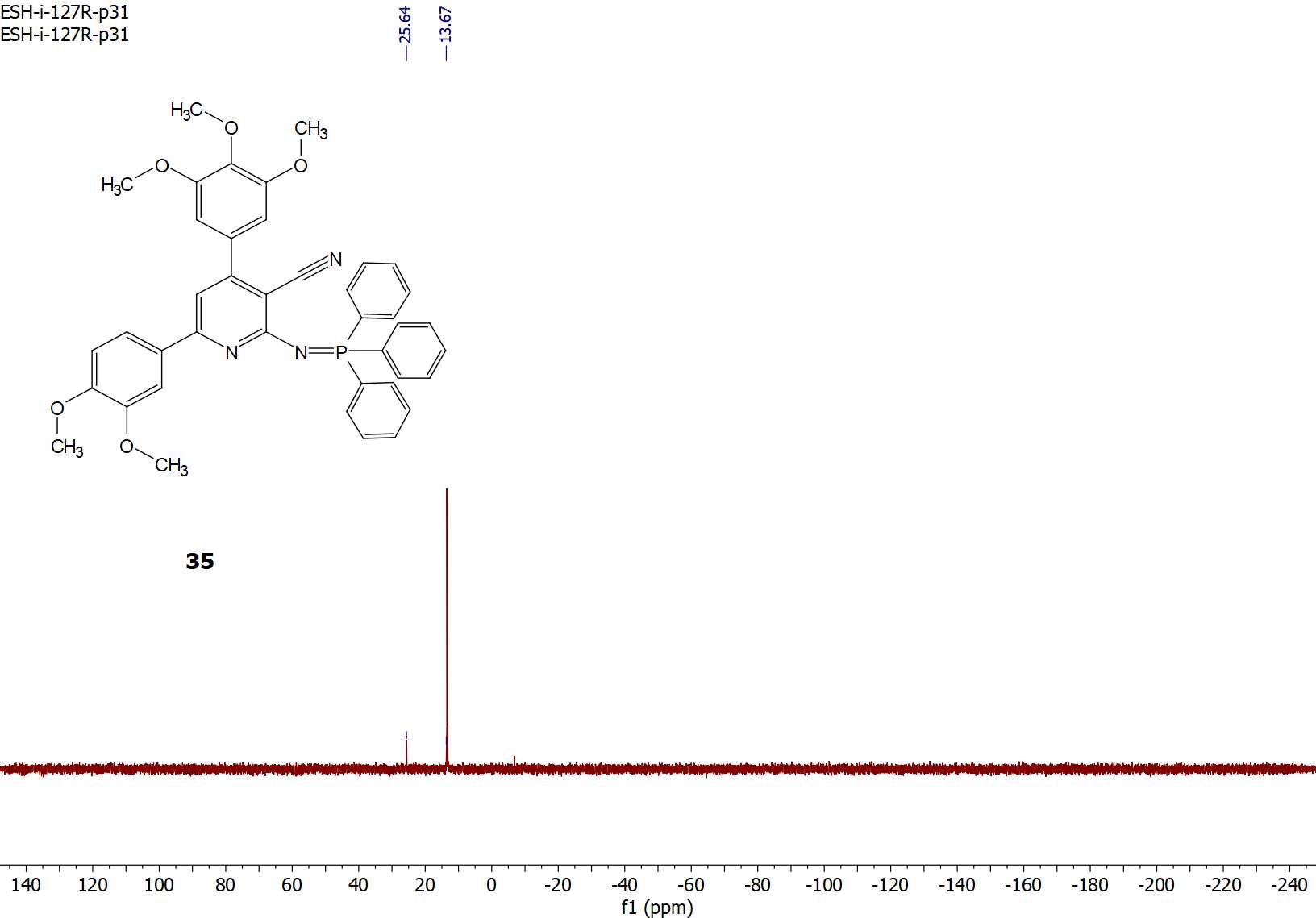


**Fig. S54:** ^31^P-NMR spectrum of compound 35.


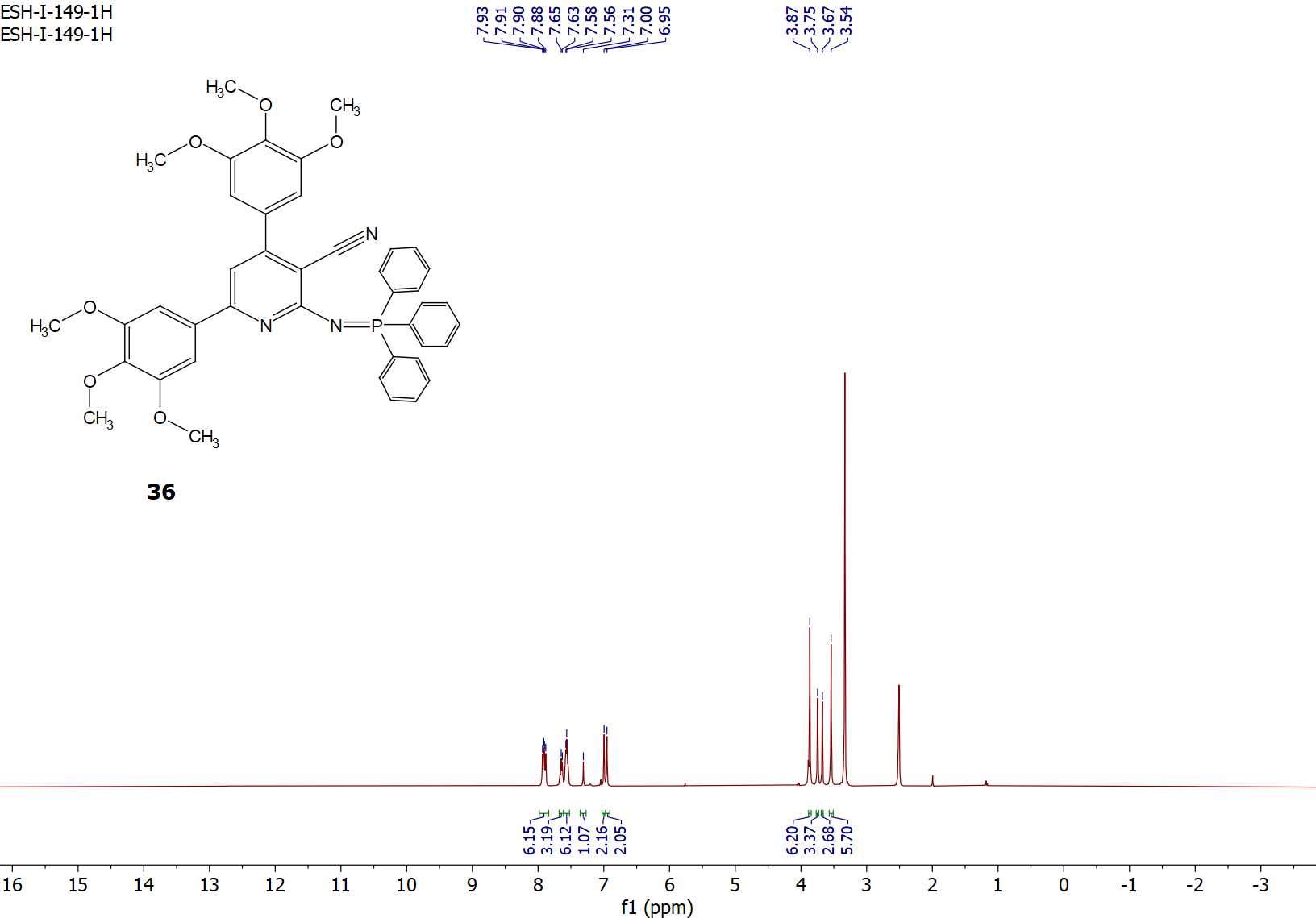


**Fig. S55:** ^1^H-NMR spectrum of compound 36.


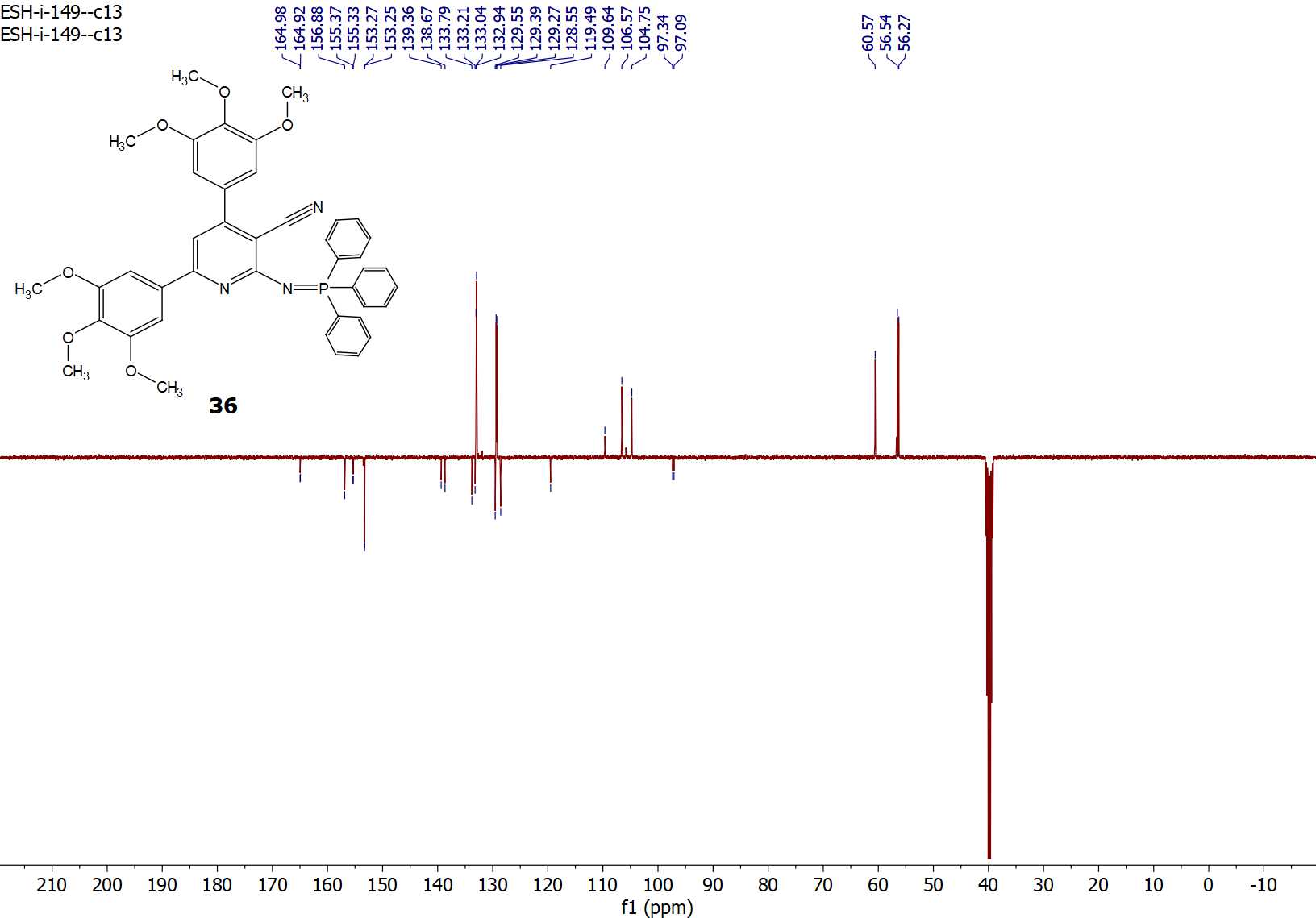


**Fig. S56:** ^13^C-APT NMR spectrum of compound 36.


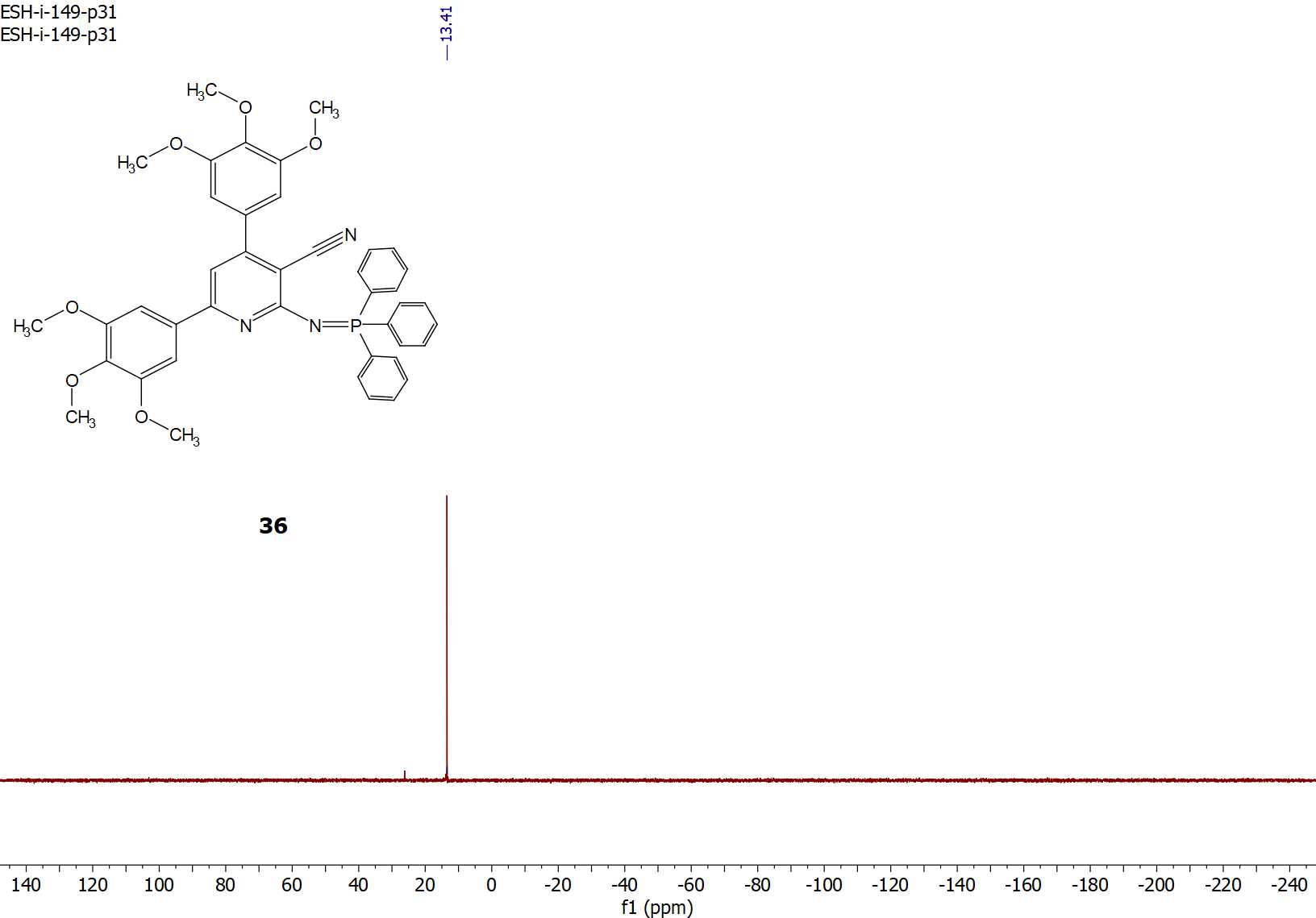


**Fig. S57:** ^31^P-NMR spectrum of compound 36.


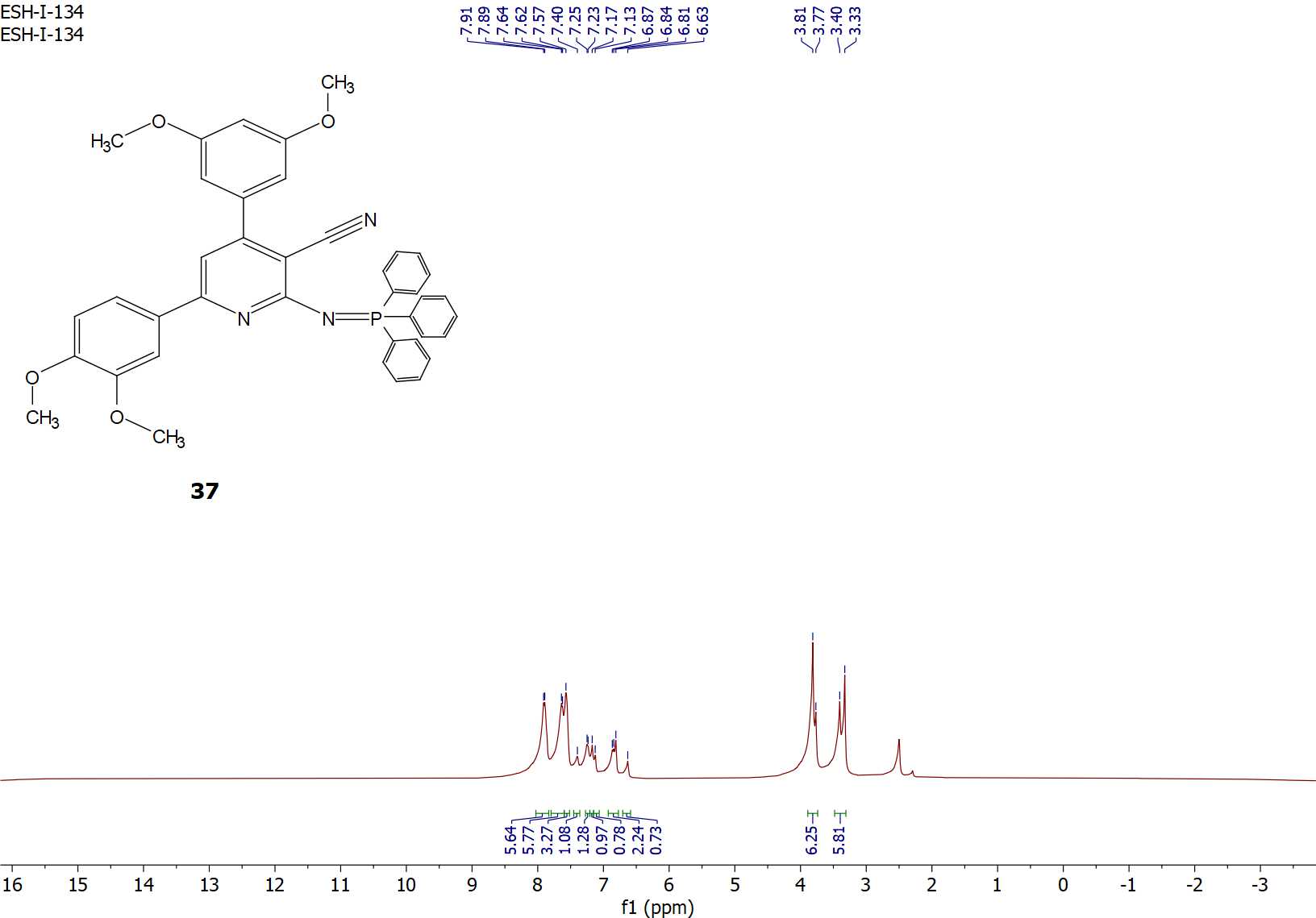


**Fig. S58:** ^1^H-NMR spectrum of compound 37.


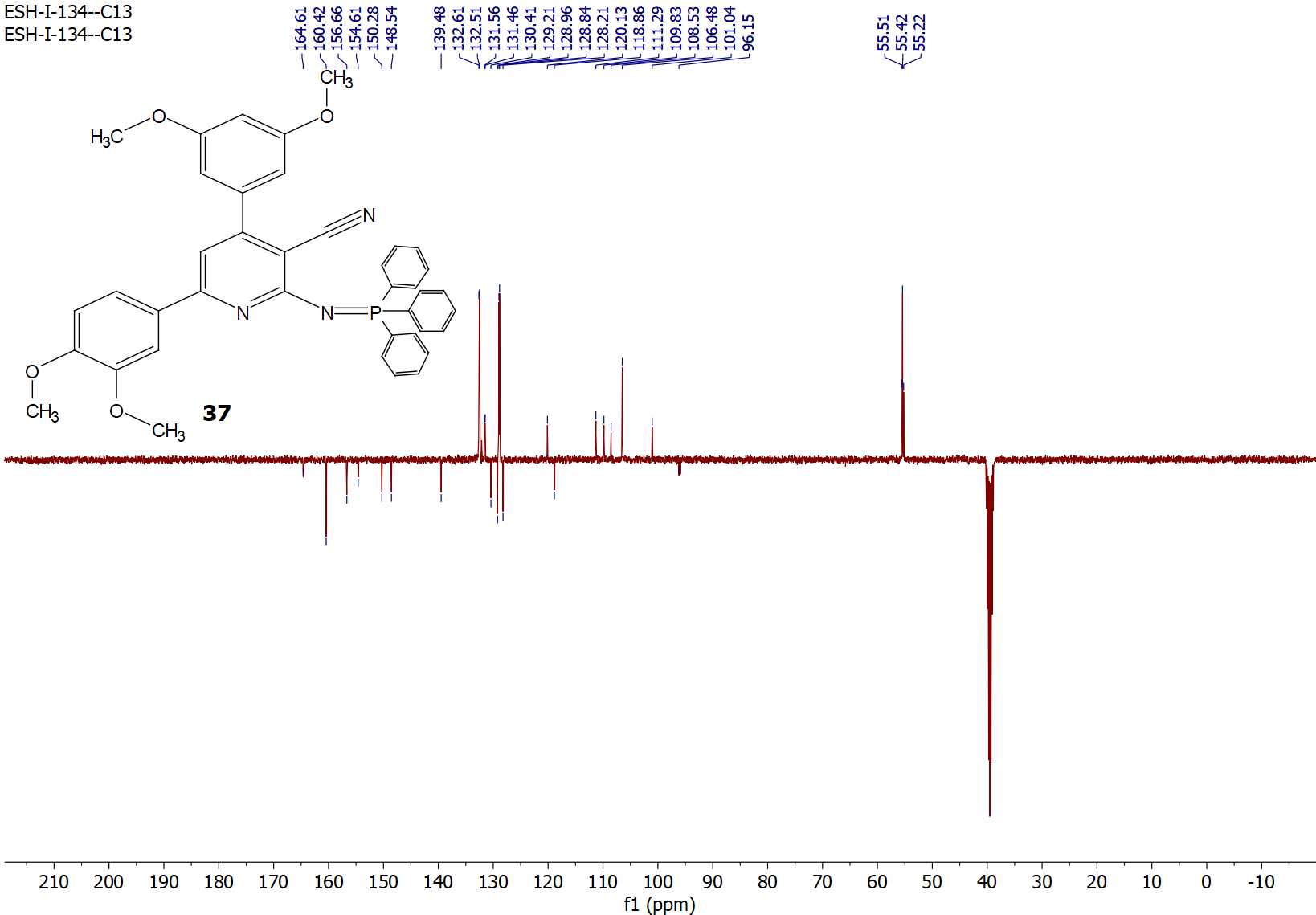


**Fig. S59:** ^13^C-APT NMR spectrum of compound 37.


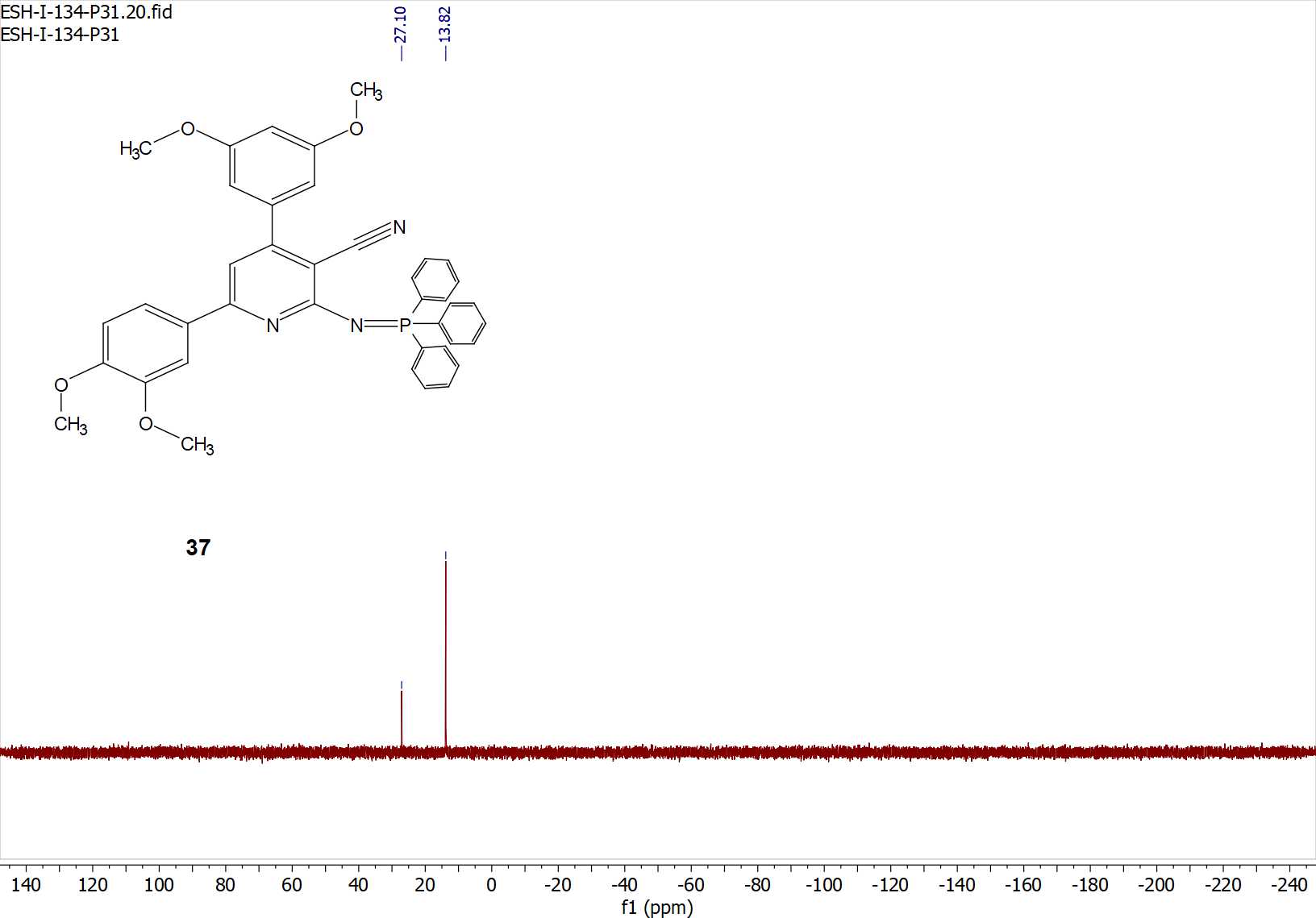


**Fig. S60:** ^31^P-NMR spectrum of compound 37.


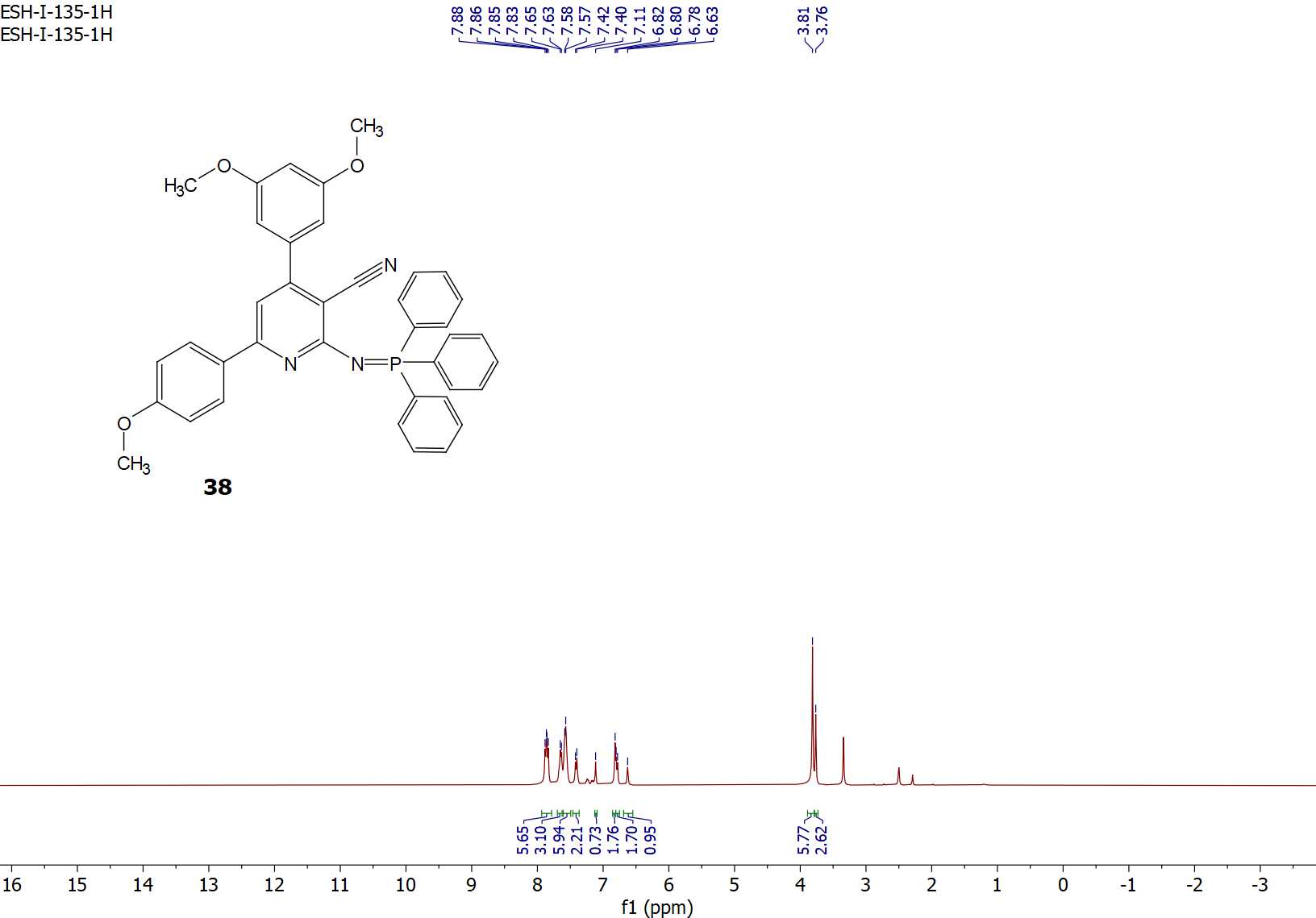


**Fig. S61:** ^1^H-NMR spectrum of compound 38.


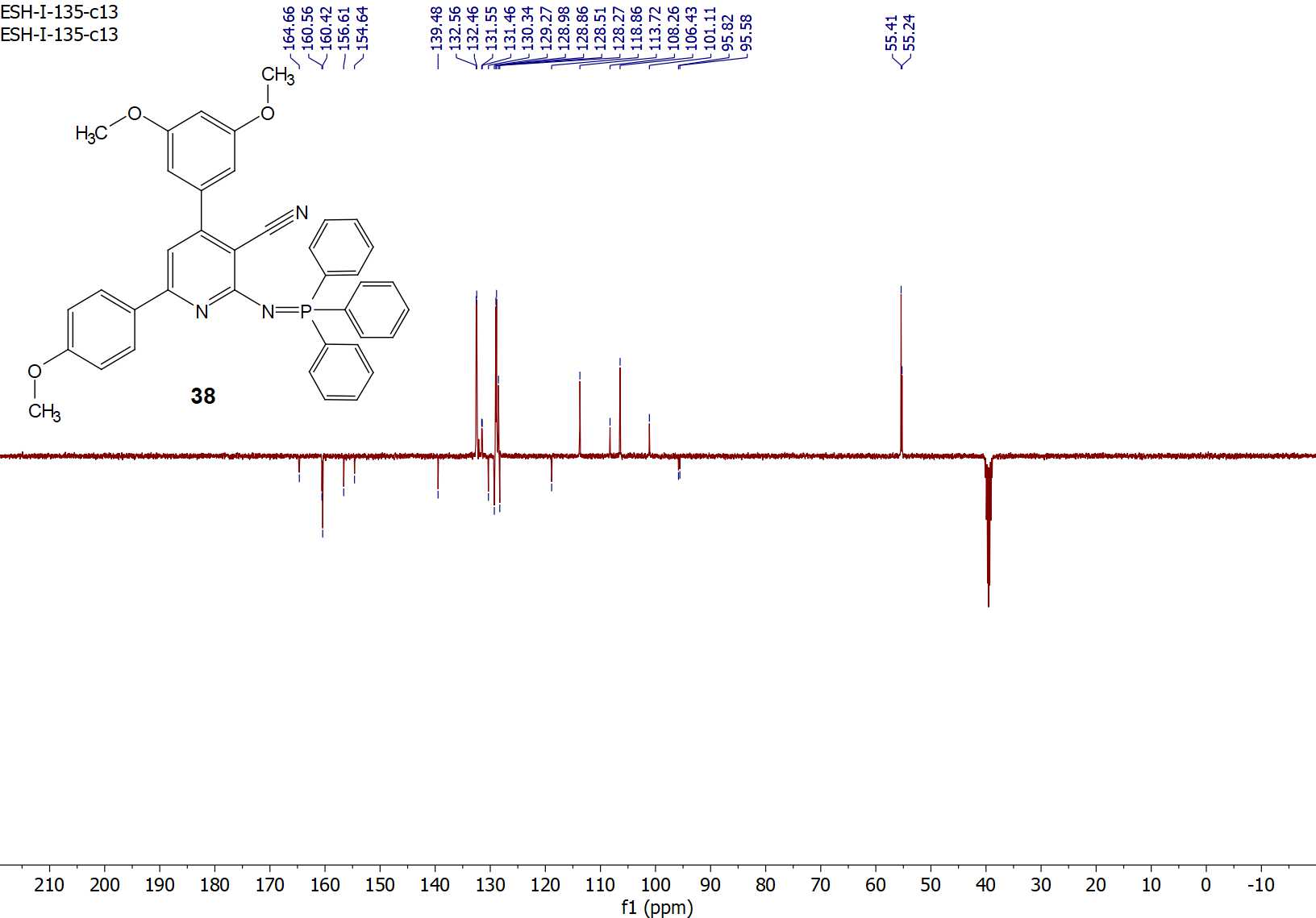


**Fig. S62:** ^13^C-APT NMR spectrum of compound 38.


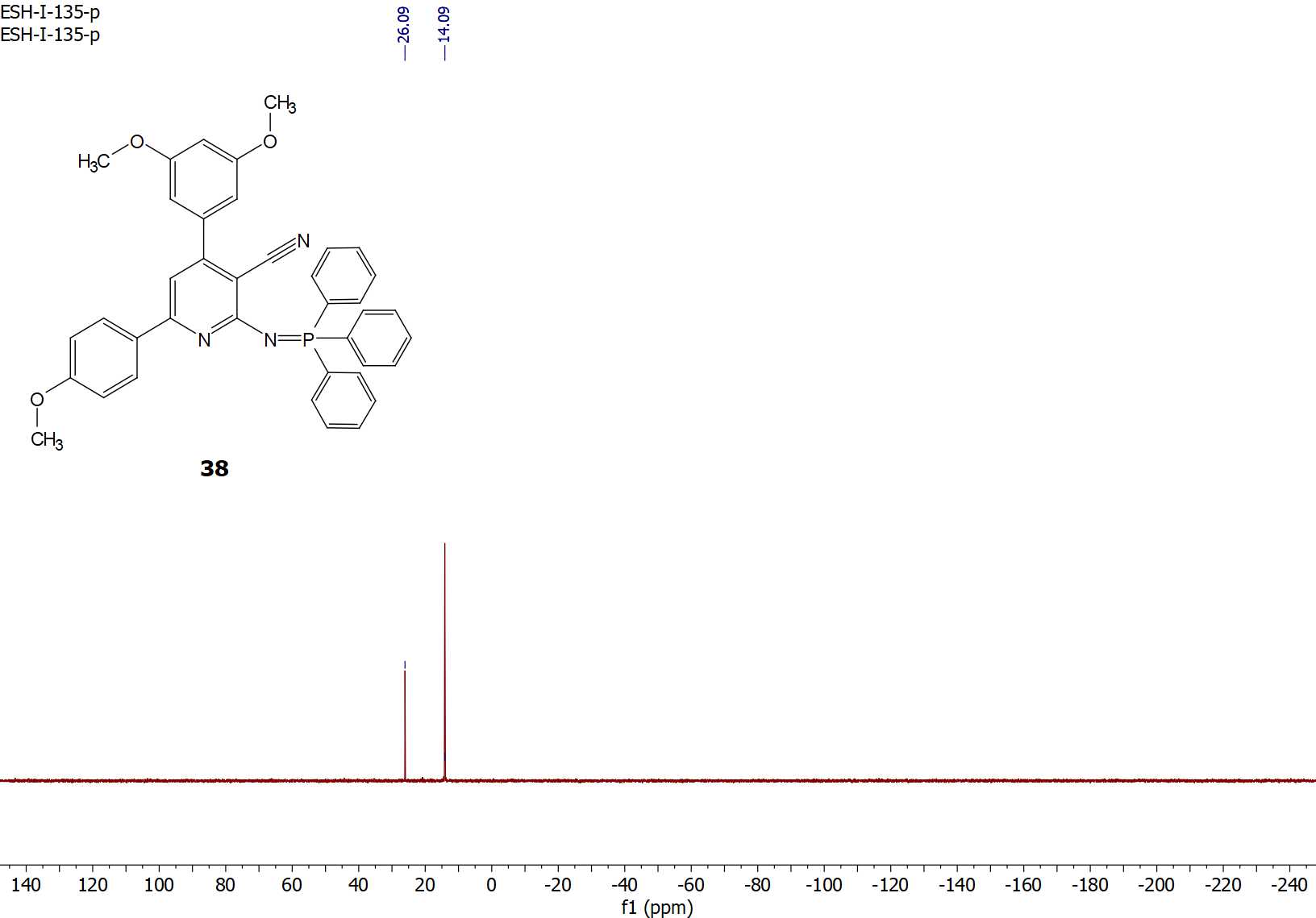


**Fig. S63:** ^31^P-NMR spectrum of compound 38.


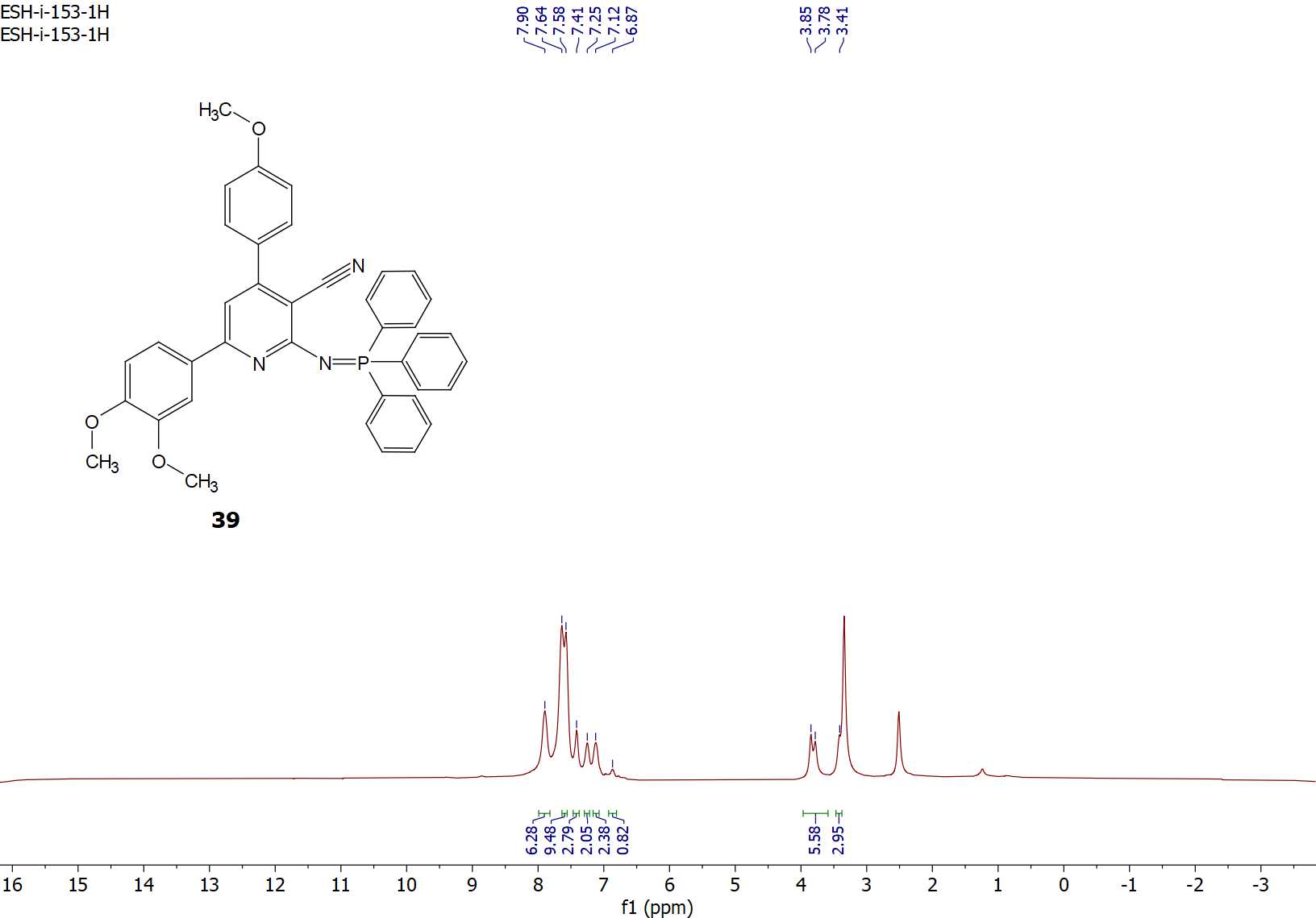


**Fig. S64:** ^1^H-NMR spectrum of compound 39.


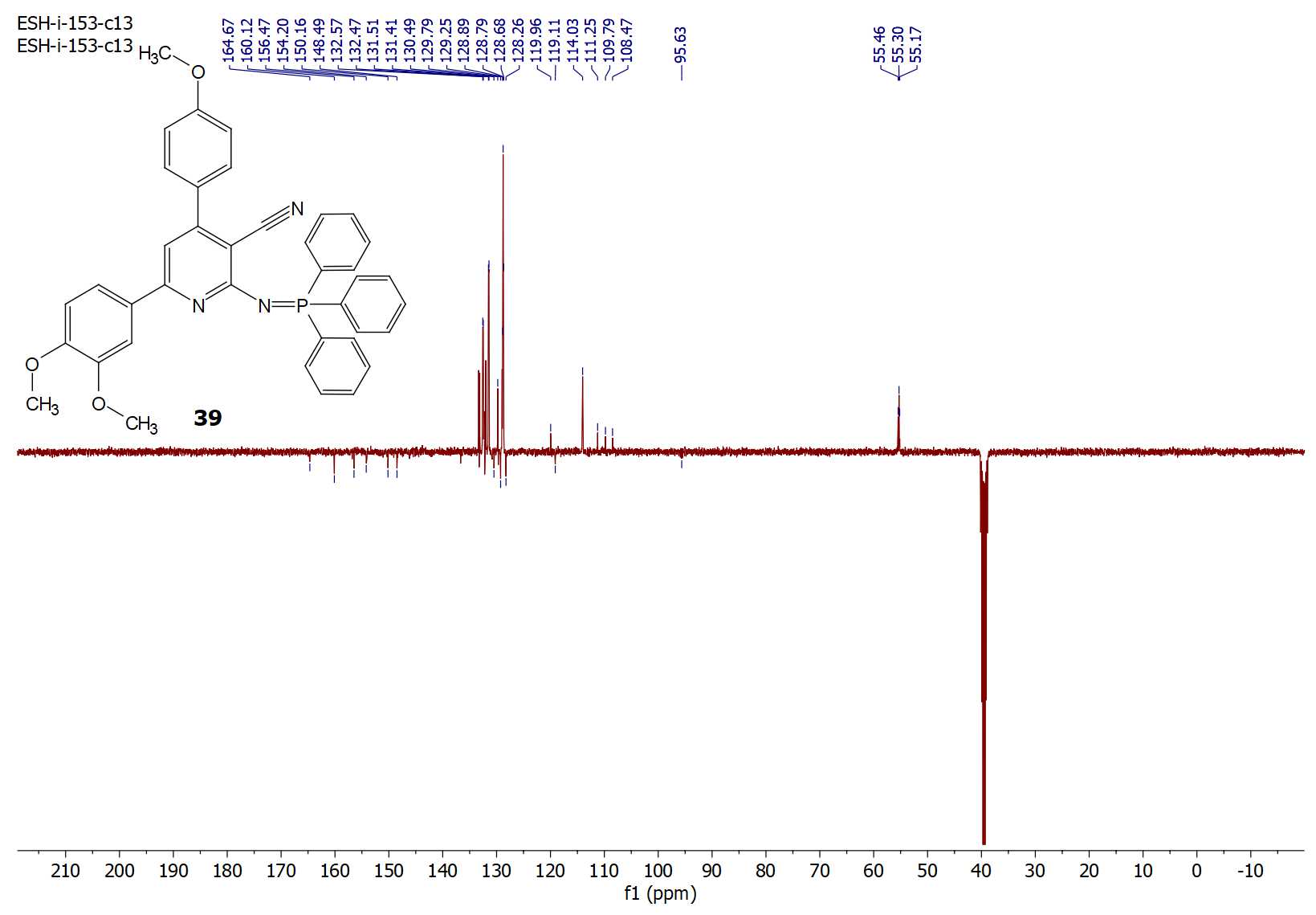


**Fig. S65:** ^13^C-APT NMR spectrum of compound 39.


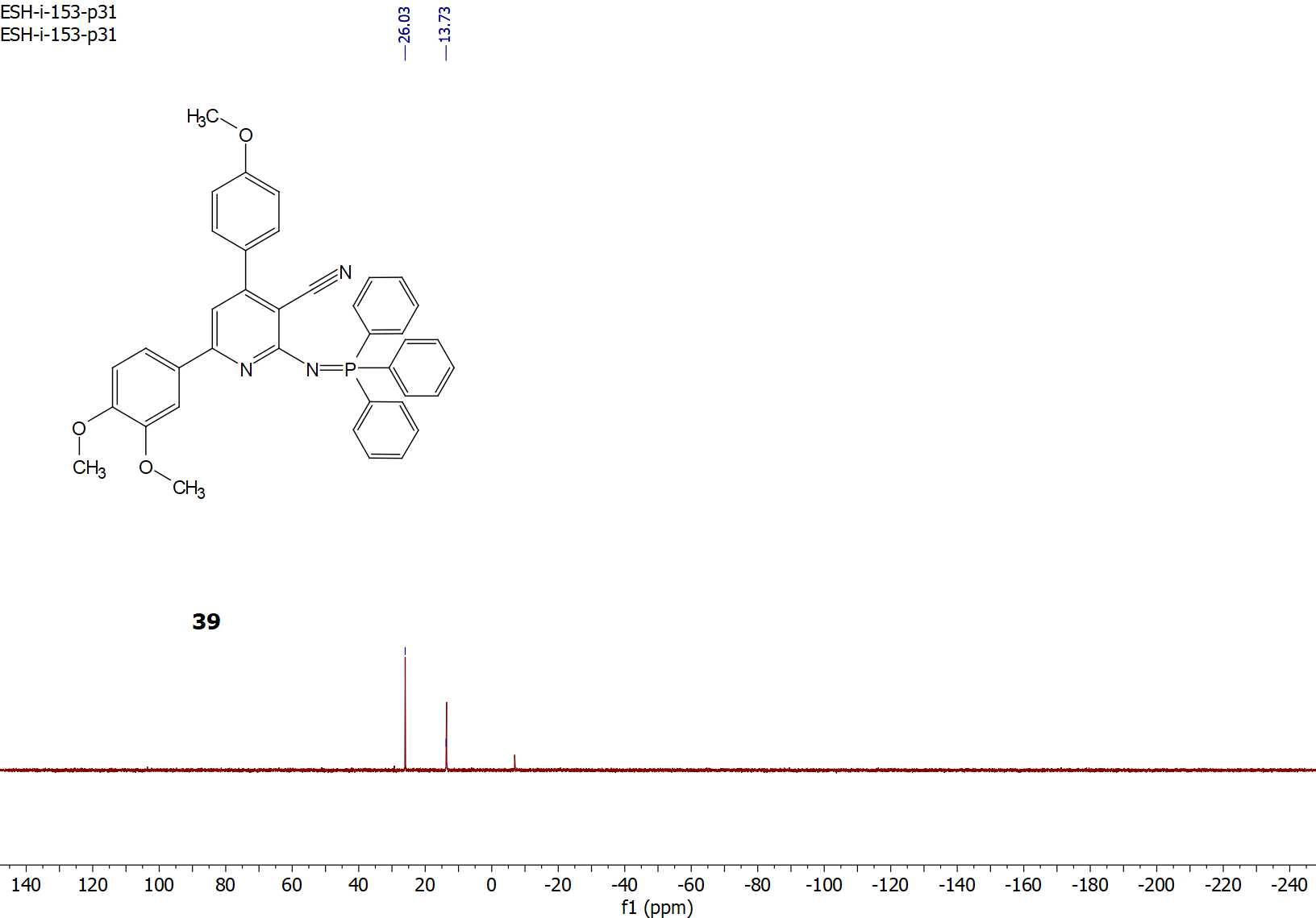


**Fig. S66:** ^31^P-NMR spectrum of compound 39.


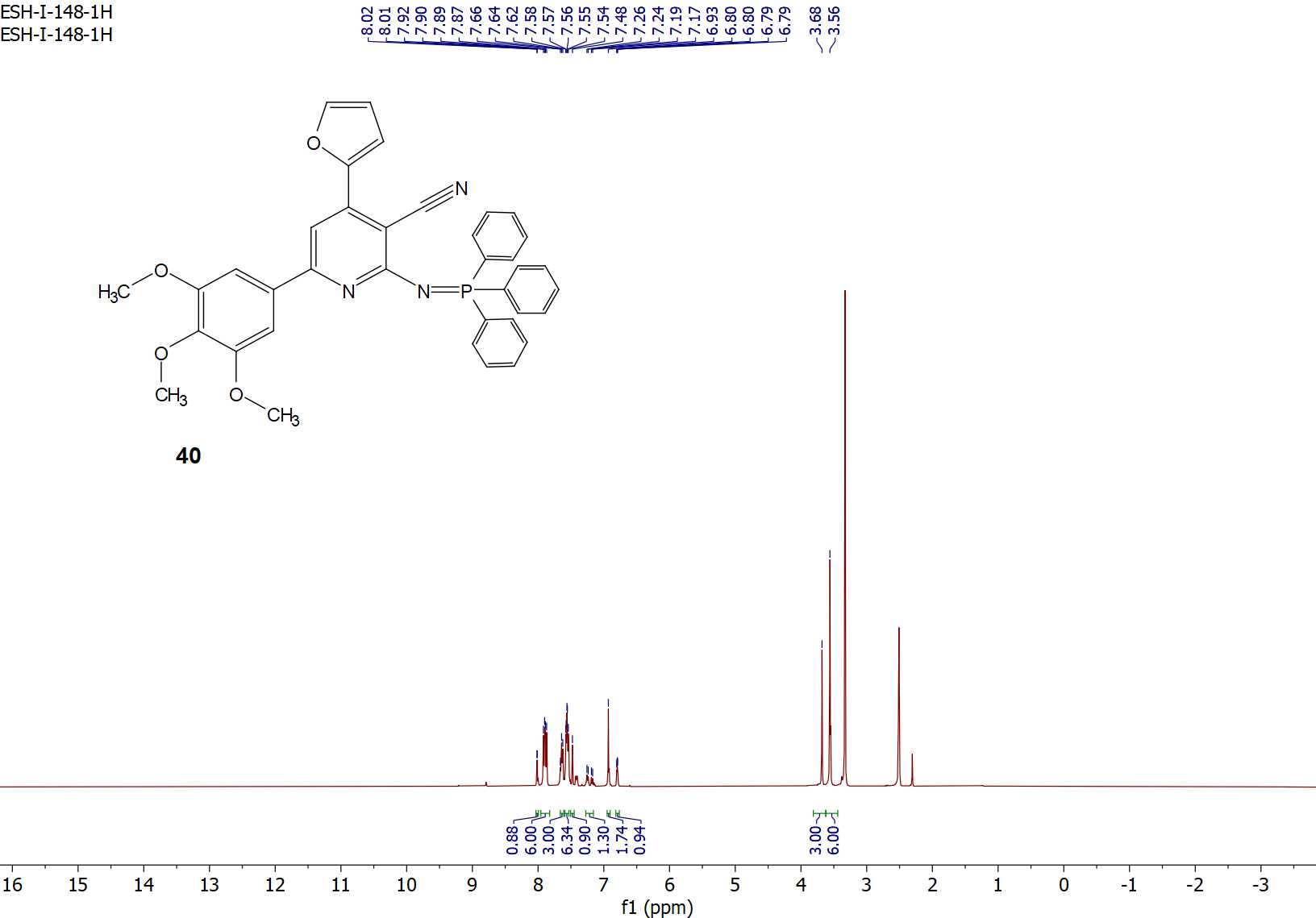


**Fig. S67:** ^1^H-NMR spectrum of compound 40.


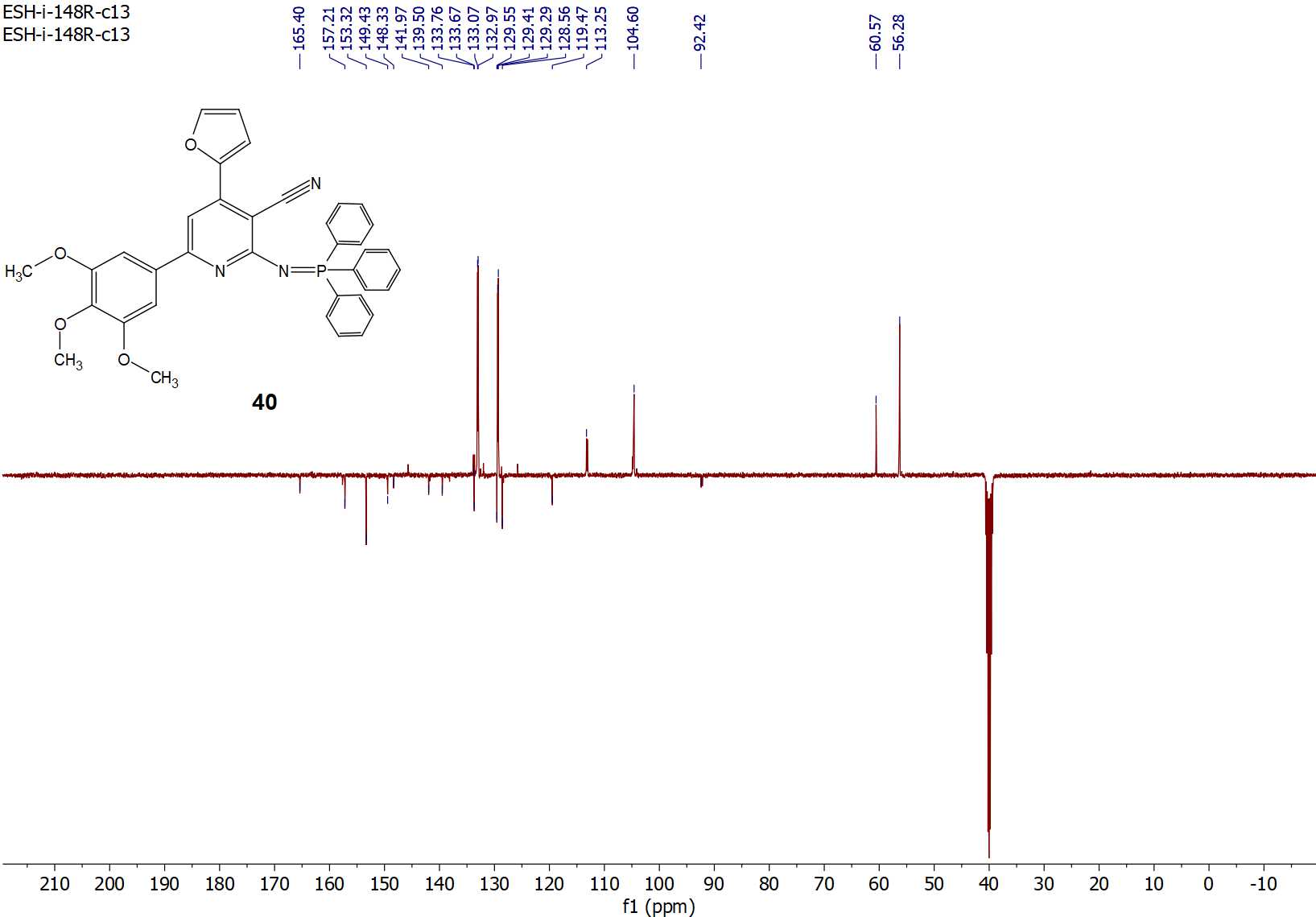


**Fig. S68:** ^13^C-APT NMR spectrum of compound 40.


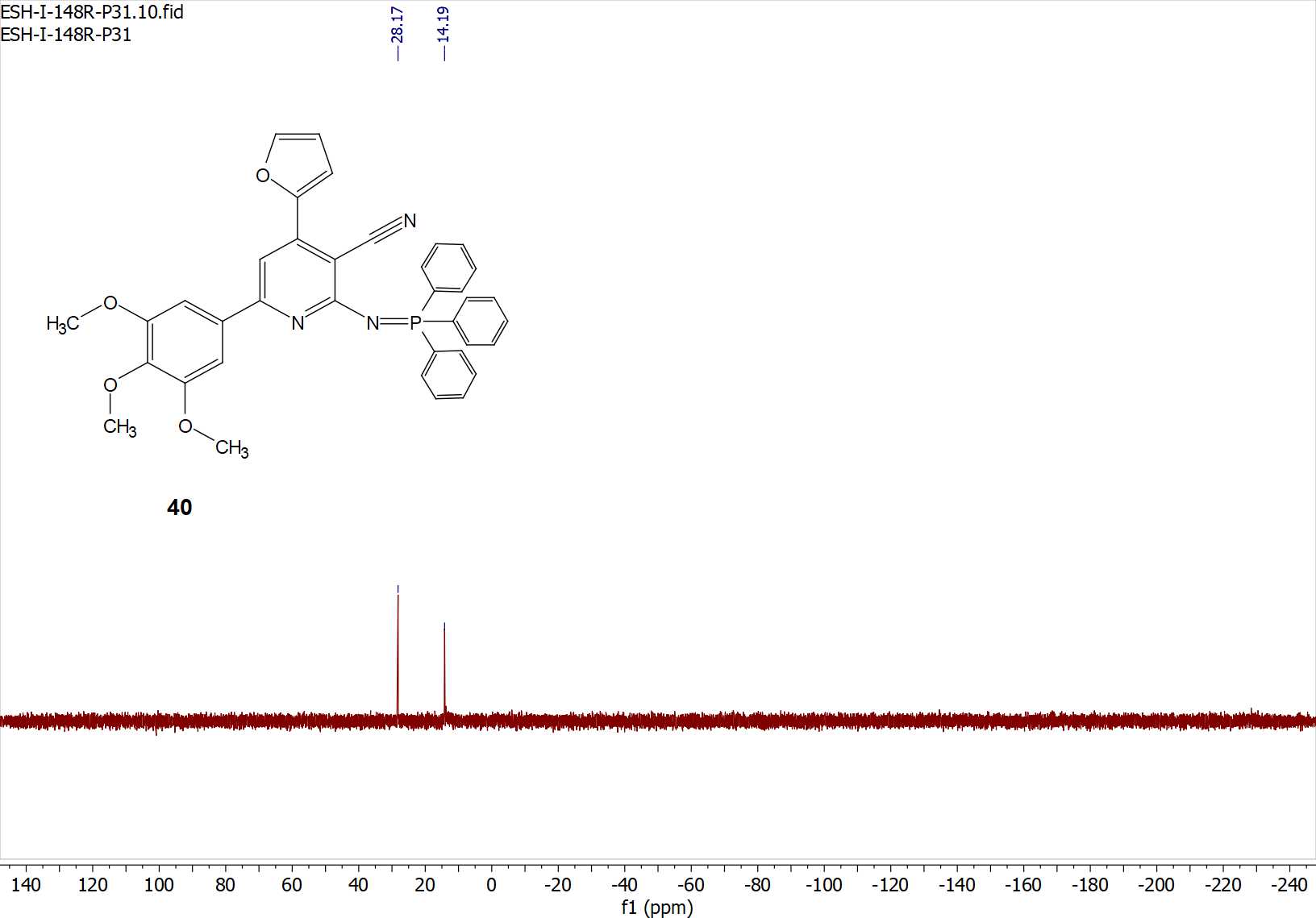


**Fig. S69:** ^31^P-NMR spectrum of compound 40.


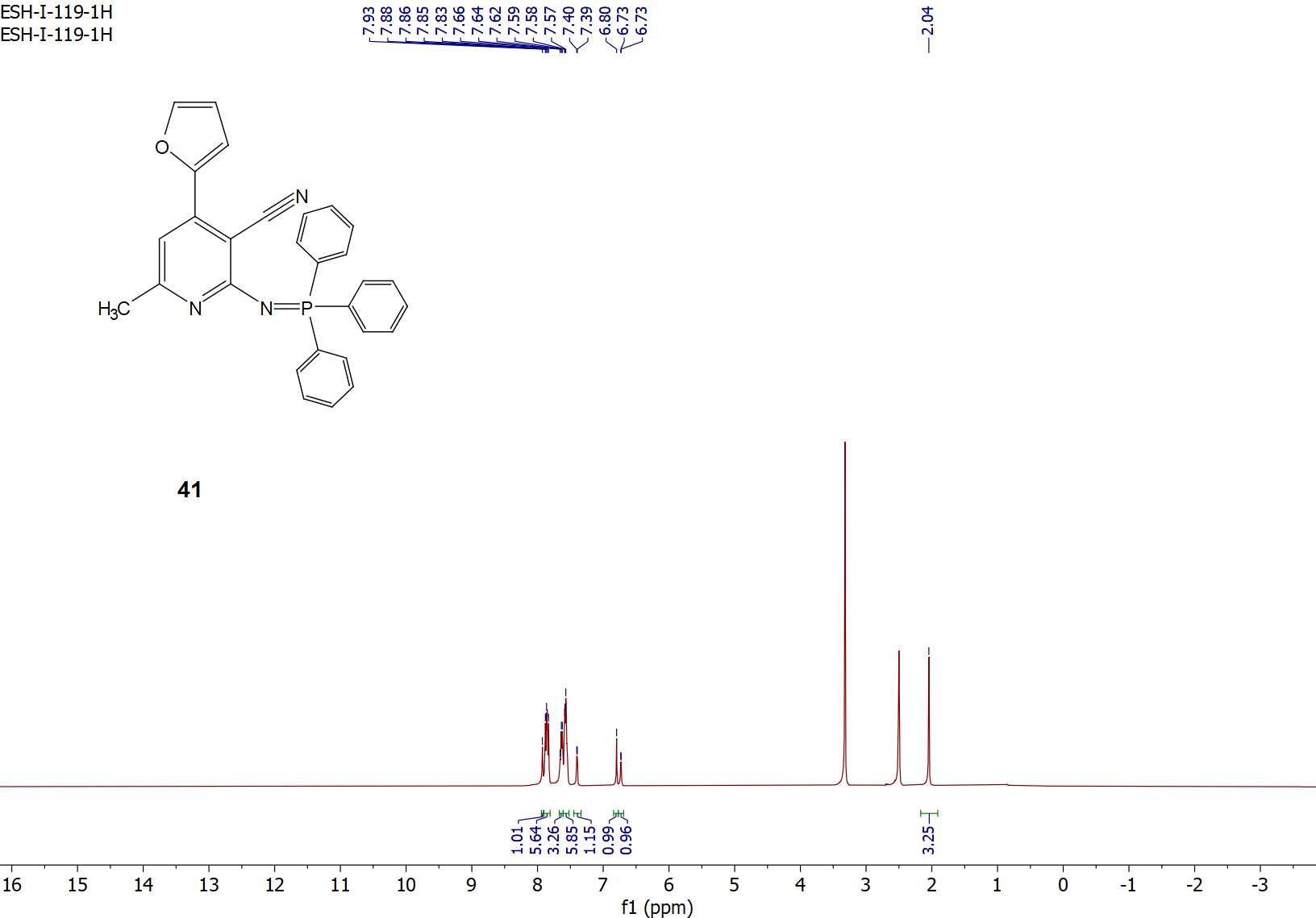


**Fig. S70:** ^1^H-NMR spectrum of compound 41.


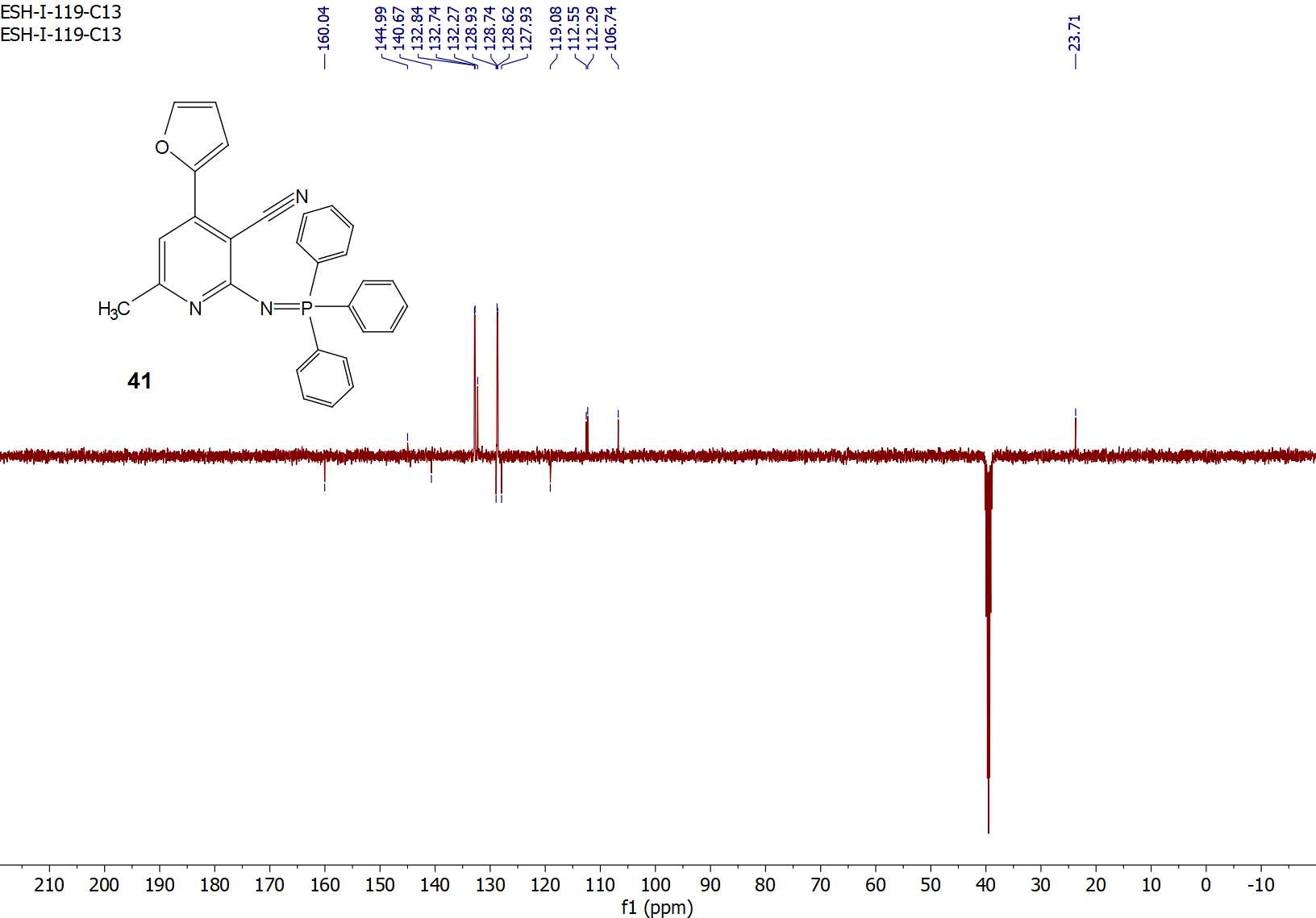


**Fig. S71:** ^13^C-APT NMR spectrum of compound 41.


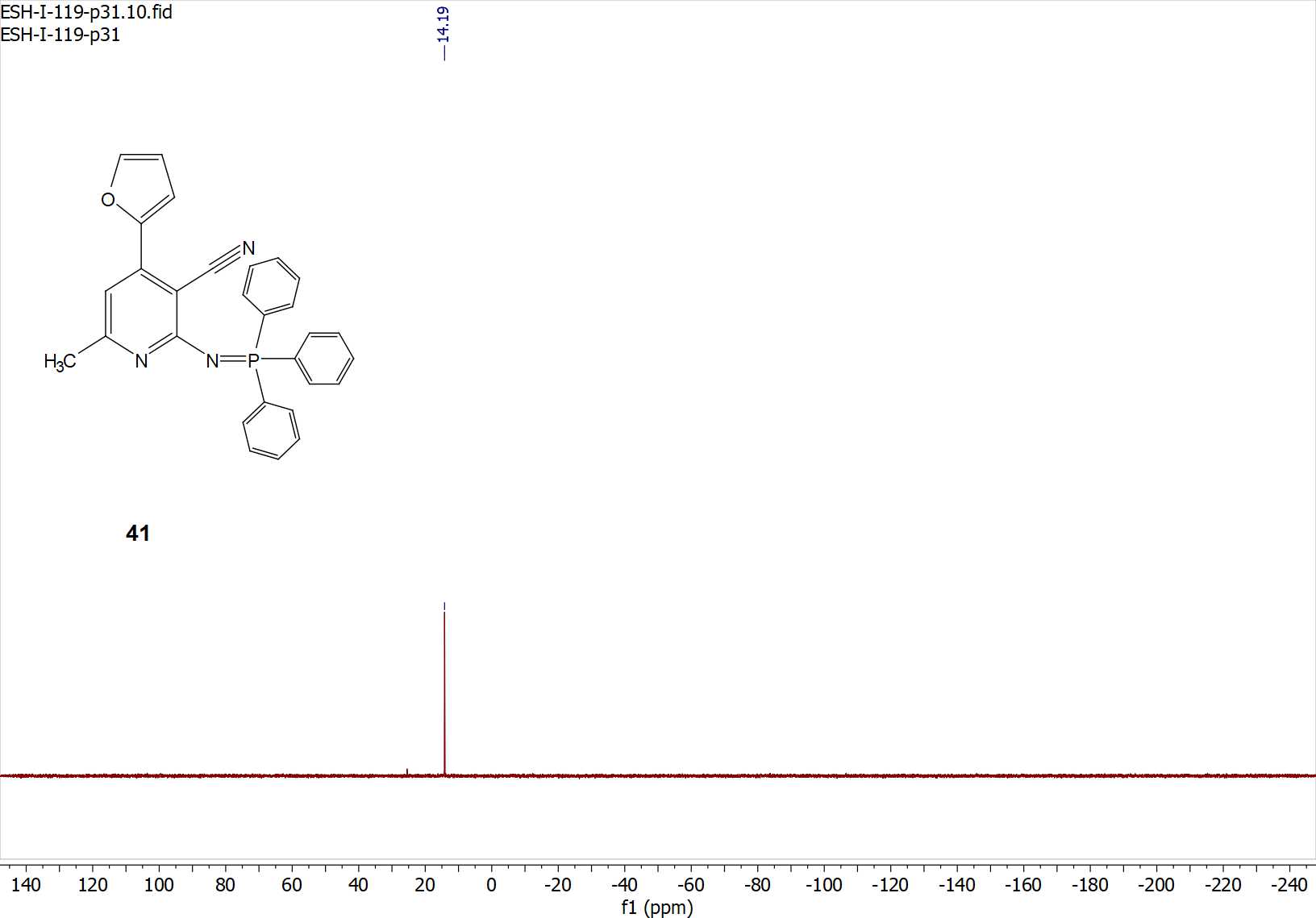


**Fig. S72:** ^31^P-NMR spectrum of compound 41.

| Compounds’ codes in paper | Corresponding codes in elemental analysis and mass spectroscopy |
| --- | --- |
| 13 | ESH-4 |
| 14 | ESH-37 |
| 16 | ESH-98C |
| 18 | ESH-33 |
| 19 | ESH-43 |
| 20 | ESH-137 |
| 21 | ESH-41 |
| 22 | ESH-45 |
| 23 | ESH-143 |
| 24 | ESH-139 |
| 26 | ESH-124 |
| 27 | ESH-125 |
| 28 | ESH-147 |
| 29 | ESH-132 |
| 30 | ESH-133 |
| 31 | ESH-150 |
| 32 | ESH-146 |
| 33 | ESH-112 |
| 34 | ESH-128 |
| 35 | ESH-127 |
| 36 | ESH-149 |
| 37 | ESH-134 |
| 38 | ESH-135 |
| 39 | ESH-153 |
| 40 | ESH-148 |
| 41 | ESH-119 |

**Elemental analysis and Mass spectroscopy**

**Table S1:** Compounds’ codes and their corresponding codes in elemental analysis and mass spectroscopy Fig.s.

**Elemental analysis**


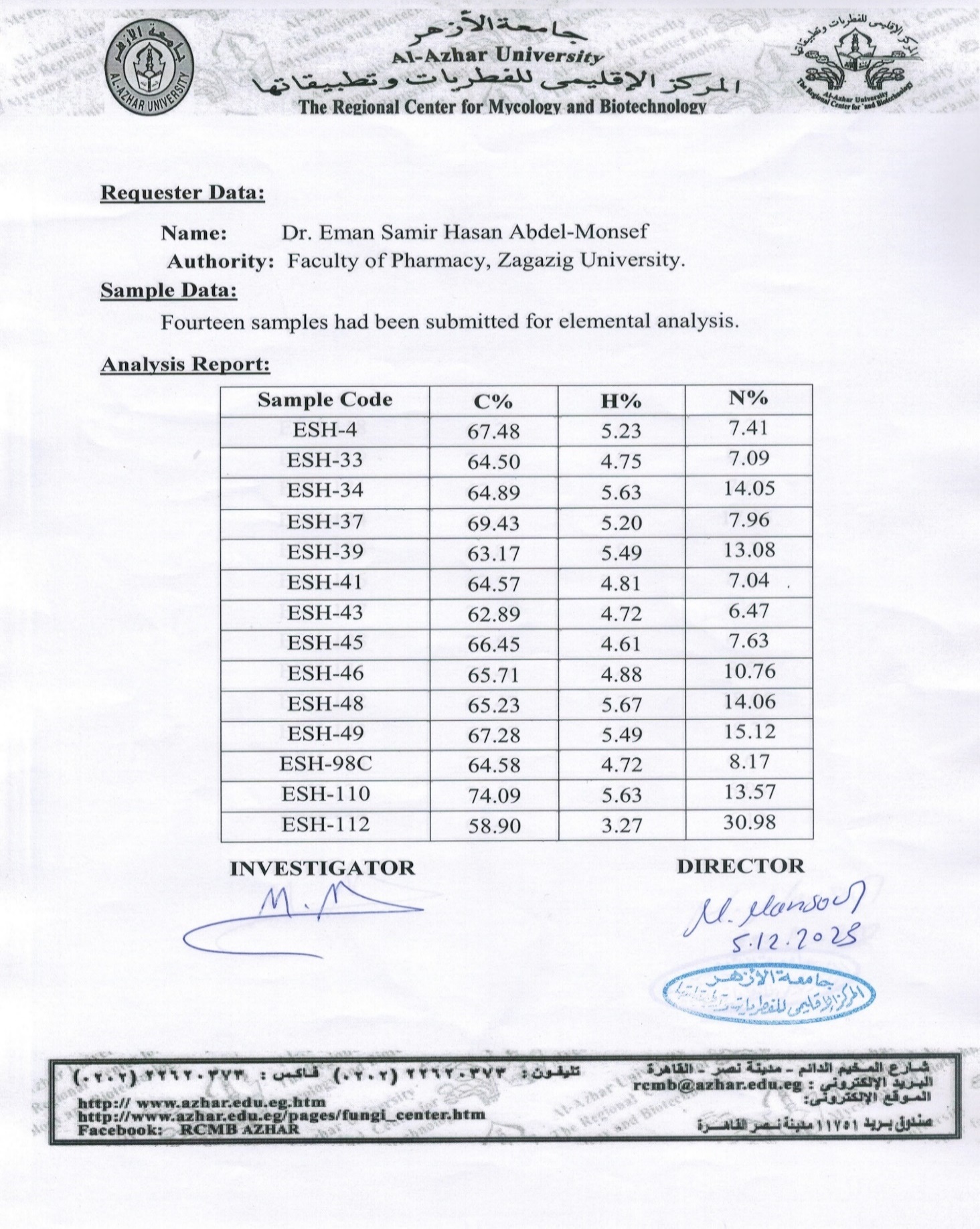


**Fig. S73:** Elemental analysis of compounds 13, 14, 16, 18, 19, 21, 22, 33.


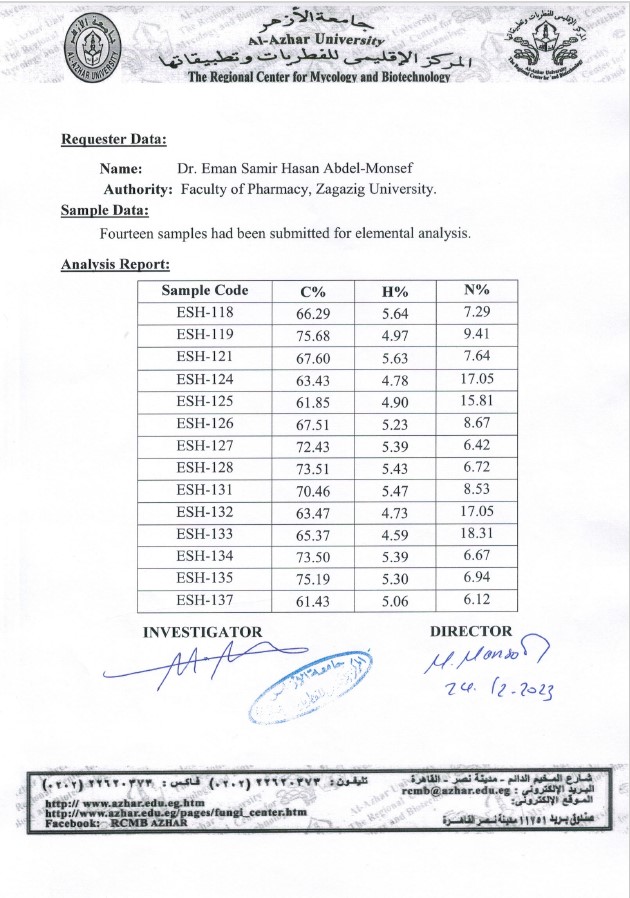


**Fig. S74:** Elemental analysis of compounds 20, 26, 27, 29, 30, 34, 35, 37, 38, 41.


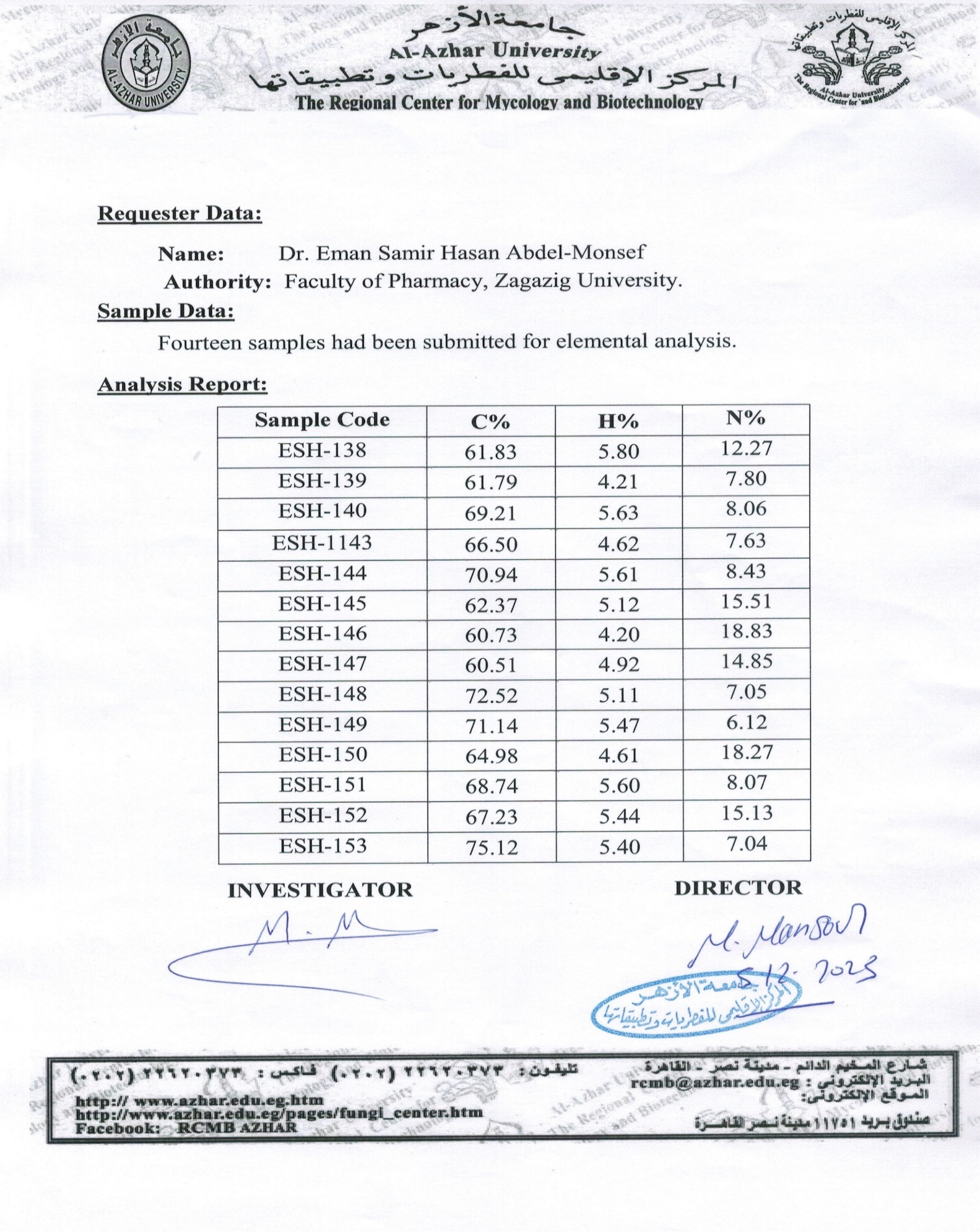


**Fig. S75:** Elemental analysis of compounds 23, 24, 28, 31, 32, 36, 39, 40.

**Mass spectroscopy**

**Fig. S76:** Mass spectrum of compound 13.

**Fig. S77:** Mass spectrum of compound 14.

**Fig. S78:** Mass spectrum of compound 16.

**Fig. S79:** Mass spectrum of compound 18.

**Fig. S80:** Mass spectrum of compound 19.

**Fig. S81:** Mass spectrum of compound 20.

**Fig. S82:** Mass spectrum of compound 21.

**Fig. S83:** Mass spectrum of compound 22.

**Fig. S84:** Mass spectrum of compound 23.

**Fig. S85:** Mass spectrum of compound 24.

**Fig. S86:** Mass spectrum of compound 26.

**Fig. S87:** Mass spectrum of compound 27.

**Fig. S88:** Mass spectrum of compound 28.

**Fig. S89:** Mass spectrum of compound 29.

**Fig. S90:** Mass spectrum of compound 30.

**Fig. S91:** Mass spectrum of compound 31.

**Fig. S92:** Mass spectrum of compound 32.

**Fig. S93:** Mass spectrum of compound 33.

**Fig. S94:** Mass spectrum of compound 34.

**Fig. S95:** Mass spectrum of compound 35.

**Fig. S96:** Mass spectrum of compound 36.

**Fig. S97:** Mass spectrum of compound 37.

**Fig. S98:** Mass spectrum of compound 38.

**Fig. S99:** Mass spectrum of compound 39.

**Fig. S100:** Mass spectrum of compound 40.

**Fig. S101:** Mass spectrum of compound 41.

**IR spectra**


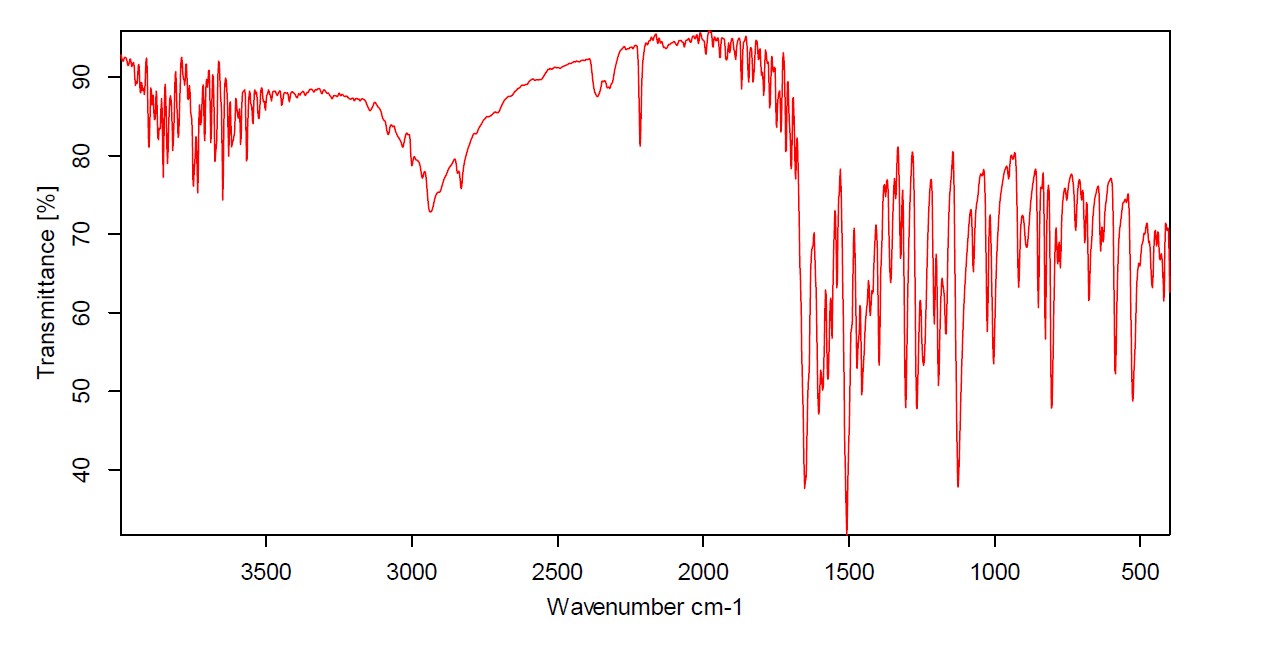


**Fig. S102:** IR spectrum of compound 10


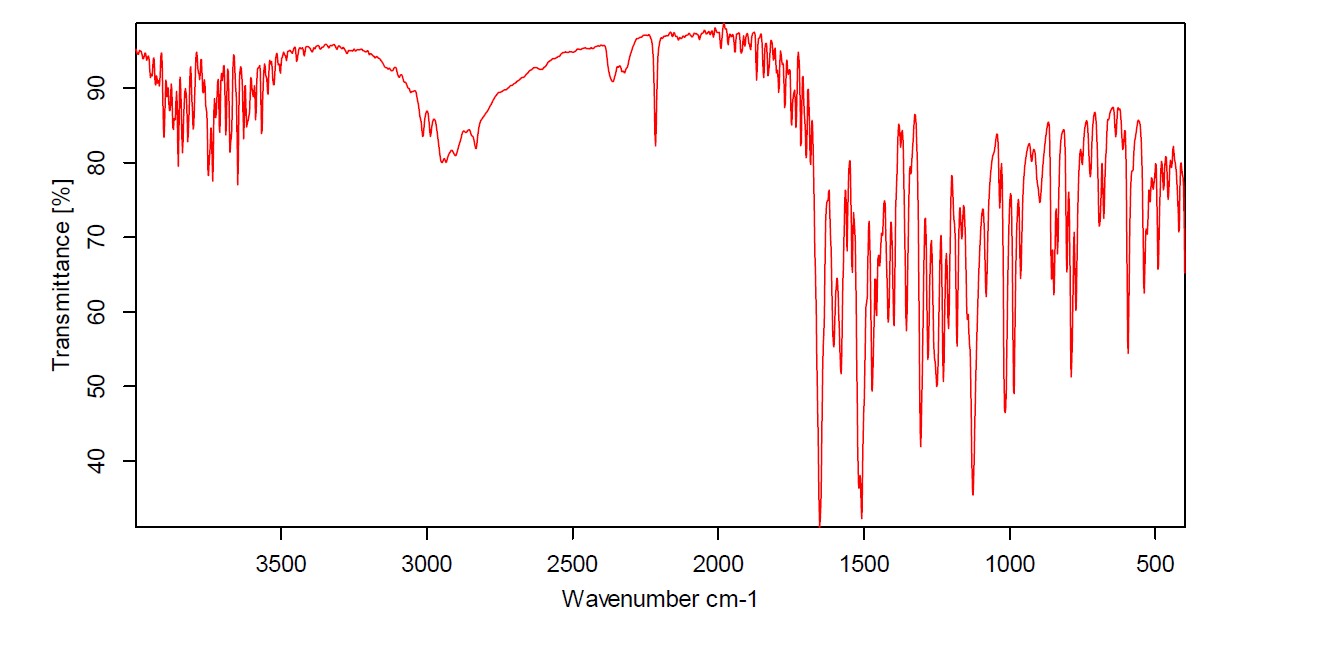


**Fig. S103:** IR spectrum of compound 11.


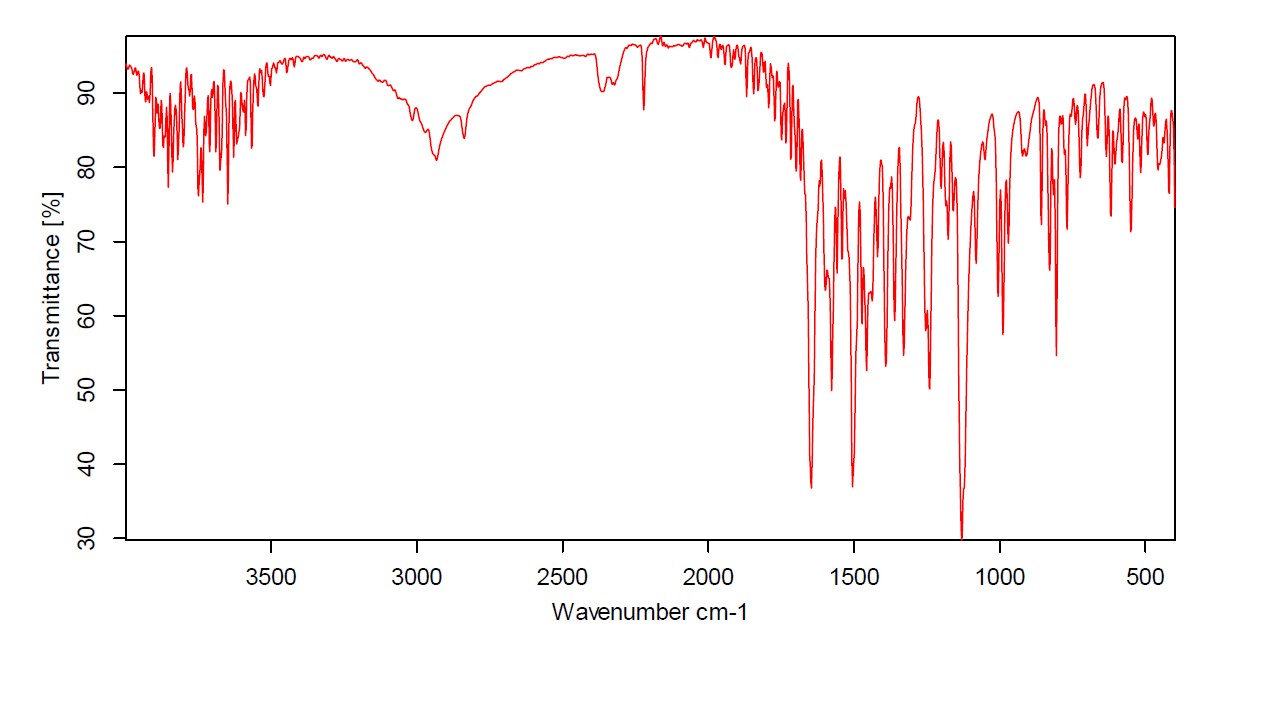


**Fig. S104:** IR spectrum of compound 12.


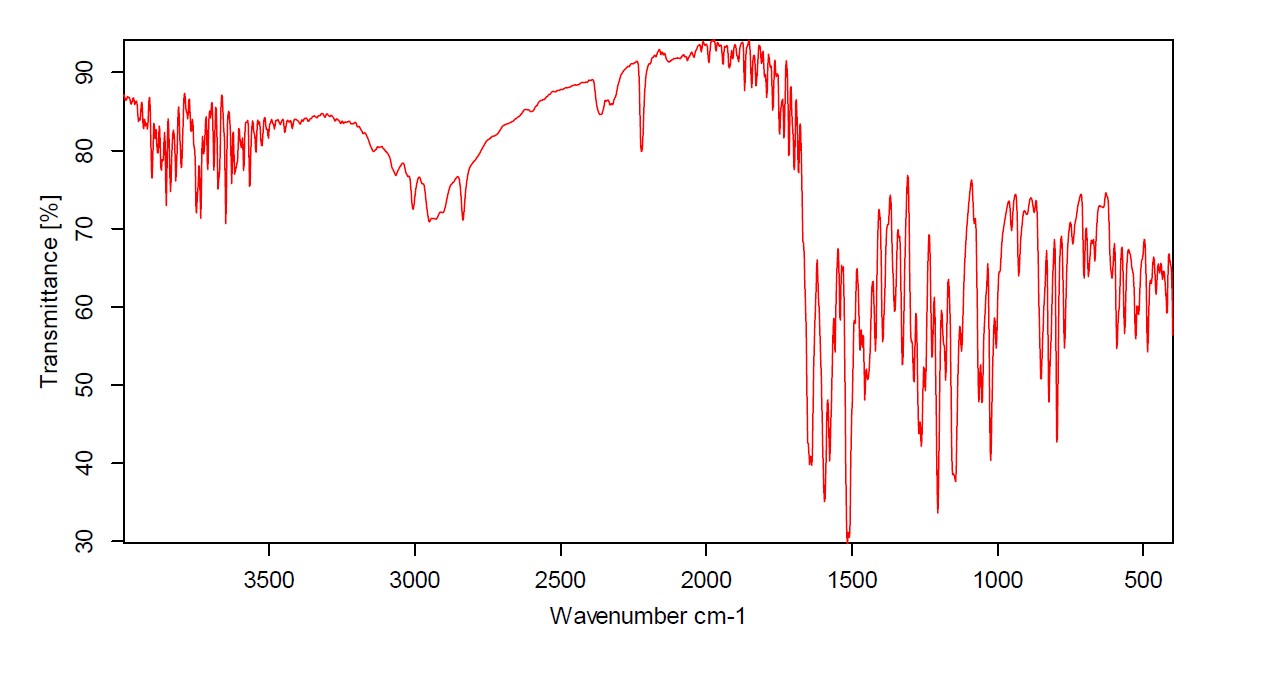


**Fig. S105:** IR spectrum of compound 13.


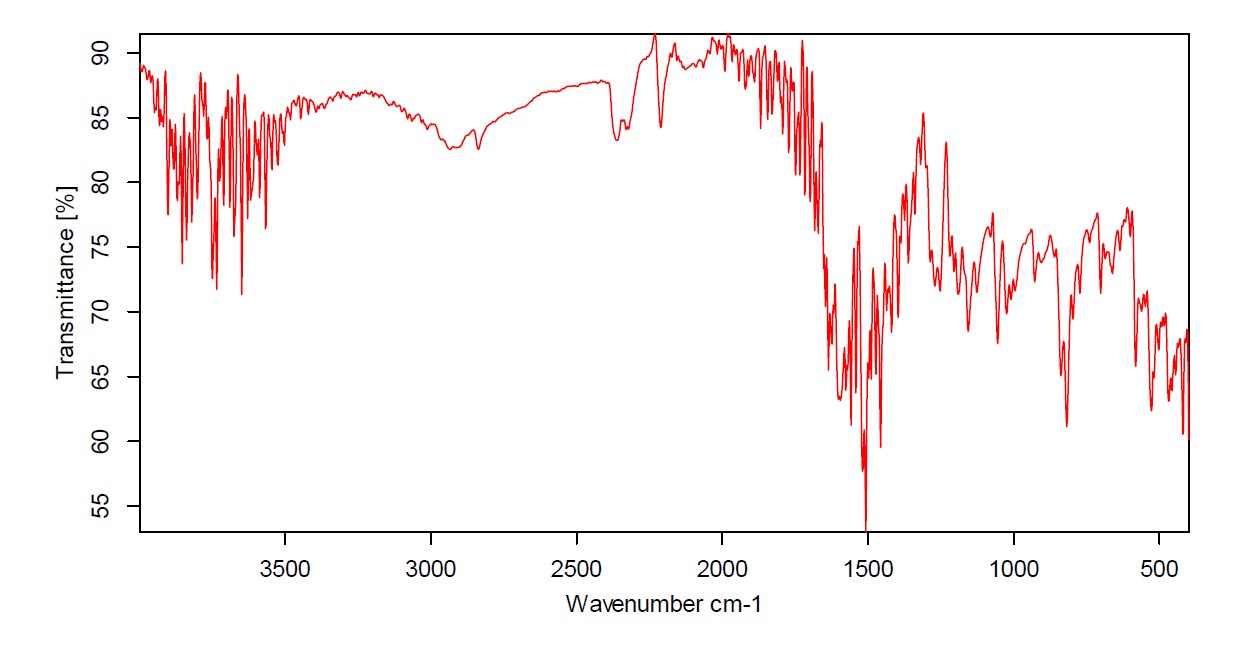


**Fig. S106:** IR spectrum of compound 14.


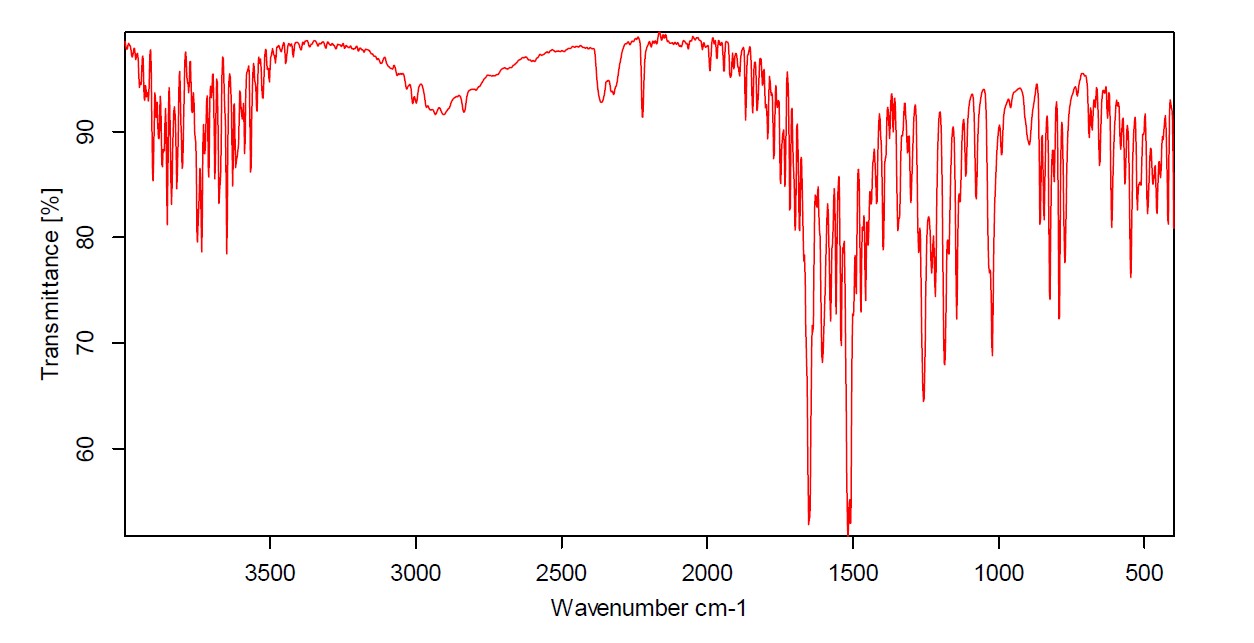


**Fig. S107:** IR spectrum of compound 15.


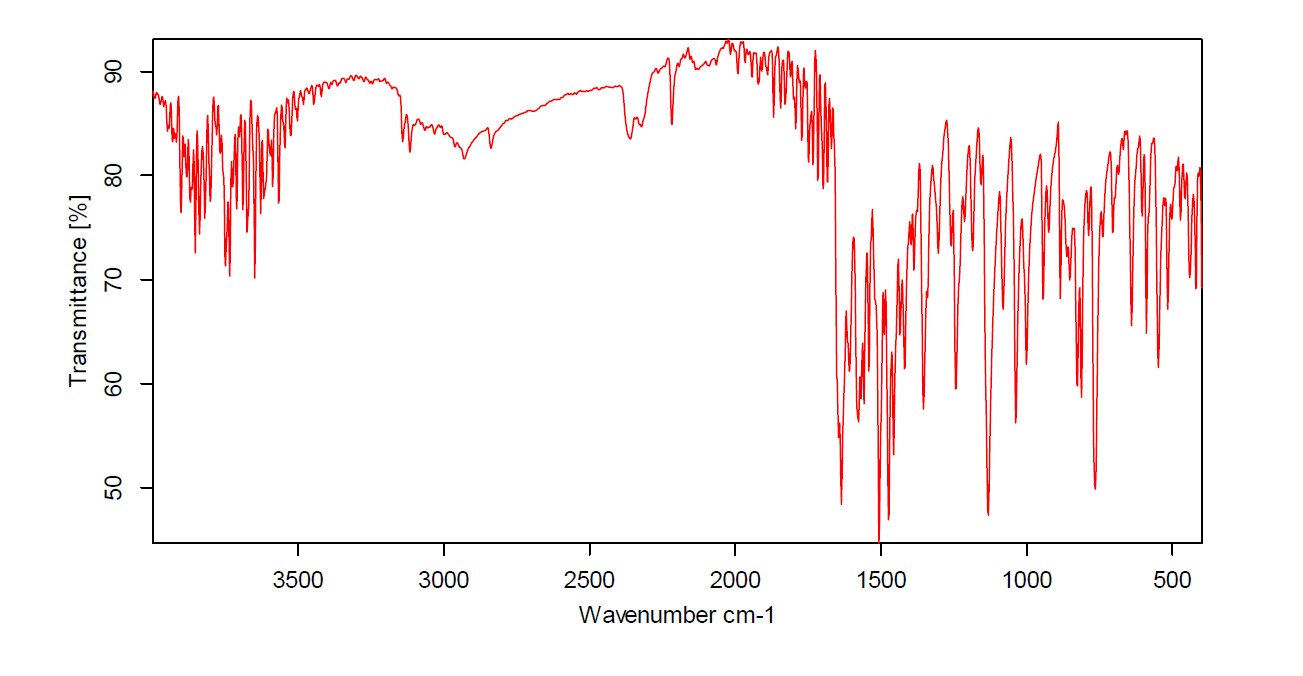


**Fig. S108:** IR spectrum of compound 16.


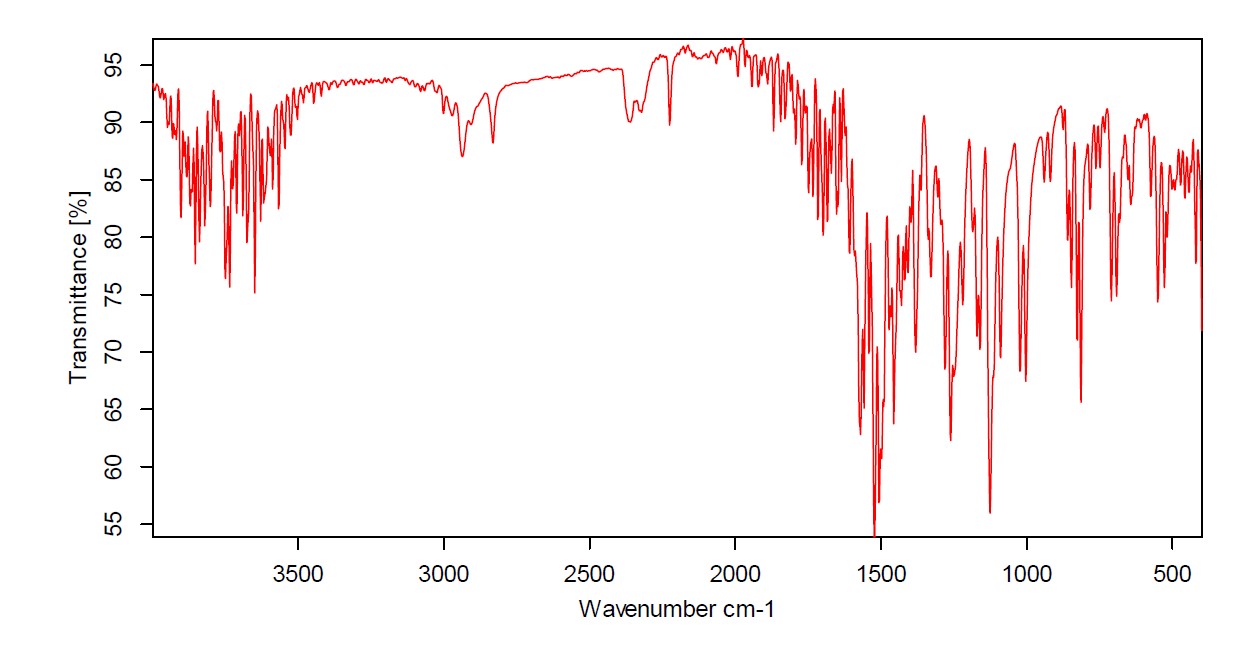


**Fig. S109:** IR spectrum of compound 18.


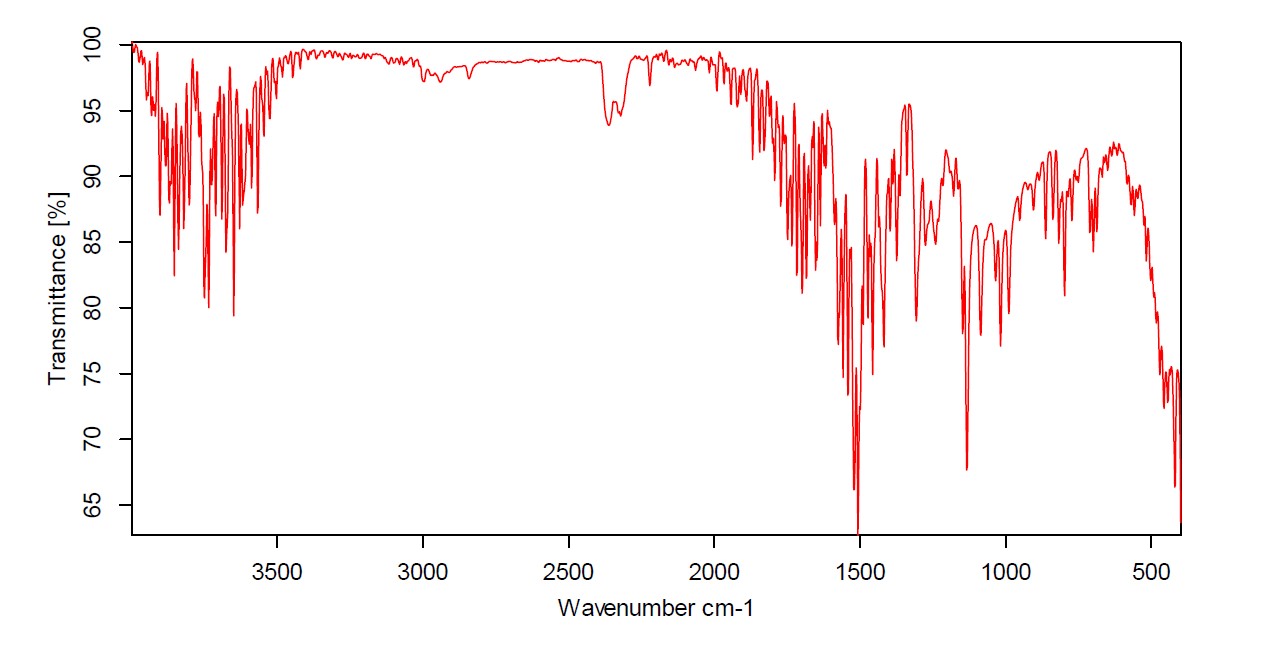


**Fig. S110:** IR spectrum of compound 19.


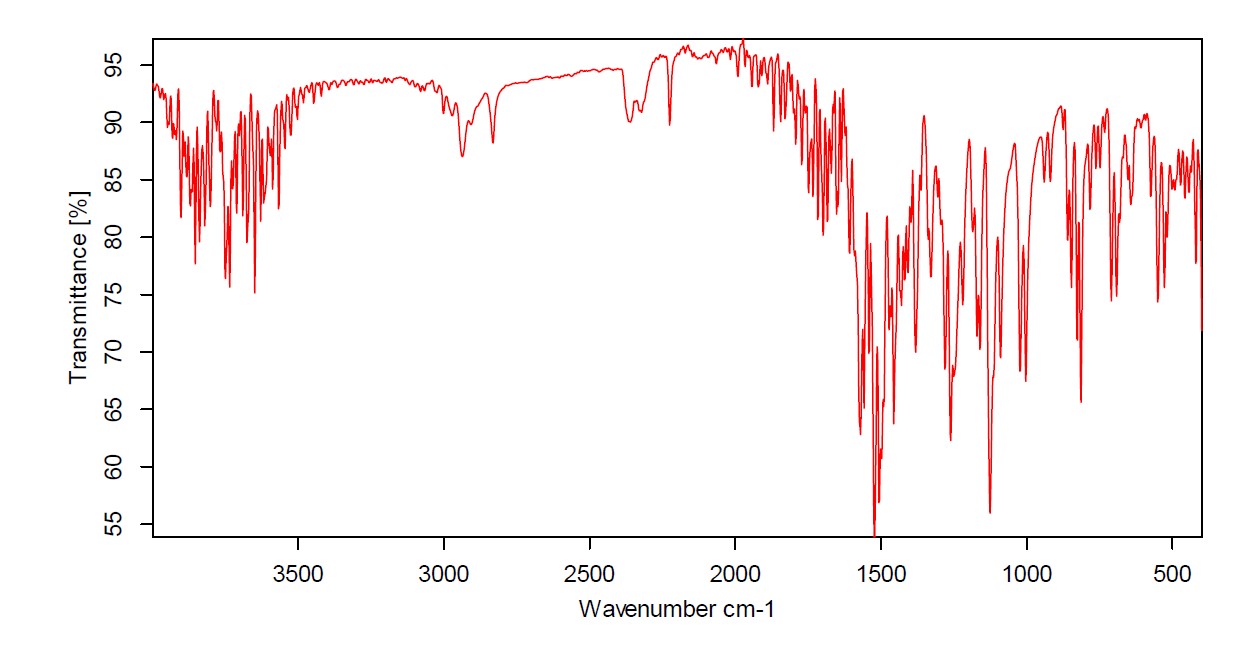


**Fig. S111:** IR spectrum of compound 20.


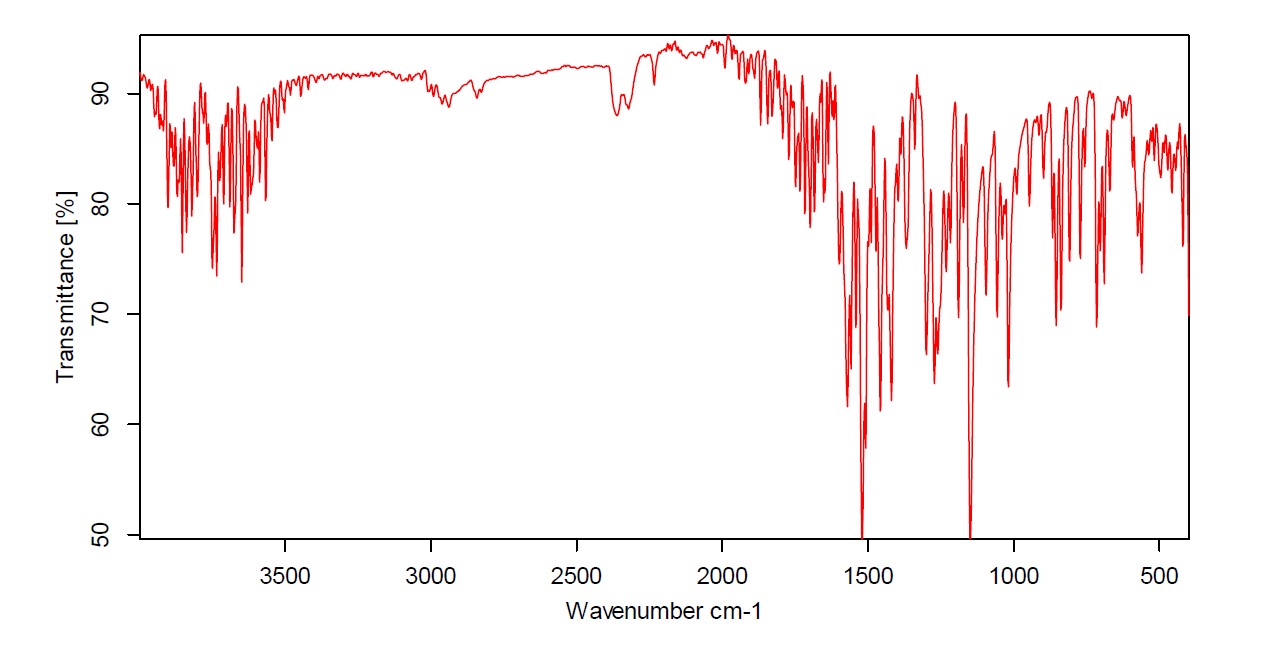


**Fig. S112:** IR spectrum of compound 21.


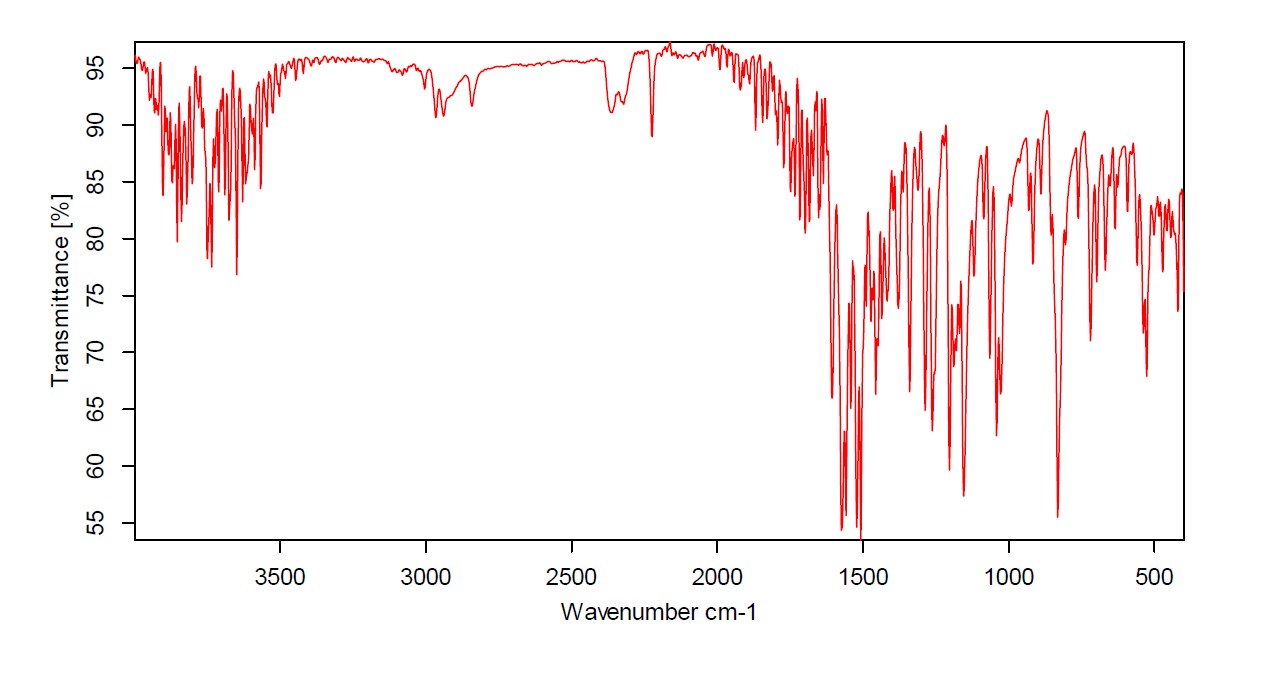


**Fig. S113:** IR spectrum of compound 22.


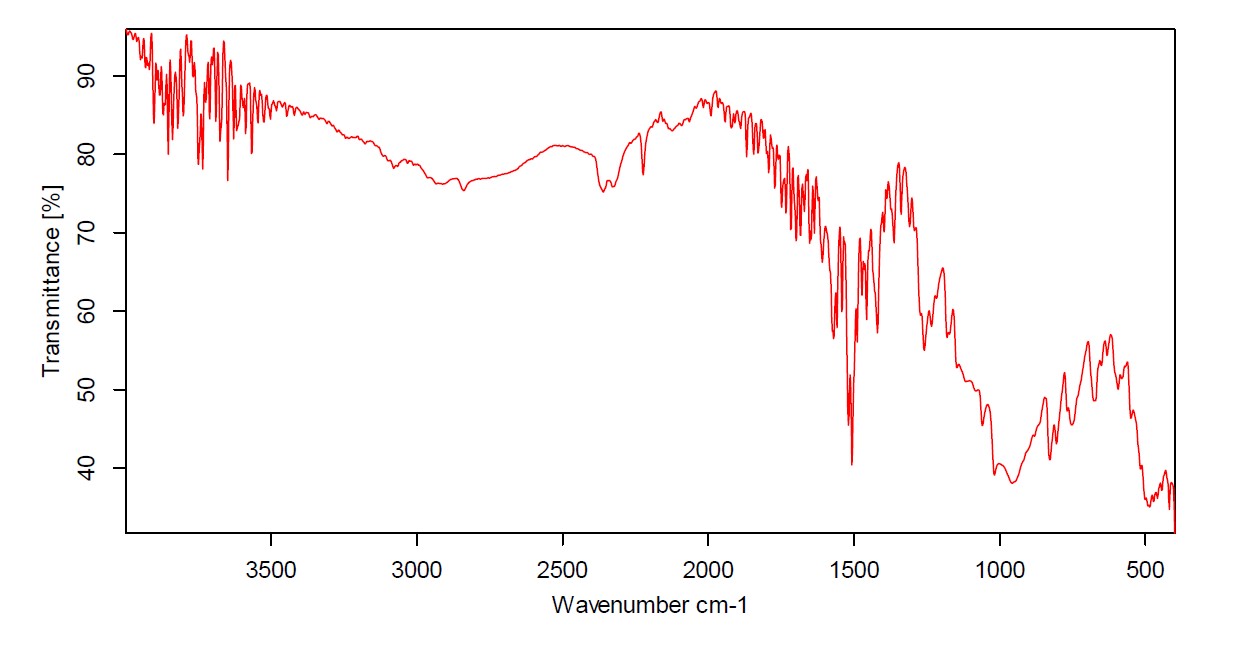


**Fig. S114:** IR spectrum of compound 23.


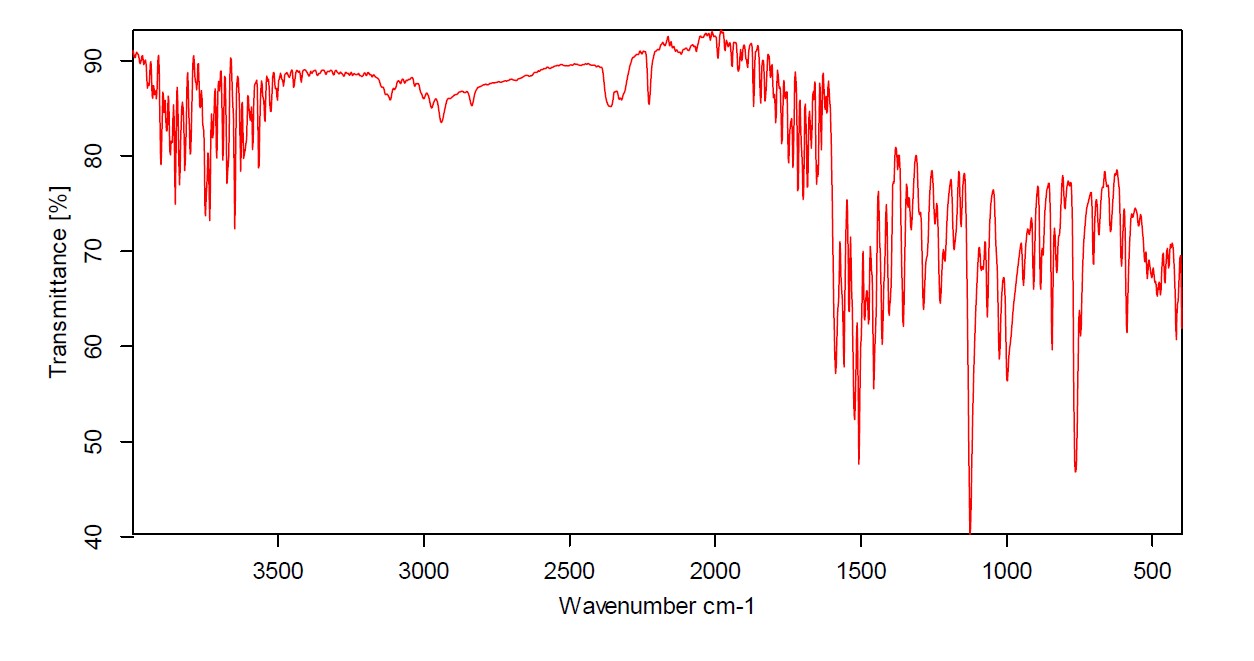


**Fig. S115:** IR spectrum of compound 24.


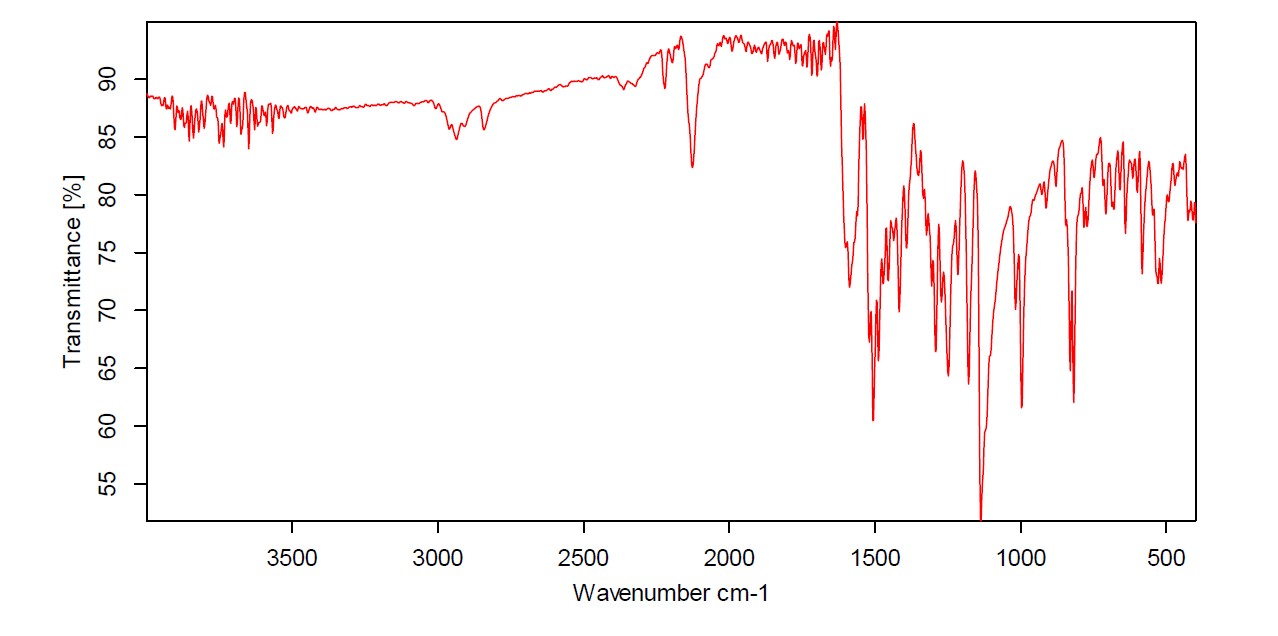


**Fig. S116:** IR spectrum of compound 26.


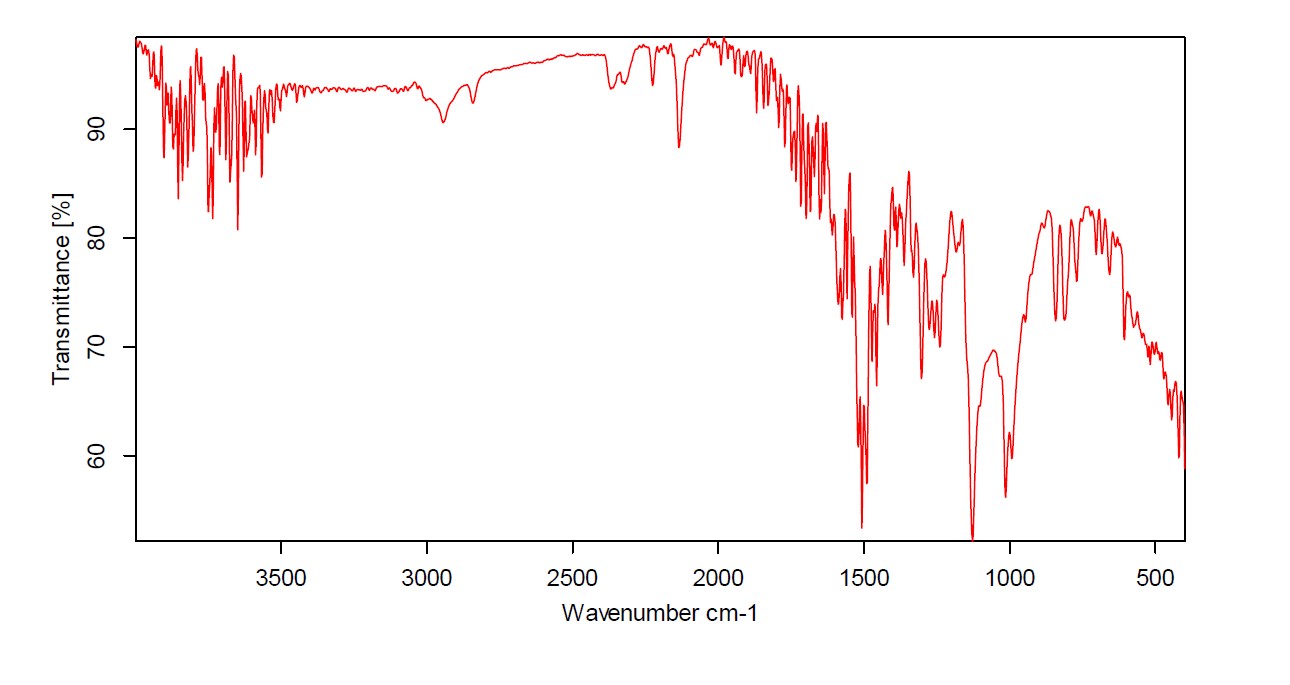


**Fig. S117:** IR spectrum of compound 27.


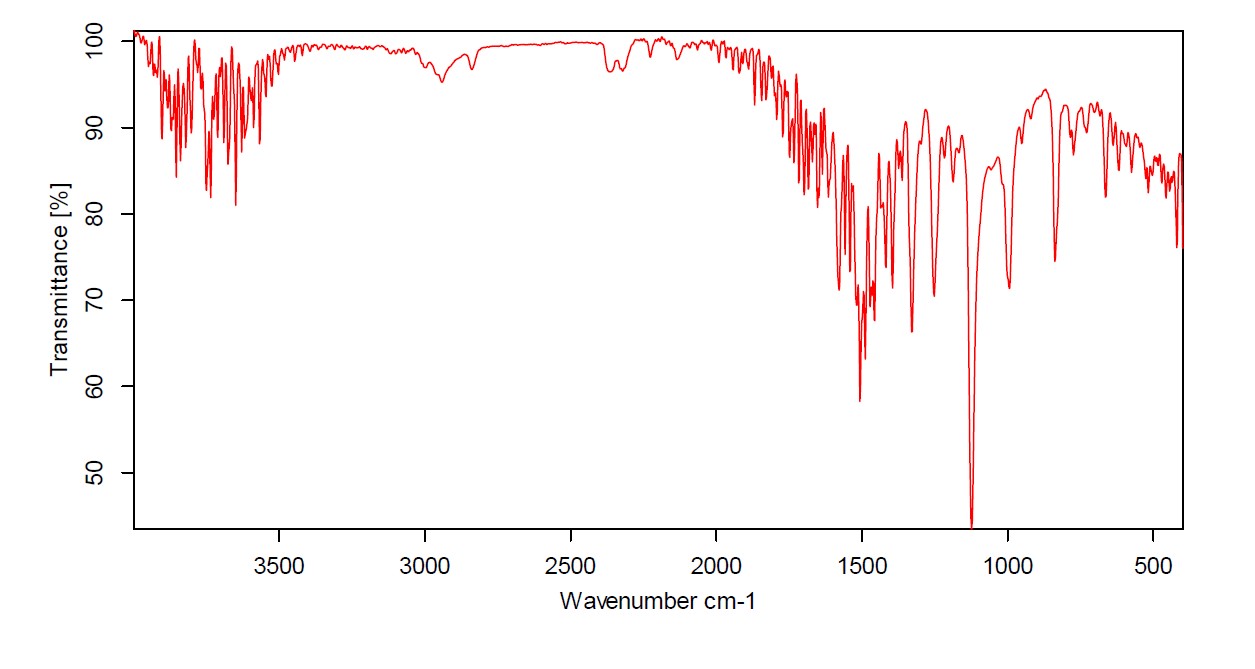


**Fig. S118:** IR spectrum of compound 28.


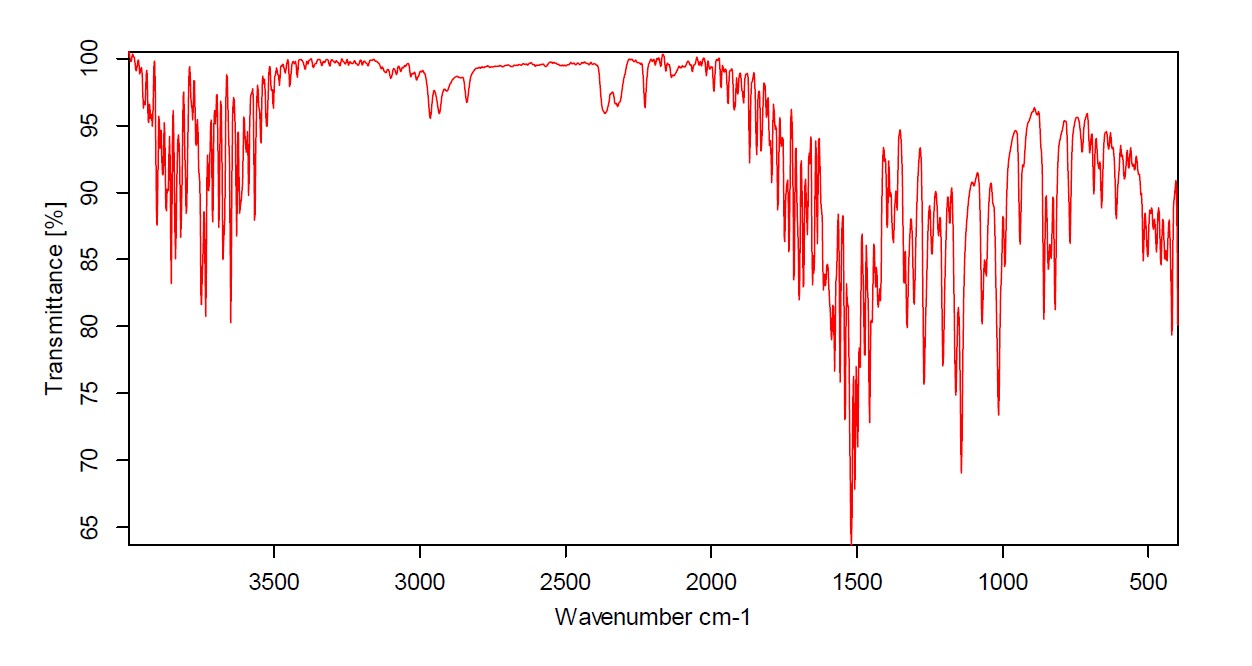


**Fig. S119:** IR spectrum of compound 29.


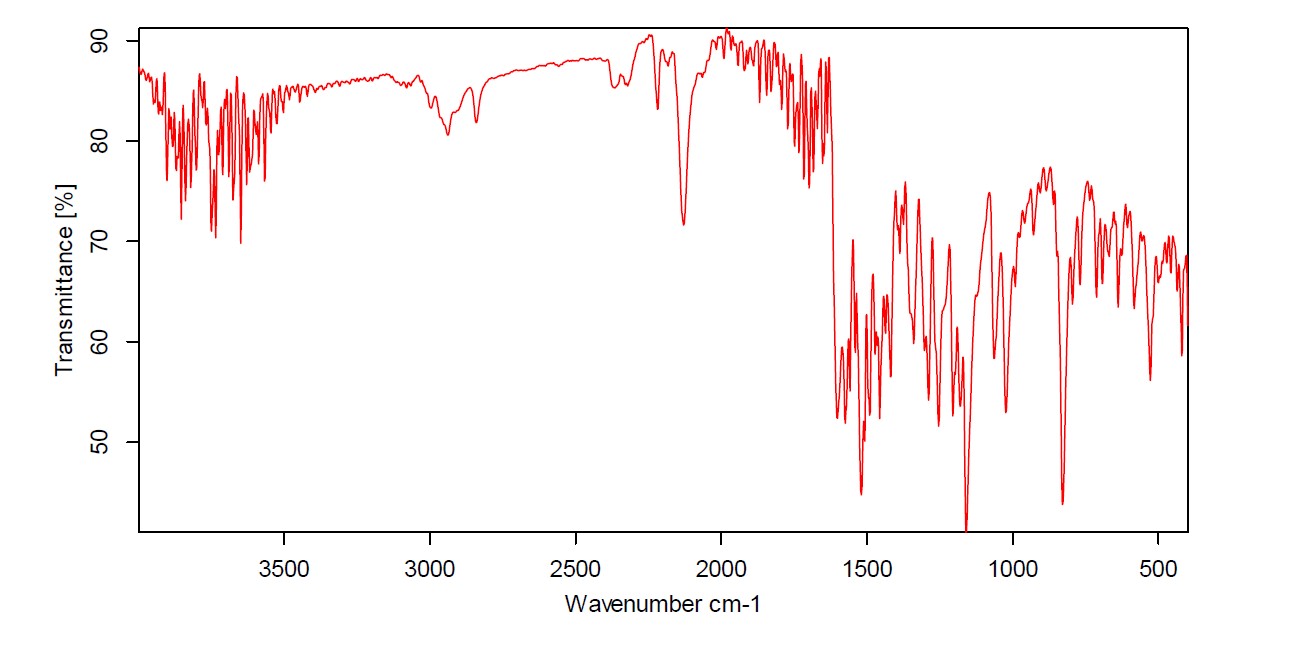


**Fig. S120:** IR spectrum of compound 30.


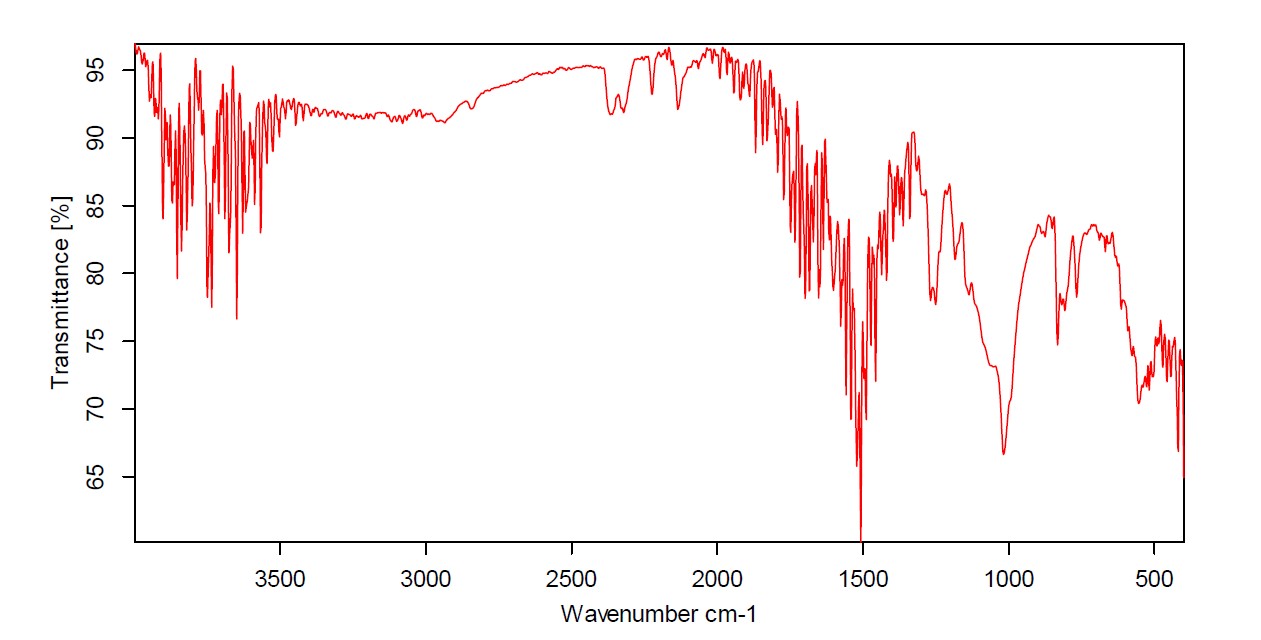


**Fig. S121:** IR spectrum of compound 31.


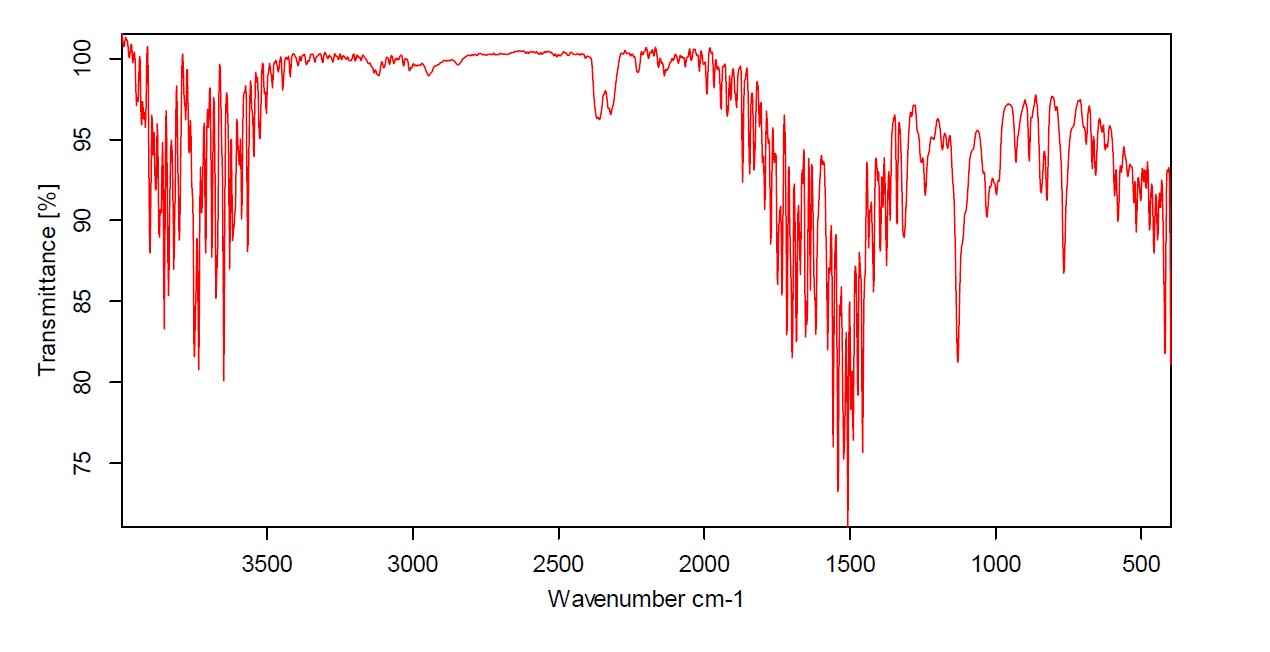


**Fig. S122:** IR spectrum of compound 32.


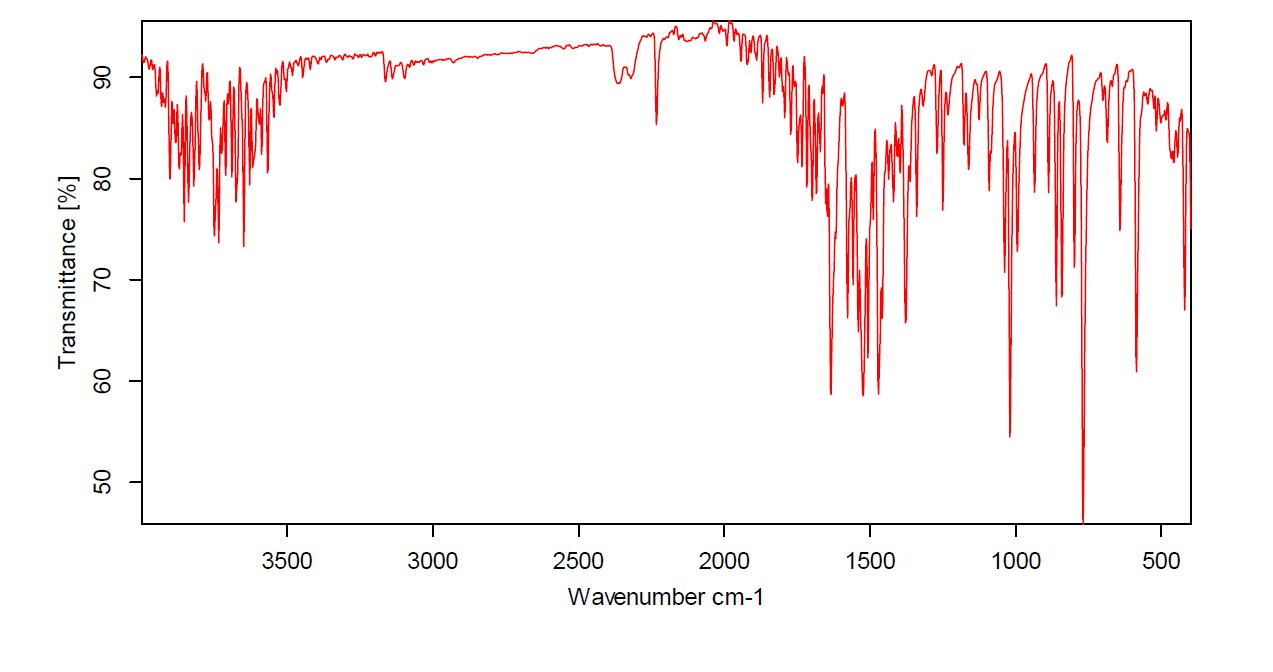


**Fig. S123:** IR spectrum of compound 33.


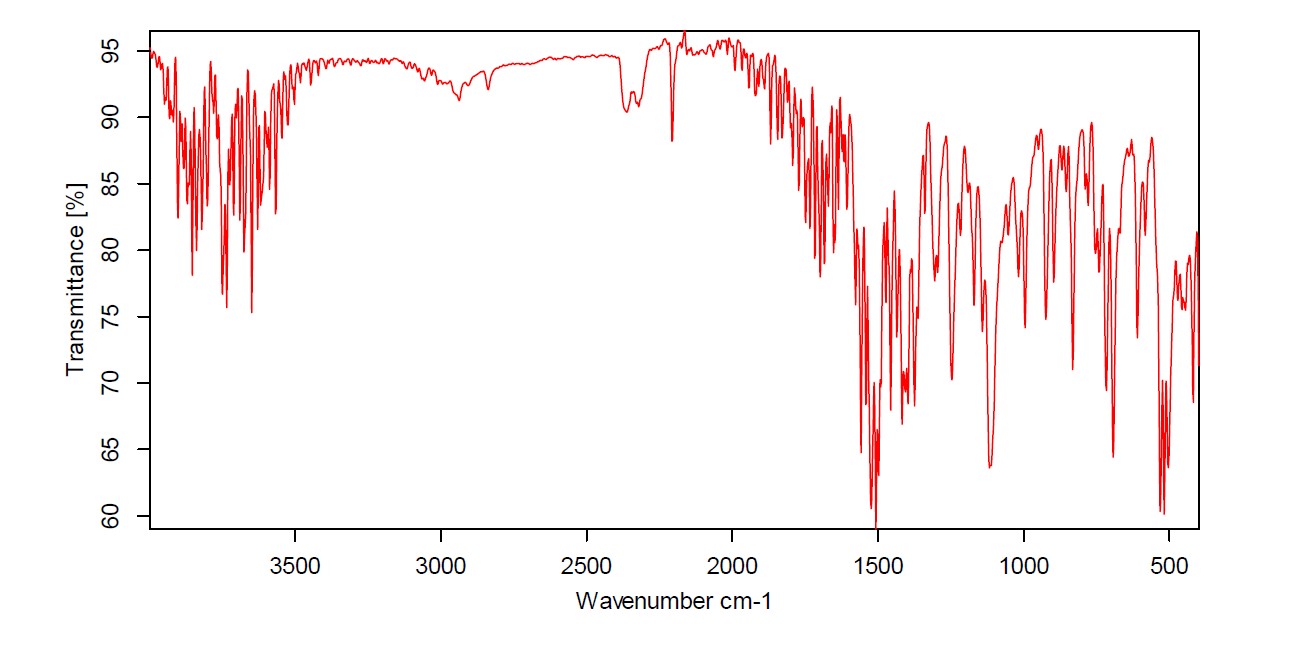


**Fig. S124:** IR spectrum of compound 34.


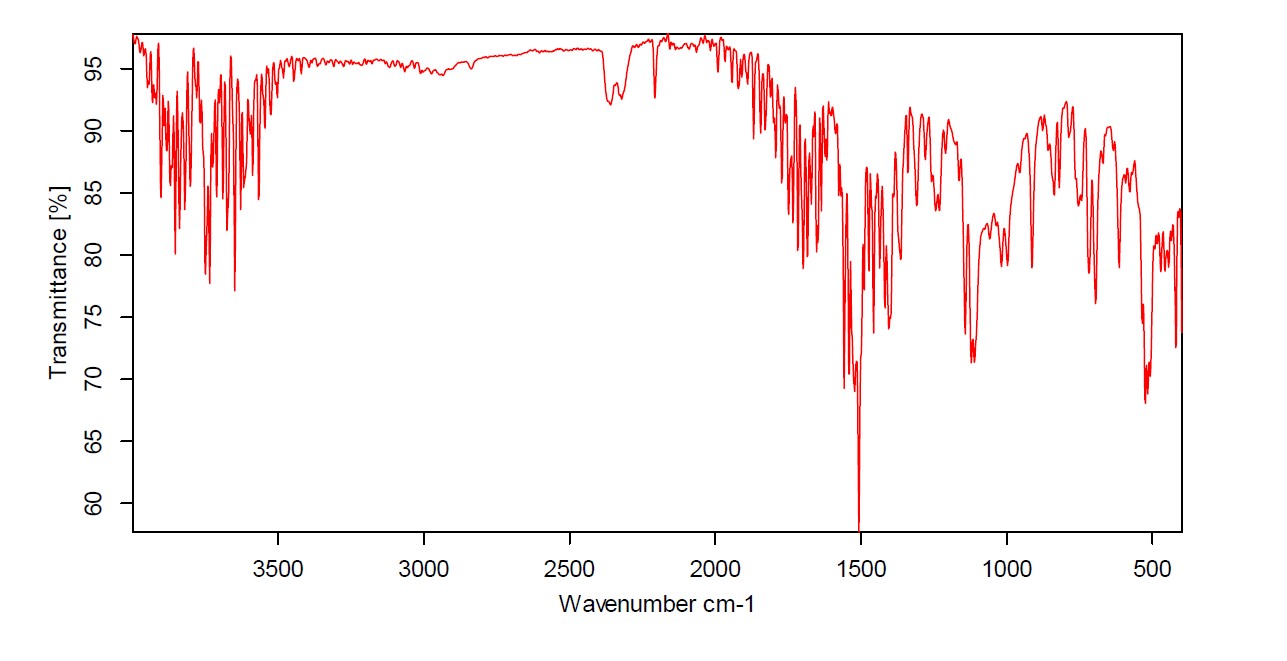


**Fig. S125:** IR spectrum of compound 35.


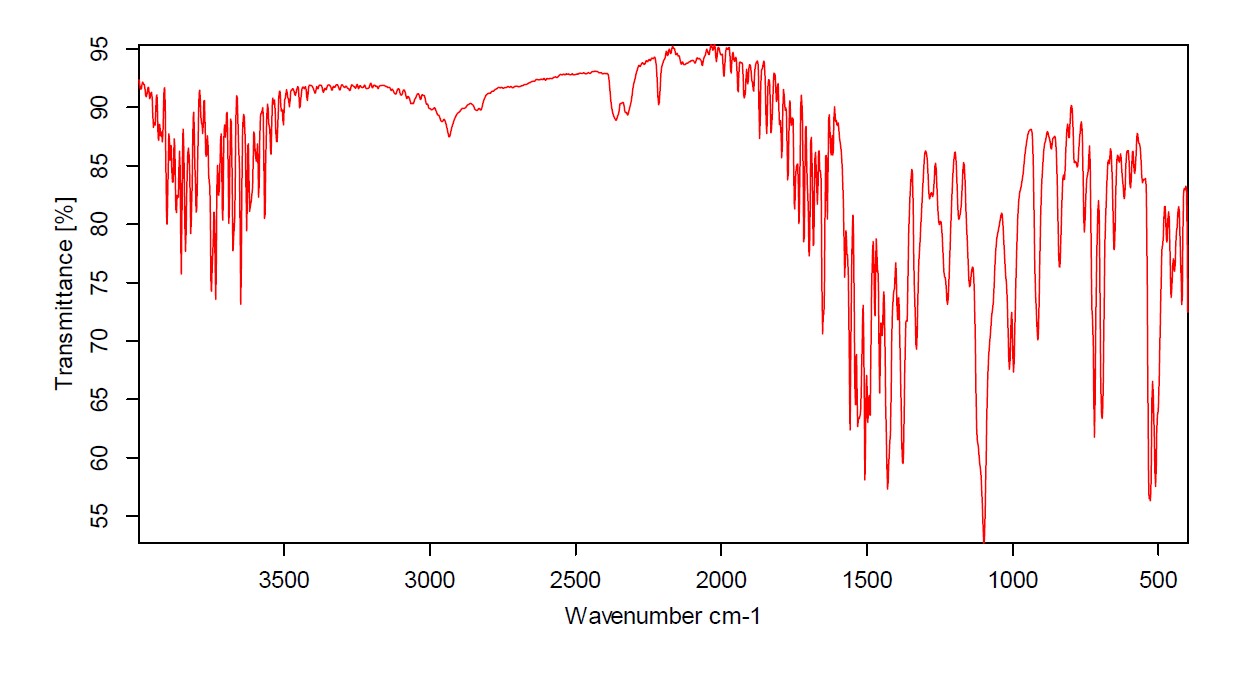


**Fig. S126:** IR spectrum of compound 36.

**Fig. S127:** IR spectrum of compound 37.

**Fig. S128:** IR spectrum of compound 38.

**Fig. S129:** IR spectrum of compound 39.

**Fig. S130:** IR spectrum of compound 40.

**Fig. S131:** IR spectrum of compound 41.

**UV-Vis analyses**

**Fig. S132:** UV spectrum of compound 10.

**Fig. S133:** UV spectrum of compound 11.

**Fig. S134:** UV spectrum of compound 12.

**Fig. S135:** UV spectrum of compound 13.

**Fig. S136:** UV spectrum of compound 14.

**Fig. S137:** UV spectrum of compound 15.

**Fig. S138:** UV spectrum of compound 16.

**Fig. S139:** UV spectrum of compound 18.

**Fig. S140:** UV spectrum of compound 19.

**Fig. S141:** UV spectrum of compound 20.

**Fig. S142:** UV spectrum of compound 21.

**Fig. S143:** UV spectrum of compound 22.

**Fig. S144:** UV spectrum of compound 23.

**Fig. S145:** UV spectrum of compound 24.

**Fig. S146:** UV spectrum of compound 26.

**Fig. S147:** UV spectrum of compound 27.

**Fig. S148:** UV spectrum of compound 28.

**Fig. S149:** UV spectrum of compound 29.

**Fig. S150:** UV spectrum of compound 30.

**Fig. S151:** UV spectrum of compound 31.

**Fig. S152:** UV spectrum of compound 32.

**Fig. S153:** UV spectrum of compound 33.

**Fig. S154:** UV spectrum of compound 34.

**Fig. S155:** UV spectrum of compound 35.

**Fig. S156:** UV spectrum of compound 36.

**Fig. S157:** UV spectrum of compound 37.

**Fig. S158**: UV spectrum of compound 38.

**Fig. S159:** UV spectrum of compound 39.

**Fig. S160:** UV spectrum of compound 40.

**Fig. S161:** UV spectrum of compound 41.

**S4.2.6. In silico study**

**S4.2.6.1. Density Functional Theory (DFT) Calculations:**

All quantum chemical calculations were performed using the Schrödinger Maestro 2025-2 suite with the Jaguar module. Initial geometries of the azido and tetrazolo tautomeric forms were built and pre-optimized in Maestro. Full geometry optimizations were then carried out at the B3LYP-D3/6-31G** level of theory with implicit solvation in water using the polarizable continuum model (PCM). Single-point energy refinements were subsequently performed at the B3LYP-D3/6-31+G** level of theory in the same solvent model (PCM, water), using the optimized geometries from the prior step.

Relative free energies (ΔG, kcal·mol⁻¹) between azide and tetrazole forms were calculated as the electronic energy difference between tautomeric pairs, converted from Hartree to kcal·mol⁻¹ (1 Ha = 627.5095 kcal·mol⁻¹).

Equilibrium constants (K) were determined using the Boltzmann relationship:

*K* = $e^{-\Delta G/RT}$

Where R = 1.987 cal K^-1^ mol^-1^ and T = 298 K

Population distributions (%) for each tautomer were derived from the Boltzmann weights of the two-state system. All reported values correspond to solution-phase free energies at 298 K.

This was done on three compounds (26, 27, 33) containing an azido-functional group with an ortho-pyridyl nitrogen, enabling the tautomerization into a tetrazole form

Raw Data (after B3LYP-D3/6-31G** geometry optimizations):

| **compound** | **Solution-phase energy (Hartree)** |
| --- | --- |
| 33 (Az) | -722.331647 |
| 33 (Tet) | -722.329822 |
| 26 (Az) | -1424.437278 |
| 26 (Tet) | -1424.439053 |
| 27 (Az) | -1538.965887 |
| 27 (Tet) | -1538.966820 |

**Table S2.** Solution-phase energies in Hartree after geometry optimization (26, 27, 33)

**Sample Calculation of relative free energies and tautomeric populations (Az vs. Tet):**

**Input data (solution-phase single-point energies, Hartree):**

- Azide 26: E_Az_ = -1424.437278 Ha
- Tetrazole 26: E_Tet_ = -1424.439053 Ha

**Step 1. Energy difference (Hartree):**

ΔE = E_Tet_ - E_Az_ = -1424.439053 - (- 1424.437278) = - 0.001775 Ha

**Step 2. Convert to kcal mol⁻¹:**

Conversion factor: 1 Ha = 627.5095 kcal mol^-1^

ΔG = -0.001775 Ha x 627.5095 = - 1.11 kcal mol^-1^

**Step 3. Convert to kJ mol⁻¹:**

ΔG = -1.11 kcal mol^-1^ x 4.184 kJ mol^-1^ = - 4.66 kJ mol^-1^

**Step 4. Equilibrium constant (at 298 K):**

*K* = $e^{-\Delta G/RT}$

Where R = 1.987 cal K^-1^ mol^-1^ and T = 298 K

K = e^−(−1.11/0.5929)^ = 6.55

**Step 5. Population distribution:**

P_Az_ = 1/(1+K) = 1/(1+6.55) = 0.132 = 13.2%

P_Tet_ = 100% - 13.2% = 86.8%

- ΔG = −1.11 kcal·mol⁻¹ (tetrazole lower)
- Populations: Azide 13.2%, Tetrazole 86.8%

**26 (Tet)**

**26 (Az)**

**27 (Tet)**

**27 (Az)**

**33 (Tet)**

**33 (Az)**

**Fig. S162:** DFT-based low energy conformations of azide/tetrazole tautomers

**S4.2.6.2. Computational Modelling & Docking:**

The computational studies described involved the use of Schrödinger’s Maestro v14.2.1 (2025-3) with access provided under a single academic user license. Calculations used the following modules: ConfGen, Epik, Glide, LigPrep, MacroModel, Prime, ProtPrep, SiteMap and the Optimized Potential for Liquid Simulations (OPLS5e) force field. A pH of 7.00 ± 2.00 was set when appropriate along with the use of H_2_O (solvation parameter). Other software used include ChemDraw Professional v25.0 and the RSCB protein data bank ([www.rcsb.org/](http://www.rcsb.org/)).

To start, three relevant target crystals were selected and downloaded from the PDB. These included human TopII ATPase domain bound to AMP-PNP (PDB 1ZXM), human TopII in complex with DNA and anti-cancer agent etoposide (PDB 3QX3), and the tubulin-RB3-SLD-TTL complex bound to ABI-274. These three proteins underwent a standard ProtPrep workflow, which adds hydrogens, assigns bond orders, and optimizes H-bond networks. Ramachandran plots confirmed appropriate protein backbone geometries (Fig. S162). This step was followed by a standard receptor grid generation workflow, where the relative bound ligands (AMP-PMP, etoposide, ABI-274) were used as the center coordinate to produce a relative docking grid (10x10x10 Å). Once completed, the targets were now prepared for subsequent docking studies. The various ligands of interest: 20, 22, 24, 26, 27, 33, 35, 37, 38, 41 (and 26_tet, 27_tet, 33_tet tautomers) were manually drawn using the Maestro 2D sketcher module before a LigPrep workflow, involving low energy minimizations, Epik ionization step (pH 7.00 ± 2.00), adding hydrogens, assigning bond orders, checking tautomers, and generating relevant physiological states.

At this stage, the prepared ligands underwent a standard Glide docking workflow using Maestro’s OPLS force-field (Extra Precision) looking at the top 10 binding poses across the generated grids of TopII (catalytic site), TopII (DNA interface), and Tubulin (colchicine-binding site). In each case, a quantitative docking score (in kcal/mol) was calculated to prioritize ligands. This was followed by visual inspection of poses to analyze intermolecular forces driving molecular recognition. A doxorubicin control was used in all dockings against the 3 targets.

**TopII-DNA (3QX3)**

**Tubulin (6PC4)**

**TopII (1ZXM)**

**Fig. S163:** Ramachandran plots of TopII ATPase (1ZXM), TopII-DNA (3QX3), and Tubulin (6PC4) highlighting optimal protein preparation via phi/psi angles. In all three cases, >95% of residues are in the allowed regions. Any residue outside represents flexible amino acids: Gly, Pro, Pre-Pro, Pre-Gly.

**Fig. S164:** Docking scores of ligands against TopII (1ZXM), TopII-DNA (3QX3), and tubulin (6PC4).
